# Electrophoretic-deposited MXene titanium coatings in regulating bacteria and cell response for peri-implantitis

**DOI:** 10.3389/fchem.2022.991481

**Published:** 2022-09-29

**Authors:** Si Huang, Yu Fu, Anchun Mo

**Affiliations:** ^1^ State Key Laboratory of Oral Diseases, National Clinical Research Center for Oral Diseases, Sichuan University, Chengdu, China; ^2^ Department of Implantology, West China Hospital of Stomatology, Sichuan University, Chengdu, China

**Keywords:** Ti_3_C_2_T_x_ coating, MRSA, implant-associated infections, antibacterial ability, cell behavior, nano-biomaterials

## Abstract

**Background:** Two-dimensional(2D)MXenes have continued to receive increasing interest from researchers due to their graphene-like properties, in addition to their versatile properties for applications in electronic devices, power generation, sensors, drug delivery, and biomedicine. However, their construction and biological properties as titanium coatings to prevent peri-implantitis are still unclear.

**Materials and methods:** In this work, few-layer Ti_3_C_2_T_x_ MXene coatings with different thicknesses at varied depositing voltages (30, 40, and 50 V) were constructed by anodic electrophoretic deposition without adding any electrolytic ions. *In vitro* cytocompatibility assay was performed on preosteoblasts (MC3T3-E1) cell lines after the characterization of the coating. Meanwhile, the antibacterial activity against bacteria which are closely related to peri-implantitis including *Staphylococcus aureus (S. aureus)* and its drug-resistant strain *MRSA* was further investigated.

**Results:** MXene-coated titanium models with different thicknesses were successfully assembled by analyzing the results of characterization. The compounding of Ti_3_C_2_T_x_ could significantly improve the initial adhesion and proliferation of MC3T3-E1 cells. Moreover, the coating can effectively inhibit the adhesion and cell activity of *S. aureus* and *MRSA*, and *MRSA* expressed greater restricting behavior than *S. aureus*. The ability to promote antibacterial activity is proportional to the content of Ti_3_C_2_T_x_. Its antioxidant capacity to reduce ROS in the culture environment and bacterial cells was first revealed.

**Conclusion:** In summary, this work shows a new avenue for MXene-based nano-biomaterials under the clinical problem of multiple antibiotic resistance.

## Graphical Abstract

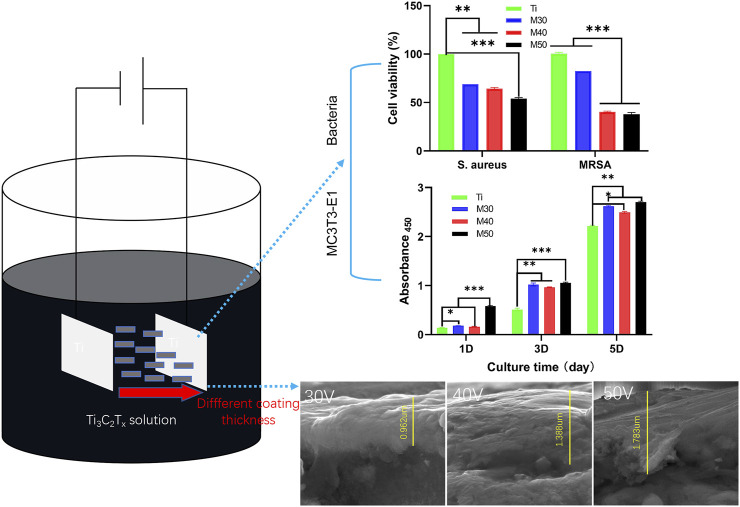

## 1 Introduction

Peri-implantitis, a biological complication mediated by impaired immunity at the implant/tissue interface, is attributed to bacterial biofilms on the implant surface (Kotsakis and Olmedo, 2021). The clinical manifestation is severe progressive inflammatory destruction of the peri-implant bone leading to the failure of surgery (Konstantinidis et al., 2015; Safioti et al., 2017; Kotsakis and Olmedo, 2021). Many clinical studies have shown that the Gram-positive (Gram (+)) facultative coccus *Staphylococcus aureus* (*S. aureus*) is the principal pathogen arousing peri-implantitis (Thurnheer and Belibasakis, 2016; Wu et al., 2022). Furthermore, the bacteria that form biofilms on the titanium surface are resistant to antimicrobial agents, particularly to β-lactams (Wu et al., 2022). Methicillin-resistant *Staphylococcus aureus* (*MRSA*) exhibits multidrug resistance to several substances belonging to different antibiotic classes and is prevalent in implant-related infections (Zhou et al., 2022). Undoubtedly, as in other fields of medicine, anti-adherent and bactericidal surfaces to limit the early adhesion and activity of bacteria on titanium implant surfaces are the main research goals (Cacaci et al., 2020). The surface energy, morphology, wettability, wear resistance, and electrochemical charges of a titanium substrate all influence biofilm adhesion and formation (Song et al., 2015). The most universal way to interfere with the early stages of microbial adhesion is to modify the chemical or micro/nanostructure of the out-layer on the titanium substrate via many methods such as sol-dip (Safaee et al., 2021), electrospinning nanotechnology (Zhang et al., 2014), silanization (Qiu et al., 2018), and electrophoretic deposition (EPD) (Qiu et al., 2017). The current superficial modifications include the coatings of surfactants (Vacheethasanee and Marchant, 2000), inorganic nanomaterials (Qiu et al., 2017), proteins (An et al., 1996; Sang et al., 2021), antimicrobial peptides (Hwang et al., 2021), and hydrophilic negatively charged polysaccharides such as heparin and hyaluronic acid (Morra and Cassineli, 1999; Hildebrandt, 2002). However, most surface modification materials usually lack multifunctional properties. In recent years, research studies on titanium surface modification materials with both biomechanical advantages and antibacterial properties have been growing, and the pros and cons of different modification methods of titanium-based surfaces have also attracted extensive attention.

Inorganic 2D MXenes with hexagonal structures similar to graphene (Bennett-Jackson et al., 2019) are made up of transition metal carbides, nitrides, or carbonitrides with a general formula of M_n+1_X_n_T_x_ (*n* = 1–3) in which M is an early transition metal (e.g. Sc, Ti, Zr, Hf, V, Nb, Ta, Cr, and Mo), X is a carbon or nitrogen, and T_x_ represents abundant surface functional groups (–OH, =O, –F, and rarely, –Cl) opposed to the surface of the M layer during the production of MXene by chemically etching the MAX phase (Kang et al., 2019; Bhardwaj et al., 2022). Due to their outstanding properties (i.e., large surface area, great biocompatibility, antibacterial properties, excellent hydrophilicity and electrical conductivity, flexible surface functionalization, and targeted therapeutic properties), MXenes have been diffusely used in the biomedical field (Huang et al., 2018; Zamhuri et al., 2021). In tissue engineering, MXenes can promote the proliferation, differentiation, adhesion, and migration of a variety of tissue-engineered stem cells, such as human mesenchymal stem cells (Jang and Lee, 2021) and human neural stem cells (Guo et al., 2022) without obvious cytotoxicity; Ti_3_C_2_ is the first negatively charged Maxine synthesized by etching the Al layer in Ti_3_AlC_2_ (Naguib et al., 2011). Rasool et al. (2016) found that the antibacterial activity of Ti_3_C_2_T_x_ against Gram (-) *Escherichia coli* and Gram (+) *Bacillus subtilis* was higher than that of the currently widely used 2D antibacterial agent graphene oxide (GO) (Rasool et al., 2017). The order of antibacterial activity against these two strains was monolayer Ti_3_C_2_T_x_ ≫multilayer Ti_3_C_2_T_x_ > Ti_3_AlC_2_. Meanwhile, Jastrzebska et al. (2017) also reported that Ti_2_CT_x_ MXenes lacked bactericidal properties against *Botulinum, S. aureus, and Bacillus*. Mild apoptosis of *Bacillus* occurs only when bacterial cells are drawn into the swelling layer and placed between individual flakes of the expanded TiCT_x_. Thus, the differences in thickness and surface chemistry of MXenes can affect their toxicity and antibacterial activity. At present, the antibacterial applications of Ti_3_C_2_T_x_ mainly focus on composite hydrogels (Yin et al., 2020; Li et al., 2022), composite membranes (Rasool et al., 2017; Pandey et al., 2018; Zha et al., 2019; Wu et al., 2021), composite scaffold (Zhou et al., 2021), etc., and there is no research on the antibacterial use of Ti_3_C_2_T_x_ alone in titanium-based coatings without adding any auxiliary materials. It has been reported that layered Ti_3_C_2_T_x_ nanosheets with high hydrophilicity can generate edge-cutting and wrapping effects after absorbing the surface of microorganisms (Rasool et al., 2016). Because of the anionic nature of its surface, Ti_3_C_2_T_x_ may also react with some molecules in the cell wall and cytoplasm of microorganisms, destroying the cell structure and leading to the death of microorganisms (Soleymaniha et al., 2019). This reveals the powerful antibacterial application potential of Ti_3_C_2_T_x_.

EPD is an important craft of colloidal depositing, which can deposit various materials from colloidal suspensions (Hu et al., 2020). In view of its high deposition rate, uniform film formation, simple equipment, high material purity, and unrestricted substrate size, EPD stands out among many titanium-coating methods and is diffusely used in the preparation of medical thin films and coatings (Sikkema et al., 2020). The process parameters in EPD (e.g. suspension concentration, applied voltage value, and deposition time) have a considerable influence on the morphology and performance of the coating (Chavez-Valdez et al., 2013). Therefore, coatings with different coverage, thickness, microstructural morphology, density, and adhesion strength can be obtained by changing the process parameters, and the mechanical properties, corrosion resistance, and bonding strength of the titanium substrate can be adjusted within a certain range. In this study, we selected the few-layer MXene (Ti_3_C_2_T_x_) colloidal dispersion with a negative charge as the raw material, and the coatings were prepared on the surface of a pure titanium sheet by anodic EPD for the first time under different deposition voltages. In the circumstance of frequent antibiotic resistance, it is significant to investigate how to apply a new nano- Ti_3_C_2_T_x_ coating without adding any electrolytic ions to the titanium inert surface to solve the clinical problem of peri-implantitis. We plan to carry out a series of surface characterizations and assessments, combining the cell activity evaluation of the mouse pre-osteoblast cell line MC3T3-E1 with the *in vitro* antibacterial property investigation of *S. aureus* and *MRSA*. This work aims to prepare an MXene coating model with superior mechanical and biological properties and provide a new coating modification method for bone defect repairing materials represented by titanium.

## 2 Materials and methods

### 2.1 Specimen preparation

One side of the pure titanium plates with the dimensions 10 × 10 × 1 mm^3^ was polished step-by-step with series abrasive papers (600–1500 mesh) to a mirror flat and then placed in acetone, absolute ethanol, and ultrapure water for ultrasonic cleaning for 20 min, which were denoted as Ti. The preparation process of Ti_3_C_2_T_x_ few-layer dispersion is summarized as follows. In the state of magnetic stirring, 1 g of LiF was added to 20 ml of 9M HCl, and then 1 g of Ti_3_AlC_2_ powders (purchased from Jilin 11 technology Co., Ltd.) were slowly poured into the mixture within 20 min. After stirring continuously for 24 h (35°C, 200 r/min), the resulting etching solution was washed with ultrapure water through centrifugation (3,500 rpm, 5 min for each circle) and mixed by hand alternately for about 8–9 times, until the pH of the supernatant was above 6, collecting the supernatant as the Ti_3_C_2_T_x_ few-layer dispersion and stored at 4°C for use. Thinking over the easy-oxidized characteristics of MXene at high temperature and high voltage, the fresh Ti_3_C_2_T_x_ few-layer dispersion (0.15 mg/ml) was sonicated at low speed for 30 min in an ice-water bath beforehand and negatively charged Ti_3_C_2_T_x_ was uniformly deposited on the surface of the titanium sheet by anodic electrophoresis at 30, 40, and 50 V for 2 min, which were characterized as M30, M40, and M50, respectively. The samples were cut with a diamond saw blade under ice-water cooling to expose the cross-sectional topography for observation.

### 2.2 Surface characterization

A field-emission scanning electron microscope (FE-SEM, Nova Nano SEM 450, United States) with an accelerating voltage of 20 KV was used to observe Ti, M30, M40, and M50. X-ray diffraction (XRD, Rigaku Smartlab, Bruker) with a wide-angle diffraction-Cu target (5–85°) radiation source was applied to investigate the phase composition of the surface of each sample. Raman spectra of various sample surfaces were obtained using a Raman microscope system (Lab RAM, HR Evolution, France) with an Ar-ion laser (514 nm) for excitation. The chemical composition of various samples was determined by X-ray photoelectron spectroscopy (XPS, Thermo Fischer, ESCALAB 250Xi, United States) using an Al ka excitation light source (hv = 1486.6 eV). The static water contact angle of each sample surface was measured using a contact angle meter (CAM, Kruss DSA100, Germany). Nanoindenter (MCT + UNIT + MST) was used to test the wear resistance of each coating sample surface below 20 GPa. Among them, 15 N is the maximum load of the nano-scratch experiment at a loading speed of 29.98 N/min, the scratch speed is 6 mm/min, and the scratch length is 3 mm.

### 2.3 *In vitro* cytocompatibility evaluation

#### 2.3.1 Cell culture

A mouse pre-osteoblast cell line (MC3T3-E1; ATCC CRL-2593, United States) was cultured at 37°C in a 5% CO_2_ incubator with the alpha-minimum essential medium (α-MEM, GIBCO, United States) supplemented with 10% fetal bovine serum (FBS, GIBCO, United States) and 1% penicillin–streptomycin (PS, Hy Clone, United States). The culture medium was changed every 2 days, and the MC3T3-E1 cells could be used for further studies when the confluency of cells reached around 80%. The cells were detached by incubating in a trypsin/EDTA (0.25% trypsin, 0.02% EDTA) (Gibco, Invitrogen) solution for 1 min at 37°C, then centrifuged for 5 min at 1,000 r/min and resuspended in the α-minimum essential medium for seeding on sample surfaces. A cell density of ∼2 × 10^4^ cells/well was used for all studies. Before reseeding, the specimens were sterilized in 75% ethanol for 2 h, dried by UV irradiation for 1 h on the super clean bench, and then incubated in the culture medium overnight.

#### 2.3.2 Cell cytotoxicity

The live-dead cell viability/cytotoxicity detection kit (TIANDZ, China) was used to test the cytotoxicity of the samples. After seeding the MC3T3-E1 cells on each sample for 24 h, all cells were stained by Calcein-AM (2 μM) and PI solutions (8μM) for 30 min at room temperature in the dark. Photographs of cells under a fluorescence microscope (Olympus, Japan) were taken within 1 h. According to the manufacturer’s instructions, live cells were stained green, while dead cells fluoresced red.

#### 2.3.3 Cell morphology

The MC3T3-E1 cells were seeded on specimen surfaces for 24 h and washed with PBS, then fixed with 4% paraformaldehyde solution for 10 min. After permeation with 0.5% Triton X-100 for 5 min and rinsed thrice with PBS, staining was conducted with the Fluorescein isothiocyanate-phalloidin (FITC-phalloidin, Solarbio Biotech Co., Ltd., China) with 1% albumin from bovine serum (BSA, Sigma, United States) for 30 min at room temperature in the dark. Finally, DAPI was added for a further 5 min. The stained actin cytoskeletons (green) and cell nucleus (blue) were visualized with a fluorescence microscope (Olympus, Japan).

#### 2.3.4 Cell adhesion

After seeding the MC3T3-E1 cells on each sample for 4 and 24 h, the cells on various sample surfaces were fixed in 2.5% glutaraldehyde for 4 h at 4°C and then rinsed thrice with PBS. The samples were dehydrated by using ethanol of graded v/v concentration (30, 50, 75, 85, 95, and 100%, 15 min each), and dried overnight under natural ventilation. Lastly, the samples were sputter-coated with gold and observed by SEM.

#### 2.3.5 Cell proliferation

The MC3T3-E1 cells were cultured on specimen surfaces for 1, 3, and 5 days, and proliferation was evaluated by using the cell counting kit-8 (CCK-8) assay (APE×BIO, United States). A highly water-soluble tetrazolium salt, WST-8, is reduced in the presence of an electron-coupling reagent to yield the water-soluble formazan form of the dye. The amount of formazan produced by dehydrogenase has a direct linear relationship with the number of viable cells. At each time point, 350 uL of working solution (WST-8: normal medium = 1:10 (v/v)) was introduced to each sample and three blank wells were added. After incubating at 37°C for 1 h and blowing out the staining solution, 100 ul of staining solution per well was transferred to a 96-well plate, and then the absorbances at 450 nm were examined.

### 2.4 *In vitro* antibacterial tests

#### 2.4.1 Culture of *S. Aureus* and MRSA

*S. aureus* (ATCC 29213) and *MRSA* (ATCC 43330) were purified by scribing on Luria-Bertani (LB) agar plates and incubated at 37°C overnight. A single colony was selected in a 5 ml LB liquid culture medium and incubated overnight at 37°C to obtain a bacterial suspension. The bacterial suspension was then gradually diluted by a 10% LB liquid culture medium to 10^7^ CFU/ml.

#### 2.4.2 Bacterial integrity test

The samples were first sterilized with 75% (v/v) ethanol for 2 h and dried by UV irradiation for 1 h on the super clean bench. Subsequently, bacterial suspensions (50 uL, 10^7^ CFU/ml) were inoculated on different sample surfaces and incubated for 24 h at 37°C.

Bacteria adhering to the sample surfaces were stained with a LIVE/DEAD Bacterial Staining Kit (BBcellProbe® N01/PI, BestBio) for 15 min. Then the samples were washed with 0.9% saline solution. The distribution of live bacteria (green) and dead bacteria (red) was observed by fluorescence microscopy.

The cell morphology of these two bacteria under SEM was used to compare the antibacterial activities of Ti, M30, M40, and M50. For SEM observation, the bacteria on the sample surfaces were fixed with 2.5% glutaraldehyde solution at 4°C overnight, dehydrated with gradient ethanol solutions (30, 50, 75, 85, 95, and 100% (v/v), 15 min each), and dried with hexamethyl disilazane ethanol solution series.

#### 2.4.3 Agar culture observation

Bacterial counting is to further assess the antibacterial activities of various samples. After culturing for 24 h at 37°C, the samples with bacterial suspensions were transferred to test tubes containing 4 ml of 0.9% NaCl solution and shaken well to separate the bacteria from the surface of the samples. Then, the bacterial suspensions were serially diluted 10-fold with 0.9% NaCl solution. Finally, 100 uL of the diluted bacterial suspensions were added to a standard LB agar culture medium and the culture was continued for 18 h at 37°C. Finally, photographs of agar culture plates were taken.

#### 2.4.4 Evaluation of bacterial viability

The AlamarBlue assay kit was used to assess bacterial viability on different samples. After culturing for 24 h at 37°C, 500 uL of 10% AlamarBlue was introduced to each sample and cultured for another 2 h at 37°C in the dark. Finally, 100 uL of the medium was transferred to a 96-well black plate and the corresponding fluorescence intensity (FI) was measured with an extinction wavelength of 560 nm and an emission wavelength of 590 nm. The antibacterial rate was calculated as follows:

$$\mathrm{Antibacterialratio}\left(\%\right)=\frac{\mathrm{FIcontrol-FItest}}{\mathrm{FIcontrol}}\mathrm{\times 100},$$

where FI_test_ represents the fluorescence intensity of M30, M40, and M50; FI_control_ was the fluorescence intensity of Ti.

#### 2.4.5 Intracellular reactive oxide assay

Intracellular reactive oxygen species (ROS) levels in bacteria were performed with an ROS detection kit. 2′,7′-Dichloro-dihydrofluorescein diacetate (DCFH-DA) can be converted to non-fluorescent dichlorofluorescein (DCFH) by deacetylation with intracellular esterases. DFCH can be oxidized by ROS in cells to produce 2′,7′-dichlorofluorescein (DCF). Therefore, the fluorescence intensity of DCF represents the ROS level to some extent. Bacteria were cultured on different samples for 24 h at 37°C, and then 500 uL DCFH-DA (10 mM) was introduced into each sample well. After culturing for 20 min at 37°C in the dark, 100 μL of the medium was transferred to a 96-well blank plate, and the fluorescence intensity corresponding to DCF was detected by using a microplate reader with an extinction wavelength of 488 nm and an emission wavelength of 535 nm. The ROS levels were expressed by calculating the ratio of (F _test_–F _blank_)/(F _control_–F _blank_), in which F _test_ was the fluorescence intensity of the coated samples; F _control_ was the fluorescence intensity of pure Ti; F _blank_ was the fluorescence intensity of the 24-well plate without bacteria and samples. At last, the fold increase was obtained by normalizing the control group of pure Ti.

### 2.5 Statistical analysis

The data were expressed as the mean ± standard deviation. One-way analysis of variance (ANOVA) and Tukey’s multiple comparison tests were used to analyze the statistical significance of the difference. Statistical analysis was assessed using GraphPad Prism 9.0 software. A value of *p* < 0.05 was considered statistically significant.

## 3 Results and discussion

### 3.1 Surface characterization

**FIGURE 1:**
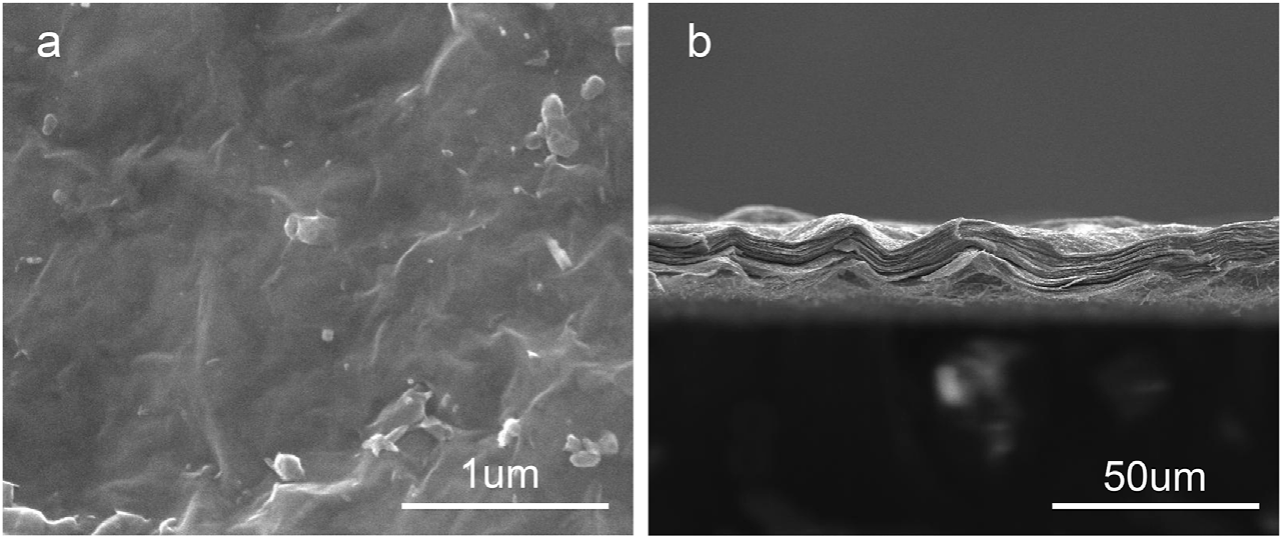
Surface **(A)** and cross-section **(B)** morphology of Ti_3_C_2_T_x_ films.

**FIGURE 2:**
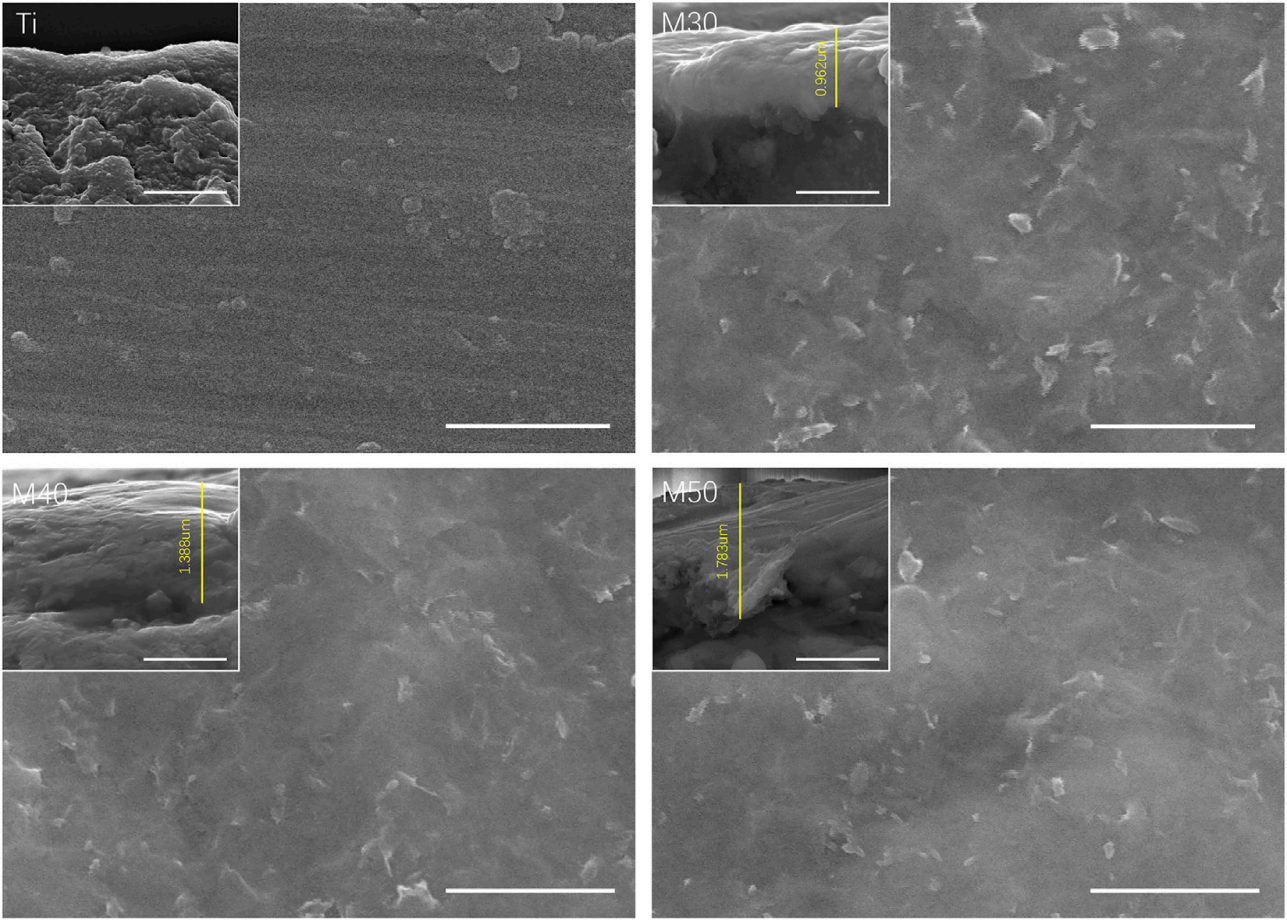
Surface and cross-section morphologies of various samples. The scale bar is 1 um.

Figures 1A,B; 2 show the surface and section morphologies of the Ti_3_C_2_T_x_ MXene film, Ti, M30, M40, and M50. From the surface of the samples, the surface of the MXene film is a wrinkled nanostructure with uniform morphology and no obvious voids and the surface of pure Ti is relatively flat after treatment, while M30, M40, and M50 display uneven surfaces such as waves, owing to the Ti_3_C_2_T_x_ deposited on the titanium surface uniformly. As for the cross-section, the cross-section of the MXene film showed a layered and tightly stacked structure similar to an “accordion” after etching from Ti_3_AlC_2_ powders, and a 2D nanosheet structure with few layers of Ti_3_C_2_T_x_ and uniform size is obtained. The cross-section morphologies of the coated samples exhibit a distinct and tightly fitted layered MXene structure as shown in Figure 2. With the increase in deposition voltages, the film thickness becomes thicker, which means an increase in the number of MXene layers.

**FIGURE 3:**
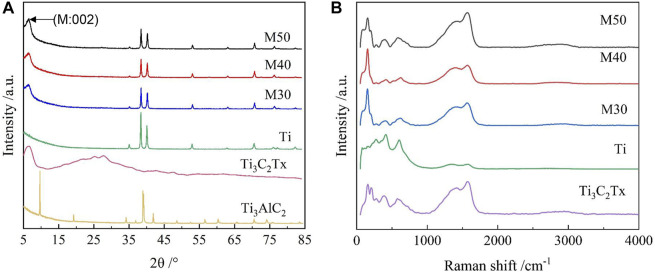
**(A)** XRD patterns of Ti_3_AlC_2_, Ti_3_C_2_T_x_, Ti, M30, M40, and M50; **(B)** Raman spectra of the samples.

The samples were further characterized by XRD and Raman spectroscopy. Regarding the phase composition of various samples, it can be seen from the XRD patterns (Figure 3A) compared with Ti_3_AlC_2_ that the position of the (002) main peak of Ti_3_C_2_T_x_ is shifted to the left by a certain angle, and the peak intensity becomes shallower and wider, indicating the decreasing of crystallization degree and the increasing of the interlayer space after etching (Allen-Perry et al., 2021). All coated samples exhibited typical α-Ti features (Jin et al., 2014), but the intensity of the main Ti phase peak was lower than that of pure titanium. Moreover, the representative (002) peak of Ti_3_C_2_T_x_ at 2θ = 7.5° remained clear and obvious in coatings. No shift to the low-angle side was observed. The resultant measurements indicated the characteristic loading of Ti_3_C_2_T_x_ on titanium sheets. Figure 3B shows the Raman spectra of various samples. The characteristic peaks of Ti_3_C_2_T_x_ at 210, 382, and 600 cm^−1^ assigned as Ti-C vibrations are highly consistent with previously reported data (Xie et al., 2019). These characteristic peaks also emerge on all coated samples, but not on pure titanium.

**FIGURE 4:**
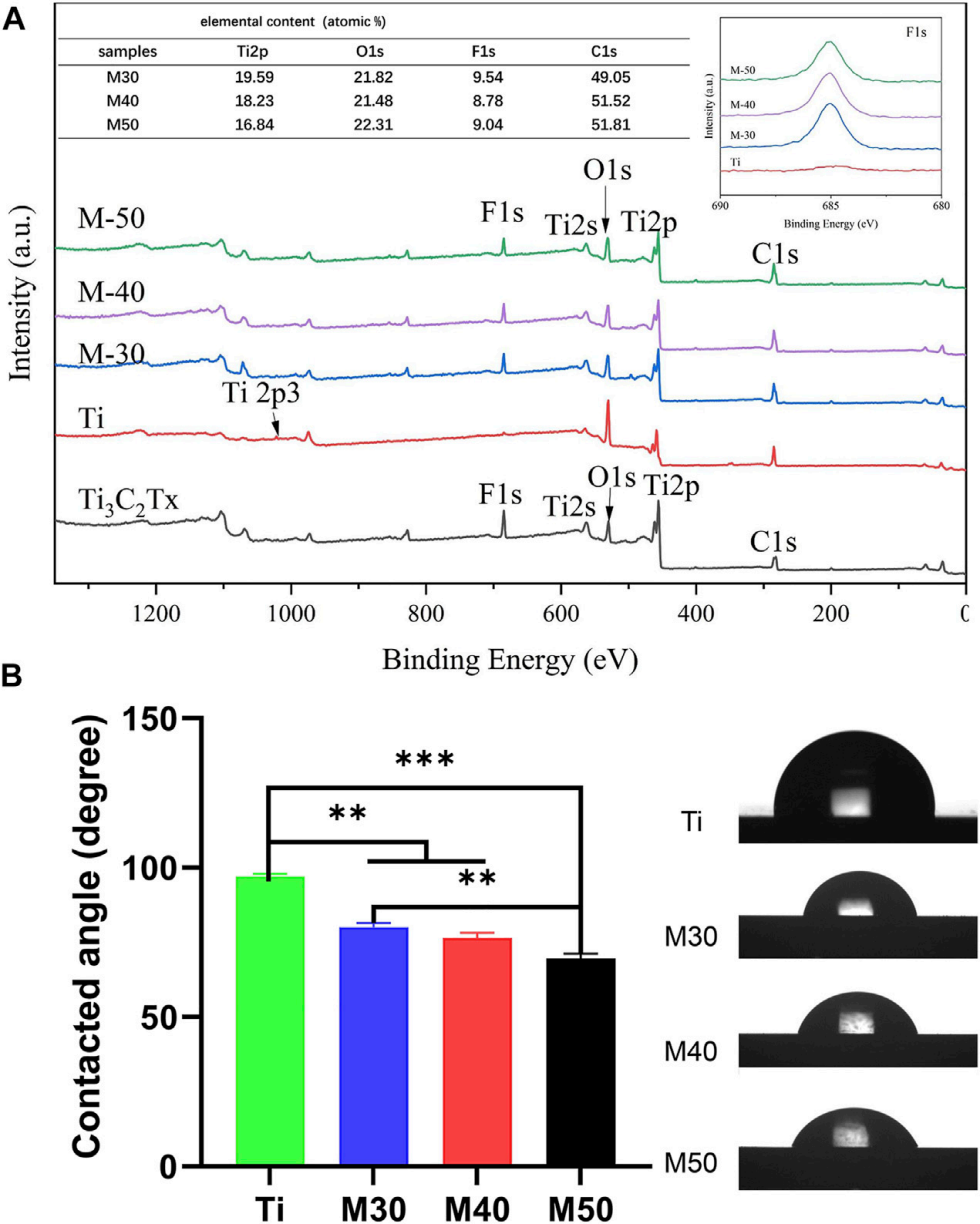
**(A)** XPS full spectra and the inset are the relative surface element content and F 1s spectra of various samples; **(B)** Water-contact angle measurements of the samples, *n* = 3, **p < 0.05, and ***p < 0.01.

The element types and chemical states of samples were investigated by the XPS technique and the results are shown in Figure 4A. The F-segment peak at 685.2 eV of Ti_3_C_2_T_x_ clearly appeared on each coated sample, but not on the pure titanium sheet. The titanium (Ti2p), carbon (C1s), and oxygen (O1s) that are corresponding to the binding energy 460.9, 285.1, and 532.4 eV, respectively, coexisted on the surface of coatings (Awasthi et al., 2020). By analyzing the elemental content table, it can be seen that with the increase in the deposition voltage, the titanium composition on the surface of the coating samples decreased step-by-step, which may be caused by the increase in the thickness of the Ti_3_C_2_T_x_ coating, which increases the coverage of the bottom pure titanium sheet. Combining the results of the XRD patterns, Raman spectra, and XPS spectra implies that Ti_3_C_2_T_x_ was successfully introduced to the titanium sheet surface by a simple electrophoretic deposition technique.

The static water contact angle was measured to evaluate whether the addition of Ti_3_C_2_T_x_ could improve the hydrophilicity of the material. As shown in Figure 4B, the pure titanium sheet exhibits mild hydrophobic behavior with a water contact angle of 97.02 ± 0.8893 , which is slightly larger than in previous studies (Yamauchi et al., 2017; Sharifi et al., 2020). This may be ascribed to the reduction of the roughness and oxygen terminal group content of the titanium sheet after grinding and polishing. With the increase in the coating thickness from the rising of the deposition voltage, the surface contact angle decreases to 80.12 ± 1.379°, 76.45 ± 1.767°, and 69.58 ± 1.616° in turn after the introduction of Ti_3_C_2_T_x_ as the surface coating. The results can be explained by changes in the physical and chemical composition of the sample surfaces, such as roughness, surface charge, and different contents of oxygen terminal groups (Lee et al., 2015; Liu et al., 2017; Zhou et al., 2017). In general, the equilibrium contact angle for hydrophilic surfaces is below 90°, while the contact angle for hydrophobic surfaces is greater than 90°. Surfaces with moderate hydrophilicity are more prone to bind cells than extremely hydrophobic or hydrophilic surfaces (Agarwalla et al., 2020). Since the hydrophilicity of biomaterials has a considerable impact on cell adhesion, proliferation, and differentiation (Bacakova et al., 2011; Qi et al., 2012), a slight decline in the water contact angle is of great importance.

**FIGURE 5:**
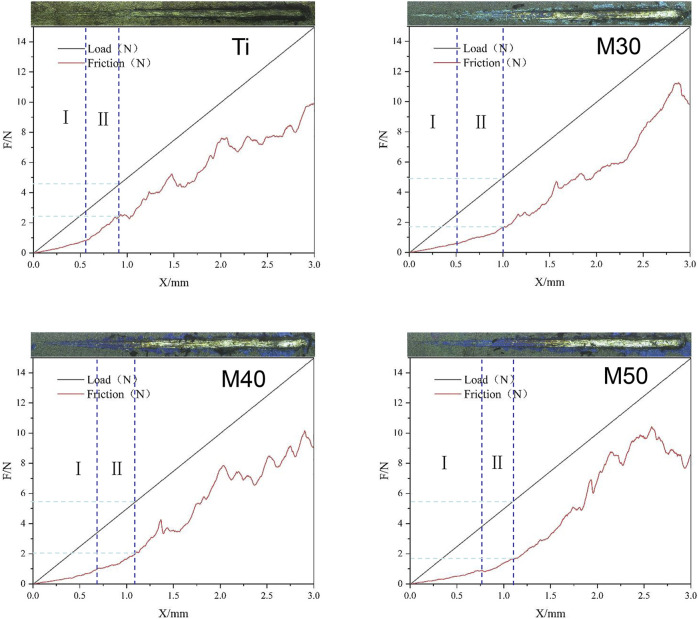
Load/fiction (N)-displacement (mm) curve and upper apex are surface topography after being scratched under load of the samples.

In the nano-scratch experiment, the elastic deformation stage (I) and plastic deformation stage (II) of the samples’ stress-induced failure have been marked in Figure 5. For stage (I), the probe indenter was in contact with the sample surface and moved forward, and then the friction force was generated. Under the action of friction and positive pressure, elastic deformation occurred. At this moment, the friction increased linearly with the increasing load; in stage (II), the pressure and friction continued to increase. When the combined force of the pressure and friction exceeded the elastic deformation limit of the coating, it reached the plasticity deformation stage. The scratches kept deepening and the friction coefficient (lateral force/normal force) also increased. When the needle tip of the indenter scratched the surface and reached the substrate, the friction curve had an inflection point; this load is the critical load for the failure of the adhesion of the film substrate (Zhang et al., 2020). The critical load is affected by the hardness and elastic modulus of the coating, the substrate, the structure, and thickness of the film, etc. (Rustamov et al., 2019). After the end of stage (II), the sample surface was ruptured. The positive pressure and scratch depth continued to increase. At this time, the coating began to peel off in large pieces. In addition, the scratch width becomes wider. The friction force and plastic deformation suddenly increase, and the friction force curve appeared as a violent fluctuation rather than a relatively smooth rising straight line like stages (I) and (II). As shown in Figure 5, the surface of the pure titanium sheet reaches the critical load when the normal pressure is 4.652 N and the friction force is 2.511 N. The test values of the coated specimens (M30/40/50) are 4.923, 5.451, and 5.458 N (the normal pressure) and 1.781, 2.087, 1.794 N (the friction force), respectively. The results show that the surface friction force and friction coefficient of the coated samples are lower than those of pure titanium sheets; the thicker the coating, the greater will be the critical load. At the same time, the longer it takes to be worn through by the same load, that is, the better the wear resistance.

### 3.2 *In vitro* cytocompatibility evaluation

#### 3.2.1 Cell cytotoxicity

**FIGURE 6:**
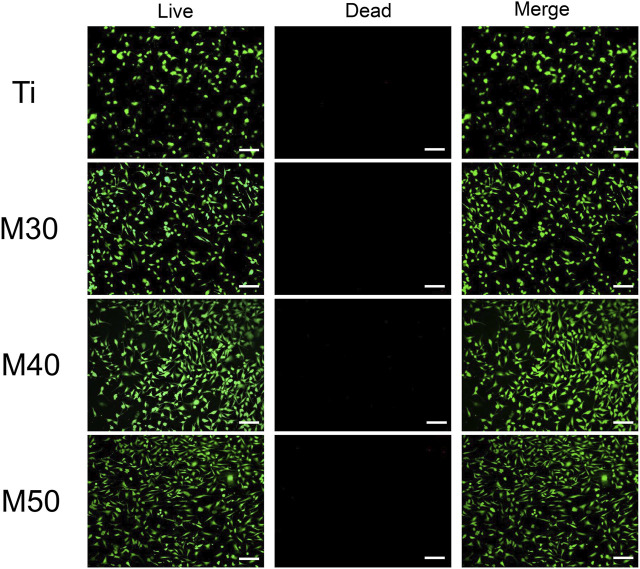
Live/dead double staining of MC3T3-E1 after seeding on various samples for 24 h. Live cells were stained fluorescent green and dead cells were stained red. The scale bar is 100 μm.

After being coated with Ti_3_C_2_T_x_, the biological properties of the samples were investigated preliminarily by using MC3T3-E1. Cells were seeded on Ti, M30, M40, and M50 for 24 h, immediately followed by live/dead double staining to evaluate the cell cytotoxicity. As shown in Figure 6, compared with the pure titanium sheet, the number of living cells on the coated samples increased to a certain extent, which may be related to different proliferation activities. There existed sporadic red fluorescence and mainly green fluorescence on each sample surface. This result indicated that the coatings are non-toxic, which may result from the low concentration and flat surface of the Ti_3_C_2_T_x_ MXene. Excessive oxidizing residue (Ti_2_O_3_) can result in rupturing of the cell membrane (Lim et al., 2021). The synthesis method of Ti_3_C_2_T_x_ in this study is mild without using HF for decreasing the degrees of Ti_2_O_3_ in the final product. Ti_3_C_2_T_x_ shows a dose- and chemical composition-dependent cytotoxicity (Lim et al., 2021). Scheibe et al. indicated that Ti_3_C_2_T_x_ MXene is more than 80% cytocompatible with non-malignant cells if the concentration is between 10 and 400 μg/ml (Scheibe et al., 2019). The fresh Ti_3_C_2_T_x_ few-layer dispersion (150 ug/mL) deposited on pure Ti is biocompatible.

#### 3.2.2 Cell adhesion and morphology

**FIGURE 7:**
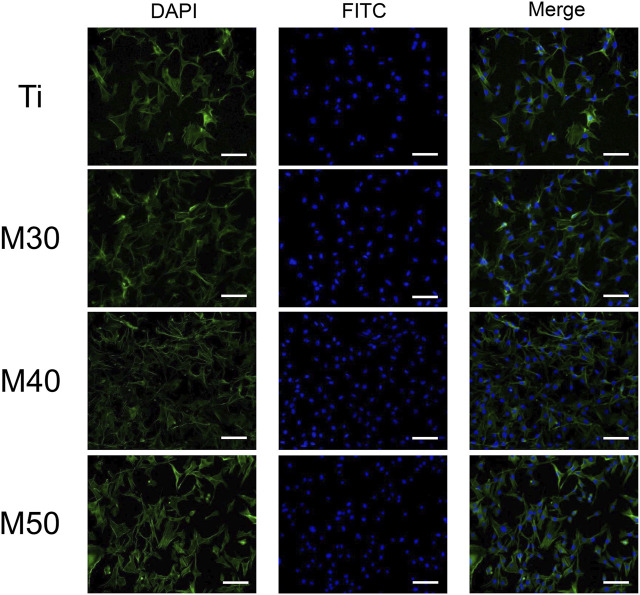
Fluorescent images of MC3T3-E1 cultured on various samples for 24 h with F-actin stained with FITC (green) and the nucleus stained with DAPI (blue). The scale bar is 100 μm.

**FIGURE 8:**
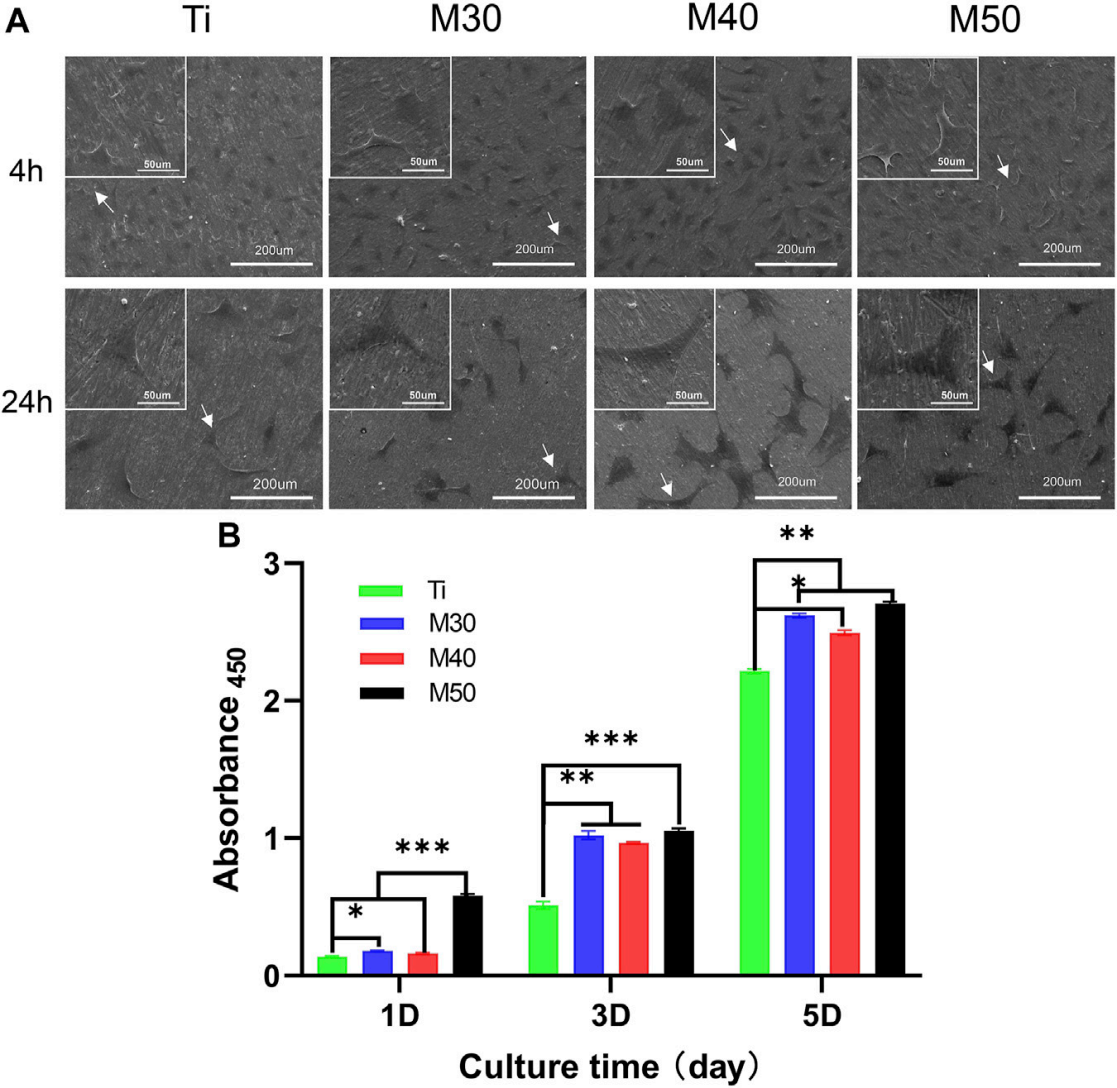
**(A)** SEM morphology of MC3T3-E1 cultured on the sample surfaces for 4 and 24 h, in which white arrows evince cellular outlines of MC3T3-E1; **(B)** absorbance 450 on each sample after 1, 3, and 5 days of seeding to show cell proliferation capacity, *n* = 5, ∗*p* < 0.1, **p < 0.05, and ∗∗∗*p* < 0.001.

To investigate the initial adhesion and morphology of MC3T3-E1, cells cultured on the sample surface for 4 and 24 h were observed under an SEM (Figure 8A). After culturing for 4 h, MC3T3-E1 cells adhered tightly to the sample surface (pointed by white arrows). The expression of filopodia and lamellipodia was more pronounced on the coated samples than in the relatively round cell morphology on pure titanium sheets. When it comes to 24 h, compared with pure titanium sheets, the cells on the coated samples’ surface had a larger spreading area and were more closely connected with each other. We also confirmed this result with fluorescence staining images (Figure 7), where most cells on pure titanium sheets exhibited spindle-shaped morphology with some filopodia. However, cells on the coated samples exhibited polygonal morphology with sufficient lamellipodia. The expressions of F-actin on the coated samples were slightly higher than that on pure Ti. With the increasing Ti_3_C_2_T_x_ layers, the expressions of F-actin showed a slightly upward trend. The aforementioned phenomenon may indicate that the charge interaction and the enhanced wettability between the surface of Ti_3_C_2_T_x_ and cells are beneficial to the adhesion and growth of cells (Bacakova et al., 2011; Guo et al., 2016). The number of adherent cells increased along with the water contact angle of the sample surface reducing, which was similar to the findings of Arima and Iwata (2007).

#### 3.2.3 Cell proliferation

The cell proliferation capacity of MC3T3-E1 on various samples was investigated by comparing the number of viable cells seeded on sample surfaces for 1, 3, and 5 days with the cck-8 assay. As shown in Figure 8B, the absorbance values of all four groups gradually increased with the extension of the culture time, indicating that the cells were in a suitable growth environment. The cell proliferation rate on the coated samples was significantly higher than that on the pure titanium sheet (*p* < 0.05). Among the individual coating samples, M50 exhibited the highest cell proliferation, followed by M30 and M40. The trend was especially vivid when the cells were cultured for 1 day (*p* < 0.001).

### 3.3 *In vitro* antibacterial tests

#### 3.3.1 Bacterial integrity testing

**FIGURE 9:**
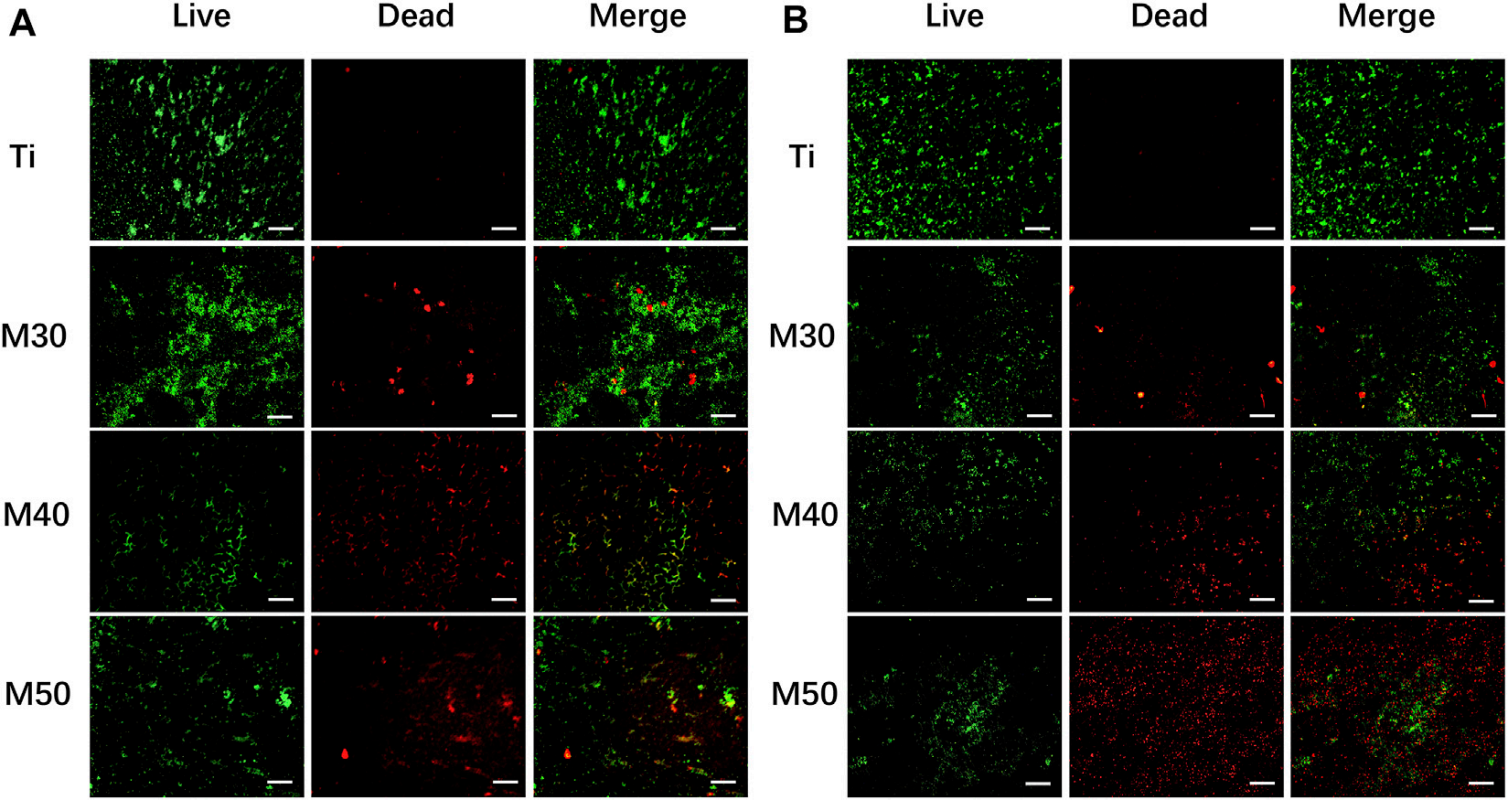
Live/dead fluorescent staining images of bacteria adhered to the Ti_3_C_2_T_x_-coated titanium sheets and titanium sheet: **(A)** *S. aureus*; **(B)** *MRSA* (live bacteria were stained fluorescent green and dead red). The scale bar is 100 μm.

The bacterial viability after coming into contact with the samples was evaluated by fluorescence microscopy observation. The bacteria were stained with an N01/PI fluorescent probe at first. The fluorescent probe N01 could pass through the cell membrane of live bacteria and exhibited bright green fluorescence after excitation. However, the PI could not enter the live bacteria, and thus, could stain the dead bacteria with a damaged cell membrane alone and exhibited red fluorescence after excitation. As shown in Figure 9, more dead bacteria were observed on the coated sheets, whereas a relatively small number of dead and mostly live bacteria were observed on the titanium sheet. This result verified the antibacterial ability of Ti_3_C_2_T_x_ coatings and the destruction of bacterial membrane integrity. *S. aureus* (Figure 9A) showed small-scale aggregation on the samples’ surface, while *MRSA* (Figure 9B) showed a more dispersed distribution.

**FIGURE 10:**
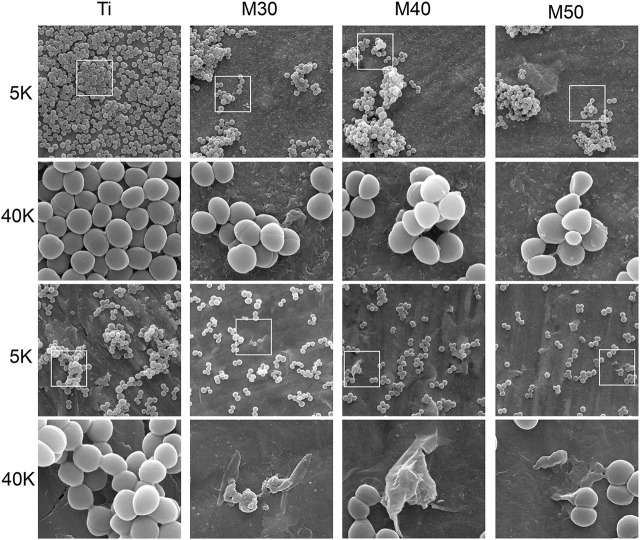
Morphological observation by SEM of both *S. aureus* (top plane) and *MRSA* (bottom plane) on various sample surfaces at different magnifications.

The antibacterial activity of Ti, M30, M40, and M50 was further assessed with morphological changes of Gram (+) *S. aureus* and *MRSA*. After culturing for 24 h at 37°C, the results are shown in Figure 10. At a low magnification of 5 K, many bacteria adhered to the pure Ti surface and aggregated together, while the amount and distribution of bacteria decreased with the increasing thickness of Ti_3_C_2_T_x_ coatings. But for *S. aureus*, there was an increase in the agglomeration stackability between bacteria on the coated samples. The antibacterial properties of Ti_3_C_2_T_x_ may be partly attributed to the negative charge and good electrical conductivity on its surface (Rasool et al., 2016), which is associated with the presence of terminal groups such as = O, –OH, and–F, and a TiO2 surface-passivation layer. A titanium sheet is a conductive metal, and Ti_3_C_2_T_x_ with a high negative surface charge is tightly coated on the surface of the titanium sheet, which synergistically enhances the conductivity of the coated sample. Simultaneously, both chemical reactions and physical repulsion may occur owing to the negatively charged bacteria surface (Simanski et al., 2013). As the deposition voltage increases, the surface of the coating material may have a higher cathodic potential, thus showing a stronger anti-adhesion ability (Canty et al., 2019). Under high magnification at 40 K, it can be clearly seen that both bacteria grow well on pure titanium sheets with typical features of smooth, spherical, and intact cell morphology. As for the cell body morphology of *S. aureus*, merely small-sized bacterial cell bodies with no obvious agglomeration were shrunken, and there were no observable bacterial cell bodies with a severely deformed cell membrane; while disorganized *MRSA* with disrupted cell bodies could all be found on the surface of M30, M40, and M50. This may be the result of the reduced edge-cutting effect caused by the state change of Ti_3_C_2_T_x_ (Zamhuri et al., 2021). Differences in surface chemistry may affect the toxicity and antibacterial activity of the Ti_3_C_2_T_x_ MXene (Lim et al., 2021). Compared to bacteria adhered to pure Ti with intact cell morphology, bacteria adhered to coatings are captured or encapsulated by nanometer-thick Ti_3_C_2_T_x_ and form aggregates. Likewise, the bacterial surface morphology gradually became shrunken and rough. Understanding the interaction between the surface of the Ti_3_C_2_T_x_-coating material and the cell envelope is of great significance for evaluating its application as a bacteriostatic agent in implantation clinics. The great electrical conductivity of Ti_3_C_2_T_x_ films has been confirmed (Ti_3_C_2_T_x_ monolayer: 4600 ± 1100 S/cm) (Ling et al., 2014; Lipatov et al., 2019; Tian et al., 2019), which is similar to or even exceeds that of reduced graphene oxide. The main component of the Gram (+) bacterial cell wall is peptidoglycan (PG), which is composed of the anionic sugar polymer wall called teichoic acid (WTA) (Xiong et al., 2022). These WTAs are critical in maintaining bacterial structure, replication, and other major cellular functions (Silhavy et al., 2010; Rohde, 2019). The anionic backbone on WTAs makes them always negatively charged (Brown et al., 2013). The bacterial cell membrane is characterized by phospholipids, comprising different ionic molecules (Halder et al., 2015). The presence of membrane potential and ionic molecules enables electrostatic interactions on the bacterial surface (Kim et al., 2019). The membrane permeability and membrane potential of bacteria have a remarkable variation when exposed to electric fields (Canty et al., 2019). The possible contact-active antibacterial process of Ti_3_C_2_T_x_ coating mainly included three steps. First, the bacterial cells contacted the surface of hydrophilic Ti_3_C_2_T_x_ under the static state (Yang et al., 2019). Subsequently, the electrostatic repulsion between Ti_3_C_2_T_x_ and WTA creates a stress pressure, which is able to rupture the cell wall when a threshold value is reached. The size of this small defect may diverge, leading to the formation of stomata that ultimately mediates the formation of Gram (+) wall defects (Rauch and Leigh, 2015; Alimohammadi et al., 2018). When the outer cell wall is damaged, the strong electrostatic interactions between the negatively charged Ti_3_C_2_T_x_ surface and positively charged phosphatidylcholine lipids lead to the damage of the integrity of the cell membrane (Ou et al., 2016). The mechanism, in this case, could be explained by the formation of conductive bridges over the insulating lipid bilayer, mediating electron transfer within bacterial cells (Vecitis et al., 2010; Li et al., 2014), possibly causing some molecules in the inner side of the cell membrane and cytoplasm to react, leading to apoptosis and necrosis of bacterial cells (Muraih et al., 2011; Bayer et al., 2013; Kim et al., 2019). After that, the dead bacterial cells could be easily washed off from the membrane surface because of the great hydrophilicity of Ti_3_C_2_T_x_. Thus, the Ti_3_C_2_T_x_ coatings exhibited prominent anti-biofouling properties.

#### 3.3.2 Agar culture

**FIGURE 11:**
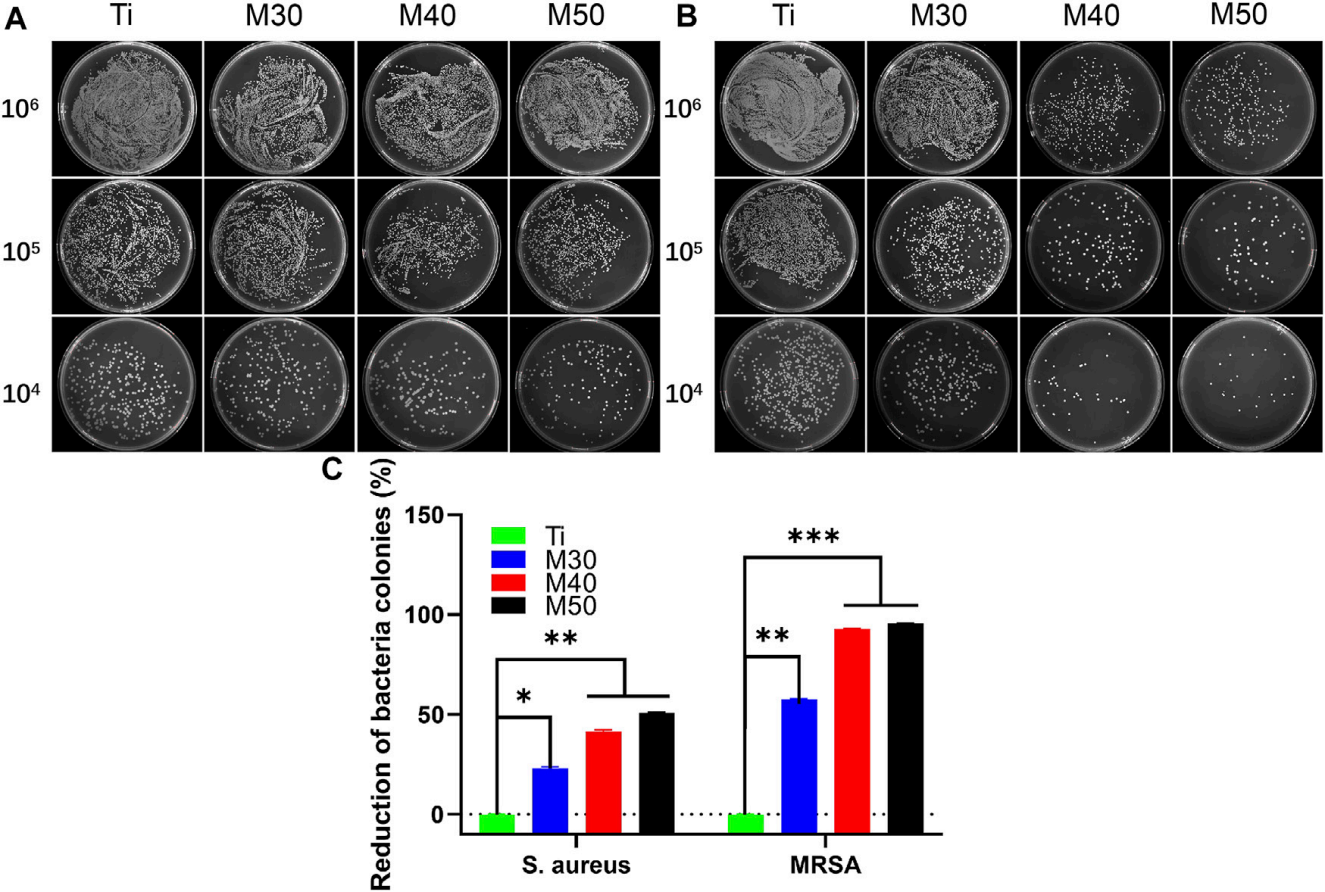
Photographs of recultivated **(A)** *S. aureus* and **(B)** *MRSA* colonies on agar culture plates; **(C)** counting analysis of reduction percentages of bacterial colonies. *p < 0.05, **p < 0.01, and ∗∗∗*p* < 0.001.

For bacteria counting, after collecting and diluting the bacterial culture solution on the sample surfaces, 100 ul of the mixed bacterial solution was cultured on the agar plate for another 18 h. As shown in Figure 11, for *MRSA* (Figure 11B), a large number of bacterial colonies can be seen on the pure titanium sheet at a density of 10^6^ CFU/ml. Compared to Ti, the number of bacterial colonies on the coated samples was significantly reduced, especially for M40 and M50. Moreover, the number of colonies gradually decreased with the increasing coating thickness. After serial dilution, the trend could be more clearly recognized as M50 had the best antibacterial activity, followed by M40. The splitting and growth of *MRSA* were significantly inhibited in the aforementioned two coating samples; nevertheless, the reduction of *S. aureus* (Figure 11A) on the CFU of 10^6^ coated samples compared to pure titanium was not as pronounced as *MRSA*. After successive ten-fold dilution, it can be relatively distinct to find out that the number of colonies of M30 is higher than that of the other two coating samples. The antibacterial activity of Ti_3_C_2_T_x_ films on Ti may be surface- and layer-dependent rather than edge-dependent. Thus, the antimicrobial effectiveness of the Ti_3_C_2_T_x_ films could increase with the increase in the number of layers. This result is similar to the antibacterial properties of flat GO sheets on a PET substrate invented by Mangadlao et al. (2015). Penicillin-binding proteins (PBPs) are peptidase enzymes located in the cell membrane necessary for cell wall biosynthesis and maintenance (Meroueh et al., 2006; Lade and Kim, 2021), which catalyzes the cross-linking of adjacent stem peptides to synthesize the PG backbone, to warrant mechanical strength and flexibility during all stages of bacterial growth (Turner et al., 2014; Egan et al., 2020). *S. aureus* has four native PBPs: PBP1, PBP2, PBP3, and PBP4 while *MRSA* contains a fifth PBP, known as PBP2a, a transpeptidase encoded by the mecA gene (Fishovitz et al., 2014; Ferrer-González et al., 2021). Inhibition of PG biosynthesis and disruption of PG integrity will result in bacterial growth ceasing (Nikolaidis et al., 2014; Naclerio and Sintim, 2020). Likewise, it is speculated that WTA can diffuse out of the PG layer with a pronounced charge dependence (Baur et al., 2014; Kurokawa et al., 2016), while WTAs play a vital role in antibiotic resistance in *MRSA* (Farha et al., 2013); they increase the vulnerability of bacteria to metals, ions, antimicrobials, and peptides (Romero-Urbina et al., 2015). Due to the electrostatic interaction between Ti_3_C_2_T_x_ and the bacterial surface, the collapse of the bacterial outer cell wall scaffold caused the exposure of the cell membrane. Oxygen-containing groups on the Ti_3_C_2_T_x_ MXene can bind with hydrogen bonds between lipopolysaccharide chains of the cell membrane to inhibit bacterial growth by preventing nutrient uptake (Rasool et al., 2016). Consequently, the phenomenon that the inhibitory activity of *MRSA* is higher than that of *S. aureus* may be correlated to the alteration in the cell wall thickness and structure caused by the different cross-linking methods of peptidoglycan on these two bacteria surfaces (Romero-Urbina et al., 2015; Rasool et al., 2017).

#### 3.3.3 Bacterial viability assessment

**FIGURE 12:**
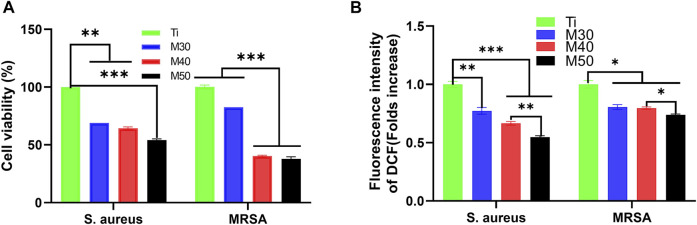
**(A)** Bacterial cell viability of *S. aureus* and *MRSA*; **(B)** intracellular ROS levels of each sample. *n* = 5, *p < 0.05, **p < 0.01, and ∗∗∗*p* < 0.001.

We used the AlamarBlue assay kit for bacterial viability detection on each sample. After incubating for 24 h at 37°C, 500 uL of 10% AlamarBlue was added and cultured for another 2 h. At last, 100 uL of the medium was transferred to a 96-well black plate, and the corresponding fluorescence intensity was detected. The results are shown in Figure 12A, for both bacteria, M50 exhibited the lowest bacterial viability (*p* < 0.001 vs. Ti). After conversion into the bacteriostatic rate, in the tested results of *S. aureus*, the antibacterial rates of the coating samples (M30/40/50) were 31.03, 35.63, and 45.98%, respectively. As for MRSA, the antibacterial rates were successively 17.39, 59.70, and 62.03%. The ability to inhibit *MRSA* of M30 is not as obvious as that against *S. aureus*, yet the antibacterial rates against *MRSA* of M40 and M50 are all over 50%, which is higher than that against *S. aureus*. Interestingly, combining the results of the previous two experiments, it can be seen that the antibacterial effect of the coated samples against *MRSA* is slightly better than that against *S. aureus* on the whole.

#### 3.3.4 Intracellular reactive oxide species assay

Previous studies have pointed to oxidative stress as the identical mechanism for the antimicrobial activity of several metals, metal-oxides, and carbon-based nanomaterials (Zhang et al., 2010; Li et al., 2012). The condition for oxidative stress of cells to occur is exposure to higher levels of reactive oxygen species (ROS) such as free radicals, O_2_^•-^, ^•^OH, and H_2_O_2_. To figure out whether ROS production led to bacteria death, we measured the ROS levels using the DCFH-DA assay. The results are displayed in the data plot (Figure 12B). Compared with pure Ti, the ROS tested by these two bacteria on the coated samples showed a decreasing trend with increasing coating thickness, and the M50 was the lowest. It indicated that the antibacterial effect of the coated samples was not directly associated with the generation of ROS. This is a peculiar phenomenon compared to previous studies (Rasool et al., 2016; Yang et al., 2021). Ti_3_C_2_T_x_ and its reaction surface have strong reducing activity which is likely to reduce the reactive oxygen species in the culture environment and bacterial cells (Mashtalir et al., 2014). From another aspect, the Ti_3_C_2_T_x_ coating on the Ti surface may form an electron-enriched physiological environment around the implant, thereby reducing the bacterial invasion of the surrounding tissue. Meanwhile, it can also reduce the damage degree to cells by oxidative stress, showing that the coating composite materials have certain antioxidant properties. In the results of *S. aureus*, the ROS level of M50 was significantly lower than that of pure titanium sheets (*p* < 0.001), and the reducing extent of the ROS level in *MRSA* between the coated samples and pure Ti was not as evident as that in *S. aureus*. Except for ROS-dependent oxidative stress, another mechanism of MXene toxicity is that strong attachment between MXene and cell membrane through the ionic interaction, hydrophobic, Van der Waals forces, and receptor–ligand binding led to membrane destabilization and loss of cell integrity. The direct contact between the MXene and cell membrane also results in MXene accumulation and ultimately cell death (Ganguly et al., 2018). That being so, the specific mediating mechanism for the antibacterial activity of Ti_3_C_2_T_x_ titanium coating remains to be further explored.

## 4 Conclusion

In this research, a series of chemical characterization results (XRD, Raman, and XPS) confirmed that few-layer Ti_3_C_2_T_x_ MXene was directly compounded on the surface of pure titanium sheets by anodic electrophoretic deposition without adding any electrolytic ions. The construction of a 2D MXene titanium coating model was successfully completed. Under SEM, the thickness of Ti_3_C_2_T_x_ coating increases with the deposition voltages. In the meantime, the static water contact angle test and nano-scratch experiment show that the coatings have excellent hydrophilicity and bonding strength, which brought up a prerequisite for the antibacterial activity of the titanium coating. Then, the results of *in vitro* cytocompatibility experiments confirmed that the coating material has no cytotoxicity, and the composite of Ti_3_C_2_T_x_ can significantly improve the initial adhesion and proliferation of MC3T3-E1 cells, and the degree of improvement is in positive proportion to the content of Ti_3_C_2_T_x_ to some extent. The surface of the Ti_3_C_2_T_x_ coating exhibits a good antibacterial effect, among which M50 is the best. They can inhibit adhesion and prevent the formation of a biofilm of *S. aureus* and its drug-resistant strain *MRSA*, which are closely related to the occurrence of peri-implantitis. This effect may result from the pressure of the cell wall through electrostatic repulsion that destroys the bacterial envelope leading to the exposure of the cell membrane. The direct contact between the MXene and cell membrane (through the ionic interaction, hydrophobic, Van der Waals forces, and receptor–ligand binding) causes MXene accumulation and damage to the integrity of the cell membrane, and ultimately leads to cell death. The specific antibacterial-mediated mechanism remains to be further explored. It can be reasonably envisaged that stronger biological activity and antibacterial property of coatings may be obtained by increasing the voltage value within an allowable scope (30–100 V). But coincidentally, we discovered the antioxidant capacity to reduce ROS in the culture environment and bacterial cells, which may reduce the degree of oxidative stress damage to cells. Through the comparative experimentations under the same condition, it was found that the antibacterial activity of the composites against *MRSA* was better than that of *S. aureus*, which may involve the difference in the cross-linking mode and thickness of peptidoglycan on the surface of these two bacteria. Therefore, under the unfavorable situation of multiple antibiotic resistance, the application of the new nano-Ti_3_C_2_T_x_ coating on titanium inert surfaces to solve the clinical problem of peri-implantitis in the biomedical field is more promising.

## Data Availability

The original contributions presented in the study are included in the article/Supplementary Material; further inquiries can be directed to the corresponding author.

## References

[B1] Agarwalla S. V. Solomon A. P. Neelakantan P. Rosa V. (2020). Novel materials and therapeutic strategies against the infection of implants. emergent Mat. 3 (4), 545–557. 10.1007/s42247-020-00117-x

[B2] Alimohammadi F. Sharifian Gh M. Attanayake N. H. Thenuwara A. C. Gogotsi Y. Anasori B. (2018). Antimicrobial properties of 2D MnO(2) and MoS(2) nanomaterials vertically aligned on graphene materials and Ti(3)C(2) MXene. Langmuir 34 (24), 7192–7200. 10.1021/acs.langmuir.8b00262 29782792

[B3] Allen-Perry K. Straka W. Keith D. Han S. Reynolds L. Gautam B. (2021). Tuning the magnetic properties of two-dimensional MXenes by. Chem. Etch. 14 (3), 694. 10.3390/ma14030694 PMC7867348 33540805

[B4] An Y. H. Stuart G. W. McDowell S. J. McDaniel S. E. Kang Q. Friedman R. J. (1996). Prevention of bacterial adherence to implant surfaces with a crosslinked albumin coating *in vitro* . J. Orthop. Res. 14 (5), 846–849. 10.1002/jor.1100140526 8893783

[B5] Arima Y. Iwata H. (2007). Effect of wettability and surface functional groups on protein adsorption and cell adhesion using well-defined mixed self-assembled monolayers. Biomaterials 28 (20), 3074–3082. 10.1016/j.biomaterials.2007.03.013 17428532

[B6] Awasthi G. P. Maharjan B. Shrestha S. Bhattarai D. P. Yoon D. Park C. H. (2020). Synthesis, characterizations, and biocompatibility evaluation of polycaprolactone–MXene electrospun fibers. Colloids Surfaces A Physicochem. Eng. Aspects 586, 124282. 10.1016/j.colsurfa.2019.124282

[B7] Bacakova L. Filova E. Parizek M. Ruml T. Svorcik V. (2011). Modulation of cell adhesion, proliferation and differentiation on materials designed for body implants. Biotechnol. Adv. 29 (6), 739–767. 10.1016/j.biotechadv.2011.06.004 21821113

[B8] Baur S. Rautenberg M. Faulstich M. Grau T. Severin Y. Unger C. (2014). A nasal epithelial receptor for *Staphylococcus aureus* WTA governs adhesion to epithelial cells and modulates nasal colonization. PLoS Pathog. 10 (5), e1004089. 10.1371/journal.ppat.1004089 24788600 PMC4006915

[B9] Bayer A. S. Schneider T. Sahl H.-G. (2013). Mechanisms of daptomycin resistance in *Staphylococcus aureus*: Role of the cell membrane and cell wall, Ann. N. Y. Acad. Sci. 1277 (1), 139–158. 10.1111/j.1749-6632.2012.06819.x 23215859 PMC3556211

[B10] Bennett-Jackson A. L. Falmbigl M. Hantanasirisakul K. Gu Z. Imbrenda D. Plokhikh A. V. (2019). van der Waals epitaxy of highly (111)-oriented BaTiO3 on MXene. Nanoscale 11 (2), 622–630. 10.1039/C8NR07140C 30560967

[B11] Bhardwaj S. K. Singh H. Khatri M. Kim K. H. Bhardwaj N. (2022). Advances in MXenes-based optical biosensors: A review. Biosens. Bioelectron. X. 202, 113995. 10.1016/j.bios.2022.113995 35065477

[B12] Brown S. Santa Maria J. P. Jr. Walker S. (2013). Wall teichoic acids of gram-positive bacteria. Annu. Rev. Microbiol. 67, 313–336. 10.1146/annurev-micro-092412-155620 24024634 PMC3883102

[B13] Cacaci M. Martini C. Guarino C. Torelli R. Bugli F. Sanguinetti M. (2020). Graphene oxide coatings as tools to prevent microbial biofilm formation on medical device. Adv. Exp. Med. Biol. 1282, 21–35. 10.1007/5584_2019_434 31468360

[B14] Canty M. K. Hansen L. A. Tobias M. Spencer S. Henry T. Luke-Marshall N. R. (2019). Antibiotics enhance prevention and eradication efficacy of cathodic-voltage-controlled electrical stimulation against titanium-associated methicillin-resistant *Staphylococcus aureus* and *Pseudomonas aeruginosa* biofilms. mSphere 4 (3), e00178–19. 10.1128/mSphere.00178-19 31043516 PMC6495338

[B15] Chavez-Valdez A. Shaffer M. S. P. Boccaccini A. R. (2013). Applications of graphene electrophoretic deposition. A review. J. Phys. Chem. B 117 (6), 1502–1515. 10.1021/jp3064917 23088165

[B16] Egan A. J. F. Errington J. Vollmer W. (2020). Regulation of peptidoglycan synthesis and remodelling. Nat. Rev. Microbiol. 18 (8), 446–460. 10.1038/s41579-020-0366-3 32424210

[B17] Farha M. A. Leung A. Sewell E. W. D'Elia M. A. Allison S. E. Ejim L. (2013). Inhibition of WTA synthesis blocks the cooperative action of PBPs and sensitizes MRSA to β-lactams. ACS Chem. Biol. 8 (1), 226–233. 10.1021/cb300413m 23062620 PMC3552485

[B18] Ferrer-González E. Huh H. Al-Tameemi H. M. Boyd J. M. Lee S. H. Pilch D. S. (2021). Impact of FtsZ inhibition on the localization of the penicillin binding proteins in methicillin-resistant *Staphylococcus aureus* . J. Bacteriol. 203 (16), e0020421. 10.1128/jb.00204-21 34031040 PMC8297533

[B19] Fishovitz J. Hermoso J. A. Chang M. Mobashery S. (2014). Penicillin-binding protein 2a of methicillin-resistant *Staphylococcus aureus* . IUBMB life 66 (8), 572–577. 10.1002/iub.1289 25044998 PMC4236225

[B20] Ganguly P. Breen A. Pillai S. C. (2018). Toxicity of nanomaterials: Exposure, pathways, assessment, and recent advances. ACS Biomater. Sci. Eng. 4 (7), 2237–2275. 10.1021/acsbiomaterials.8b00068 33435097

[B21] Guo R. Xiao M. Zhao W. Zhou S. Hu Y. Liao M. (2022). 2D Ti(3)C(2)T(x)MXene couples electrical stimulation to promote proliferation and neural differentiation of neural stem cells. Acta biomater. 139, 105–117. 10.1016/j.actbio.2020.12.035 33348061

[B22] Guo S. S. Zhu X. Y. Li M. Shi L. Y. Ong J. L. T. Janczewski D. (2016). Parallel control over surface charge and wettability using polyelectrolyte architecture: Effect on protein adsorption and cell adhesion. ACS Appl. Mat. Interfaces 8 (44), 30552–30563. 10.1021/acsami.6b09481 27762557

[B23] Halder S. Yadav K. K. Sarkar R. Mukherjee S. Saha P. Haldar S. (2015). Alteration of zeta potential and membrane permeability in bacteria: A study with cationic agents. SpringerPlus 4, 672. 10.1186/s40064-015-1476-7 26558175 PMC4633473

[B24] Hildebrandt P. (2002). Glycosaminoglycans--all round talents in coating technology, Biomedizinische Technik. Biomed. Eng. 47 (1), 476–478. 10.1515/bmte.2002.47.s1a.476 12451898

[B25] Hu S. Li W. Finklea H. Liu X. (2020). A review of electrophoretic deposition of metal oxides and its application in solid oxide fuel cells. Adv. Colloid Interface Sci. 276, 102102. 10.1016/j.cis.2020.102102 31935554

[B26] Huang K. Li Z. Lin J. Han G. Huang P. (2018). Two-dimensional transition metal carbides and nitrides (MXenes) for biomedical applications. Chem. Soc. Rev. 47 (14), 5109–5124. 10.1039/c7cs00838d 29667670

[B27] Hwang Y. E. Im S. Kim H. Sohn J. H. Cho B. K. Cho J. H. (2021). Adhesive antimicrobial peptides containing 3, 4-dihydroxy-L-phenylalanine residues for direct one-step surface coating. Int. J. Mol. Sci. 22 (21), 11915. 10.3390/ijms222111915 34769345 PMC8584447

[B28] Jang J. H. Lee E. J. (2021). Influence of MXene particles with a stacked-lamellar structure on osteogenic differentiation of human mesenchymal stem cells. Mater. (Basel) 14 (16), 4453. 10.3390/ma14164453 PMC8401813 34442976

[B29] Jastrzebska A. Karwowska E. Basiak D. Zawada A. Ziemkowska W. Wojciechowski T. (2017). Biological activity and bio-sorption properties of the Ti2C studied by means of zeta potential and SEM. Int. J. Electrochem. Sci. 12 (3), 2159–2172. 10.20964/2017.03.06

[B30] Jin G. Cao H. Qiao Y. Meng F. Zhu H. Liu X. (2014). Osteogenic activity and antibacterial effect of zinc ion implanted titanium. Colloids Surfaces B Biointerfaces 117, 158–165. 10.1016/j.colsurfb.2014.02.025 24632388

[B31] Kang M.-H. Lee D. Sung J. Kim J. Kim B. H. Park J. (2019). “2.04 - structure and chemistry of 2D materials,” in Comprehensive nanoscience and nanotechnology. Editors Andrews D. L. Lipson R. H. Nann T.. Second Edition (Oxford: Academic Press), 55–90.

[B32] Kim W. Zou G. Hari T. P. A. Wilt I. K. Zhu W. Galle N. (2019). A selective membrane-targeting repurposed antibiotic with activity against persistent methicillin-resistant *Staphylococcus aureus* . Proc. Natl. Acad. Sci. U. S. A. 116 (33), 16529–16534. 10.1073/pnas.1904700116 31358625 PMC6697817

[B33] Konstantinidis I. K. Kotsakis G. A. Gerdes S. Walter M. H. (2015). Cross-sectional study on the prevalence and risk indicators of peri-implant diseases. Eur. J. Oral Implantol. 8 (1), 75–88. 25738181

[B34] Kotsakis G. A. Olmedo D. G. (2021). Peri-implantitis is not periodontitis: Scientific discoveries shed light on microbiome-biomaterial interactions that may determine disease phenotype. Periodontol. 2000 86 (1), 231–240. 10.1111/prd.12372 33690947

[B35] Kurokawa K. Takahashi K. Lee B. L. (2016). The staphylococcal surface-glycopolymer wall teichoic acid (WTA) is crucial for complement activation and immunological defense against *Staphylococcus aureus* infection. Immunobiology 221 (10), 1091–1101. 10.1016/j.imbio.2016.06.003 27424796

[B36] Lade H. Kim J. S. (2021). Bacterial targets of antibiotics in methicillin-resistant *Staphylococcus aureus* . Antibiot. (Basel, Switz. 10 (4), 398. 10.3390/antibiotics10040398 PMC8067735 33917043

[B37] Lee J. H. Shin Y. C. Lee S. M. Jin O. S. Kang S. H. Hong S. W. (2015). Enhanced osteogenesis by reduced graphene oxide/hydroxyapatite nanocomposites. Sci. Rep. 5, 18833. 10.1038/srep18833 26685901 PMC4685392

[B38] Li J. Wang G. Zhu H. Zhang M. Zheng X. Di Z. (2014). Antibacterial activity of large-area monolayer graphene film manipulated by charge transfer. Sci. Rep. 4, 4359. 10.1038/srep04359 24619247 PMC3950808

[B39] Li Y. Han M. Cai Y. Jiang B. Zhang Y. Yuan B. (2022). Muscle-inspired MXene/PVA hydrogel with high toughness and photothermal therapy for promoting bacteria-infected wound healing. Biomater. Sci. 10 (4), 1068–1082. 10.1039/d1bm01604k 35037673

[B40] Li Y. Zhang W. Niu J. Chen Y. (2012). Mechanism of photogenerated reactive oxygen species and correlation with the antibacterial properties of engineered metal-oxide nanoparticles. ACS Nano 6 (6), 5164–5173. 10.1021/nn300934k 22587225

[B41] Lim G. P. Soon C. F. Ma N. L. Morsin M. Nayan N. Ahmad M. K. (2021). Cytotoxicity of MXene-based nanomaterials for biomedical applications: A mini review. Environ. Res. 201, 111592. 10.1016/j.envres.2021.111592 34175291

[B42] Ling Z. Ren C. E. Zhao M. Q. Yang J. Giammarco J. M. Qiu J. (2014). Flexible and conductive MXene films and nanocomposites with high capacitance. Proc. Natl. Acad. Sci. U. S. A. 111 (47), 16676–16681. 10.1073/pnas.1414215111 25389310 PMC4250111

[B43] Lipatov A. Sinitskii A. (2019). “Electronic and mechanical properties of MXenes derived from single-flake measurements,” in 2D metal carbides and nitrides (MXenes): Structure, properties and applications. Editors Anasori B. Gogotsi Y. (Cham: Springer International Publishing), 301–325.

[B44] Liu J. Zhang H.-B. Sun R. Liu Y. Liu Z. Zhou A. (2017). Hydrophobic, flexible, and lightweight MXene foams for high-performance electromagnetic-interference shielding. Adv. Mat. 29 (38), 1702367. 10.1002/adma.201702367 28799671

[B45] Mangadlao J. D. Santos C. M. Felipe M. J. L. de Leon A. C. C. Rodrigues D. F. Advincula R. C. (2015). On the antibacterial mechanism of graphene oxide (GO) Langmuir-Blodgett films. Chem. Commun. 51 (14), 2886–2889. 10.1039/c4cc07836e 25582092

[B46] Mashtalir O. Cook K. M. Mochalin V. N. Crowe M. Barsoum M. W. Gogotsi Y. J. J. o. M. C. A. (2014). Dye adsorption and decomposition on two-dimensional titanium carbide in aqueous media. J. Mat. Chem. A 2 (35), 14334–14338. 10.1039/c4ta02638a

[B47] Meroueh S. O. Bencze K. Z. Hesek D. Lee M. Fisher J. F. Stemmler T. L. (2006). Three-dimensional structure of the bacterial cell wall peptidoglycan. Proc. Natl. Acad. Sci. U. S. A. 103 (12), 4404–4409. 10.1073/pnas.0510182103 16537437 PMC1450184

[B48] Morra M. Cassineli C. (1999). Non-fouling properties of polysaccharide-coated surfaces. J. Biomaterials Sci. Polym. Ed. 10 (10), 1107–1124. 10.1163/156856299x00711 10591135

[B49] Muraih J. K. Pearson A. Silverman J. Palmer M. (2011). Oligomerization of daptomycin on membranes. Biochimica Biophysica Acta (BBA) - Biomembr. 1808 (4), 1154–1160. 10.1016/j.bbamem.2011.01.001 21223947

[B50] Naclerio G. A. Sintim H. O. (2020). Multiple ways to kill bacteria via inhibiting novel cell wall or membrane targets. Future Med. Chem. 12 (13), 1253–1279. 10.4155/fmc-2020-0046 32538147

[B51] Naguib M. Kurtoglu M. Presser V. Lu J. Niu J. Heon M. (2011). Two-dimensional nanocrystals produced by exfoliation of Ti3AlC2. Adv. Mat. 23 (37), 4248–4253. 10.1002/adma.201102306 21861270

[B52] Nikolaidis I. Favini-Stabile S. Dessen A. (2014). Resistance to antibiotics targeted to the bacterial cell wall. Protein Sci. 23 (3), 243–259. 10.1002/pro.2414 24375653 PMC3945833

[B53] Ou L. Song B. Liang H. Liu J. Feng X. Deng B. (2016). Toxicity of graphene-family nanoparticles: A general review of the origins and mechanisms. Part. Fibre Toxicol. 13 (1), 57. 10.1186/s12989-016-0168-y 27799056 PMC5088662

[B54] Pandey R. P. Rasool K. Madhavan V. E. Aissa B. Gogotsi Y. Mahmoud K. A. (2018). Ultrahigh-flux and fouling-resistant membranes based on layered silver/MXene (Ti3C2Tx) nanosheets. J. Mat. Chem. A Mat. 6 (8), 3522–3533. 10.1039/c7ta10888e

[B55] Qi R. Cao X. Shen M. Guo R. Yu J. Shi X. (2012). Biocompatibility of electrospun halloysite nanotube-doped poly(lactic-co-glycolic acid) composite nanofibers. J. Biomaterials Sci. Polym. Ed. 23 (1-4), 299–313. 10.1163/092050610X550340 21244744

[B56] Qiu J. Geng H. Wang D. Qian S. Zhu H. Qiao Y. (2017). Layer-number dependent antibacterial and osteogenic behaviors of graphene oxide electrophoretic deposited on titanium. ACS Appl. Mat. Interfaces 9 (14), 12253–12263. 10.1021/acsami.7b00314 28345852

[B57] Qiu J. Liu L. Zhu H. Liu X. (2018). Combination types between graphene oxide and substrate affect the antibacterial activity. Bioact. Mater. 3 (3), 341–346. 10.1016/j.bioactmat.2018.05.001 29988418 PMC6026326

[B58] Rasool K. Helal M. Ali A. Ren C. E. Gogotsi Y. Mahmoud K. A. (2016). Antibacterial Activity of Ti_3_C_2_T_<i>x</i>_ MXene. ACS Nano 10 (3), 3674–3684. 10.1021/acsnano.6b00181 26909865

[B59] Rasool K. Mahmoud K. A. Johnson D. J. Helal M. Berdiyorov G. R. Gogotsi Y. (2017). Efficient antibacterial membrane based on two-dimensional Ti3C2Tx (MXene) nanosheets. Sci. Rep. 7 (1), 1598. 10.1038/s41598-017-01714-3 28487521 PMC5431673

[B60] Rauch C. Leigh J. (2015). Theoretical evaluation of wall teichoic acids in the cavitation-mediated pores formation in Gram-positive bacteria subjected to an electric field. Biochimica Biophysica Acta - General Subj. 1850 (4), 595–601. 10.1016/j.bbagen.2014.12.004 25497464

[B61] Rohde M. (2019). The gram-positive bacterial cell wall. Microbiol. Spectr. 7 (3). 10.1128/microbiolspec.GPP3-0044-2018 PMC11086966 31124431

[B62] Romero-Urbina D. G. Lara H. H. Velázquez-Salazar J. J. Arellano-Jiménez M. J. Larios E. Srinivasan A. (2015). Ultrastructural changes in methicillin-resistant *Staphylococcus aureus* induced by positively charged silver nanoparticles. Beilstein J. Nanotechnol. 6, 2396–2405. 10.3762/bjnano.6.246 26734530 PMC4685924

[B63] Rustamov I. Zhang G. Skotnikova M. Wang Y. Wang Z. (2019). Fretting wear behavior and damage mechanisms of inconel X-750 alloy in dry contact condition. J. Tribol. 141 (4), 0416031–0416038. 10.1115/1.4042038 30837780

[B64] Safaee S. Valanezhad A. Nesabi M. Jafarnia S. Sano H. Shahabi S. (2021). Fabrication of bioactive glass coating on pure titanium by sol-dip method: Dental applications. Dent. Mat. J. 40 (4), 323–956. 10.4012/dmj.2020-323 33716277

[B65] Safioti L. M. Kotsakis G. A. Pozhitkov A. E. Chung W. O. Daubert D. M. (2017). Increased levels of dissolved titanium are associated with peri-implantitis - a cross-sectional study. J. periodontology 88 (5), 436–442. 10.1902/jop.2016.160524 27858551

[B66] Sang S. Guo G. Yu J. Zhang X. (2021). Antibacterial application of gentamicin–silk protein coating with smart release function on titanium, polyethylene, and Al2O3 materials. Mater. Sci. Eng. C 124, 112069. 10.1016/j.msec.2021.112069 33947562

[B67] Scheibe B. Wychowaniec J. K. Scheibe M. Peplińska B. Jarek M. Nowaczyk G. (2019). Cytotoxicity assessment of Ti–Al–C based MAX phases and Ti_3_C_2_T_<i>x</i>_ MXenes on human fibroblasts and cervical cancer cells. ACS Biomater. Sci. Eng. 5 (12), 6557–6569. 10.1021/acsbiomaterials.9b01476 33417807

[B68] Sharifi S. Islam M. M. Sharifi H. Islam R. Nilsson P. H. Dohlman C. H. (2020). Sputter deposition of titanium on poly(methyl methacrylate) enhances corneal biocompatibility. Transl. Vis. Sci. Technol. 9 (13), 41. 10.1167/tvst.9.13.41 PMC7774111 33442495

[B69] Sikkema R. Baker K. Zhitomirsky I. (2020). Electrophoretic deposition of polymers and proteins for biomedical applications. Adv. Colloid Interface Sci. 284, 102272. 10.1016/j.cis.2020.102272 32987293

[B70] Silhavy T. J. Kahne D. Walker S. (2010). The bacterial cell envelope. Cold Spring Harb. Perspect. Biol. 2 (5), a000414. 10.1101/cshperspect.a000414 20452953 PMC2857177

[B71] Simanski M. Gläser R. Köten B. Meyer-Hoffert U. Wanner S. Weidenmaier C. (2013). *Staphylococcus aureus* subverts cutaneous defense by D-alanylation of teichoic acids. Exp. Dermatol. 22 (4), 294–296. 10.1111/exd.12114 23528217

[B72] Soleymaniha M. Shahbazi M.-A. Rafieerad A. R. Maleki A. Amiri A., Promoting role of MXene nanosheets in biomedical sciences: Therapeutic and biosensing innovations: Adv. Healthc. Mat., 8(1) (2019) 1801137. 10.1002/adhm.201801137 30362268

[B73] Song F. Koo H. Ren D. (2015). Effects of material properties on bacterial adhesion and biofilm formation. J. Dent. Res. 94 (8), 1027–1034. 10.1177/0022034515587690 26001706

[B74] Thurnheer T. Belibasakis G. N. (2016). Incorporation of staphylococci into titanium-grown biofilms: An *in vitro* "submucosal" biofilm model for peri-implantitis. Clin. Oral Implants Res. 27 (7), 890–895. 10.1111/clr.12715 26461083 PMC5057304

[B75] Tian W. VahidMohammadi A. Reid M. S. Wang Z. Ouyang L. Erlandsson J. (2019). Multifunctional nanocomposites with high strength and capacitance using 2D MXene and 1D nanocellulose. Adv. Mat. 31 (41), e1902977. 10.1002/adma.201902977 31408235

[B76] Turner R. D. Vollmer W. Foster S. J. (2014) Different walls for rods and balls: The diversity of peptidoglycan, Mol. Microbiol. 91 (5), 862–874. 10.1111/mmi.12513 24405365 PMC4015370

[B77] Vacheethasanee K. Marchant R. E. (2000). Surfactant polymers designed to suppress bacterial (Staphylococcus epidermidis) adhesion on biomaterials. J. Biomed. Mat. Res. 50 (3), 302–312. 10.1002/(sici)1097-4636(20000605)50:3<302:aid-jbm3>3.0.co;2-1 10737871

[B78] Vecitis C. D. Zodrow K. R. Kang S. Elimelech M. (2010). Electronic-structure-dependent bacterial cytotoxicity of single-walled carbon nanotubes. ACS Nano 4 (9), 5471–5479. 10.1021/nn101558x 20812689

[B79] Wu Y. Zheng W. Xiao Y. Du B. Zhang X. Wen M. (2021). Multifunctional, robust, and porous PHBV-GO/MXene composite membranes with good hydrophilicity, antibacterial activity, and platelet adsorption performance. Polymers 13 (21), 3748. 10.3390/polym13213748 34771308 PMC8588032

[B80] Wu Z. Chan B. Low J. Chu J. J. H. Hey H. W. D. Tay A. (2022). Microbial resistance to nanotechnologies: An important but understudied consideration using antimicrobial nanotechnologies in orthopaedic implants. Bioact. Mater. 16, 249–270. 10.1016/j.bioactmat.2022.02.014 35415290 PMC8965851

[B81] Xie X. Zhu Y. Li F. Zhou X. Xue T. (2019). Preparation and characterization of Ti3C2Tx with SERS properties. Sci. China Technol. Sci. 62 (7), 1202–1209. 10.1007/s11431-018-9359-4

[B82] Xiong M. Chen L. Zhao J. Xiao X. Zhou J. Fang F. (2022). Genomic analysis of the unusual *Staphylococcus aureus* ST630 isolates harboring WTA glycosyltransferase genes tarM and tagN. Microbiol. Spectr. 10 (1), e0150121. 10.1128/spectrum.01501-21 35170993 PMC8849055

[B83] Yamauchi R. Itabashi T. Wada K. Tanaka T. Kumagai G. Ishibashi Y. (2017). Photofunctionalised Ti6Al4V implants enhance early phase osseointegration. Bone & Jt. Res. 6 (5), 331–336. 10.1302/2046-3758.65.Bjr-2016-0221.R1 PMC5457646 28522447

[B84] Yang J. Bao W. Z. Jaumaux P. Zhang S. T. Wang C. Y. Wang G. X. (2019). MXene-based composites: Synthesis and applications in rechargeable batteries and supercapacitors. Adv. Mat. Interfaces 6 (8), 1802004. 10.1002/admi.201802004

[B85] Yang Z. Fu X. Ma D. Wang Y. Peng L. Shi J. (2021). Growth factor-decorated Ti(3) C(2) MXene/MoS(2) 2D bio-heterojunctions with quad-channel photonic disinfection for effective regeneration of bacteria-invaded cutaneous tissue. Small (Weinheim der Bergstrasse, Ger. 17 (50), e2103993. 10.1002/smll.202103993 34713567

[B86] Yin J. Han Q. Zhang J. Liu Y. Gan X. Xie K. (2020). MXene-based hydrogels endow polyetheretherketone with effective osteogenicity and combined treatment of osteosarcoma and bacterial infection. ACS Appl. Mat. Interfaces 12 (41), 45891–45903. 10.1021/acsami.0c14752 33012164

[B87] Zamhuri A. Lim G. P. Ma N. L. Tee K. S. Soon C. F. (2021). MXene in the lens of biomedical engineering: Synthesis, applications and future outlook. Biomed. Eng. OnLine 20 (1), 33. 10.1186/s12938-021-00873-9 33794899 PMC8017618

[B88] Zha X. J. Zhao X. Pu J. H. Tang L. S. Ke K. Bao R. Y. (2019). Flexible anti-biofouling MXene/cellulose fibrous membrane for sustainable solar-driven water purification. ACS Appl. Mat. Interfaces 11 (40), 36589–36597. 10.1021/acsami.9b10606 31513743

[B89] Zhang L. Yan J. Yin Z. Tang C. Guo Y. Li D. (2014). Electrospun vancomycin-loaded coating on titanium implants for the prevention of implant-associated infections. Int. J. Nanomedicine 9, 3027–3036. 10.2147/ijn.S63991 25028544 PMC4077604

[B90] Zhang M. Xie T. Qian X. Zhu Y. Liu X. (2020). Mechanical properties and biocompatibility of Ti-doped diamond-like carbon films. ACS omega 5 (36), 22772–22777. 10.1021/acsomega.0c01715 32954124 PMC7495474

[B91] Zhang Y. Ali S. F. Dervishi E. Xu Y. Li Z. Casciano D. (2010). Cytotoxicity effects of graphene and single-wall carbon nanotubes in neural phaeochromocytoma-derived PC12 cells. ACS Nano 4 (6), 3181–3186. 10.1021/nn1007176 20481456

[B92] Zhou L. Zheng H. Liu Z. Wang S. Liu Z. Chen F. (2021). Conductive antibacterial hemostatic multifunctional scaffolds based on Ti_3_C_2_T_<i>x</i>_ MXene nanosheets for promoting multidrug-resistant bacteria-infected wound healing. ACS Nano 15 (2), 2468–2480. 10.1021/acsnano.0c06287 33565857

[B93] Zhou T. Li G. Lin S. Tian T. Ma Q. Zhang Q. (2017). Electrospun poly(3-hydroxybutyrate-co-4-hydroxybutyrate)/graphene oxide scaffold: Enhanced properties and promoted *in vivo* bone repair in rats. ACS Appl. Mat. Interfaces 9 (49), 42589–42600. 10.1021/acsami.7b14267 29148704

[B94] Zhou Z. Zhang Q. Wang Y. (2022). Preparation and characterization of antibacterial and anti-inflammatory hyaluronic acid-chitosan-dexamethasone hydrogels for peri-implantitis repair. J. Biomater. Appl. 36 (7), 1141–1150. 10.1177/08853282211047939 34605300

