# Supplementary figures and images for: Electrophoretic-deposited MXene titanium coatings in regulating bacteria and cell response for peri-implantitis (part 1 of 2)
Source: Front Chem. 2022 Sep 29;10:991481. doi: 10.3389/fchem.2022.991481 (PMC9558740; doi:10.3389/fchem.2022.991481)

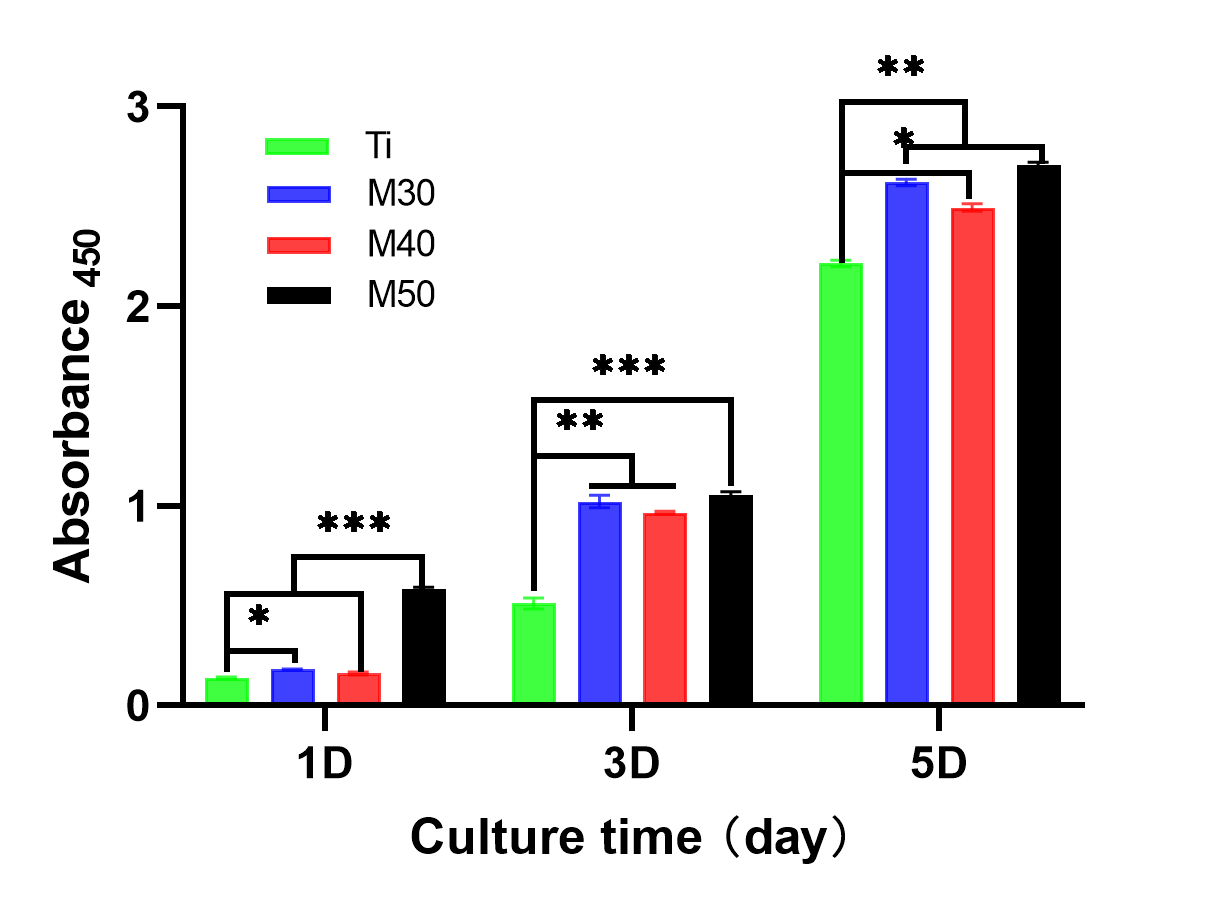

Supplement: Supplementary file 1 [file DataSheet3.ZIP › In vitro cytocompatibility assay-CCK8/Results/Data 1.tif]

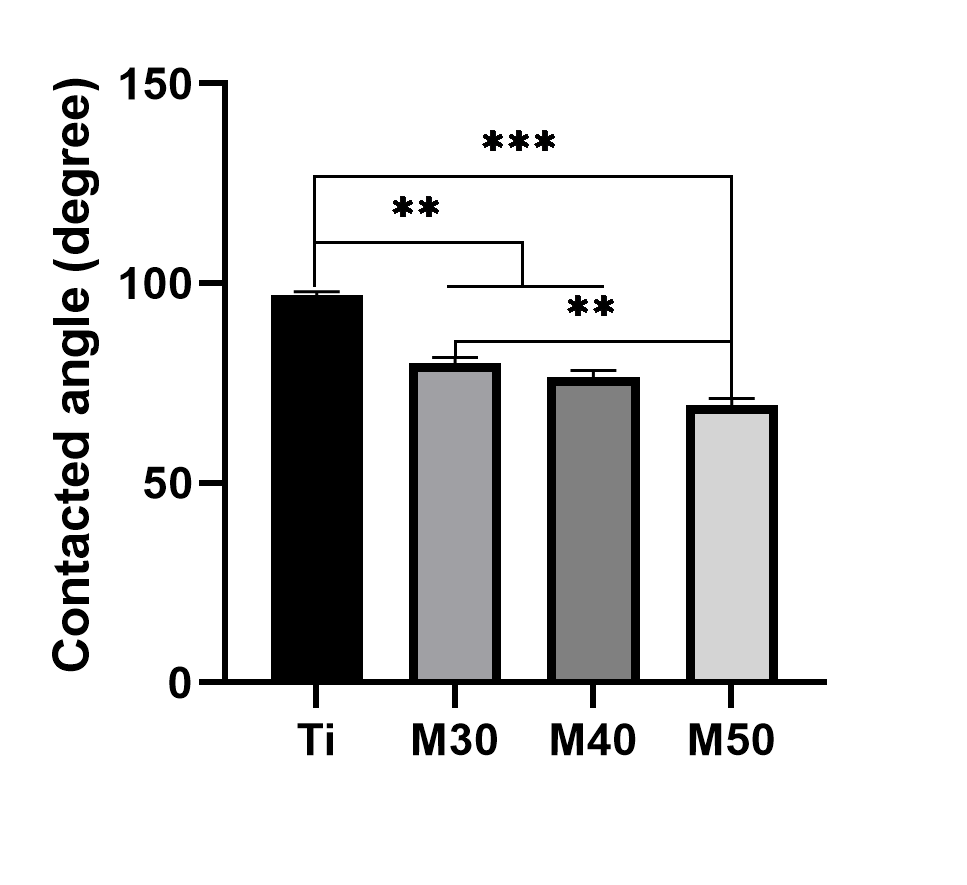

Supplement: Supplementary file 2 [file DataSheet4.ZIP › Surface characterization data/Static water contact angle/Data 1.tif]

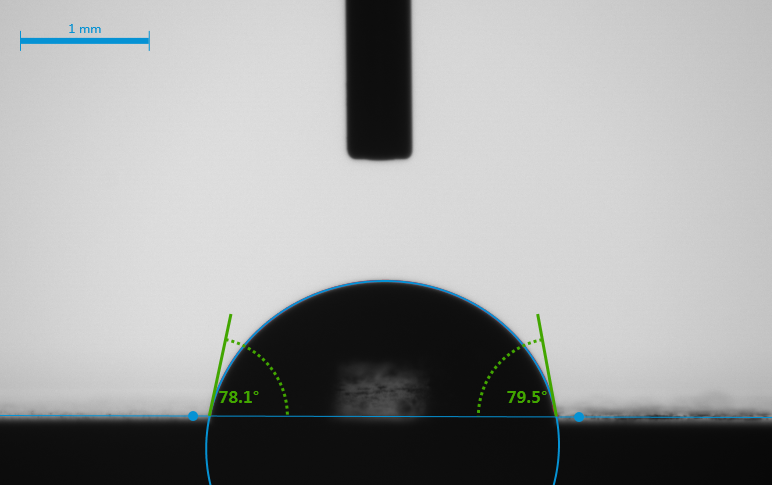

Supplement: Supplementary file 2 [file DataSheet4.ZIP › Surface characterization data/Static water contact angle/M30/1.png]

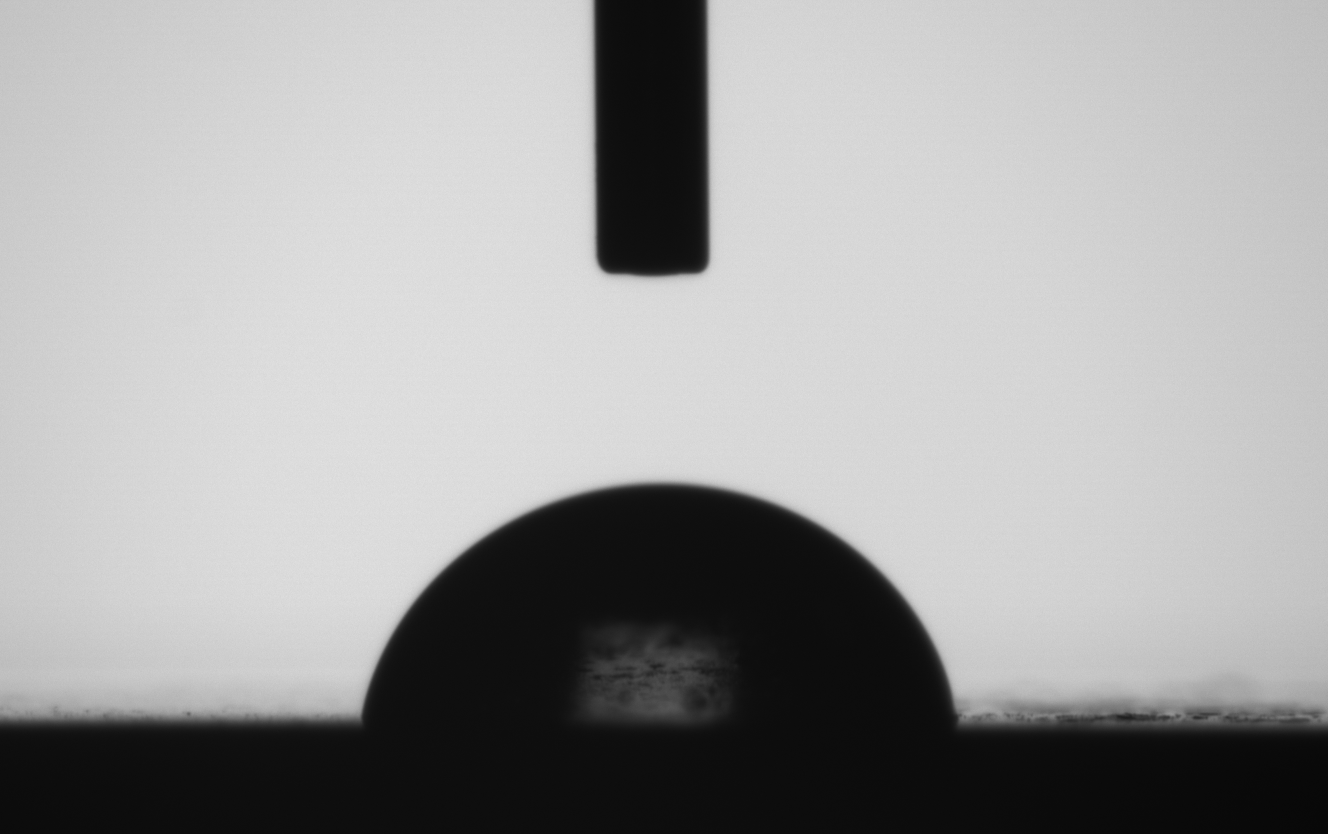

Supplement: Supplementary file 2 [file DataSheet4.ZIP › Surface characterization data/Static water contact angle/M30/1-1.png]

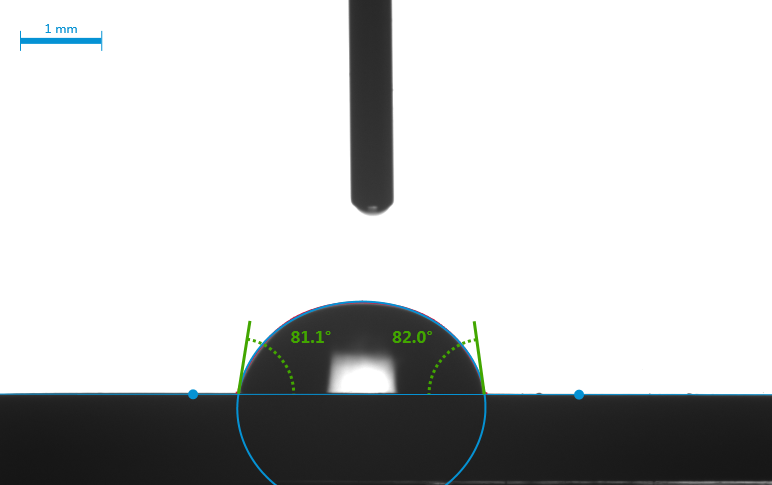

Supplement: Supplementary file 2 [file DataSheet4.ZIP › Surface characterization data/Static water contact angle/M30/2.png]

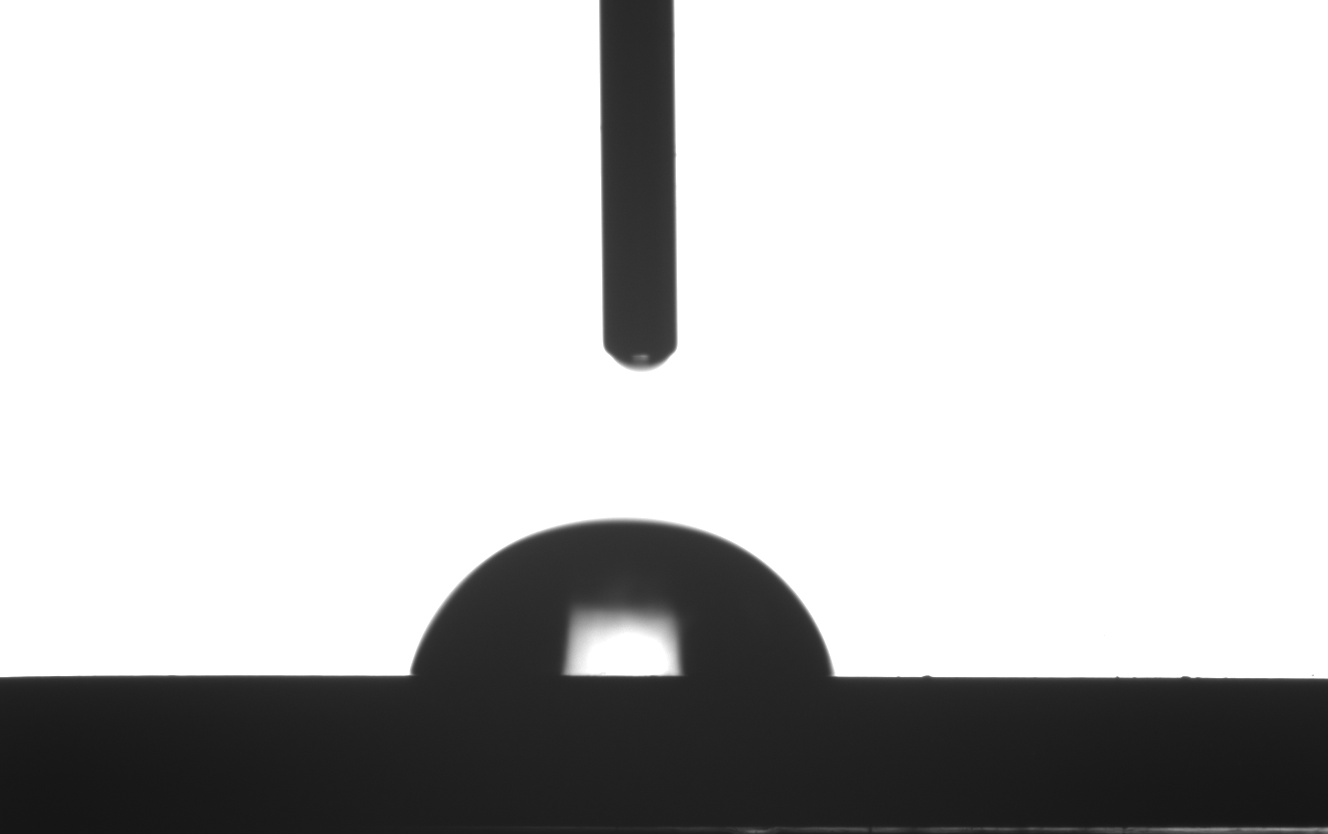

Supplement: Supplementary file 2 [file DataSheet4.ZIP › Surface characterization data/Static water contact angle/M30/2-1.png]

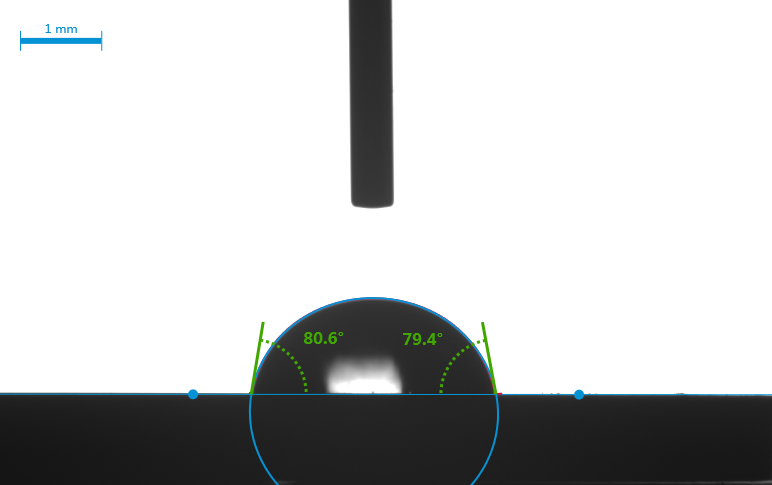

Supplement: Supplementary file 2 [file DataSheet4.ZIP › Surface characterization data/Static water contact angle/M30/3.png]

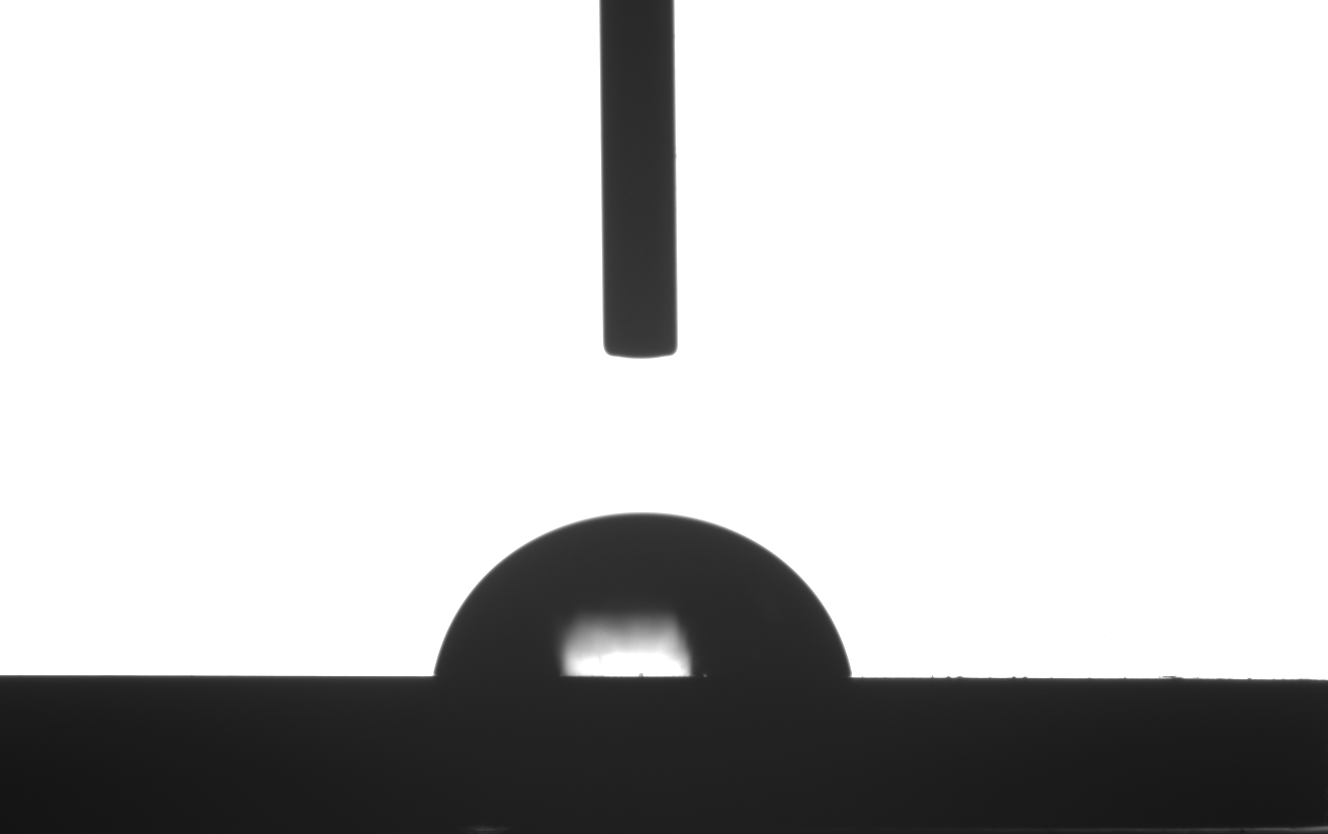

Supplement: Supplementary file 2 [file DataSheet4.ZIP › Surface characterization data/Static water contact angle/M30/3-1.png]

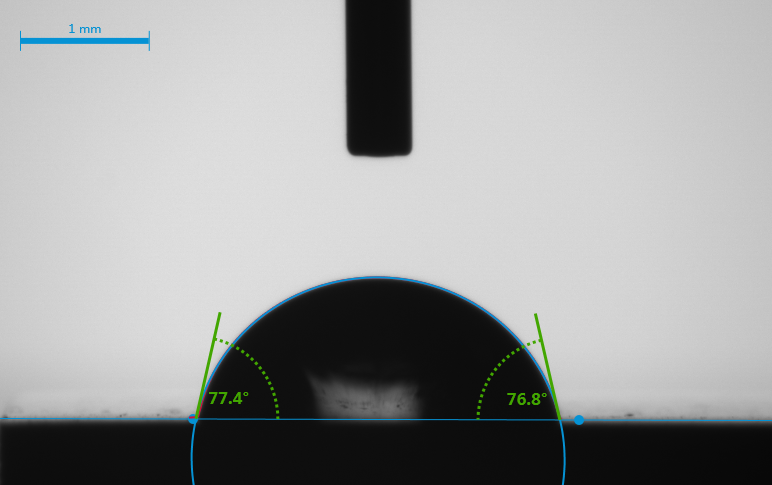

Supplement: Supplementary file 2 [file DataSheet4.ZIP › Surface characterization data/Static water contact angle/M40/1.png]

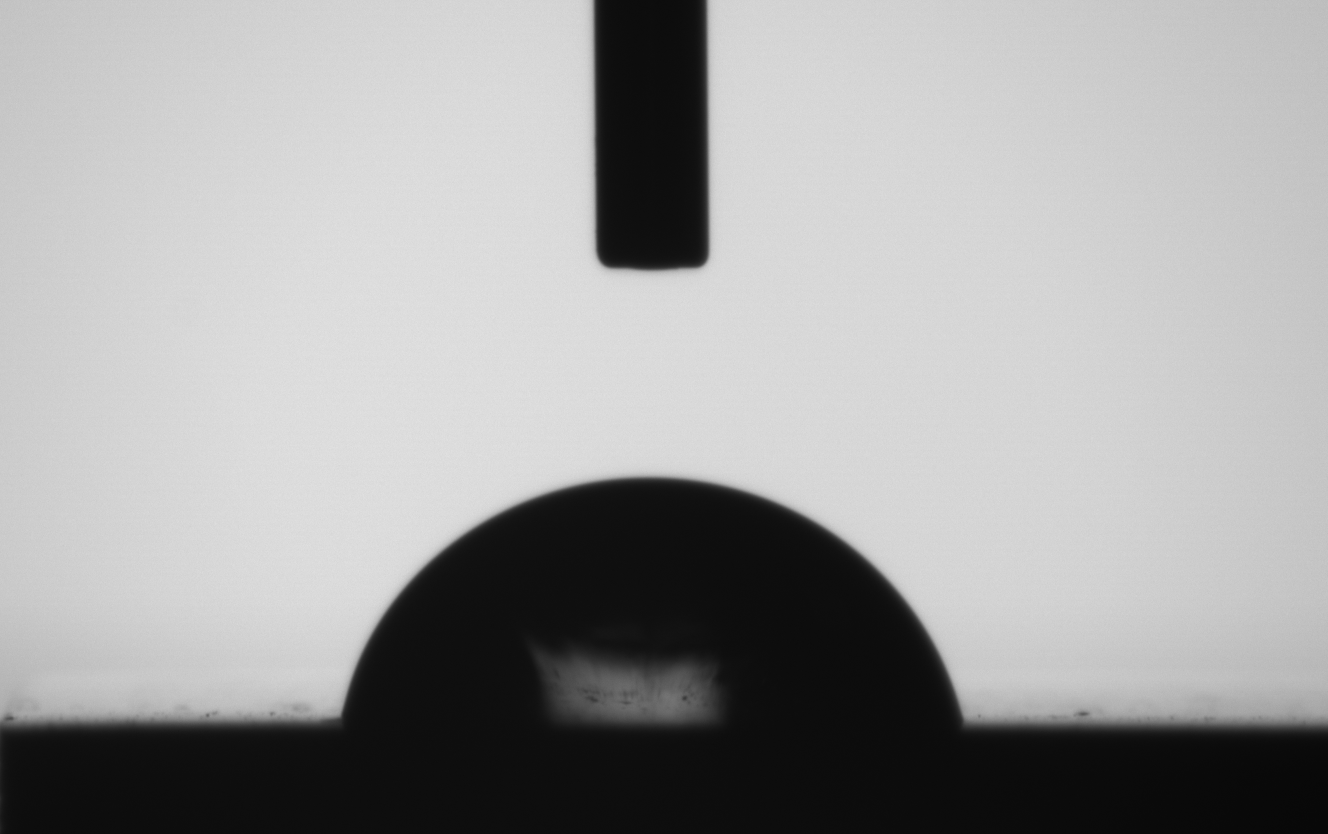

Supplement: Supplementary file 2 [file DataSheet4.ZIP › Surface characterization data/Static water contact angle/M40/1-1.png]

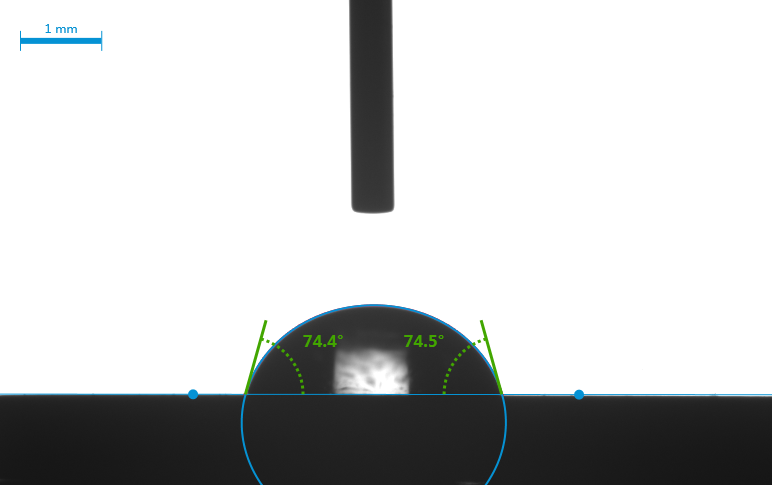

Supplement: Supplementary file 2 [file DataSheet4.ZIP › Surface characterization data/Static water contact angle/M40/2.png]

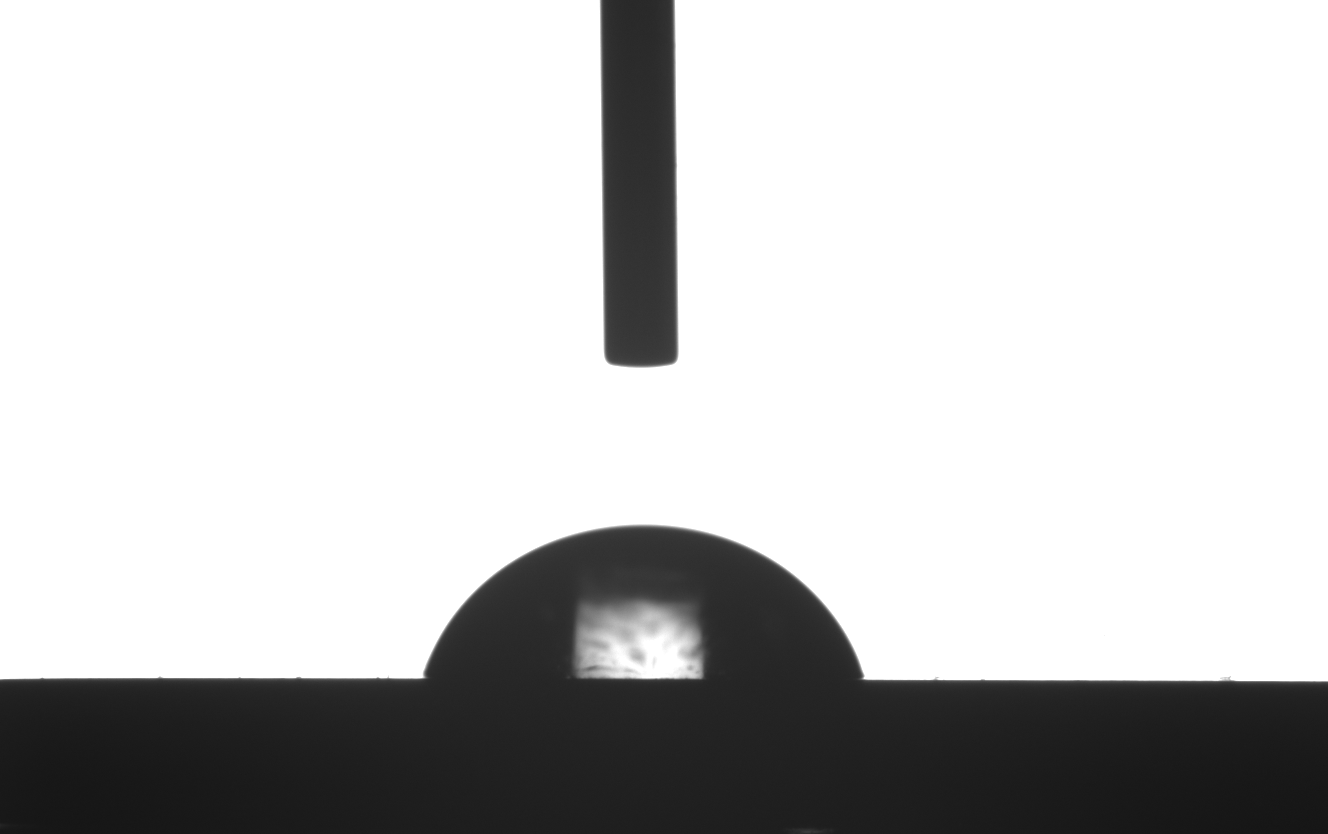

Supplement: Supplementary file 2 [file DataSheet4.ZIP › Surface characterization data/Static water contact angle/M40/2-1.png]

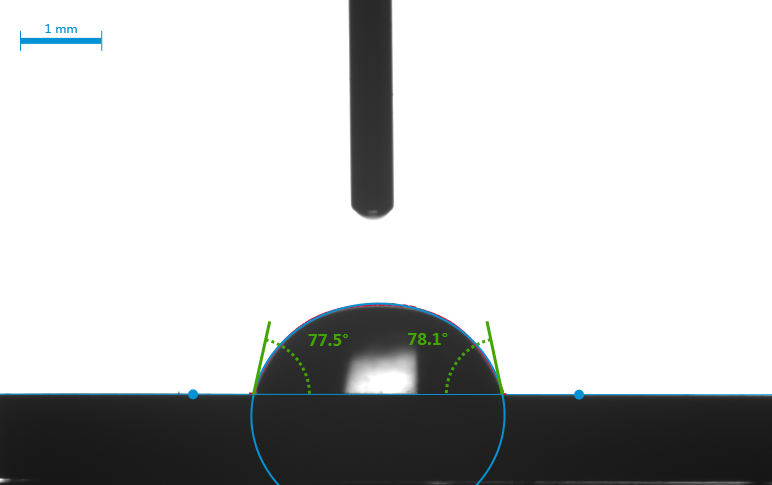

Supplement: Supplementary file 2 [file DataSheet4.ZIP › Surface characterization data/Static water contact angle/M40/3.png]

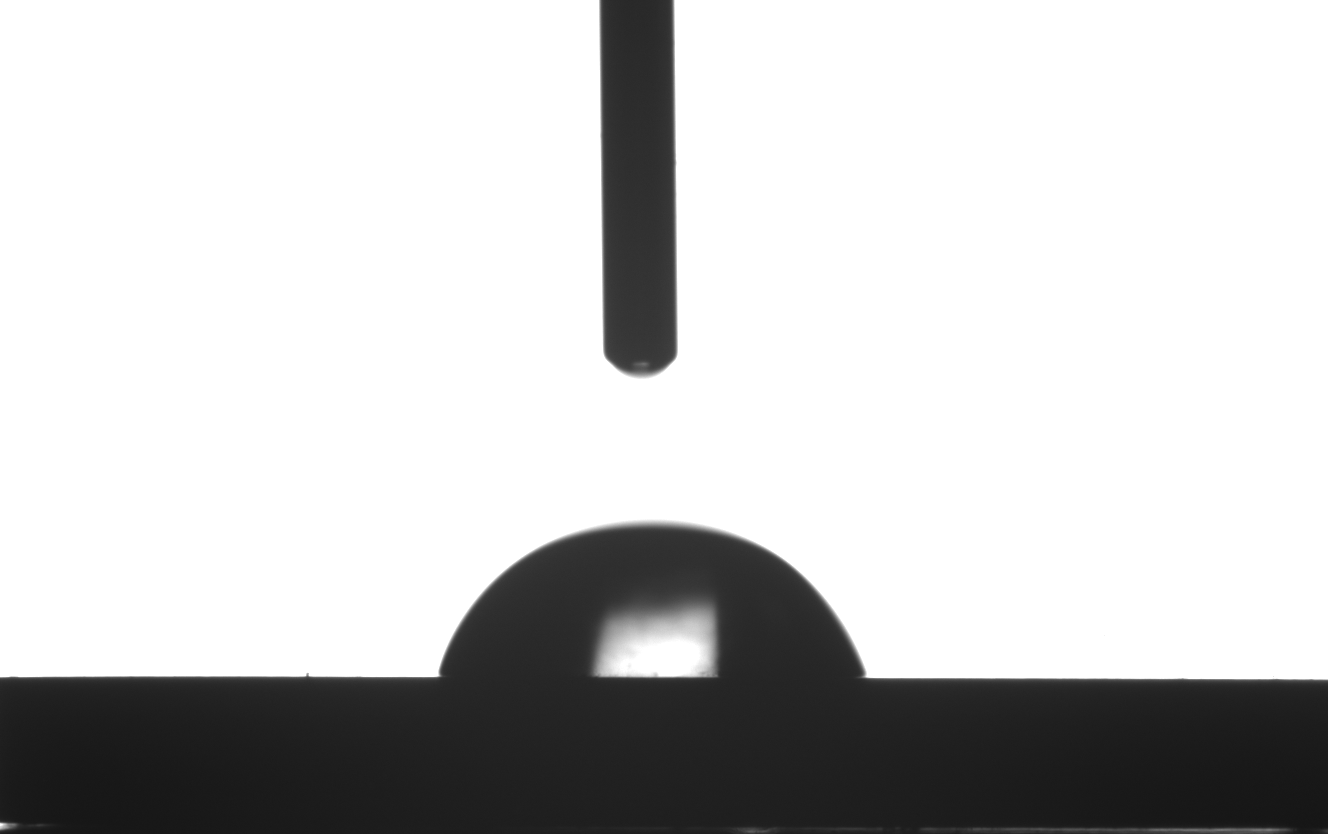

Supplement: Supplementary file 2 [file DataSheet4.ZIP › Surface characterization data/Static water contact angle/M40/3-1.png]

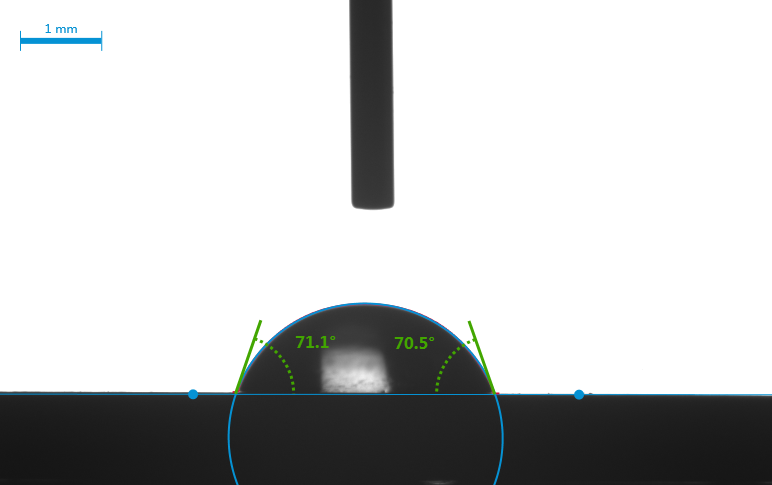

Supplement: Supplementary file 2 [file DataSheet4.ZIP › Surface characterization data/Static water contact angle/M50/1.png]

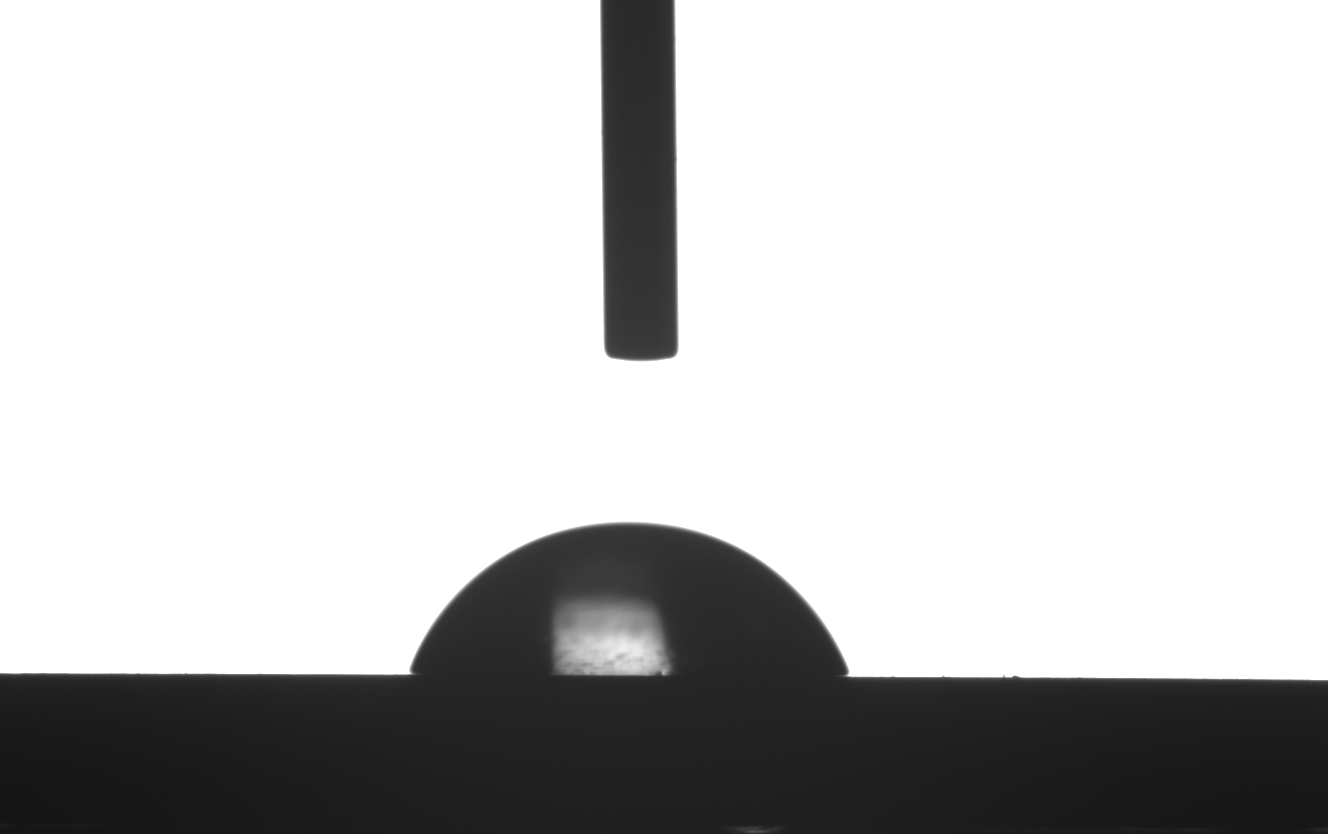

Supplement: Supplementary file 2 [file DataSheet4.ZIP › Surface characterization data/Static water contact angle/M50/1-1.png]

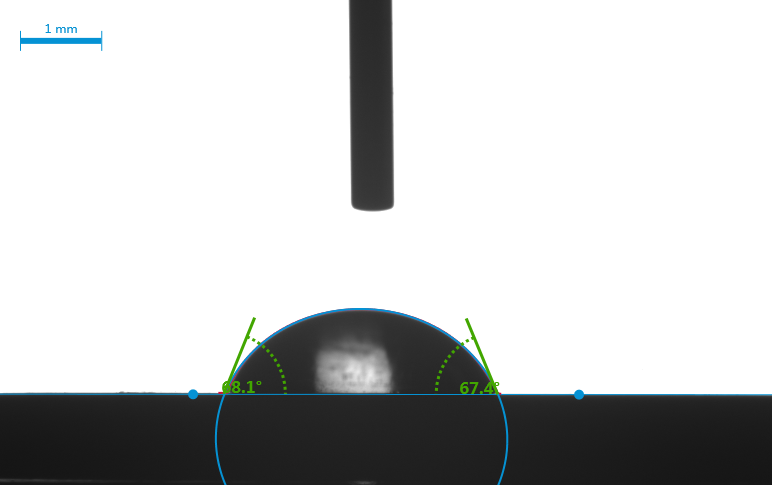

Supplement: Supplementary file 2 [file DataSheet4.ZIP › Surface characterization data/Static water contact angle/M50/2.png]

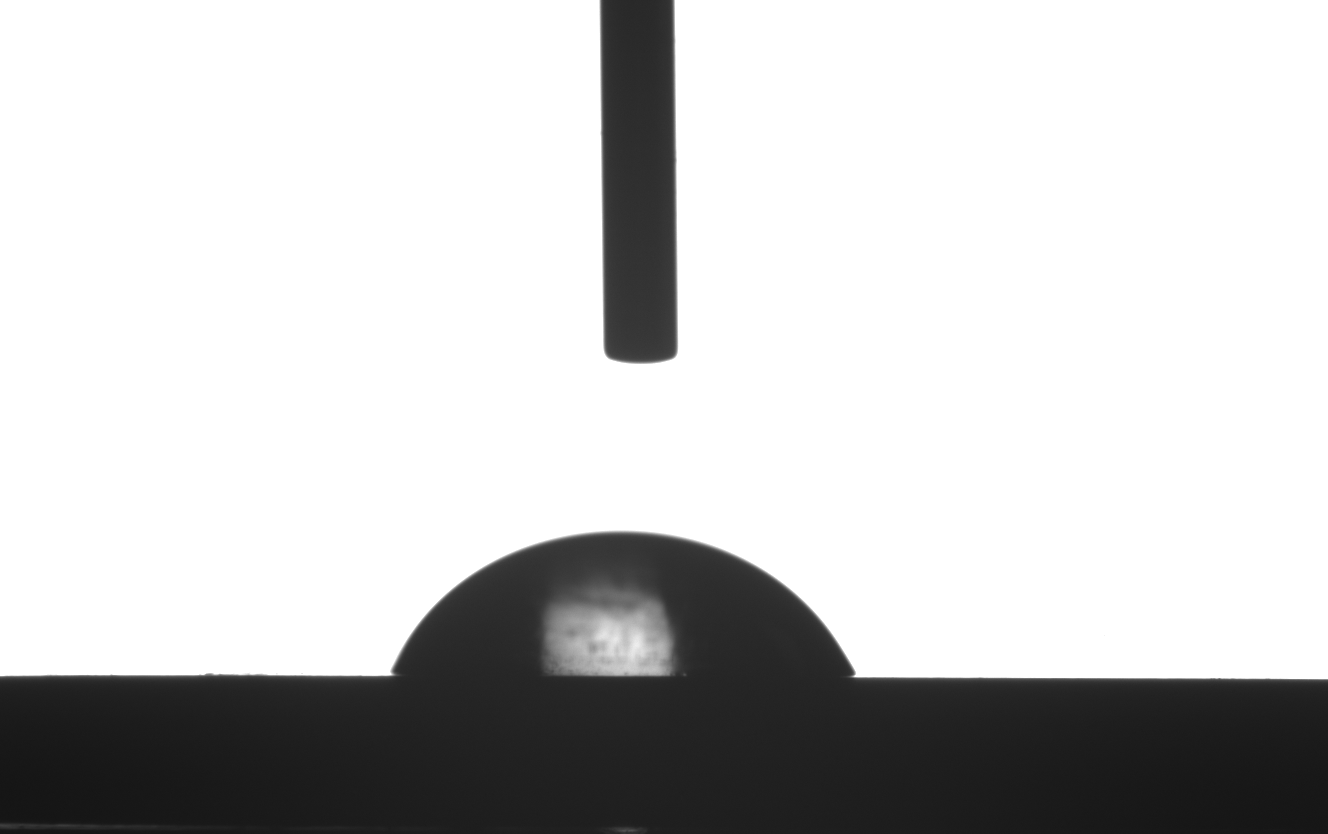

Supplement: Supplementary file 2 [file DataSheet4.ZIP › Surface characterization data/Static water contact angle/M50/2-1.png]

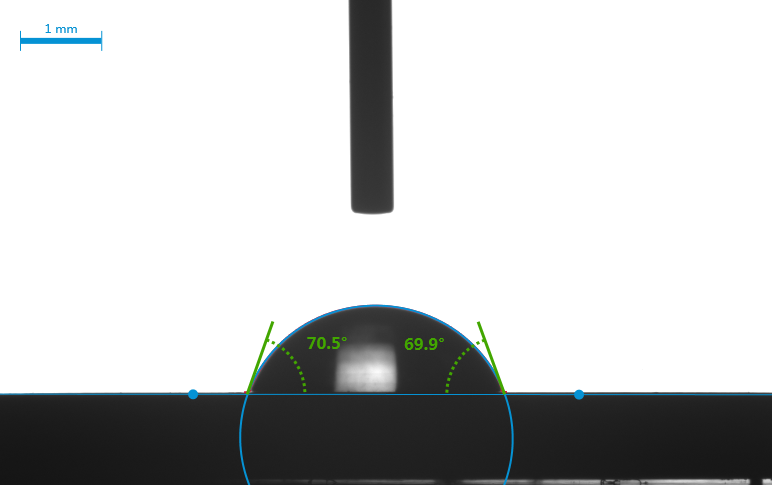

Supplement: Supplementary file 2 [file DataSheet4.ZIP › Surface characterization data/Static water contact angle/M50/3.png]

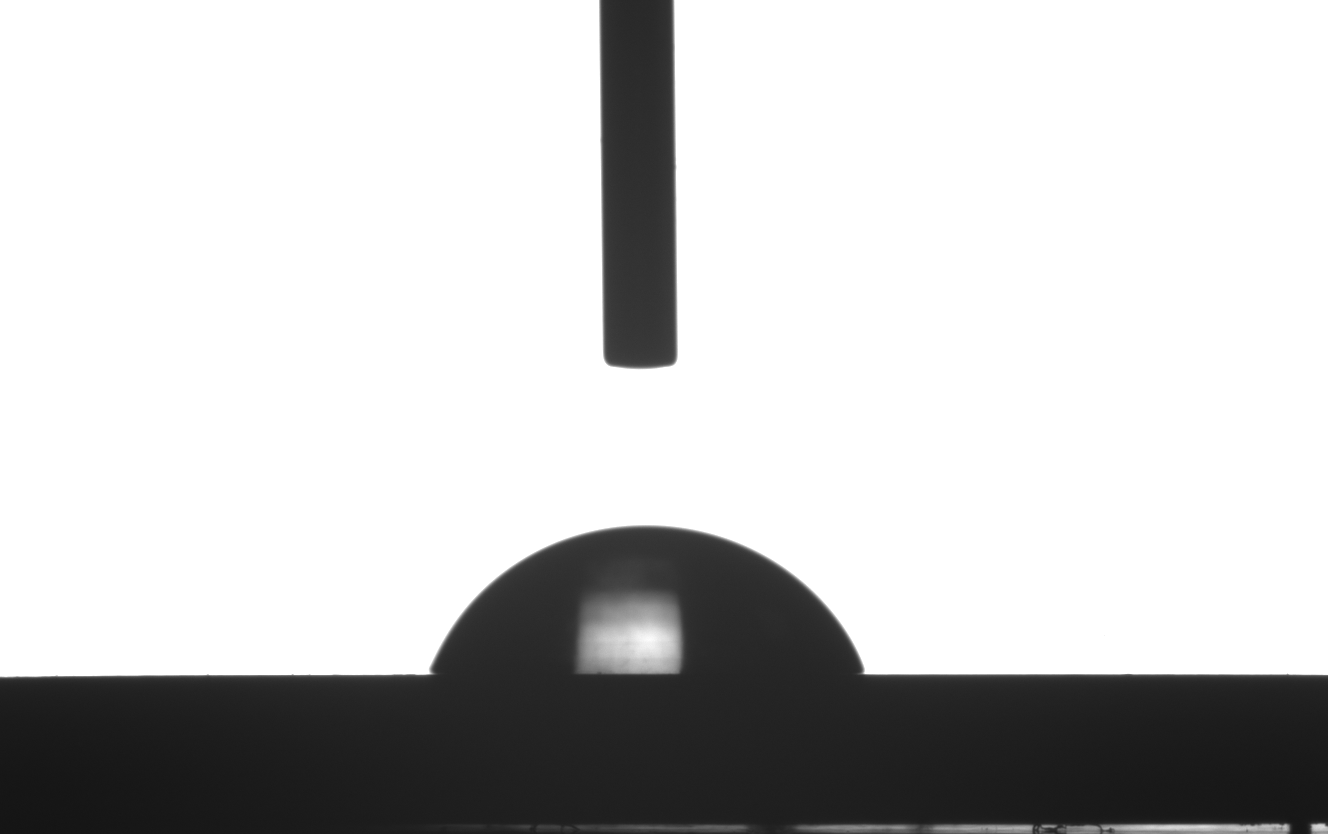

Supplement: Supplementary file 2 [file DataSheet4.ZIP › Surface characterization data/Static water contact angle/M50/3-1.png]

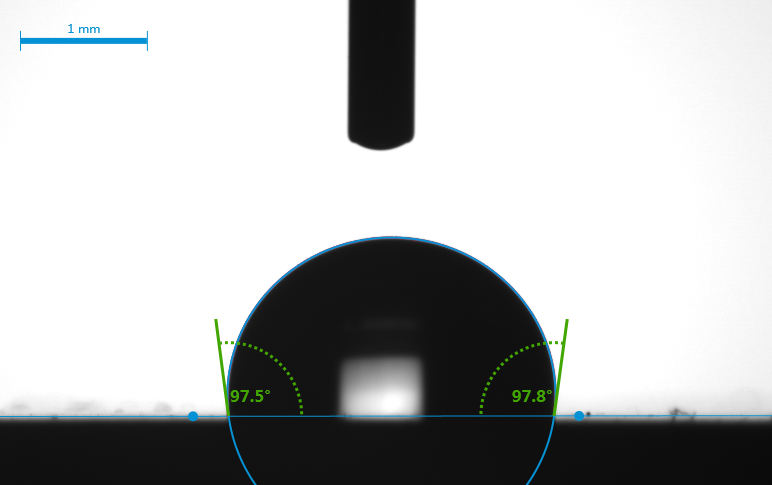

Supplement: Supplementary file 2 [file DataSheet4.ZIP › Surface characterization data/Static water contact angle/Ti/1.png]

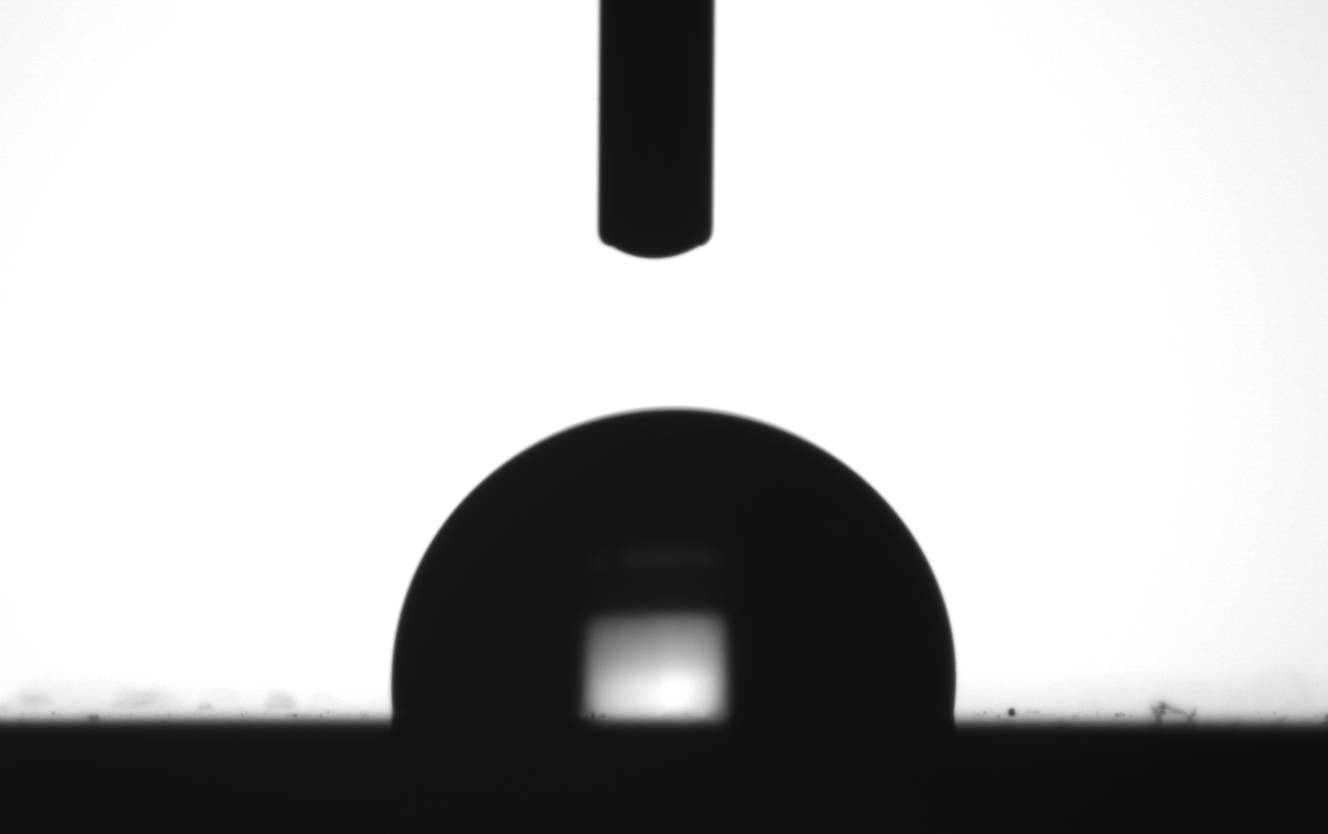

Supplement: Supplementary file 2 [file DataSheet4.ZIP › Surface characterization data/Static water contact angle/Ti/1-1.png]

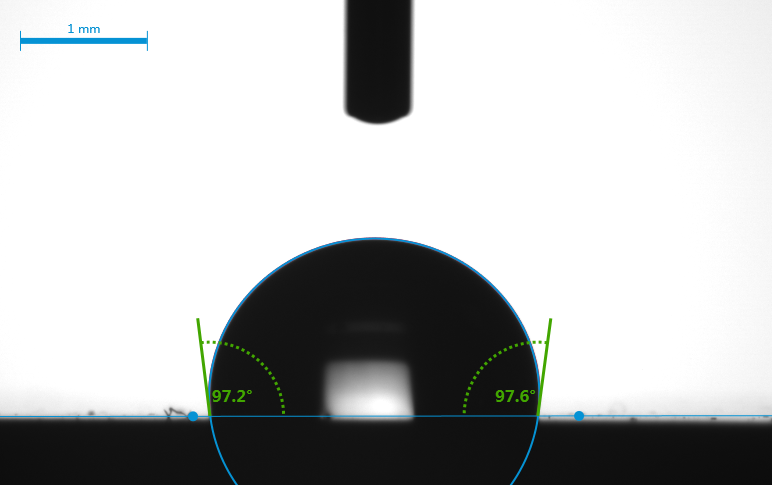

Supplement: Supplementary file 2 [file DataSheet4.ZIP › Surface characterization data/Static water contact angle/Ti/2.png]

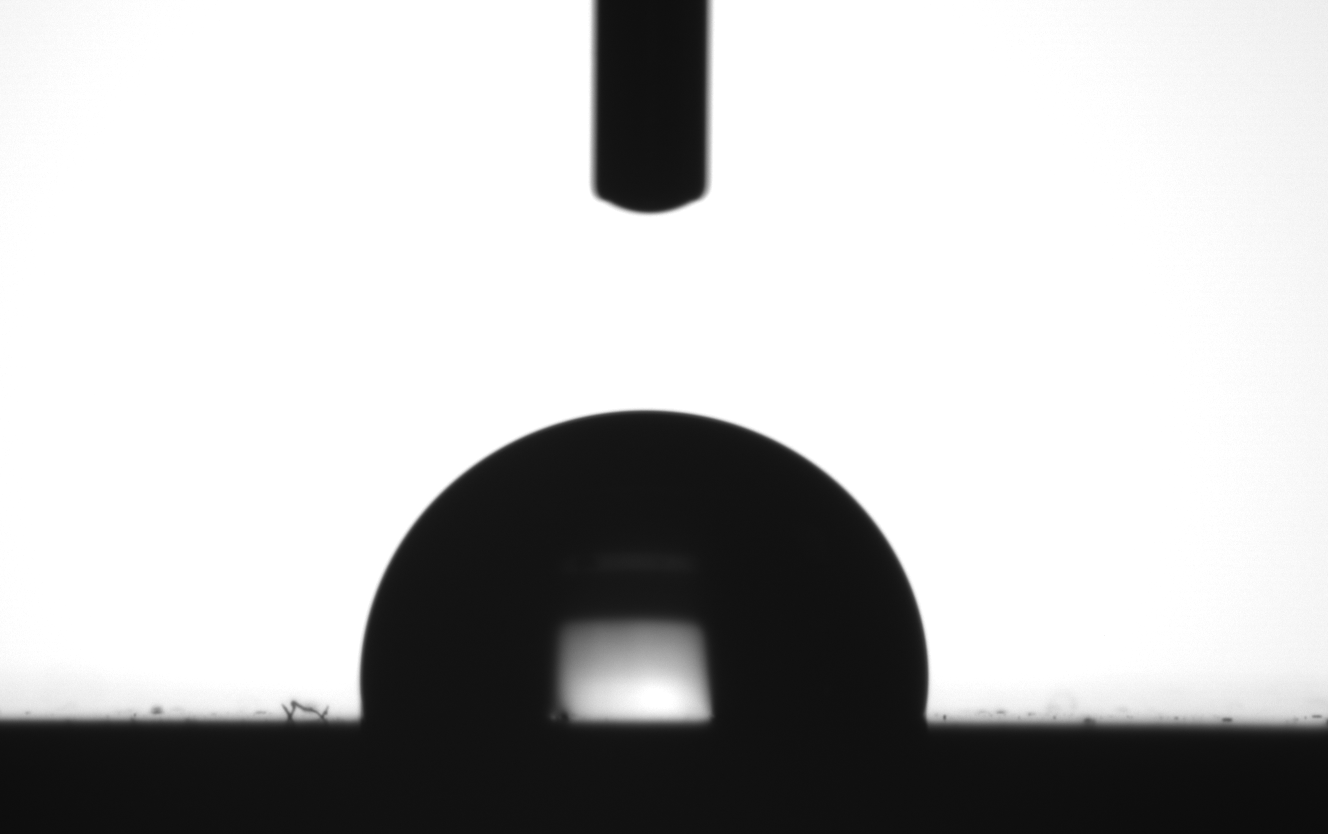

Supplement: Supplementary file 2 [file DataSheet4.ZIP › Surface characterization data/Static water contact angle/Ti/2-1.png]

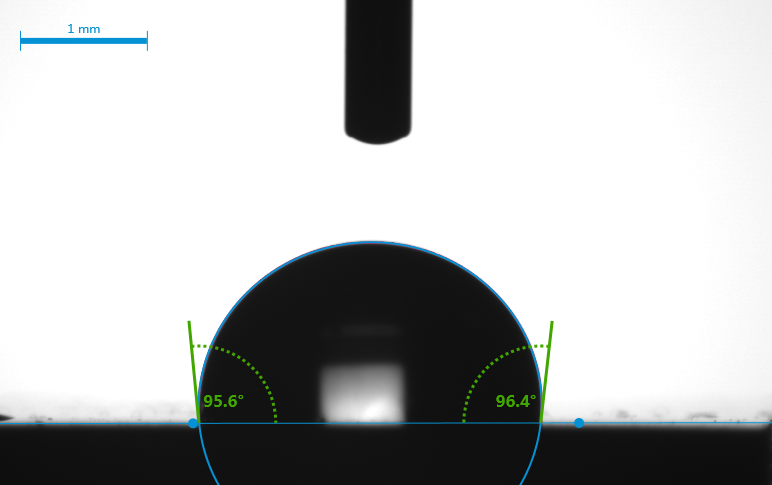

Supplement: Supplementary file 2 [file DataSheet4.ZIP › Surface characterization data/Static water contact angle/Ti/3.png]

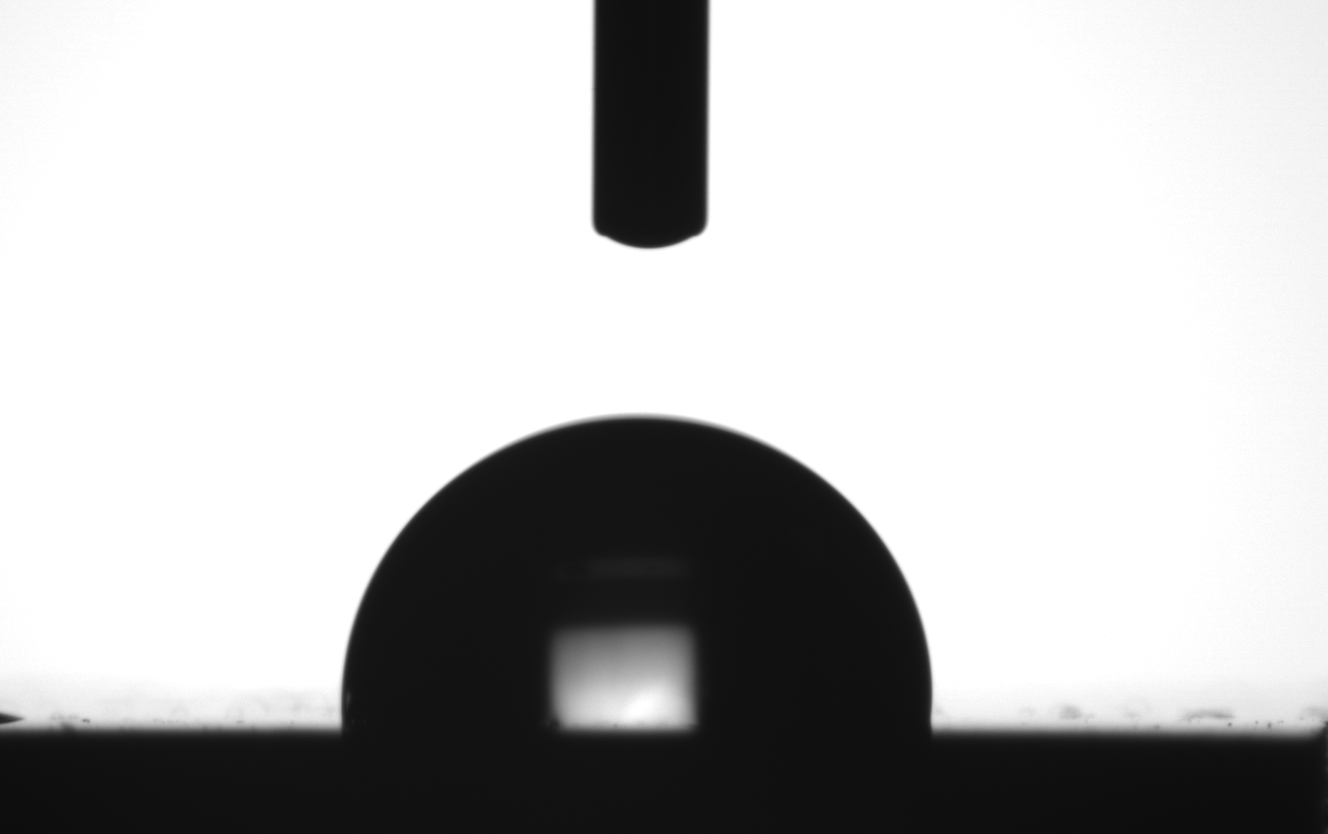

Supplement: Supplementary file 2 [file DataSheet4.ZIP › Surface characterization data/Static water contact angle/Ti/3-1.png]

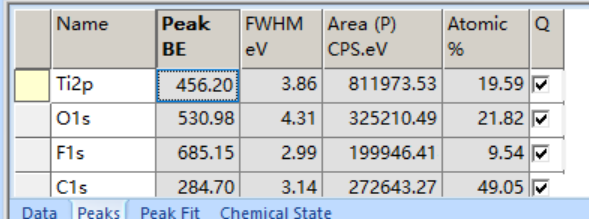

Supplement: Supplementary file 2 [file DataSheet4.ZIP › Surface characterization data/XPS/M30.png]

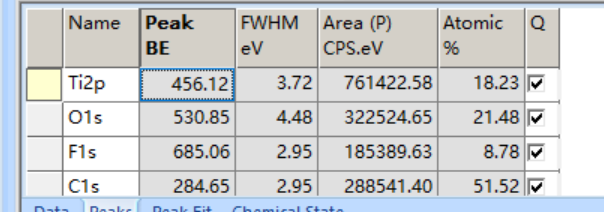

Supplement: Supplementary file 2 [file DataSheet4.ZIP › Surface characterization data/XPS/M40.png]

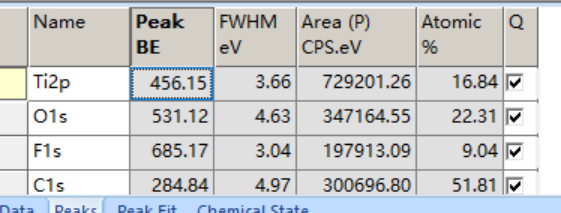

Supplement: Supplementary file 2 [file DataSheet4.ZIP › Surface characterization data/XPS/M50.png]

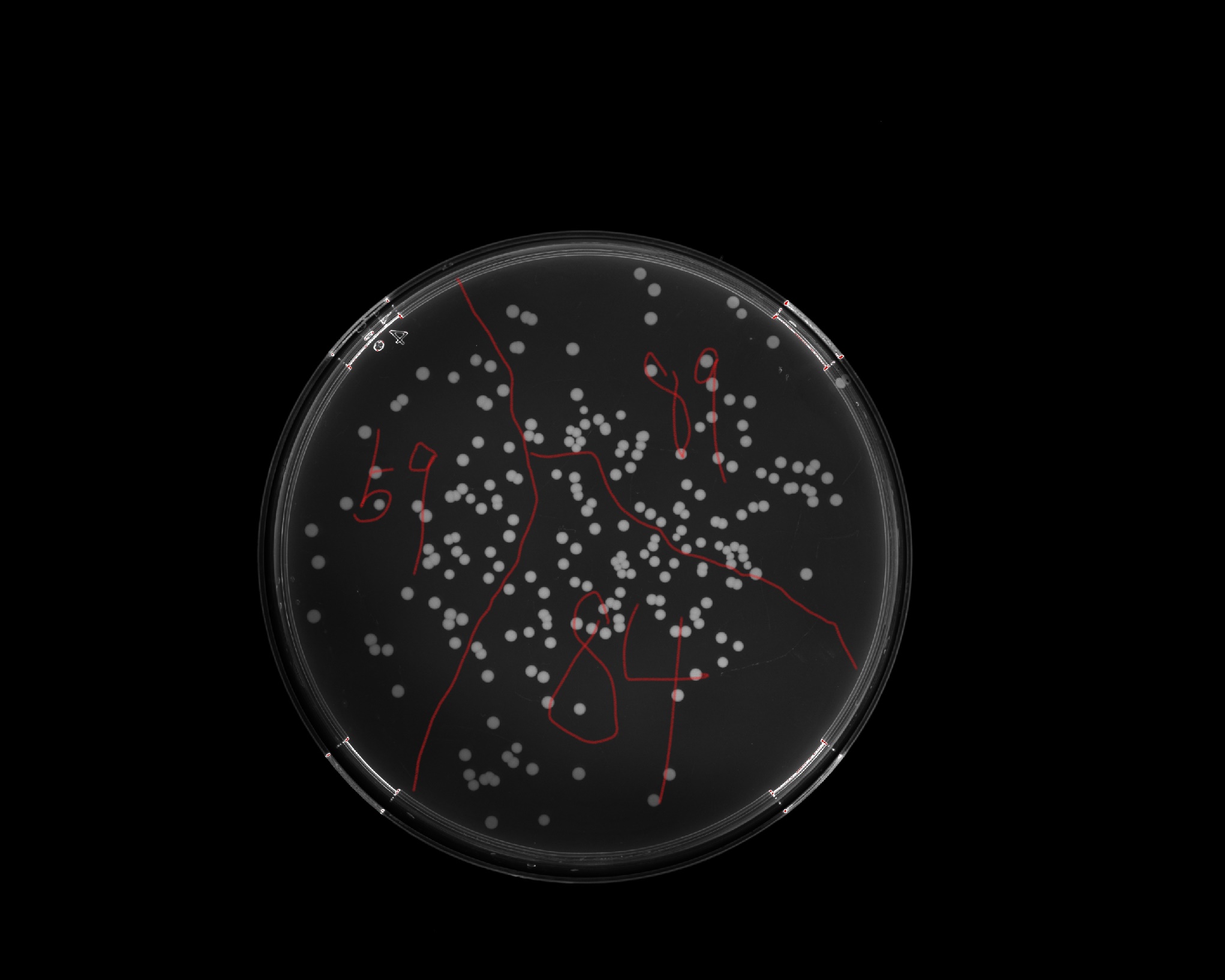

Supplement: Supplementary file 3 [file DataSheet1.ZIP › Antibacterial activity/Agar reculture observation/MRSA/Counting/InkedM 30-1000(SYBR Gold)_LI.jpg]

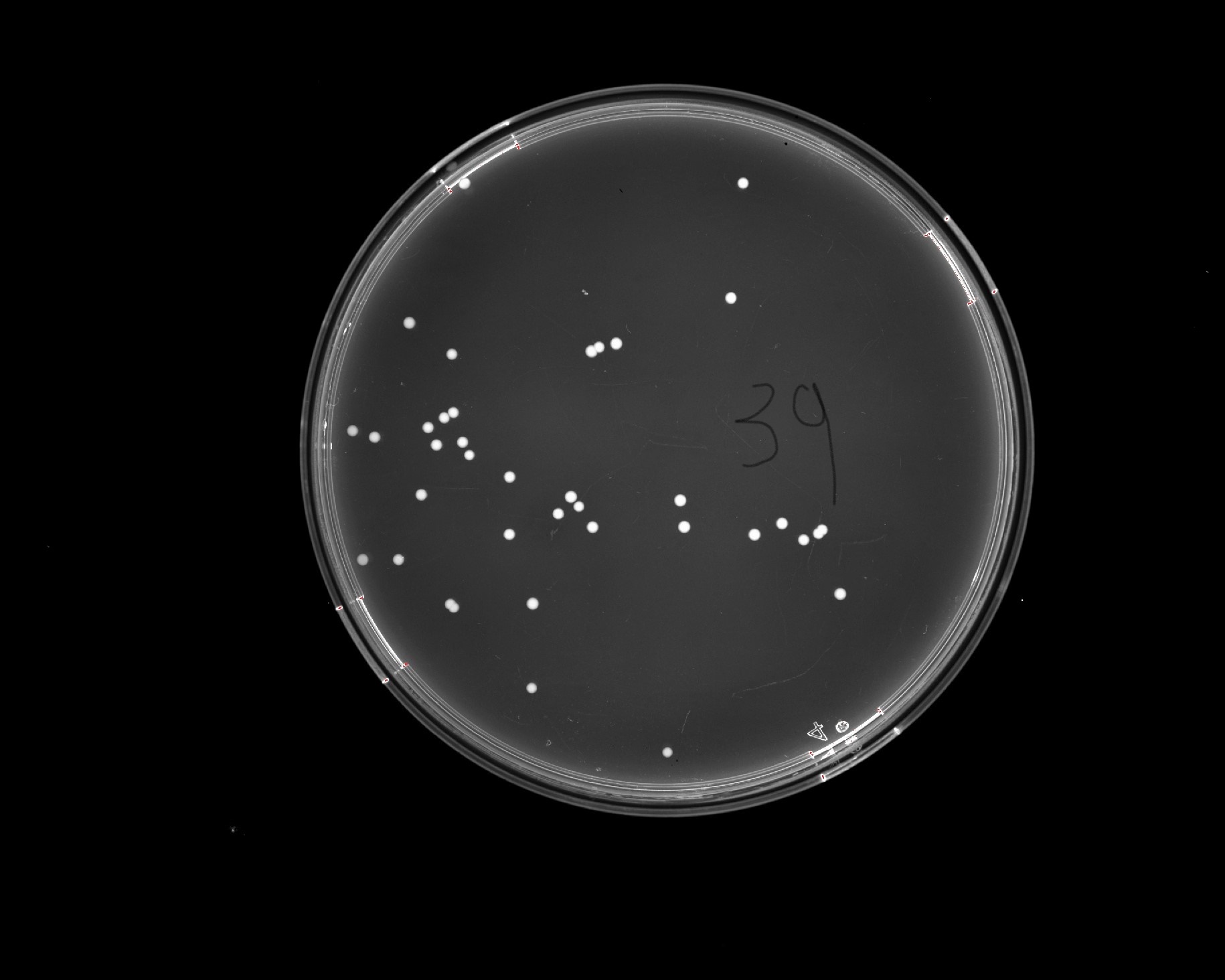

Supplement: Supplementary file 3 [file DataSheet1.ZIP › Antibacterial activity/Agar reculture observation/MRSA/Counting/InkedM 40-1000(SYBR Gold)_LI.jpg]

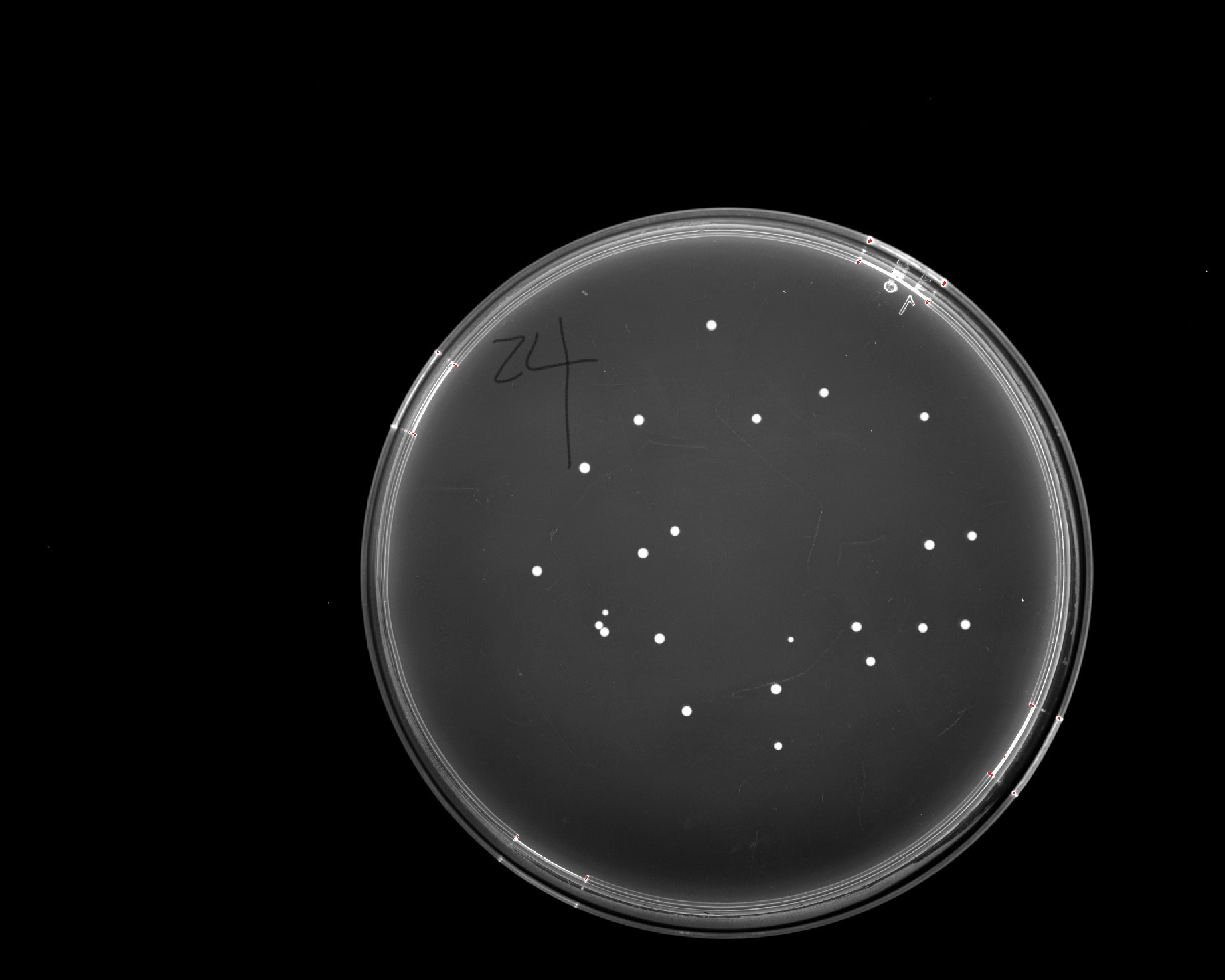

Supplement: Supplementary file 3 [file DataSheet1.ZIP › Antibacterial activity/Agar reculture observation/MRSA/Counting/InkedM 50-1000(SYBR Gold)_LI.jpg]

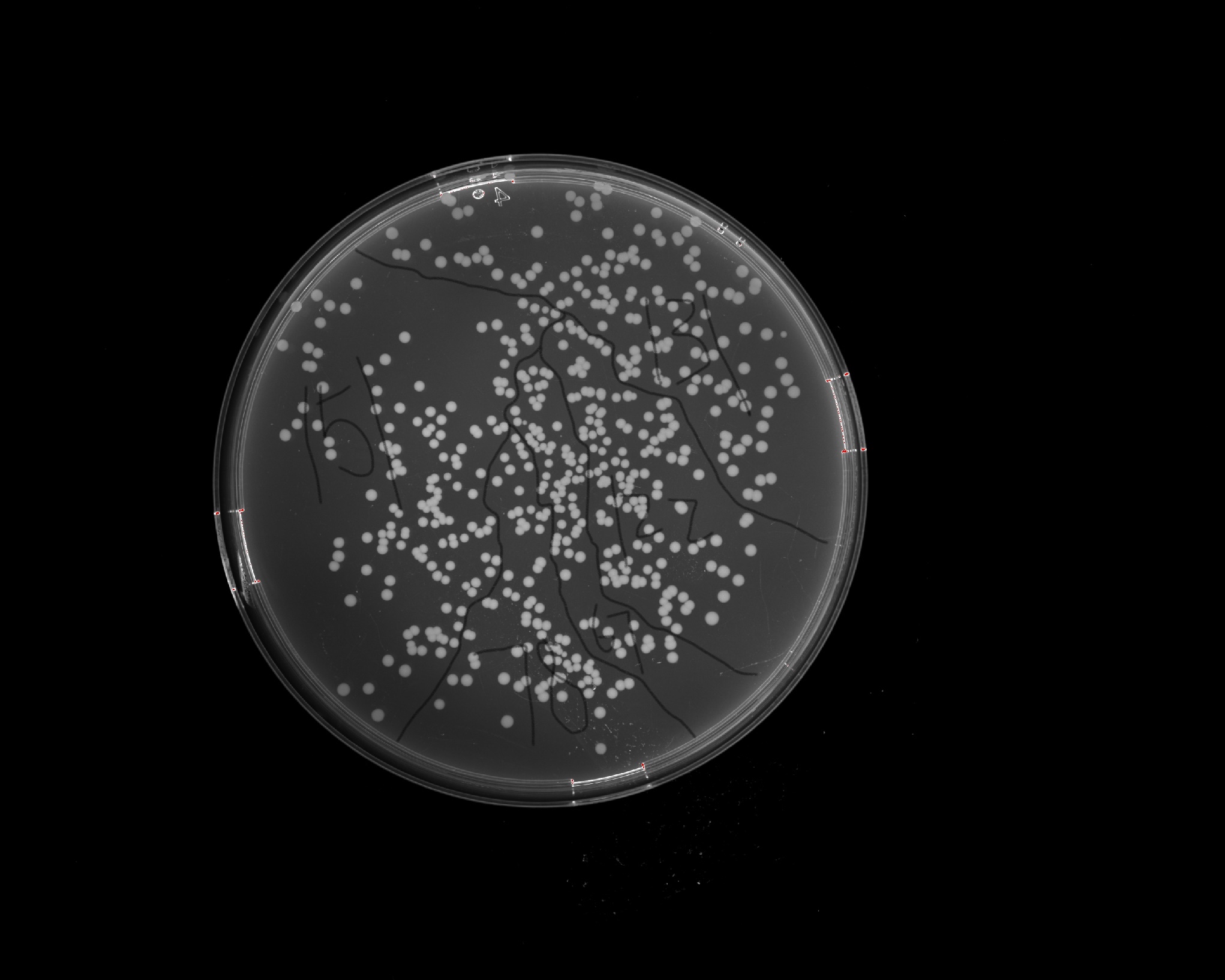

Supplement: Supplementary file 3 [file DataSheet1.ZIP › Antibacterial activity/Agar reculture observation/MRSA/Counting/InkedM Ti-1000(SYBR Gold)_LI.jpg]

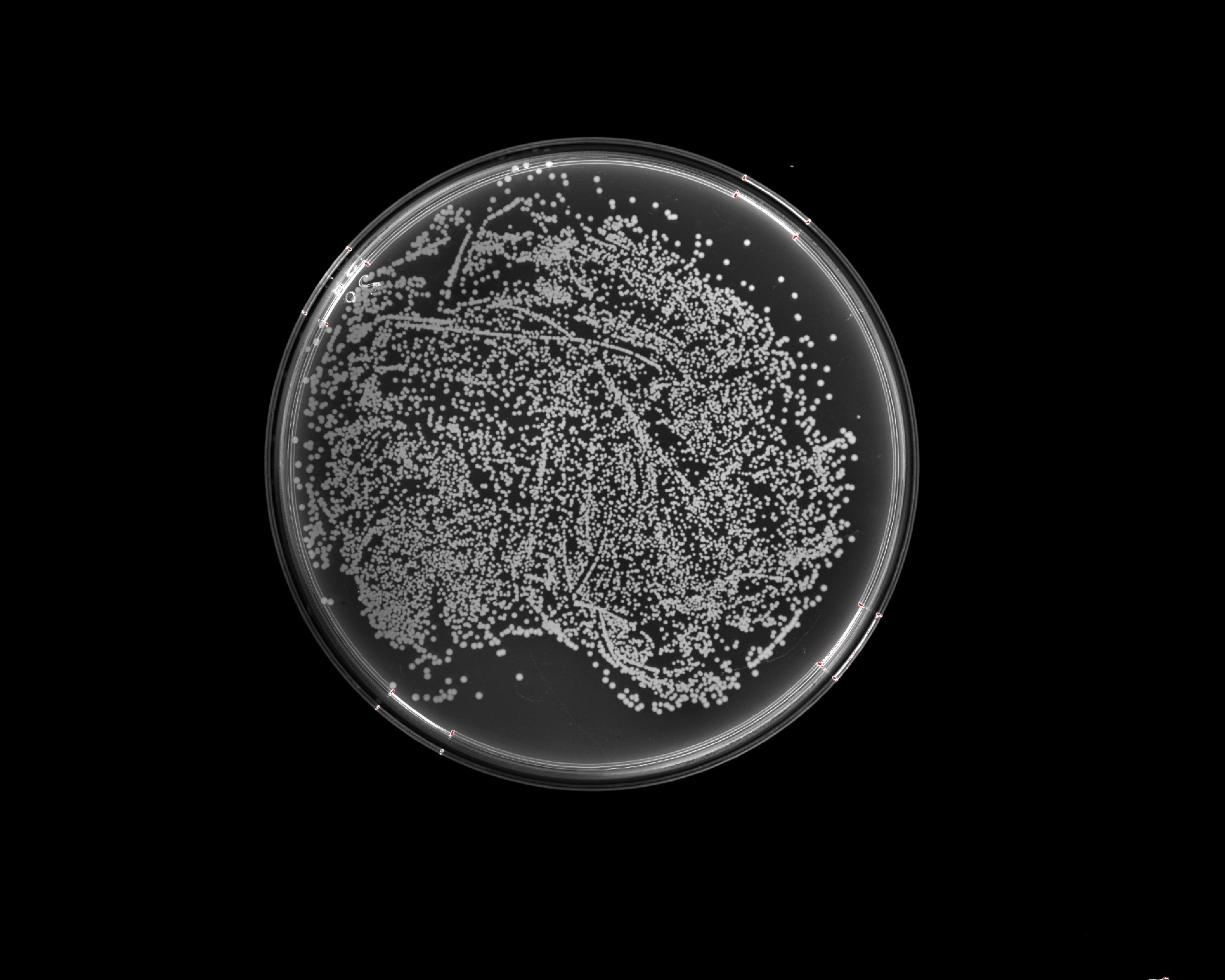

Supplement: Supplementary file 3 [file DataSheet1.ZIP › Antibacterial activity/Agar reculture observation/MRSA/M 30-10_2(SYBR Gold).jpg]

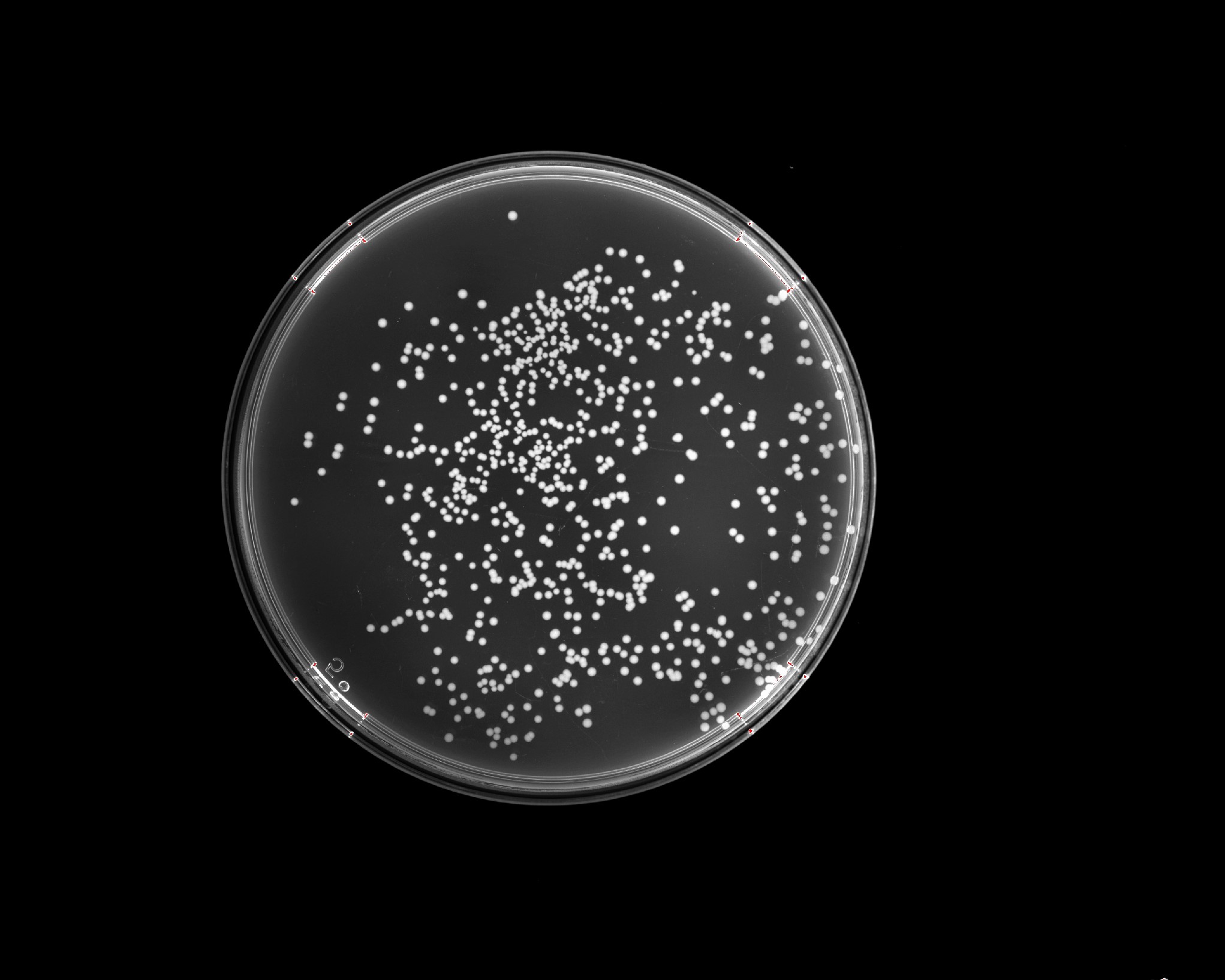

Supplement: Supplementary file 3 [file DataSheet1.ZIP › Antibacterial activity/Agar reculture observation/MRSA/M 30-100_2(SYBR Gold).jpg]

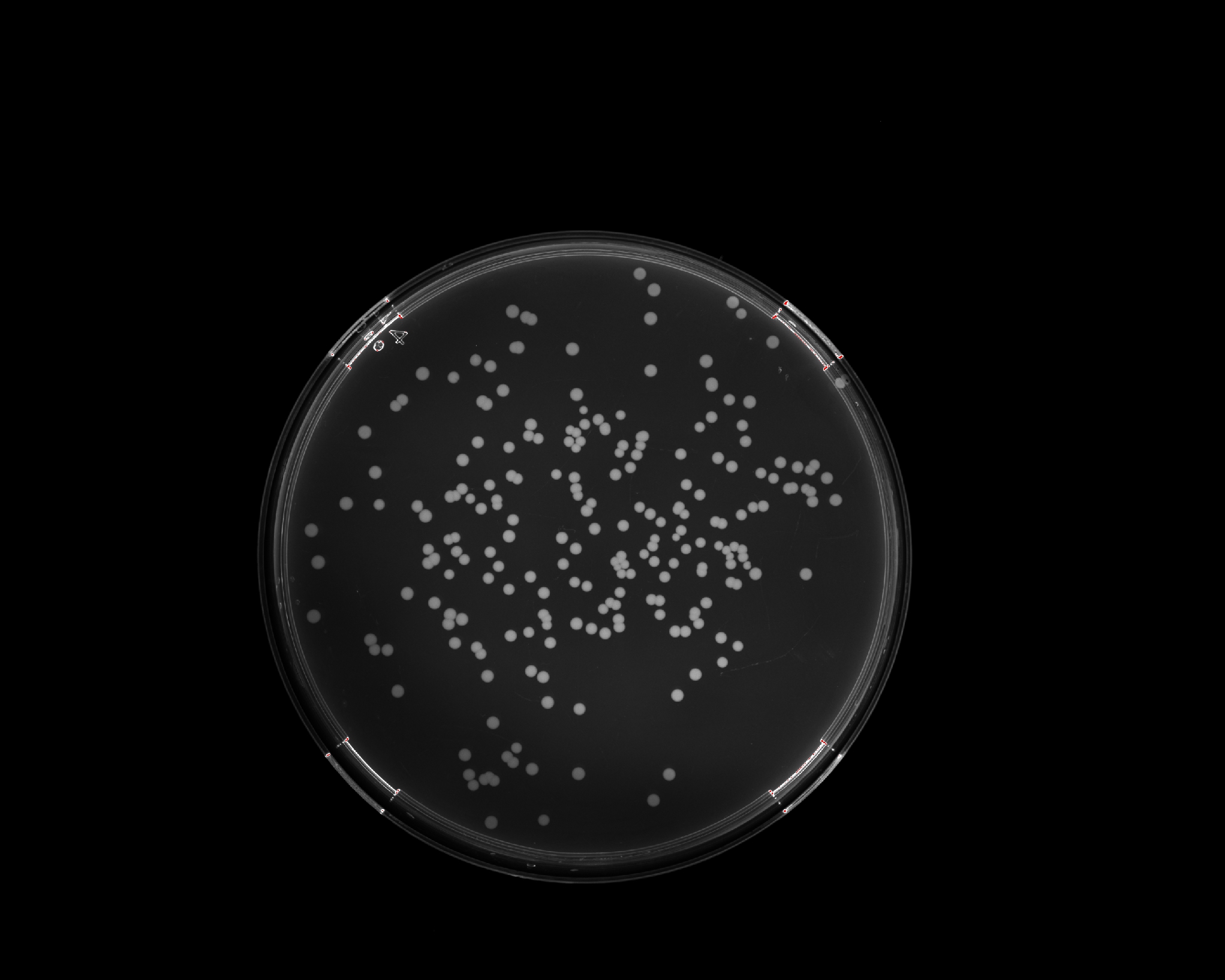

Supplement: Supplementary file 3 [file DataSheet1.ZIP › Antibacterial activity/Agar reculture observation/MRSA/M 30-1000(SYBR Gold).jpg]

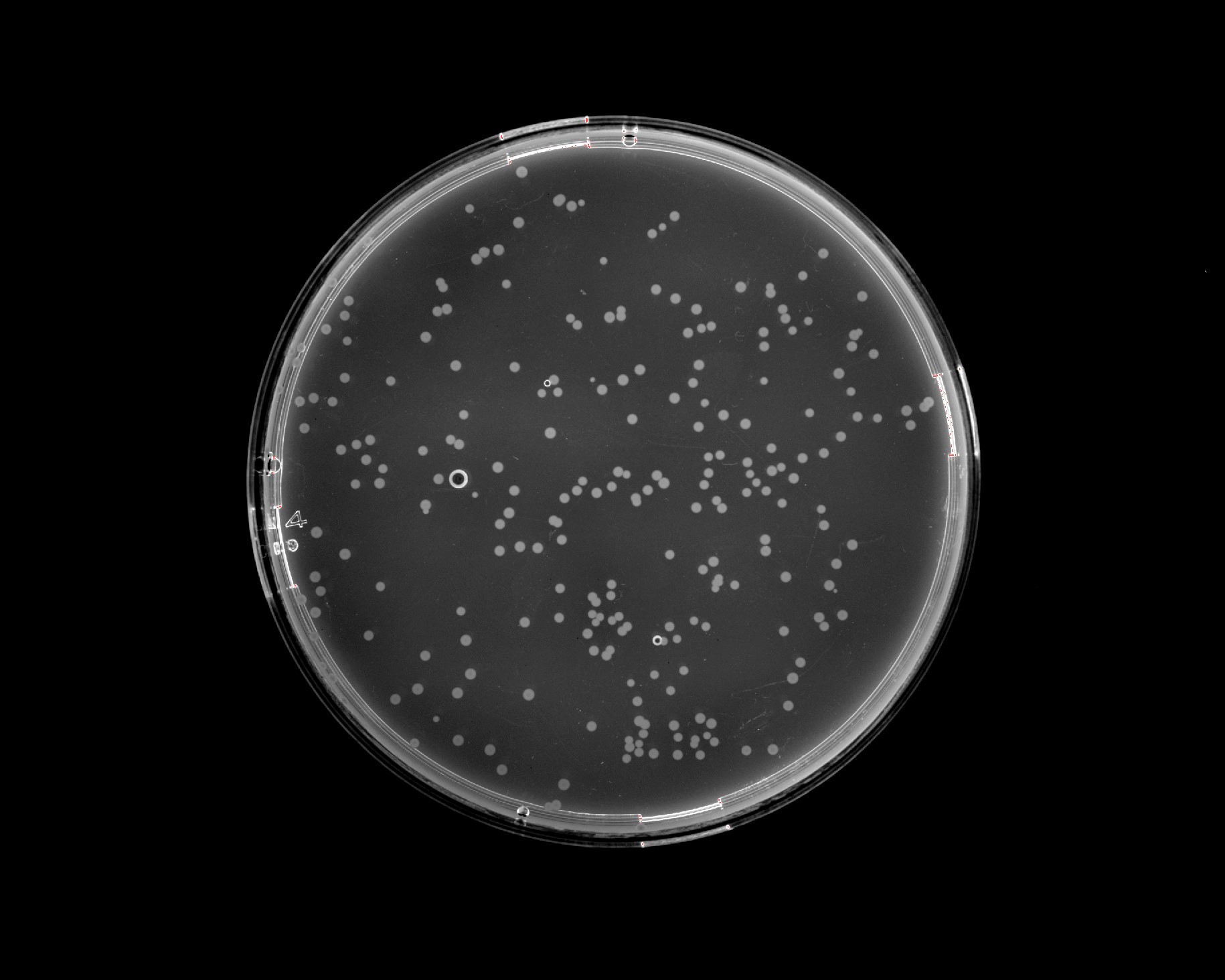

Supplement: Supplementary file 3 [file DataSheet1.ZIP › Antibacterial activity/Agar reculture observation/MRSA/M 40-10(SYBR Gold).jpg]

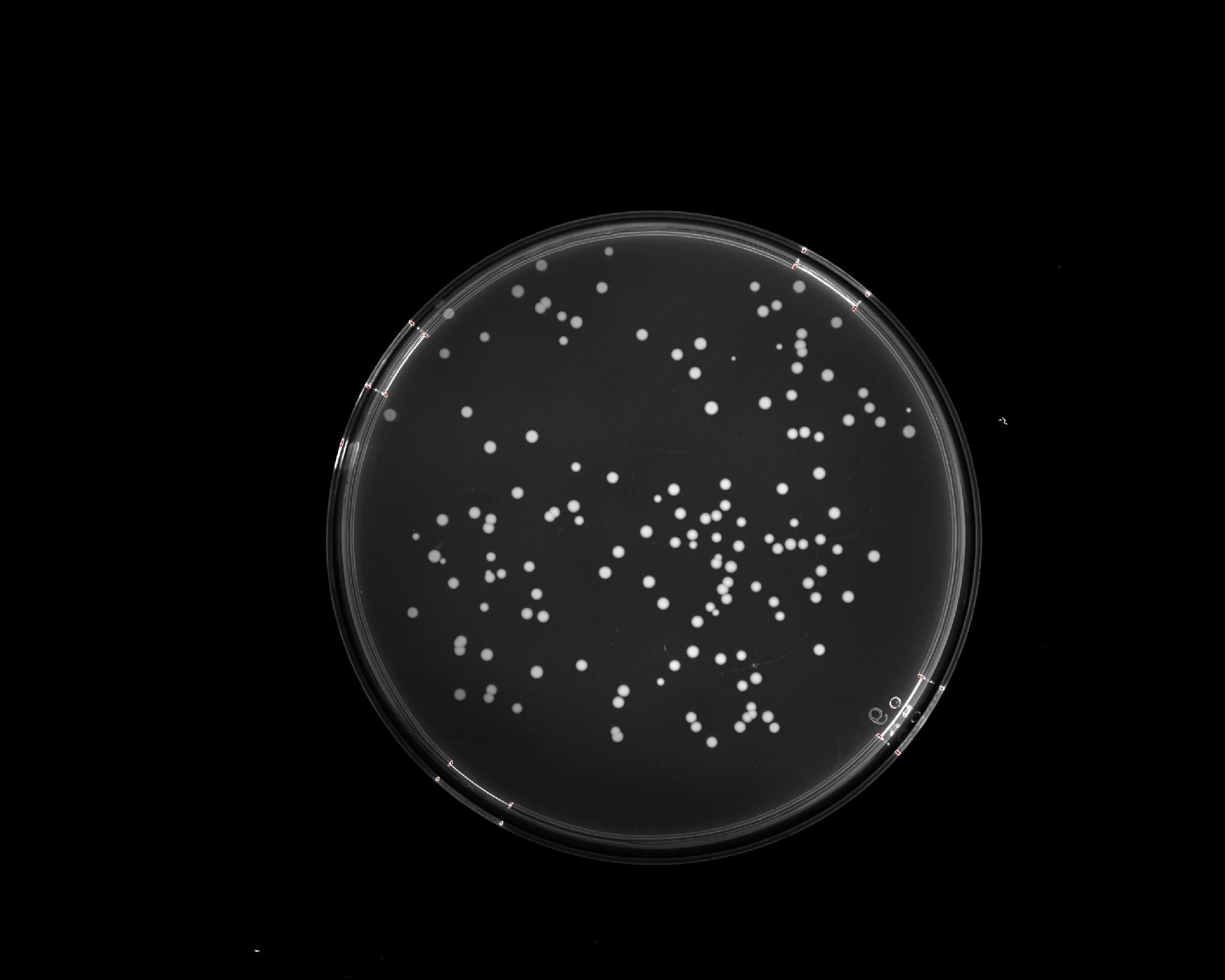

Supplement: Supplementary file 3 [file DataSheet1.ZIP › Antibacterial activity/Agar reculture observation/MRSA/M 40-100(SYBR Gold).jpg]

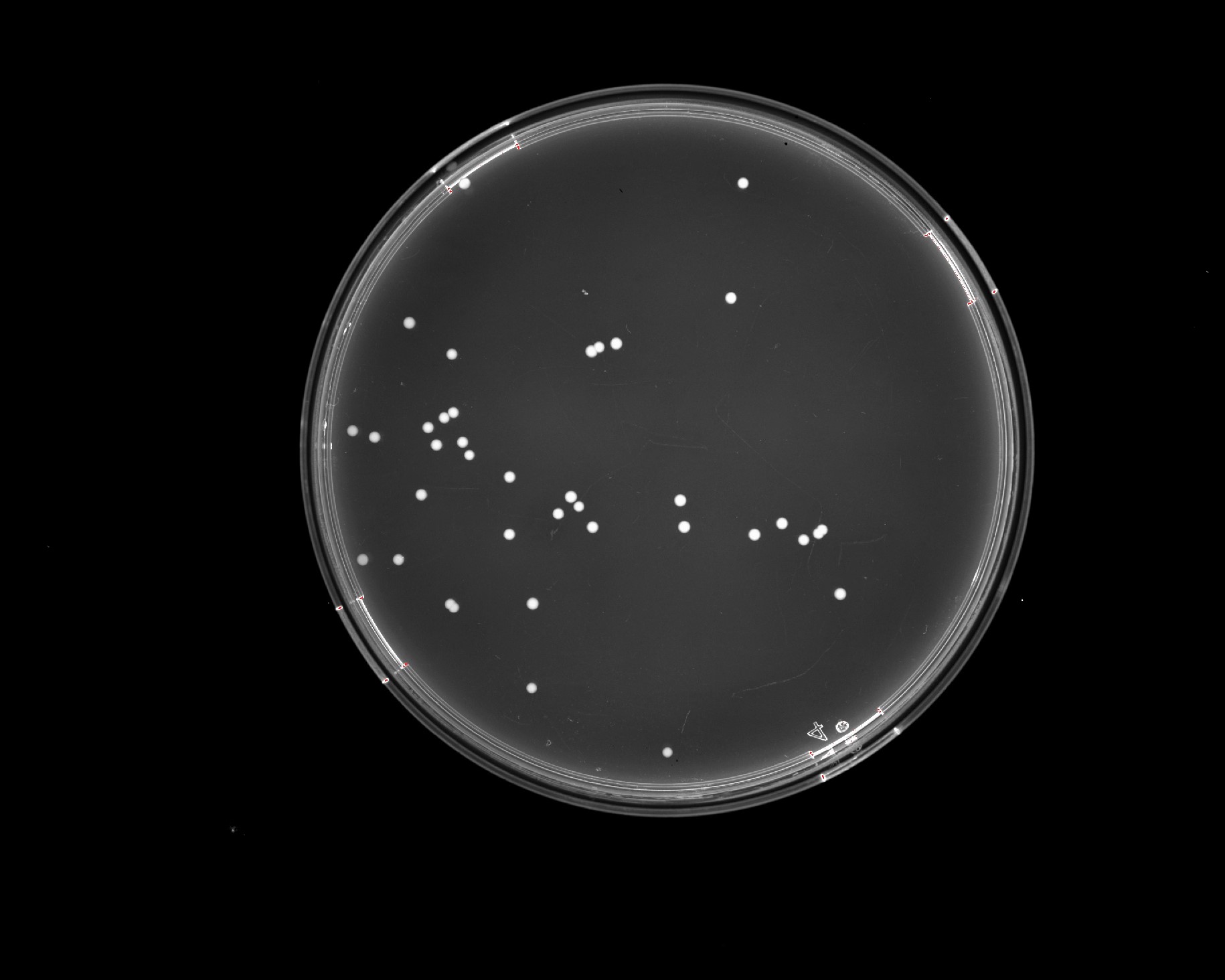

Supplement: Supplementary file 3 [file DataSheet1.ZIP › Antibacterial activity/Agar reculture observation/MRSA/M 40-1000(SYBR Gold).jpg]

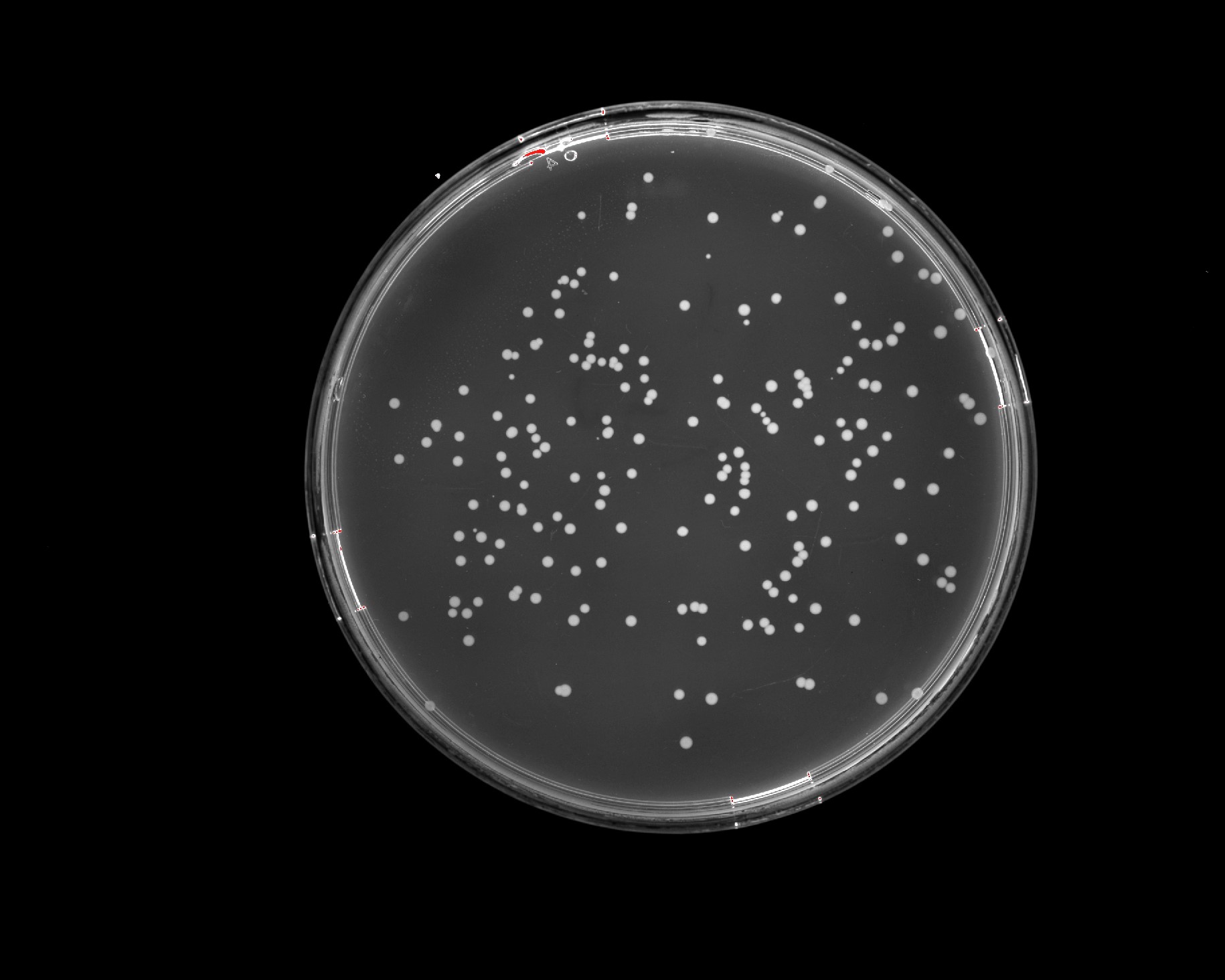

Supplement: Supplementary file 3 [file DataSheet1.ZIP › Antibacterial activity/Agar reculture observation/MRSA/M 50-10(SYBR Gold).jpg]

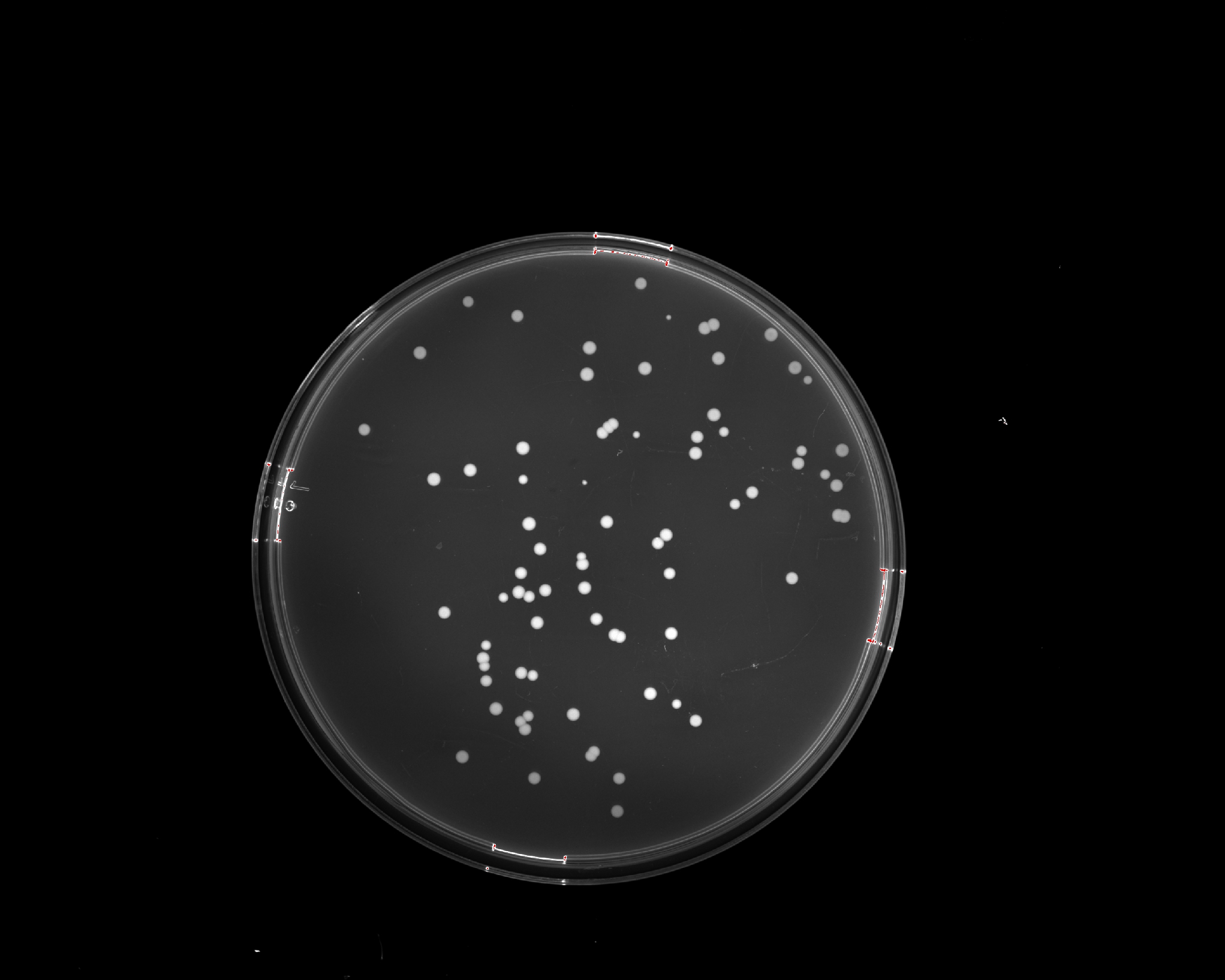

Supplement: Supplementary file 3 [file DataSheet1.ZIP › Antibacterial activity/Agar reculture observation/MRSA/M 50-100(SYBR Gold).jpg]

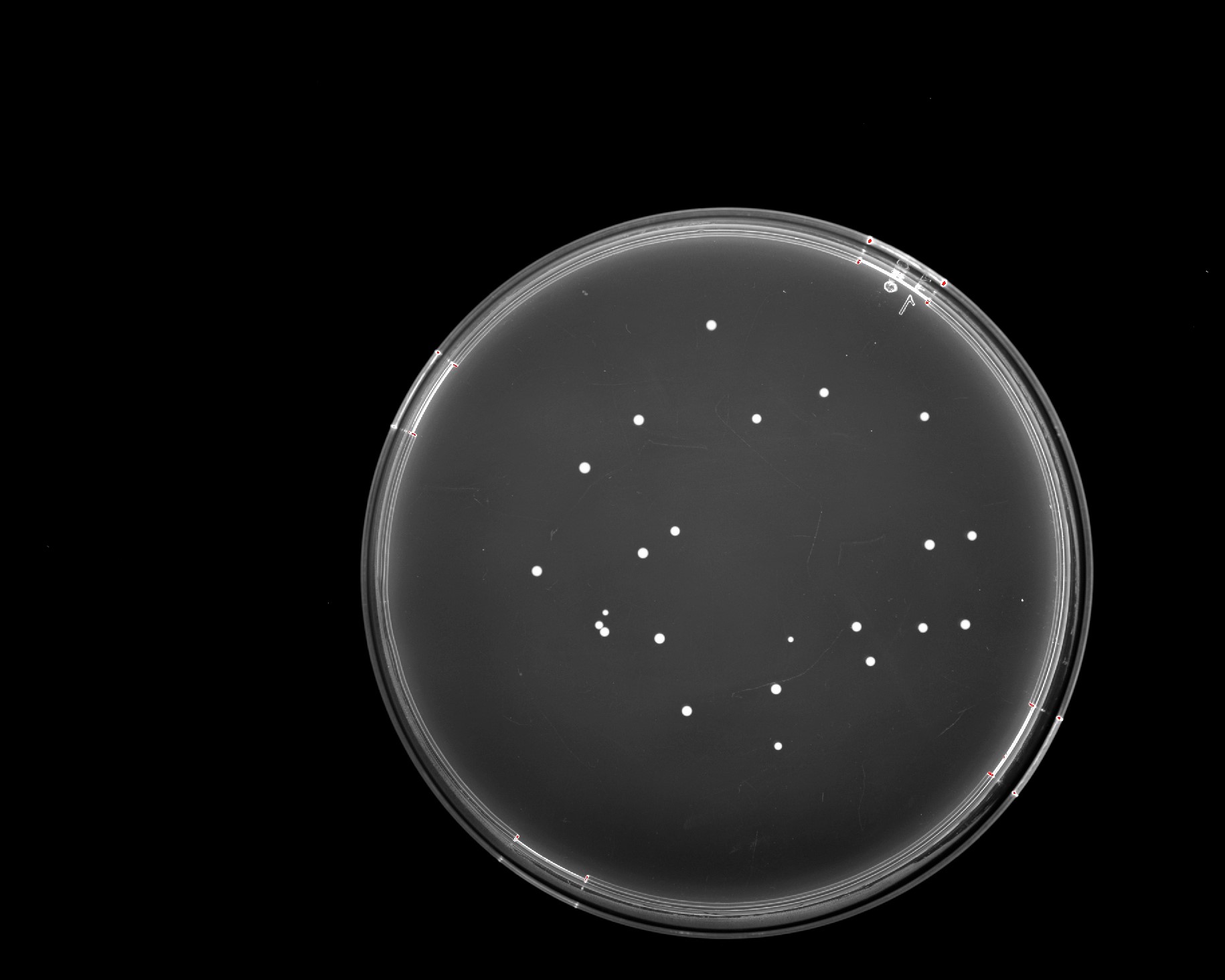

Supplement: Supplementary file 3 [file DataSheet1.ZIP › Antibacterial activity/Agar reculture observation/MRSA/M 50-1000(SYBR Gold).jpg]

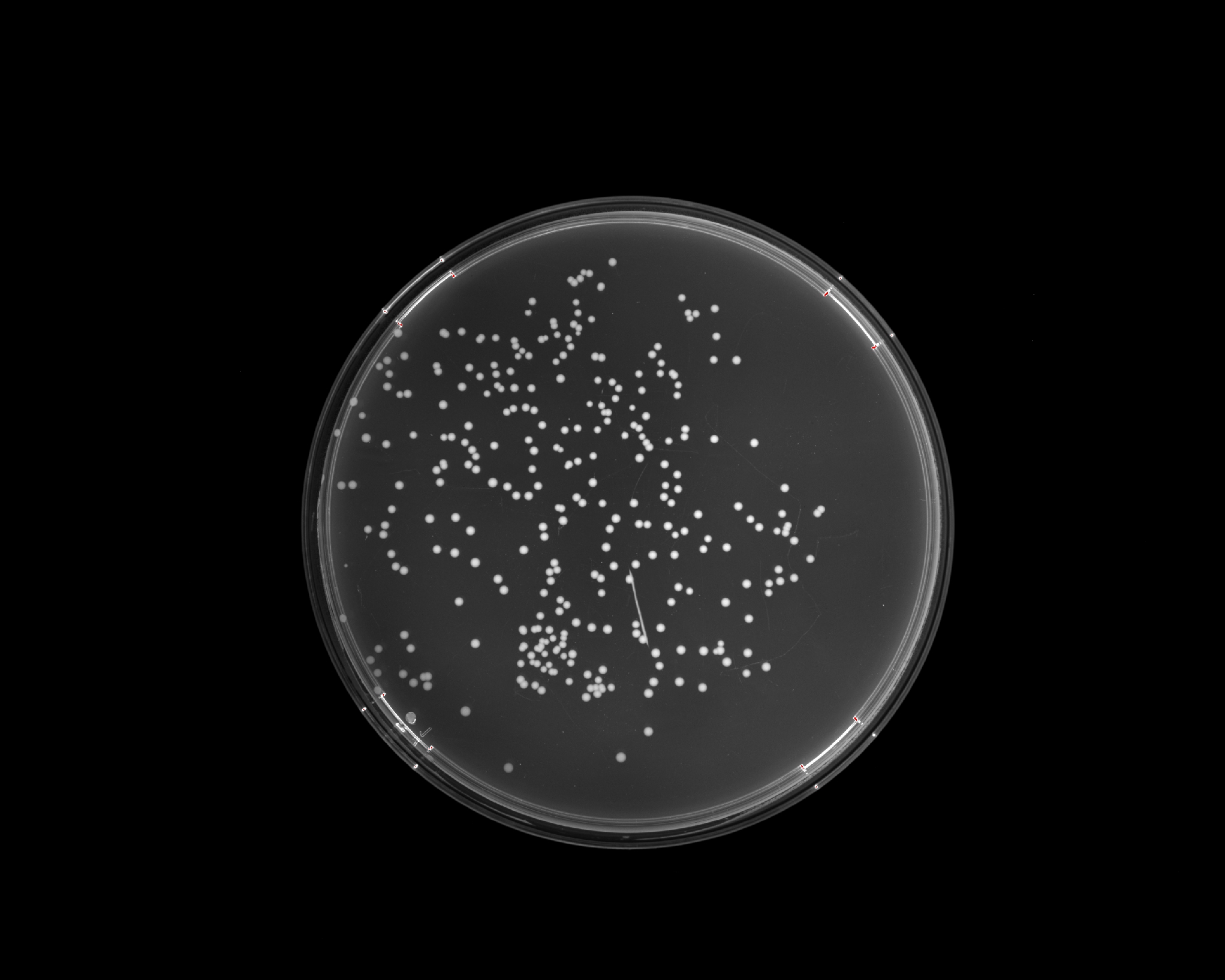

Supplement: Supplementary file 3 [file DataSheet1.ZIP › Antibacterial activity/Agar reculture observation/MRSA/M 50-10-1(SYBR Gold).jpg]

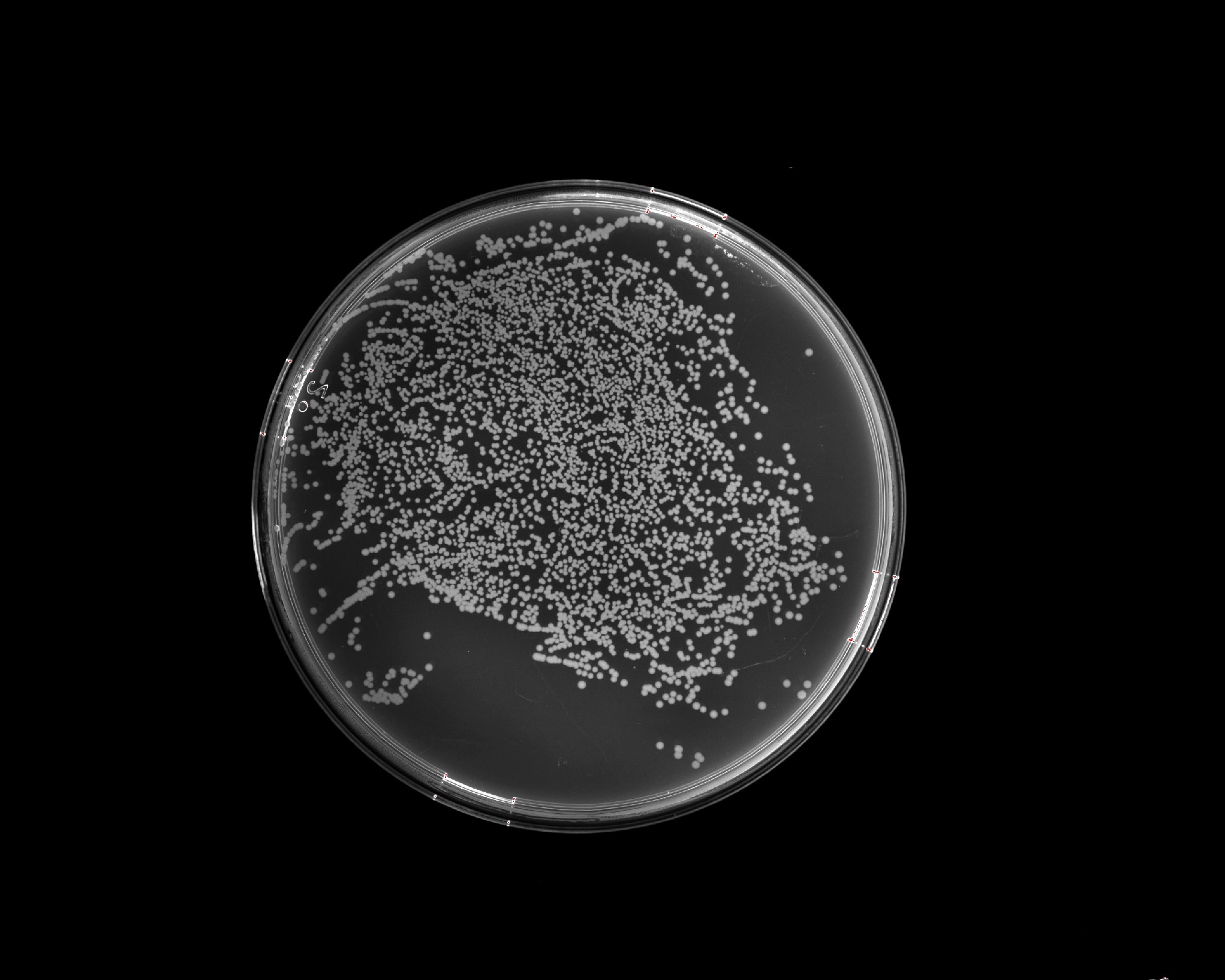

Supplement: Supplementary file 3 [file DataSheet1.ZIP › Antibacterial activity/Agar reculture observation/MRSA/M Ti -100_2(SYBR Gold).jpg]

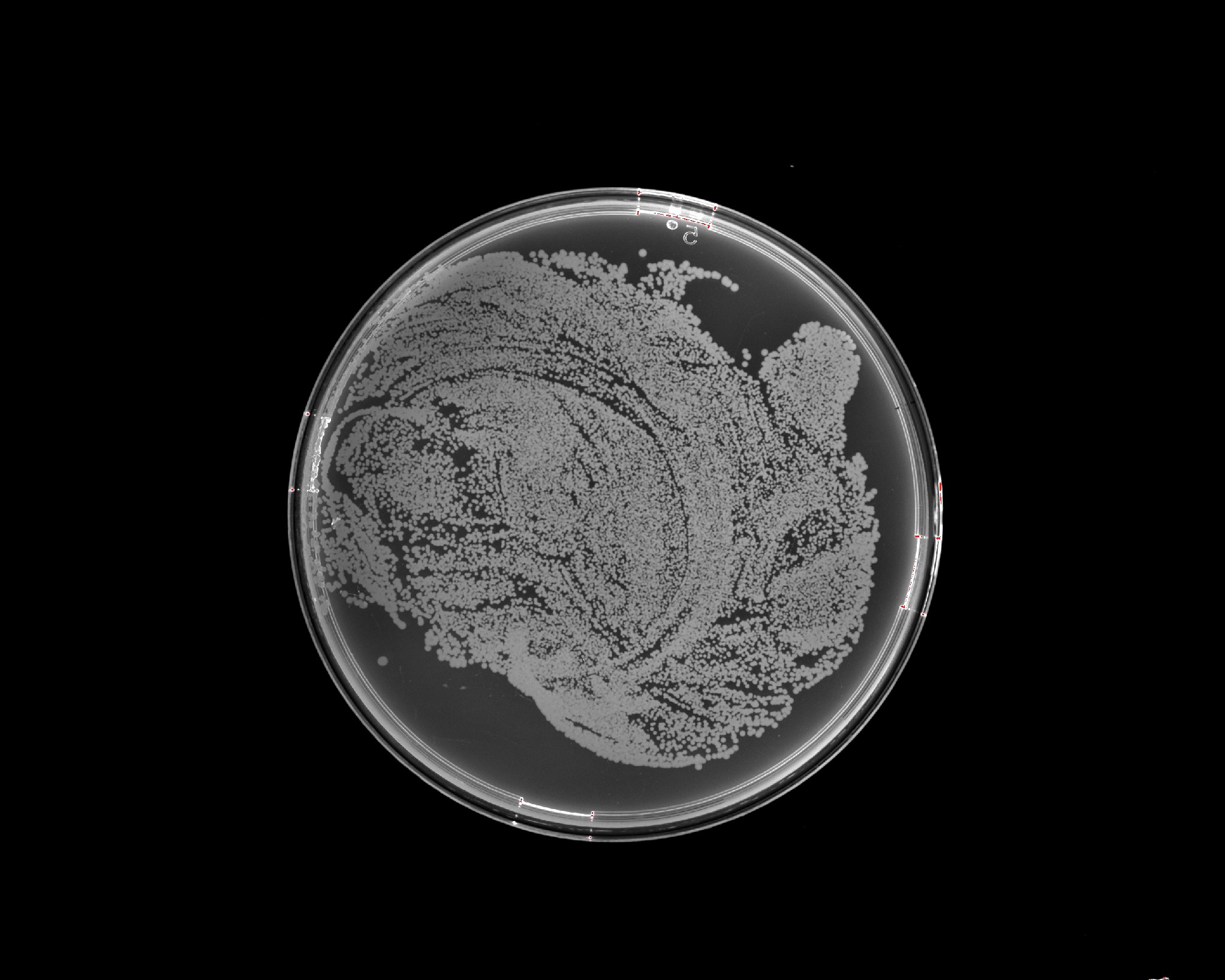

Supplement: Supplementary file 3 [file DataSheet1.ZIP › Antibacterial activity/Agar reculture observation/MRSA/M Ti-10_2(SYBR Gold).jpg]

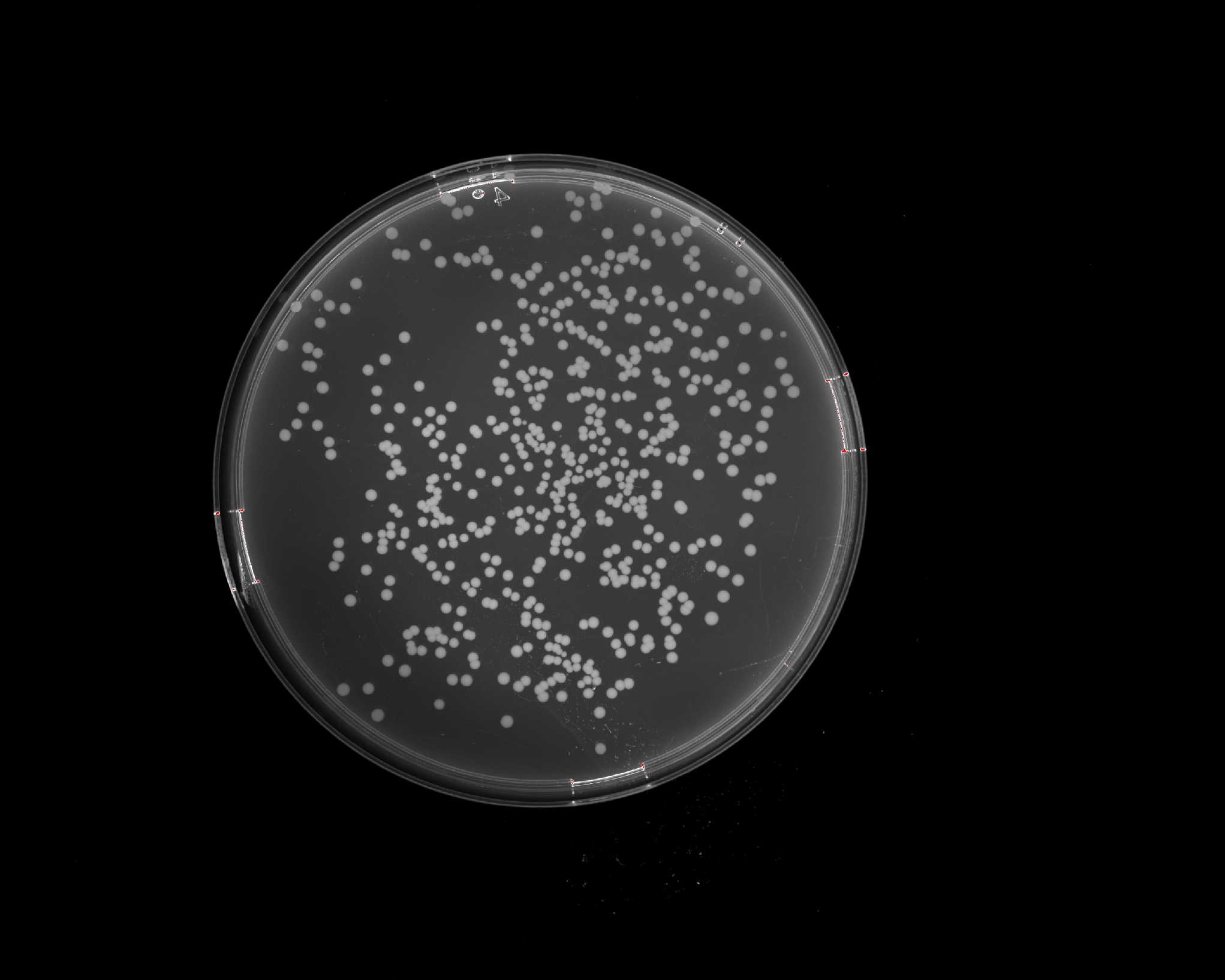

Supplement: Supplementary file 3 [file DataSheet1.ZIP › Antibacterial activity/Agar reculture observation/MRSA/M Ti-1000(SYBR Gold).jpg]

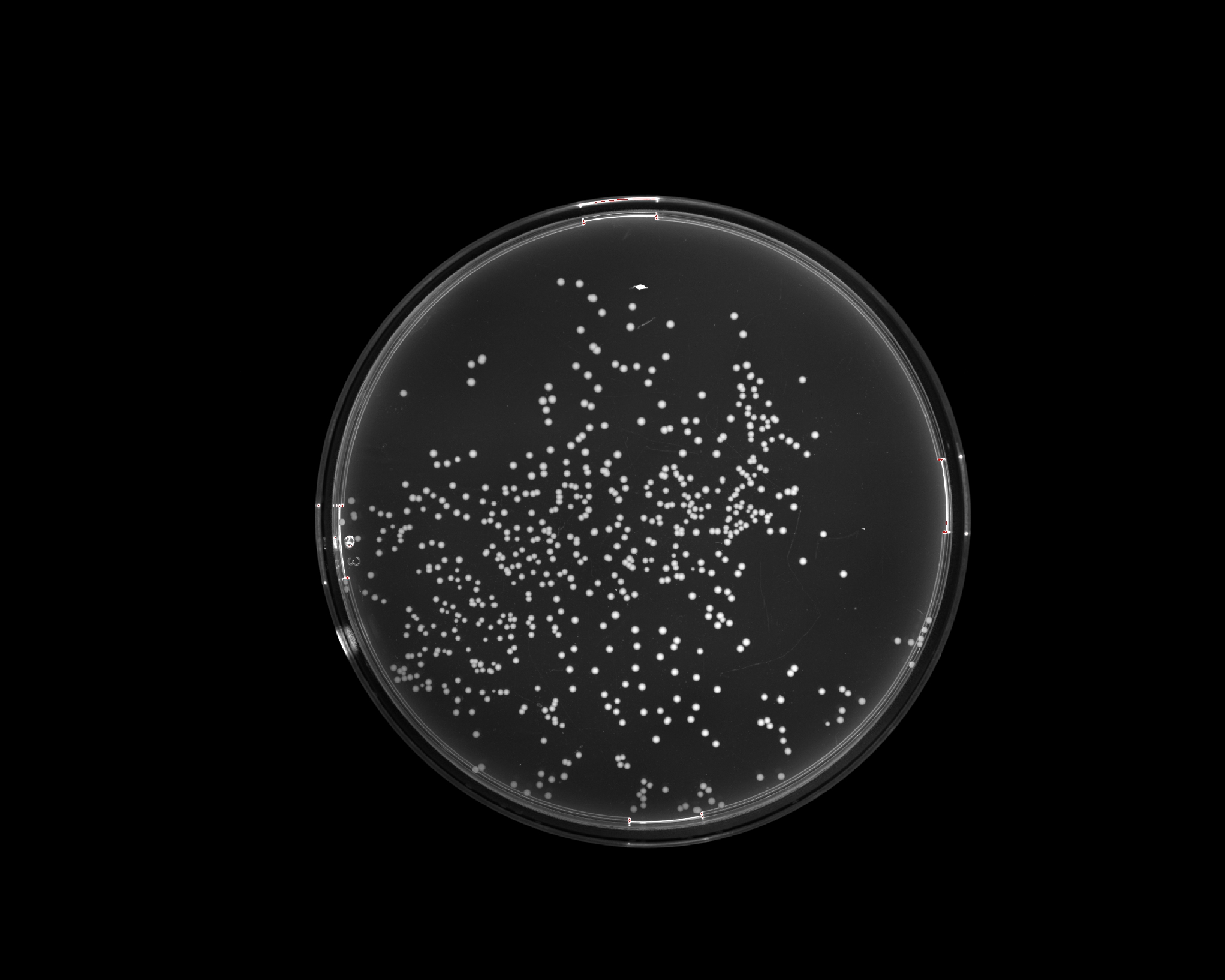

Supplement: Supplementary file 3 [file DataSheet1.ZIP › Antibacterial activity/Agar reculture observation/MRSA/M40-10_1(SYBR Gold).jpg]

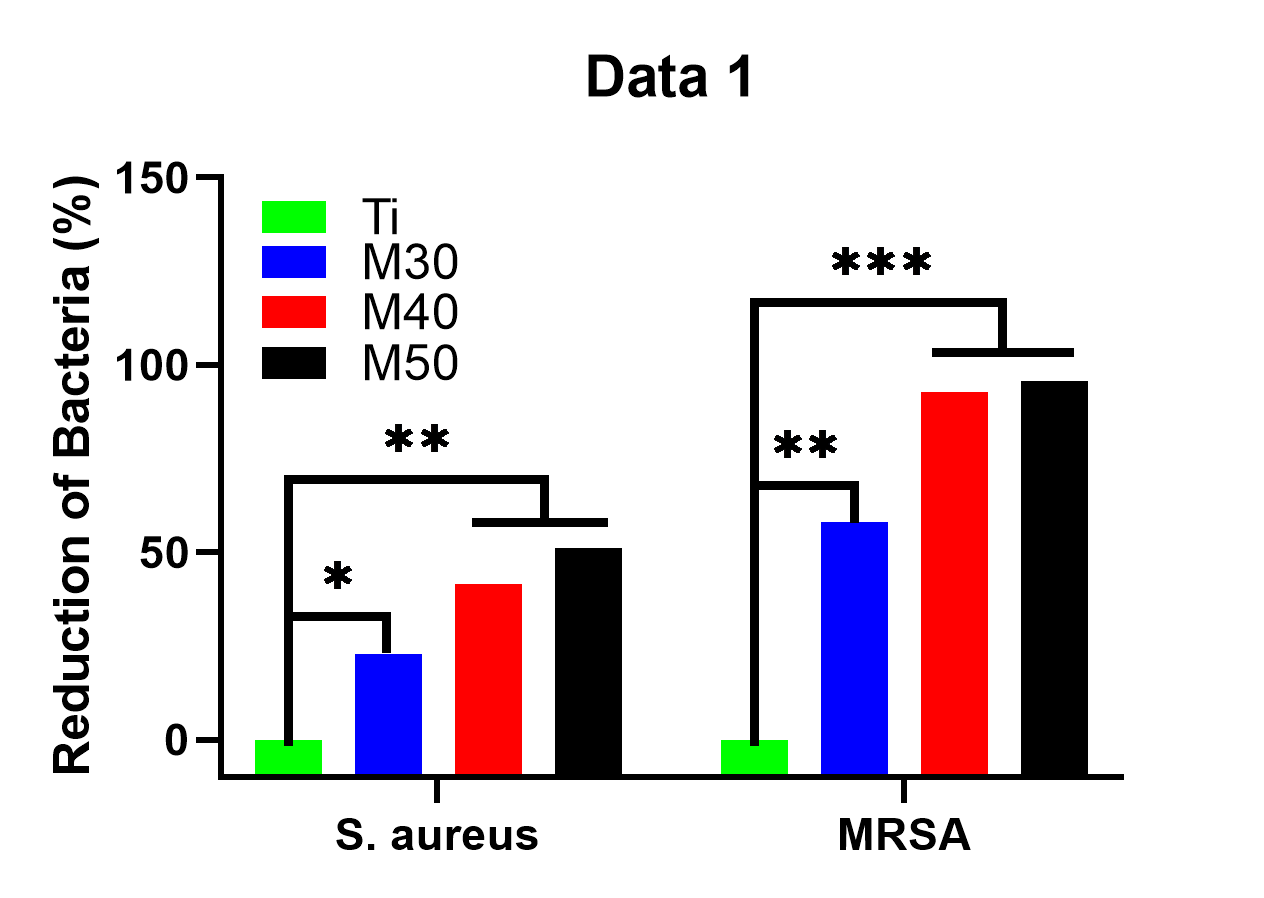

Supplement: Supplementary file 3 [file DataSheet1.ZIP › Antibacterial activity/Agar reculture observation/reduction percentages of bacteria colonies.tif]

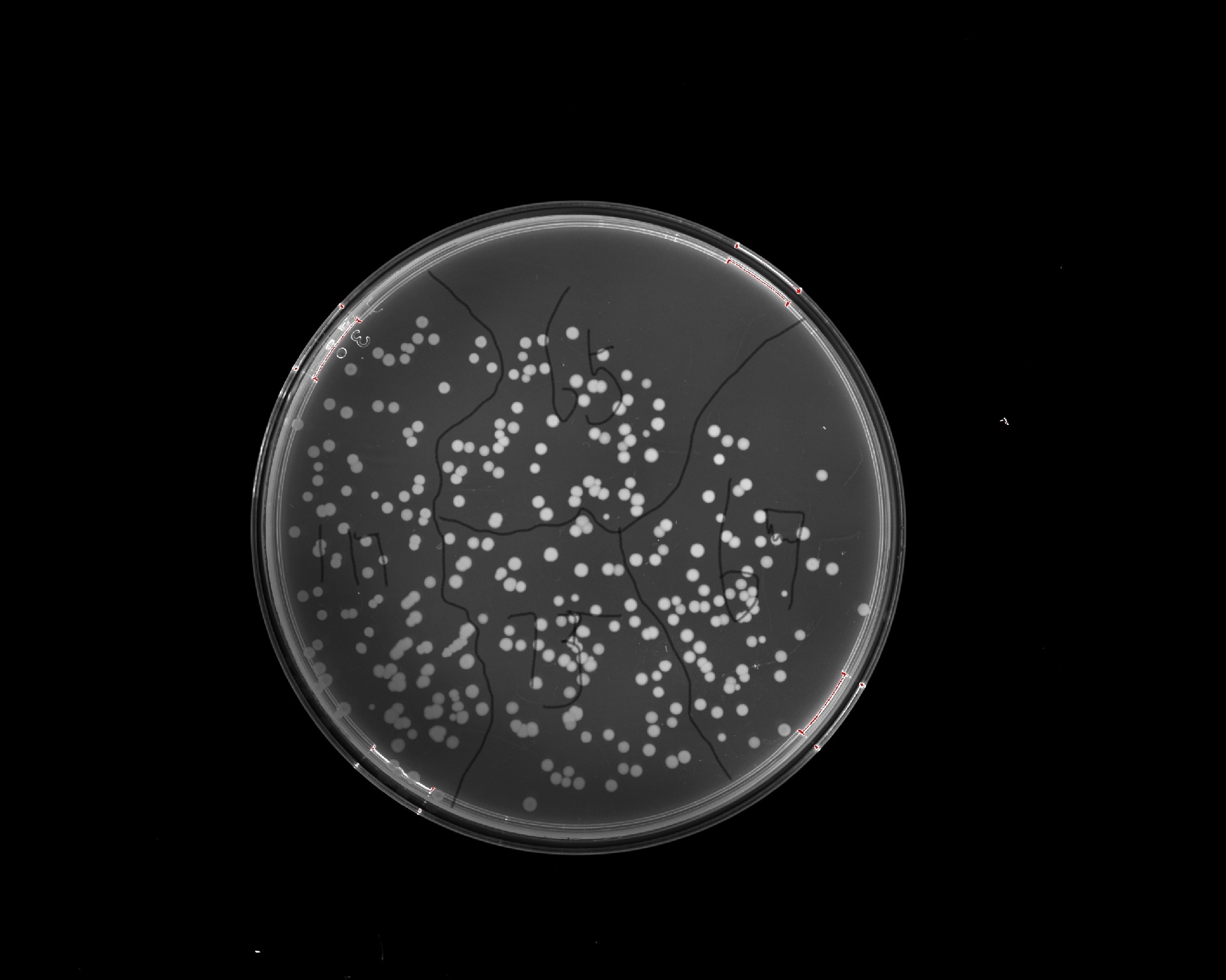

Supplement: Supplementary file 3 [file DataSheet1.ZIP › Antibacterial activity/Agar reculture observation/S. aureus/Counting/InkedInkedS Ti-1000(SYBR Gold)_LI.jpg]

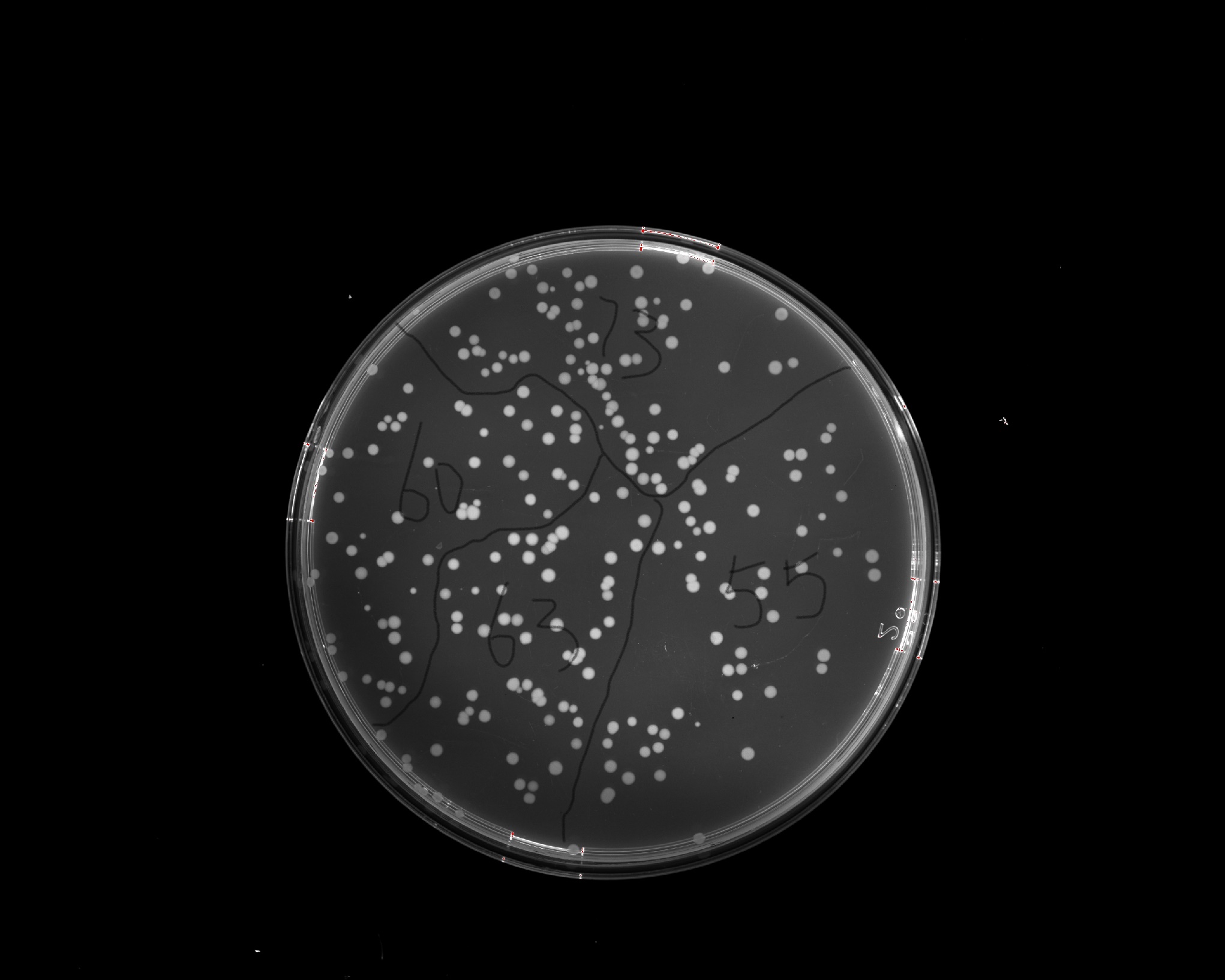

Supplement: Supplementary file 3 [file DataSheet1.ZIP › Antibacterial activity/Agar reculture observation/S. aureus/Counting/InkedS 30-1000(SYBR Gold)_LI.jpg]

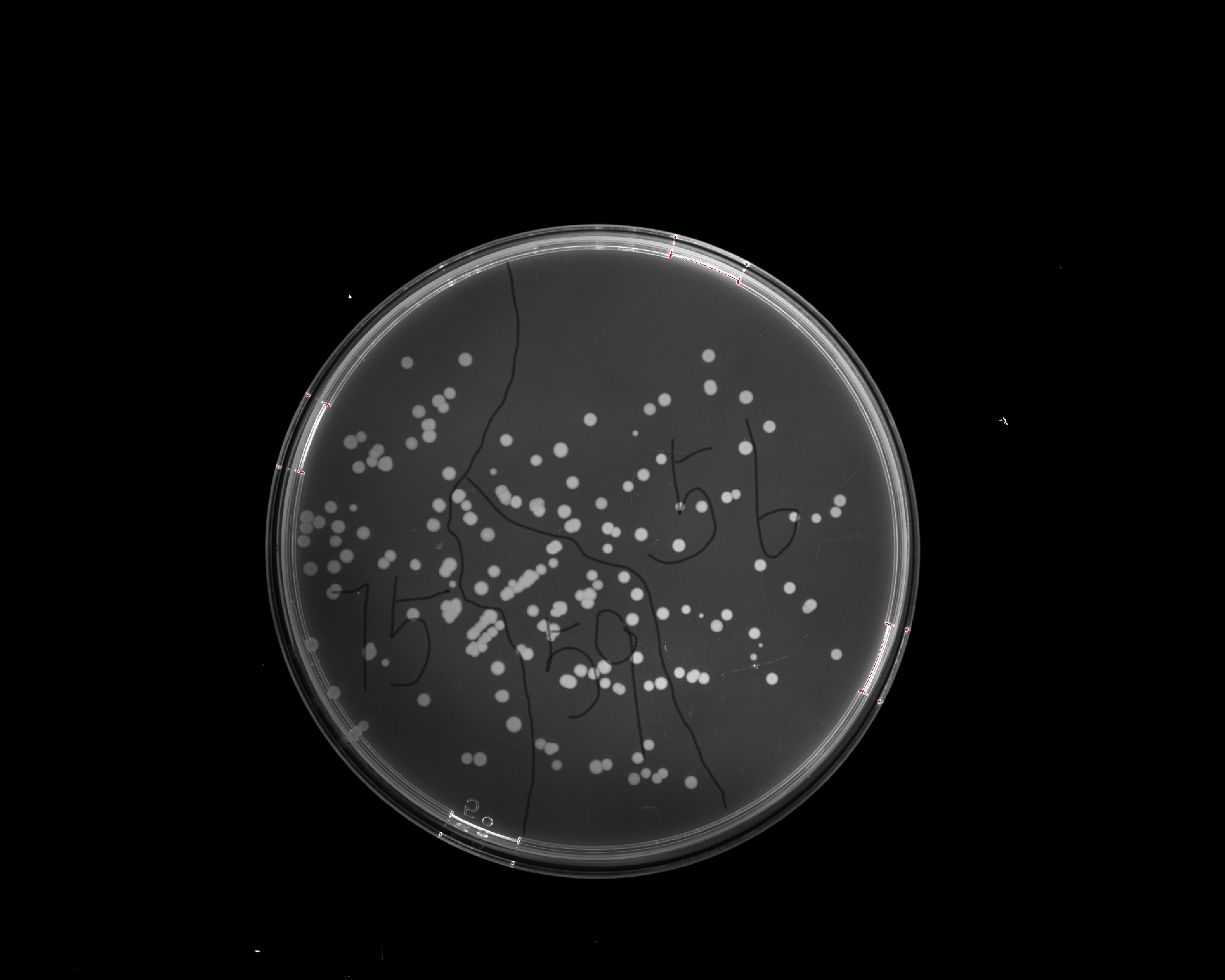

Supplement: Supplementary file 3 [file DataSheet1.ZIP › Antibacterial activity/Agar reculture observation/S. aureus/Counting/InkedS 40-1000(SYBR Gold)_LI.jpg]

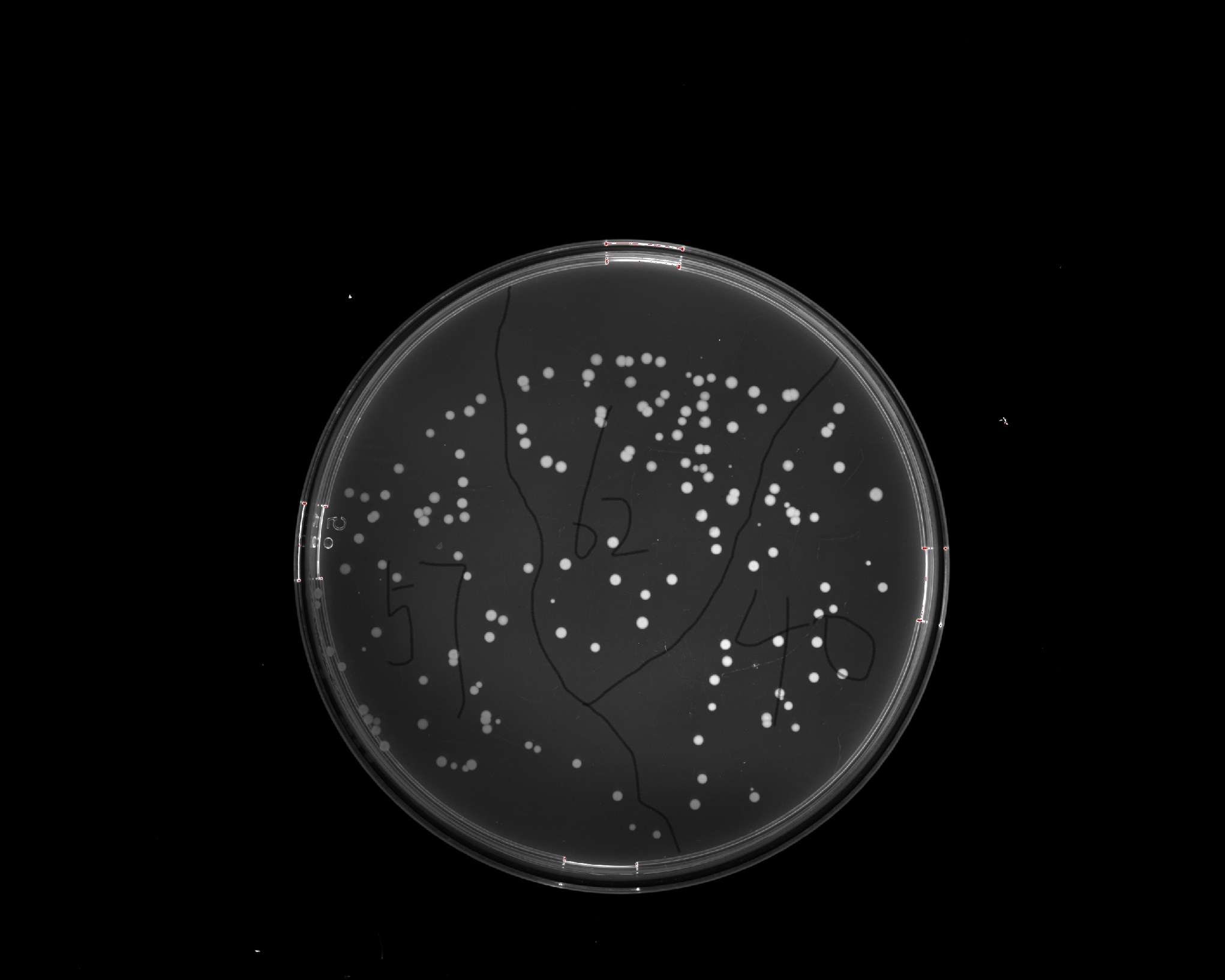

Supplement: Supplementary file 3 [file DataSheet1.ZIP › Antibacterial activity/Agar reculture observation/S. aureus/Counting/InkedS 50-1000(SYBR Gold)_LI.jpg]

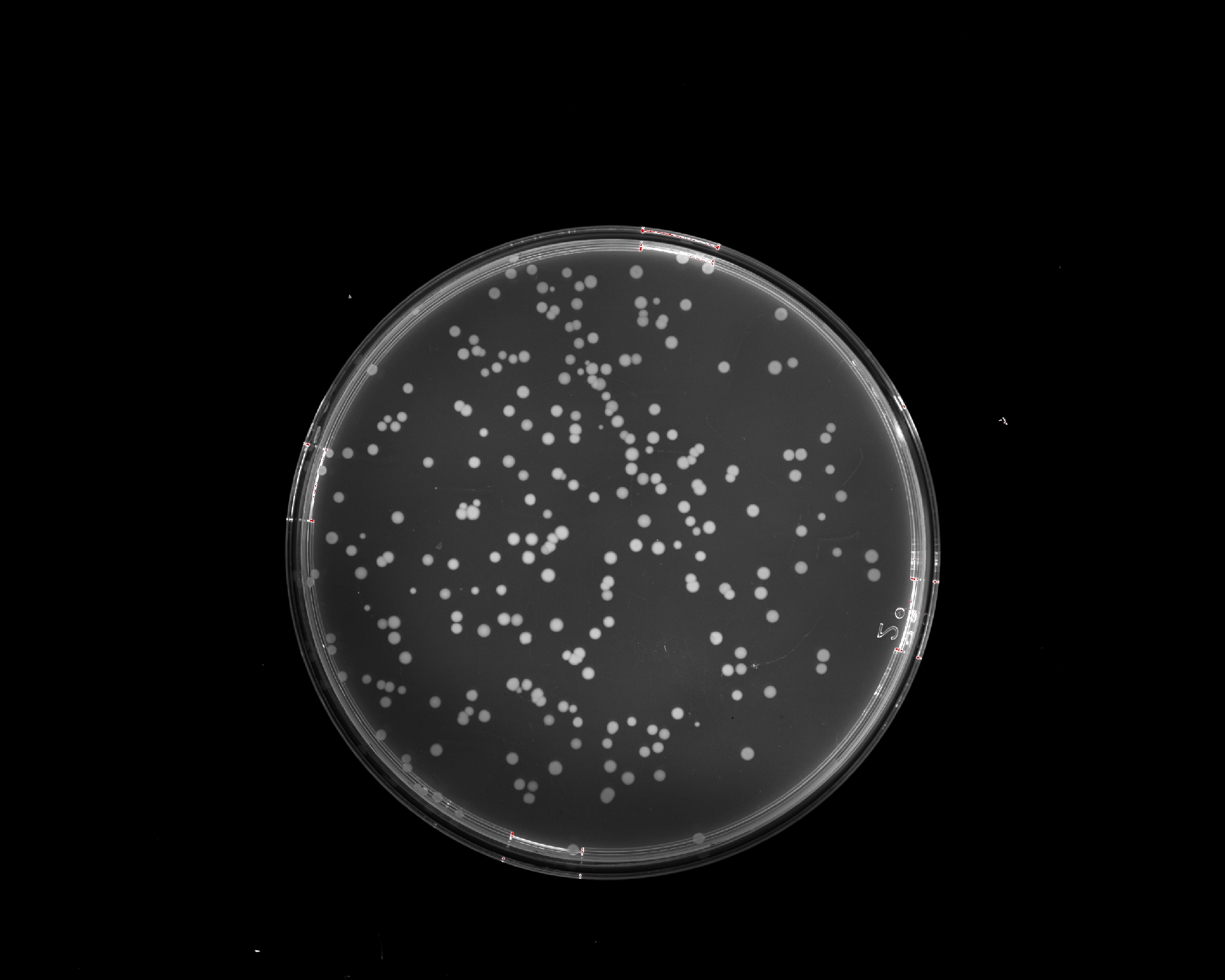

Supplement: Supplementary file 3 [file DataSheet1.ZIP › Antibacterial activity/Agar reculture observation/S. aureus/S 30-1000(SYBR Gold).jpg]

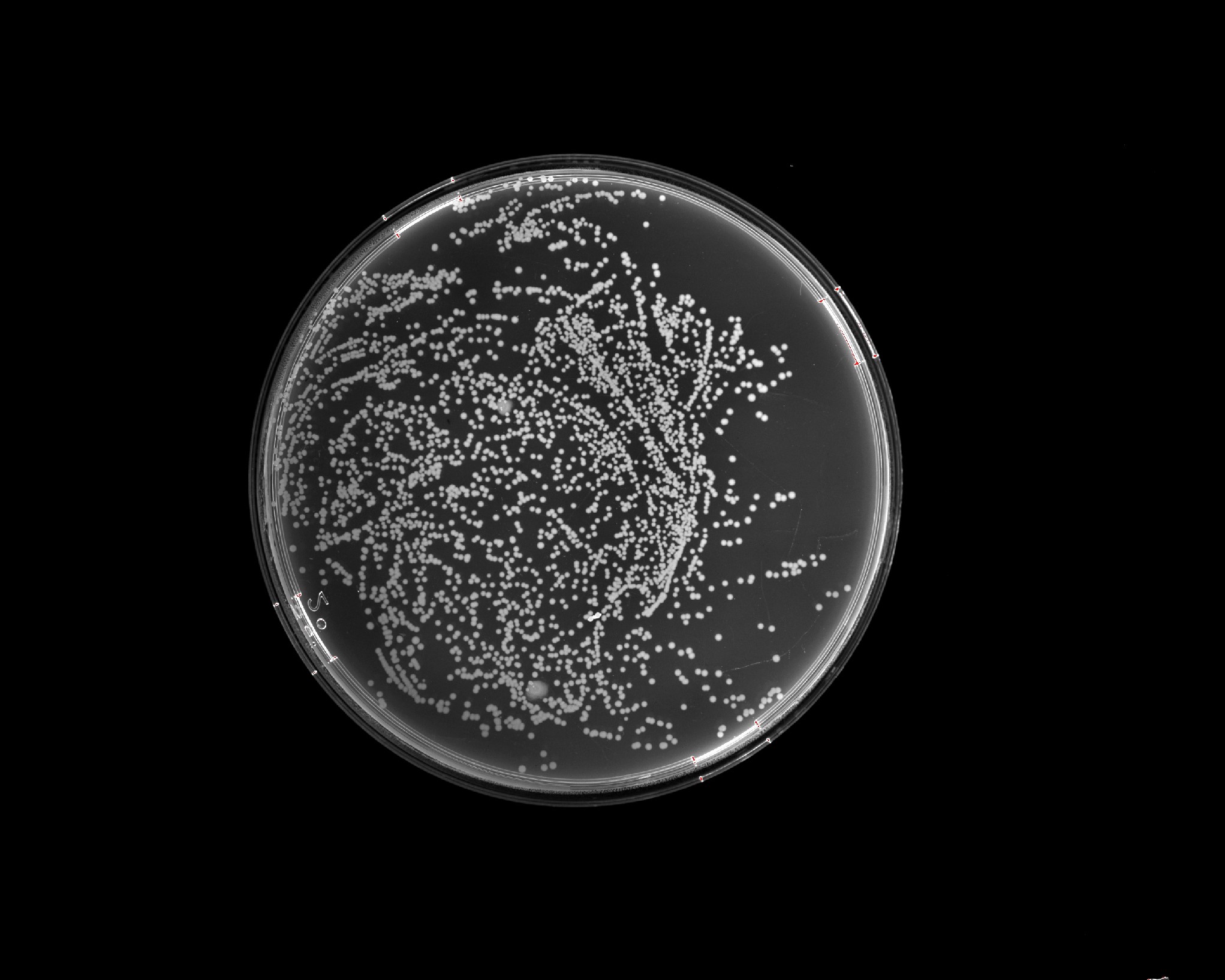

Supplement: Supplementary file 3 [file DataSheet1.ZIP › Antibacterial activity/Agar reculture observation/S. aureus/S 30-100-1 (SYBR Gold).jpg]

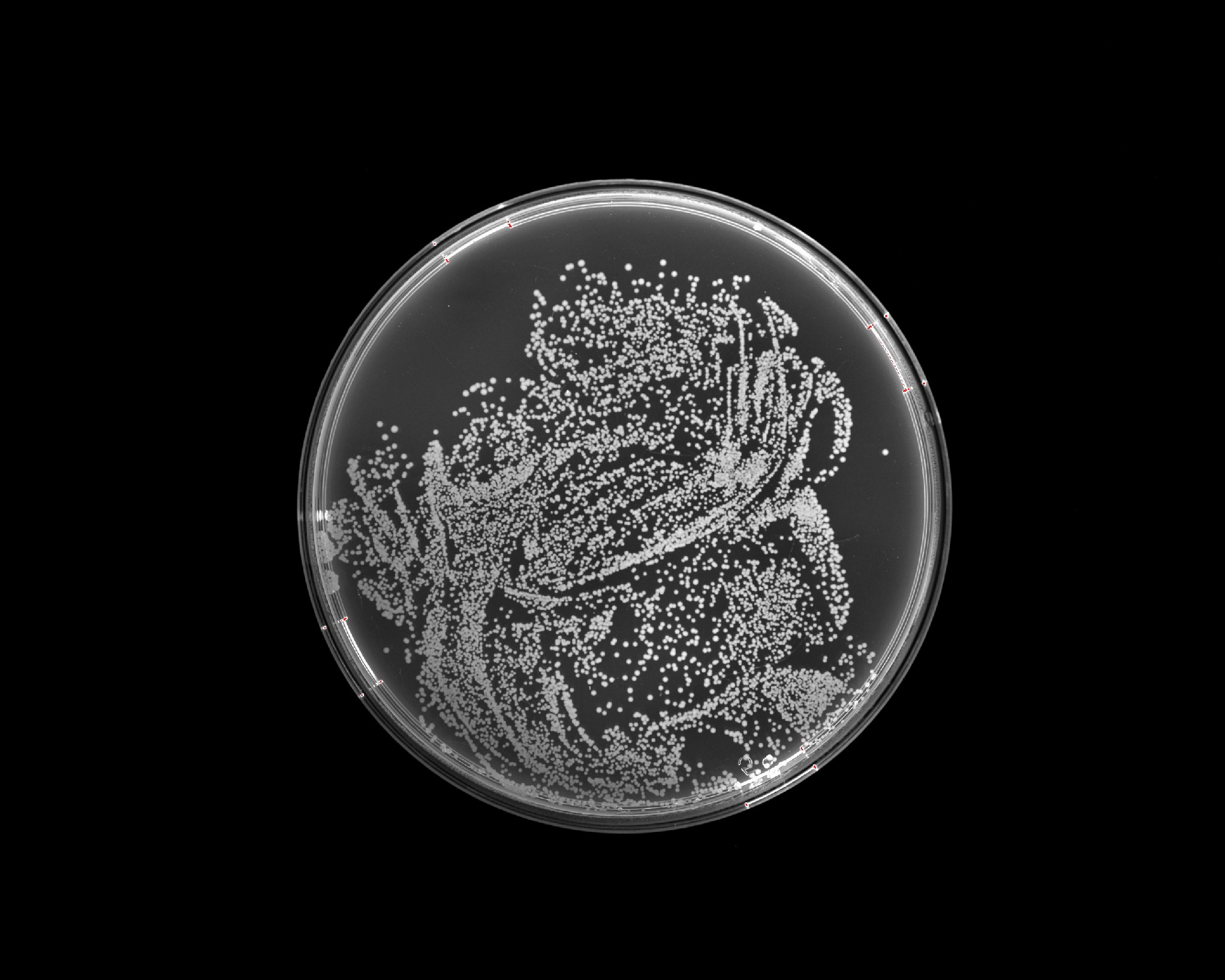

Supplement: Supplementary file 3 [file DataSheet1.ZIP › Antibacterial activity/Agar reculture observation/S. aureus/S 30-10-1 (SYBR Gold).jpg]

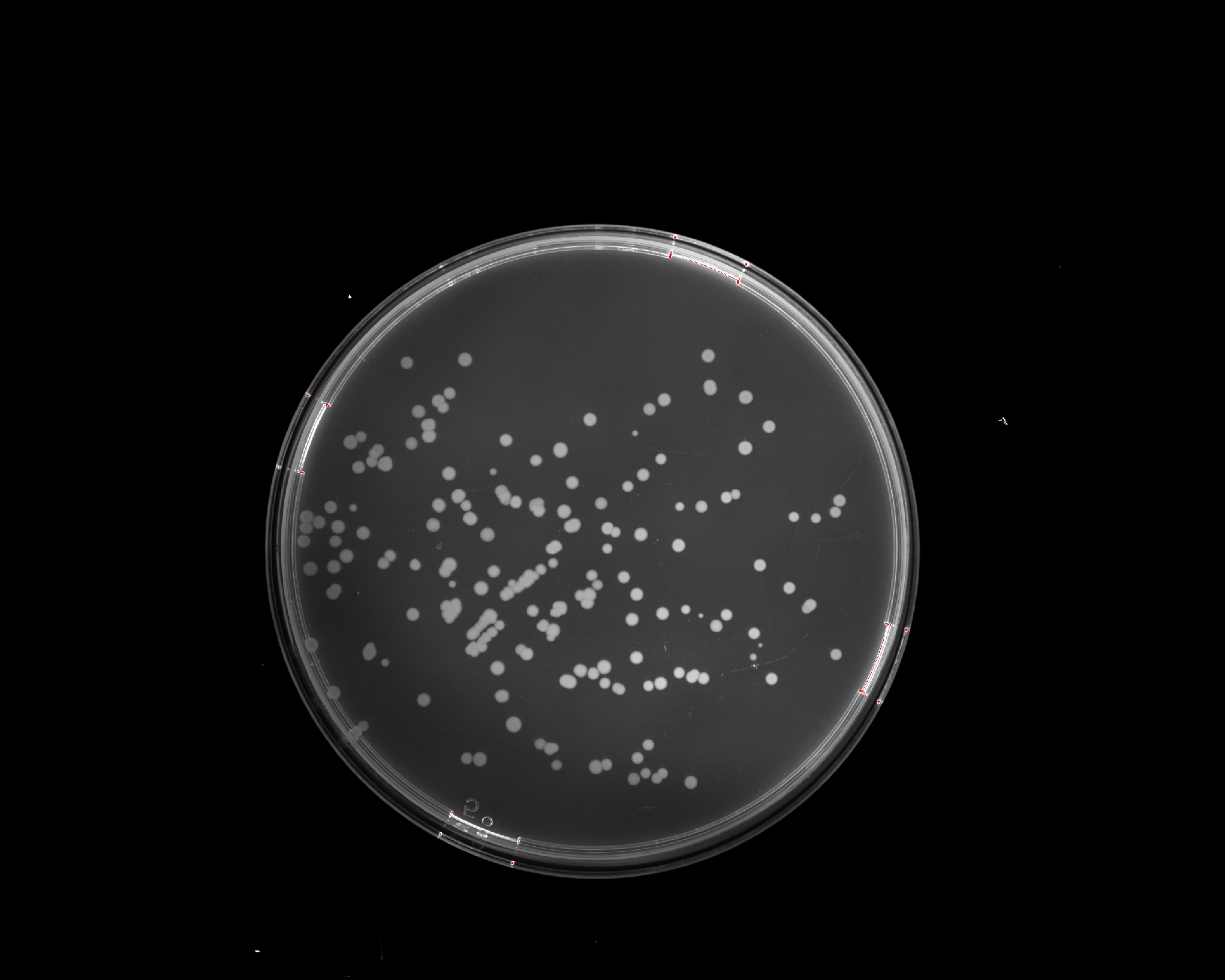

Supplement: Supplementary file 3 [file DataSheet1.ZIP › Antibacterial activity/Agar reculture observation/S. aureus/S 40-1000(SYBR Gold).jpg]

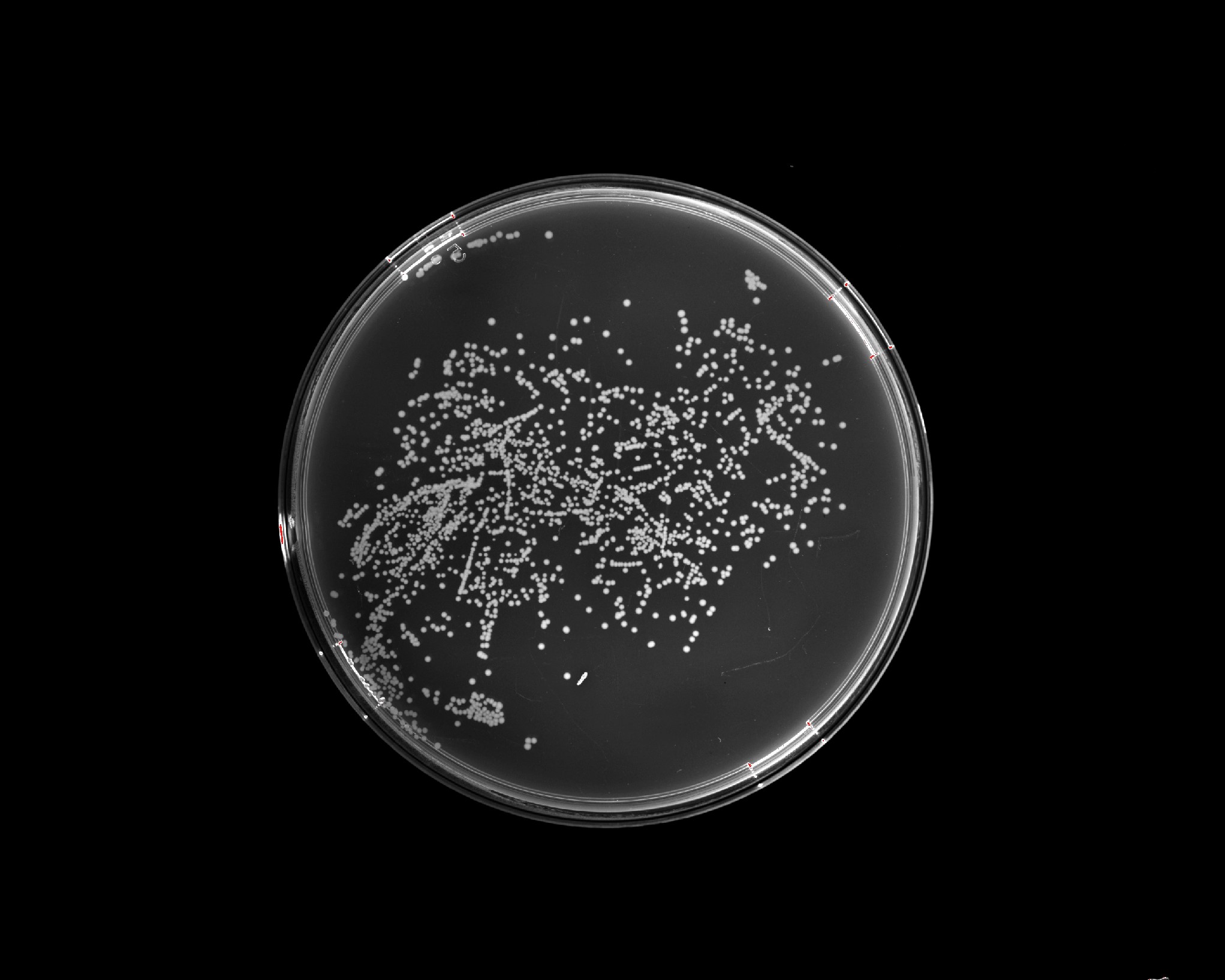

Supplement: Supplementary file 3 [file DataSheet1.ZIP › Antibacterial activity/Agar reculture observation/S. aureus/S 40-100-2 (SYBR Gold).jpg]

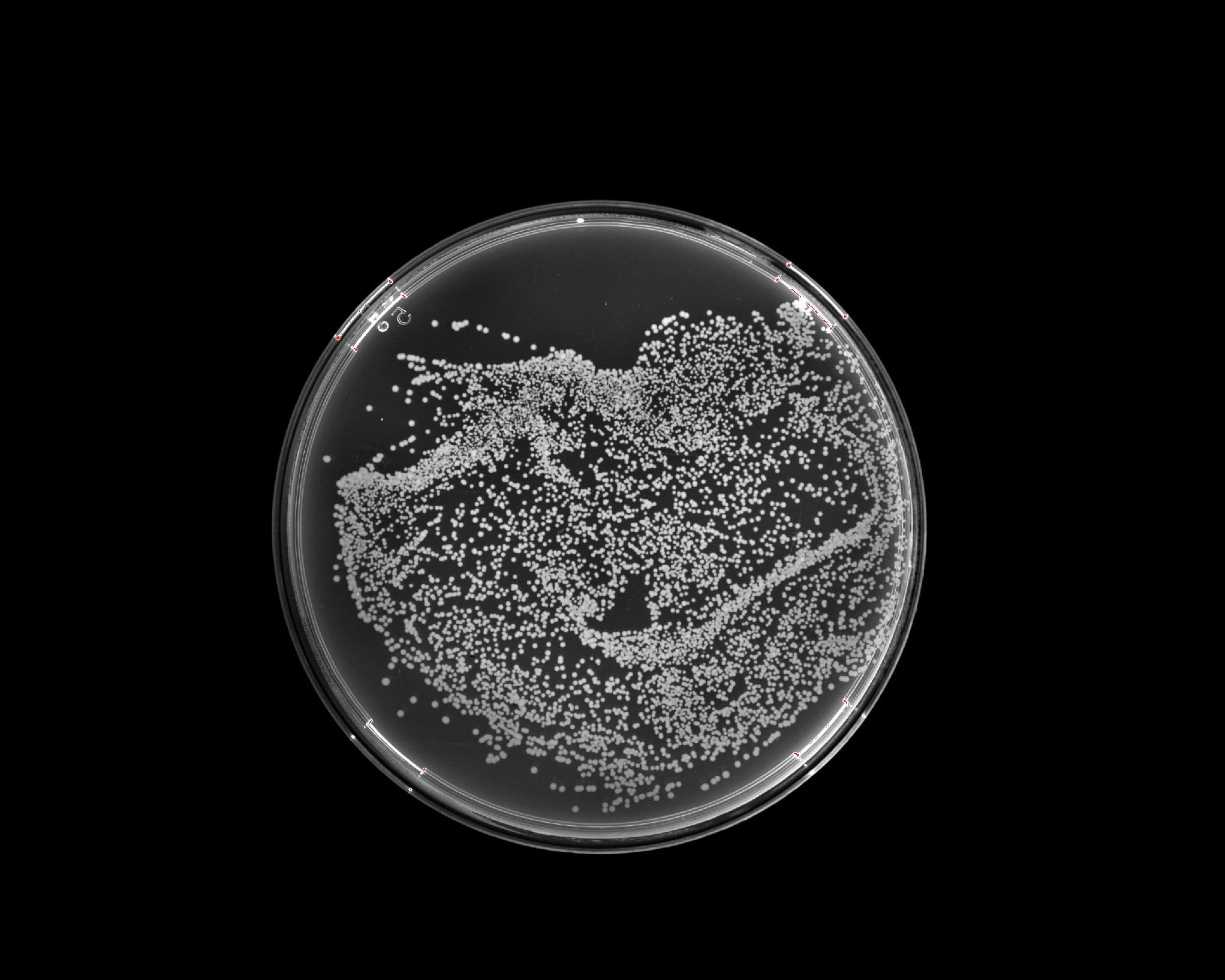

Supplement: Supplementary file 3 [file DataSheet1.ZIP › Antibacterial activity/Agar reculture observation/S. aureus/S 40-10-1 (SYBR Gold).jpg]

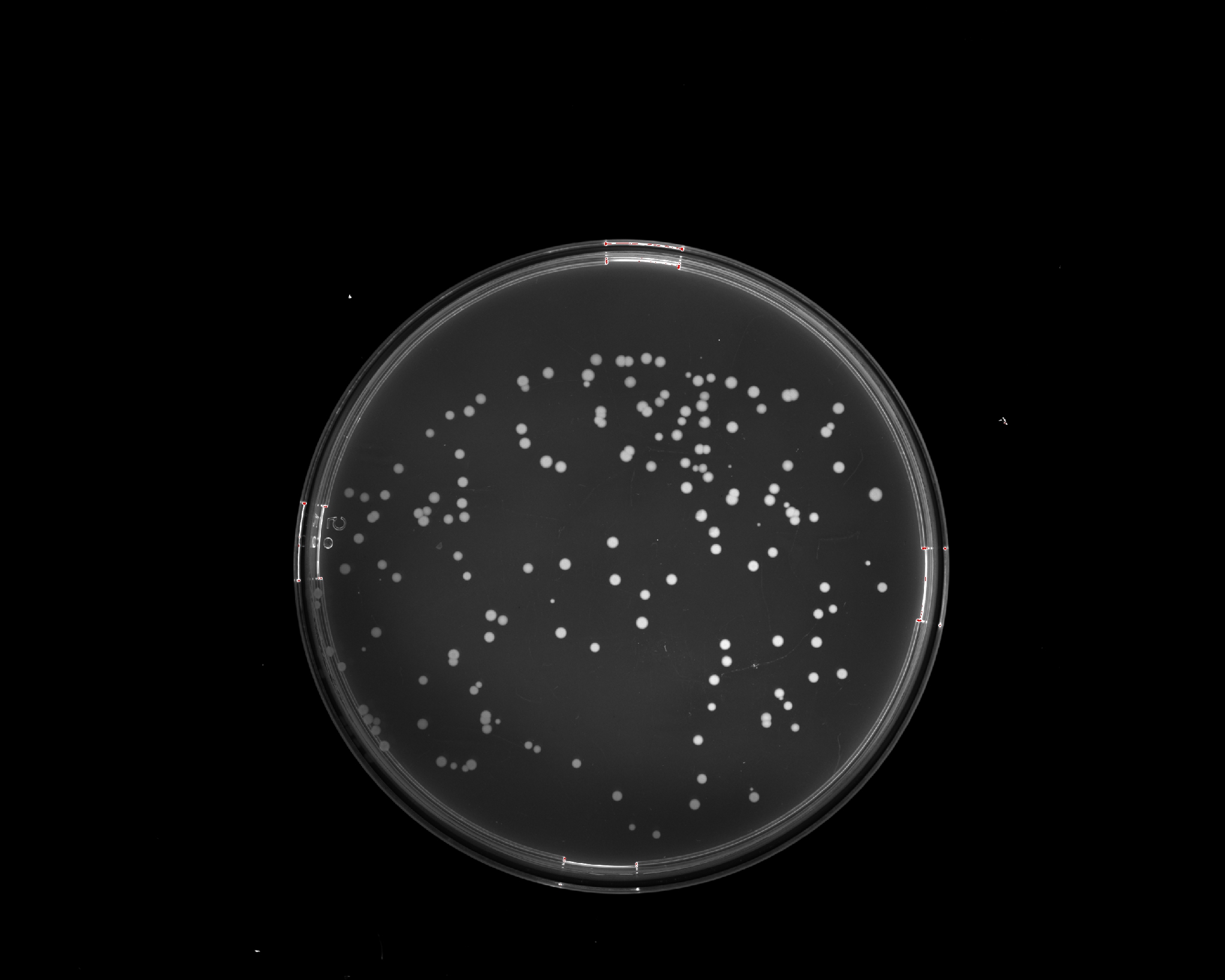

Supplement: Supplementary file 3 [file DataSheet1.ZIP › Antibacterial activity/Agar reculture observation/S. aureus/S 50-1000(SYBR Gold).jpg]

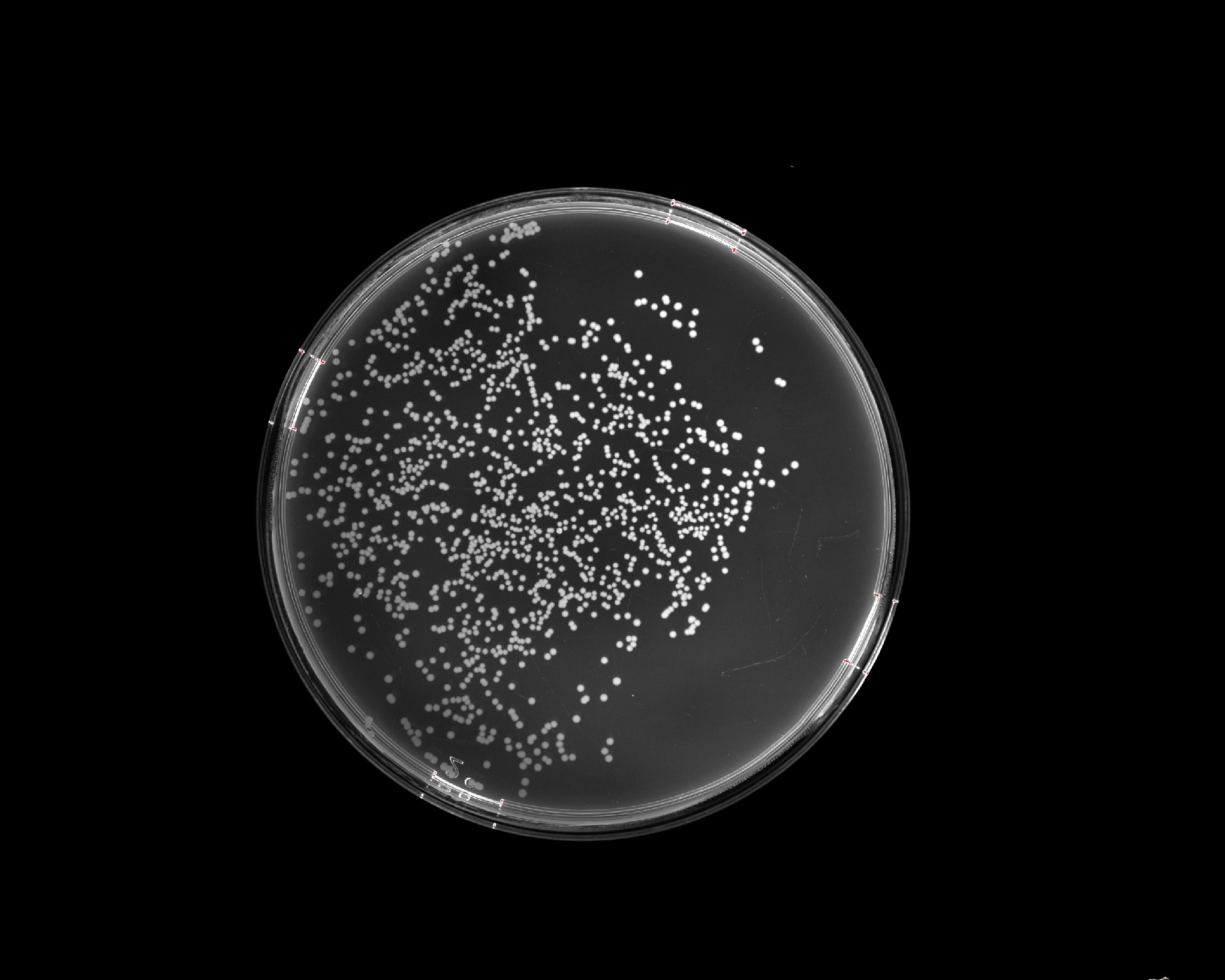

Supplement: Supplementary file 3 [file DataSheet1.ZIP › Antibacterial activity/Agar reculture observation/S. aureus/S 50-100-2(SYBR Gold).jpg]

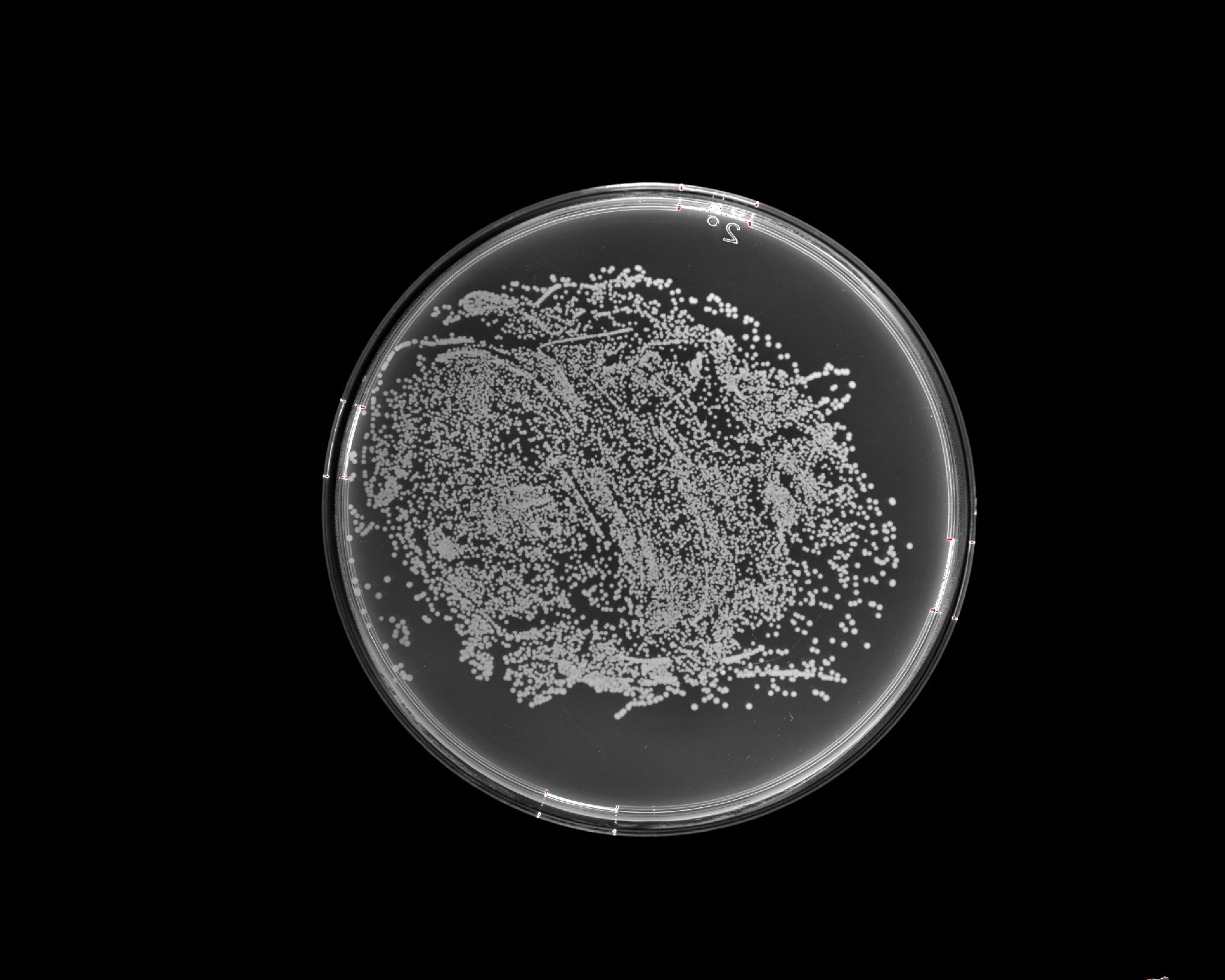

Supplement: Supplementary file 3 [file DataSheet1.ZIP › Antibacterial activity/Agar reculture observation/S. aureus/S 50-10-1(SYBR Gold).jpg]

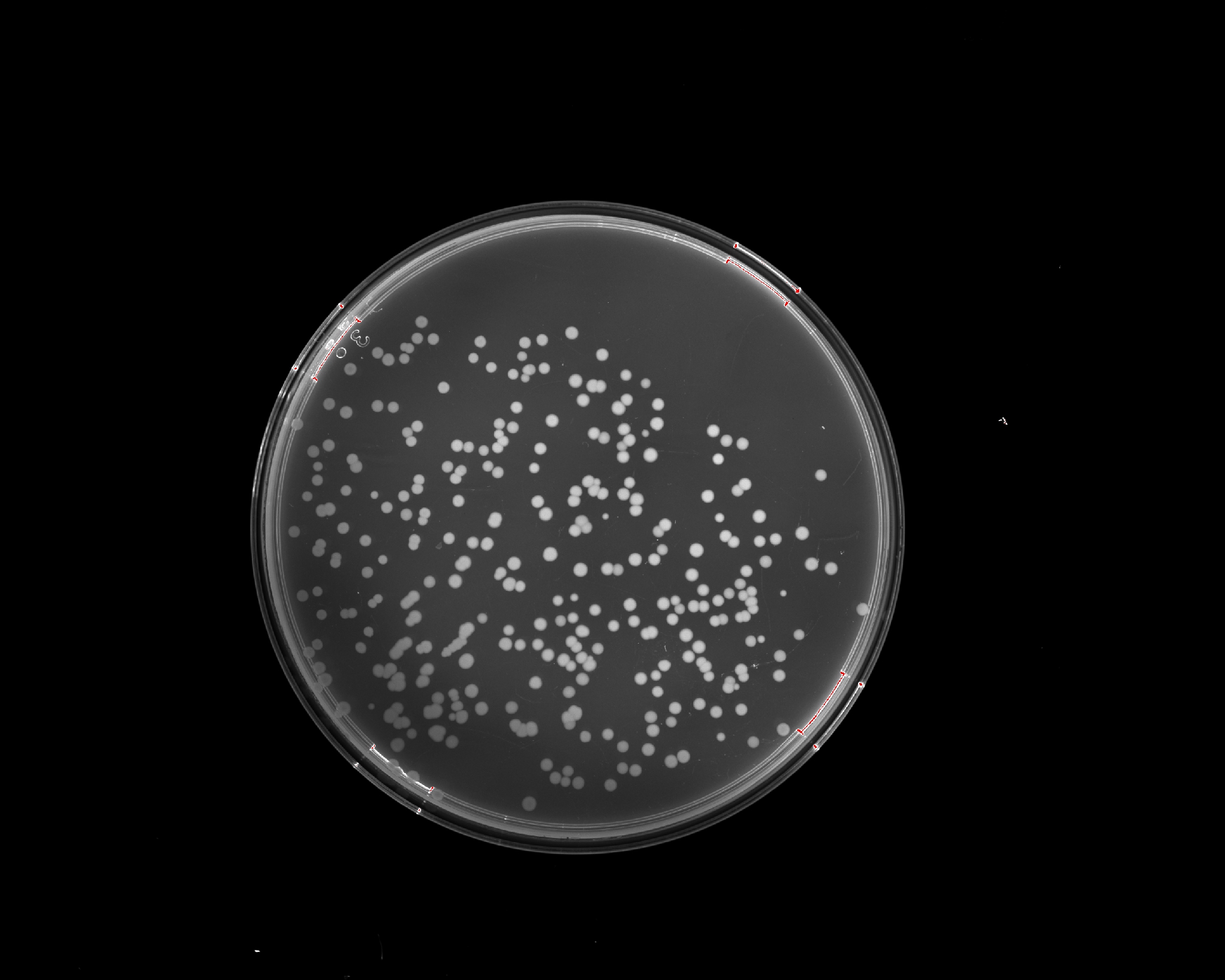

Supplement: Supplementary file 3 [file DataSheet1.ZIP › Antibacterial activity/Agar reculture observation/S. aureus/S Ti-1000(SYBR Gold).jpg]

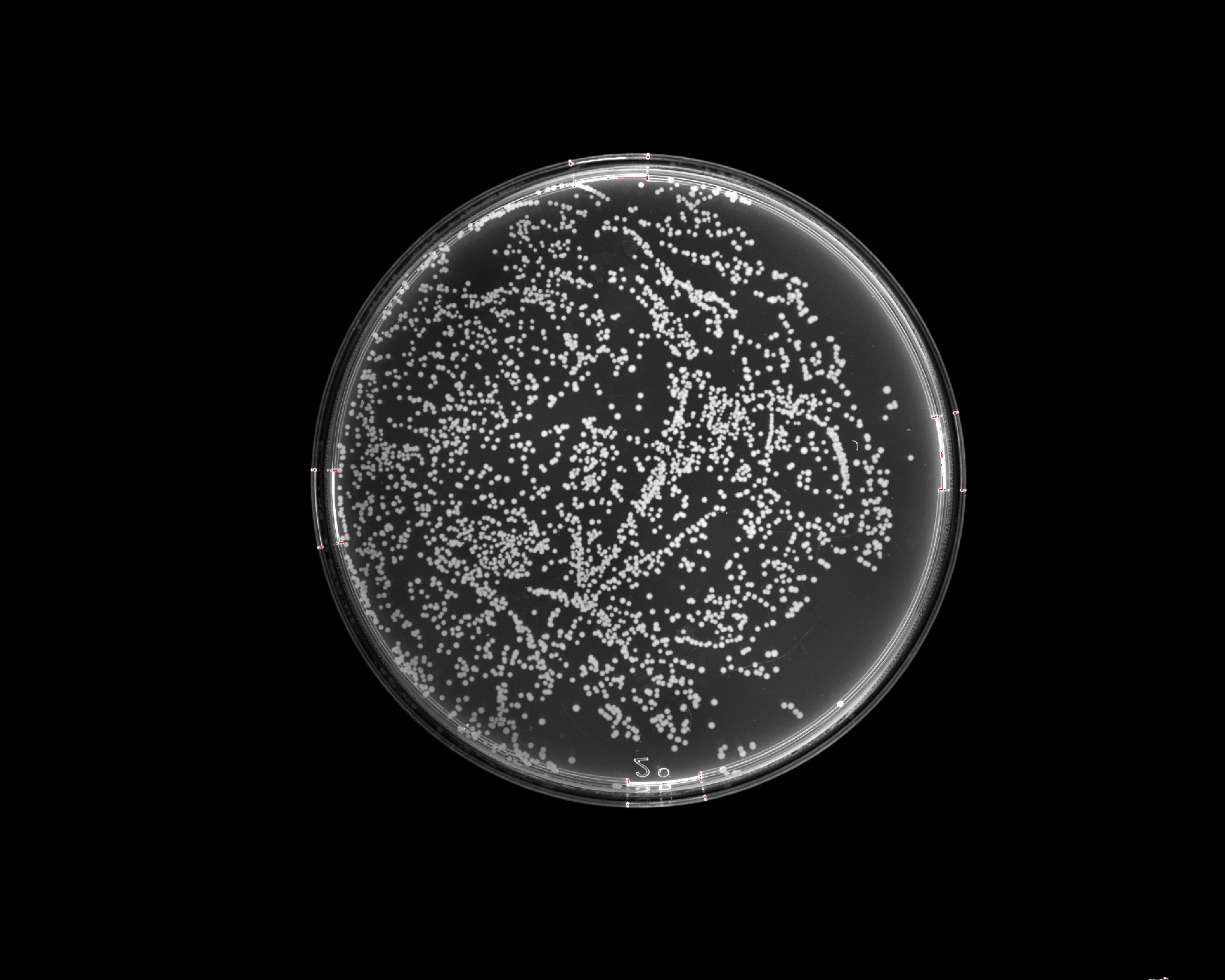

Supplement: Supplementary file 3 [file DataSheet1.ZIP › Antibacterial activity/Agar reculture observation/S. aureus/S Ti-100-1(SYBR Gold).jpg]

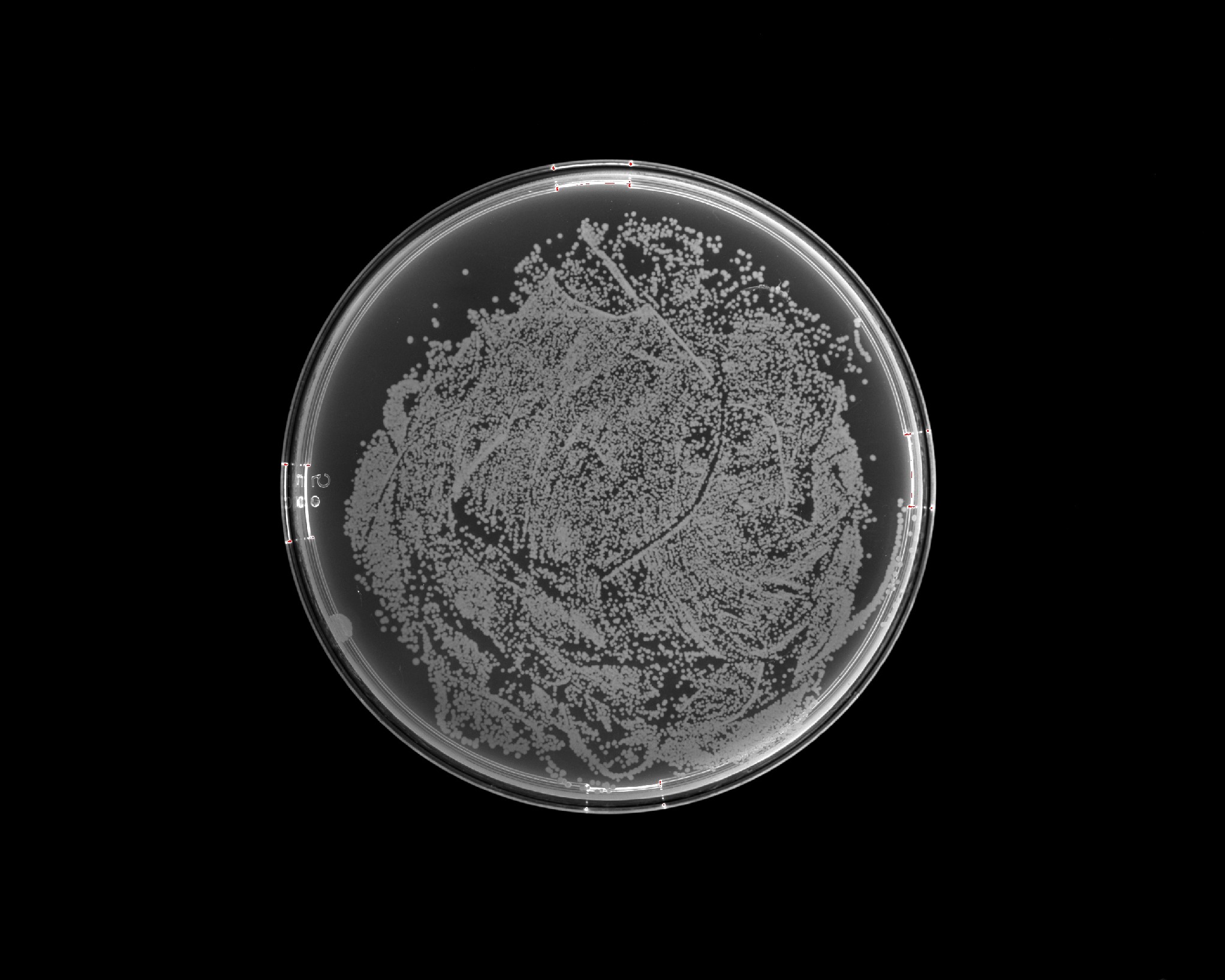

Supplement: Supplementary file 3 [file DataSheet1.ZIP › Antibacterial activity/Agar reculture observation/S. aureus/S Ti-10-1(SYBR Gold).jpg]

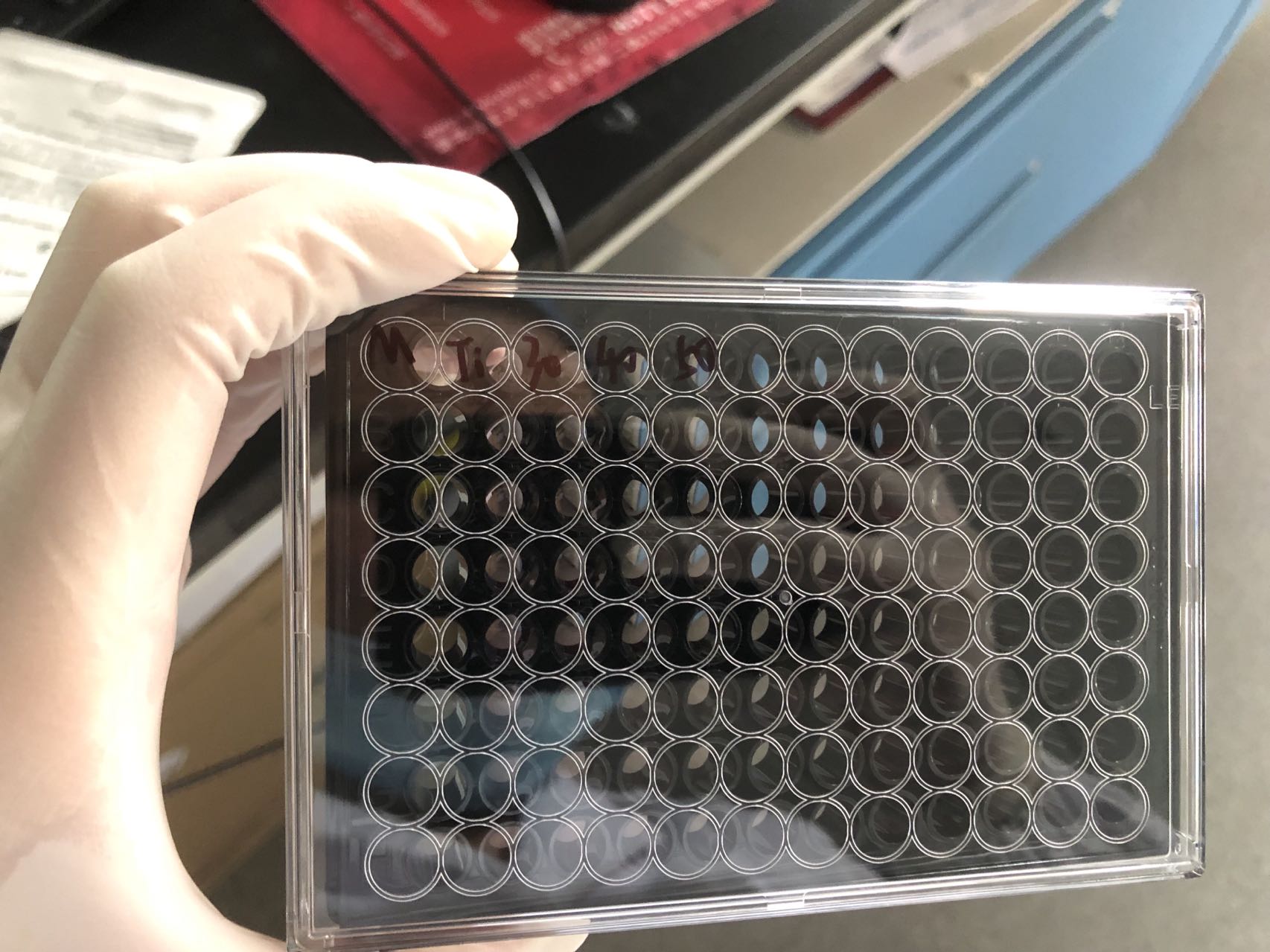

Supplement: Supplementary file 3 [file DataSheet1.ZIP › Antibacterial activity/Evaluation of bacterial viability/Mrsa/am96┐╫║┌░σ Mrsa.jpg]

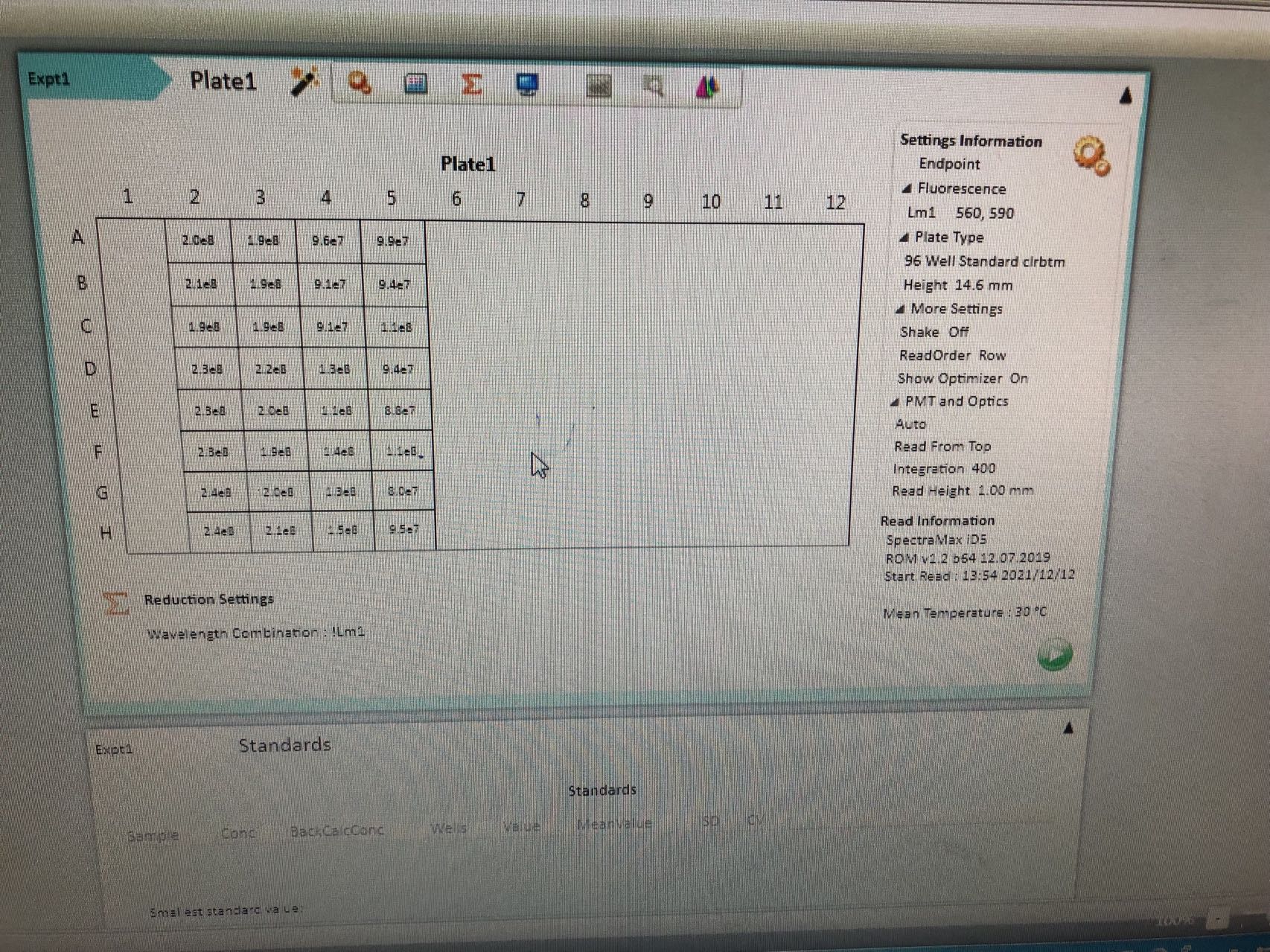

Supplement: Supplementary file 3 [file DataSheet1.ZIP › Antibacterial activity/Evaluation of bacterial viability/Mrsa/╧╕╛·╗ε┴a╩2╛▌ Mrsa.jpg]

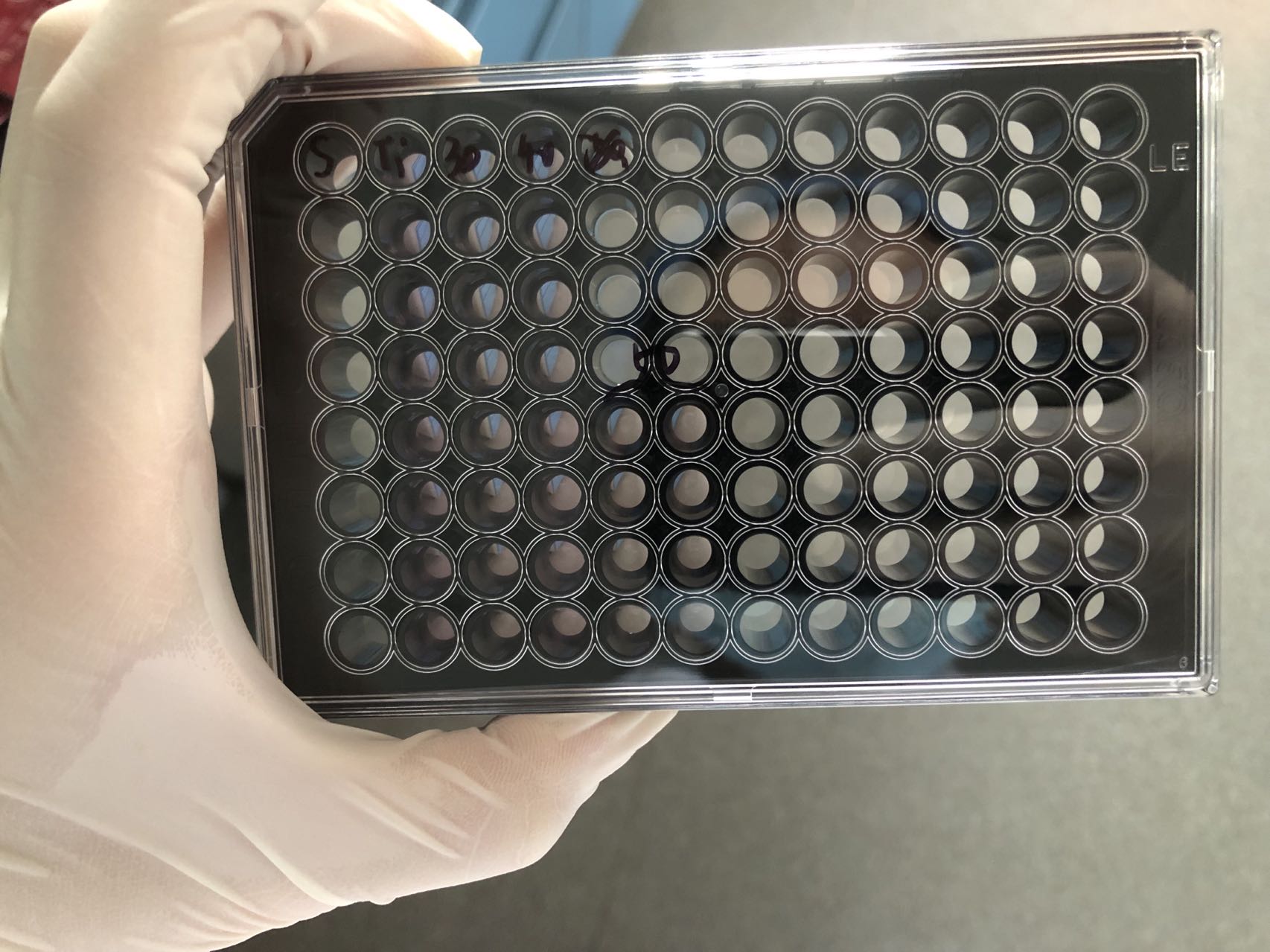

Supplement: Supplementary file 3 [file DataSheet1.ZIP › Antibacterial activity/Evaluation of bacterial viability/S. aureus/am96┐╫║┌░σ ╜≡╞╧.jpg]

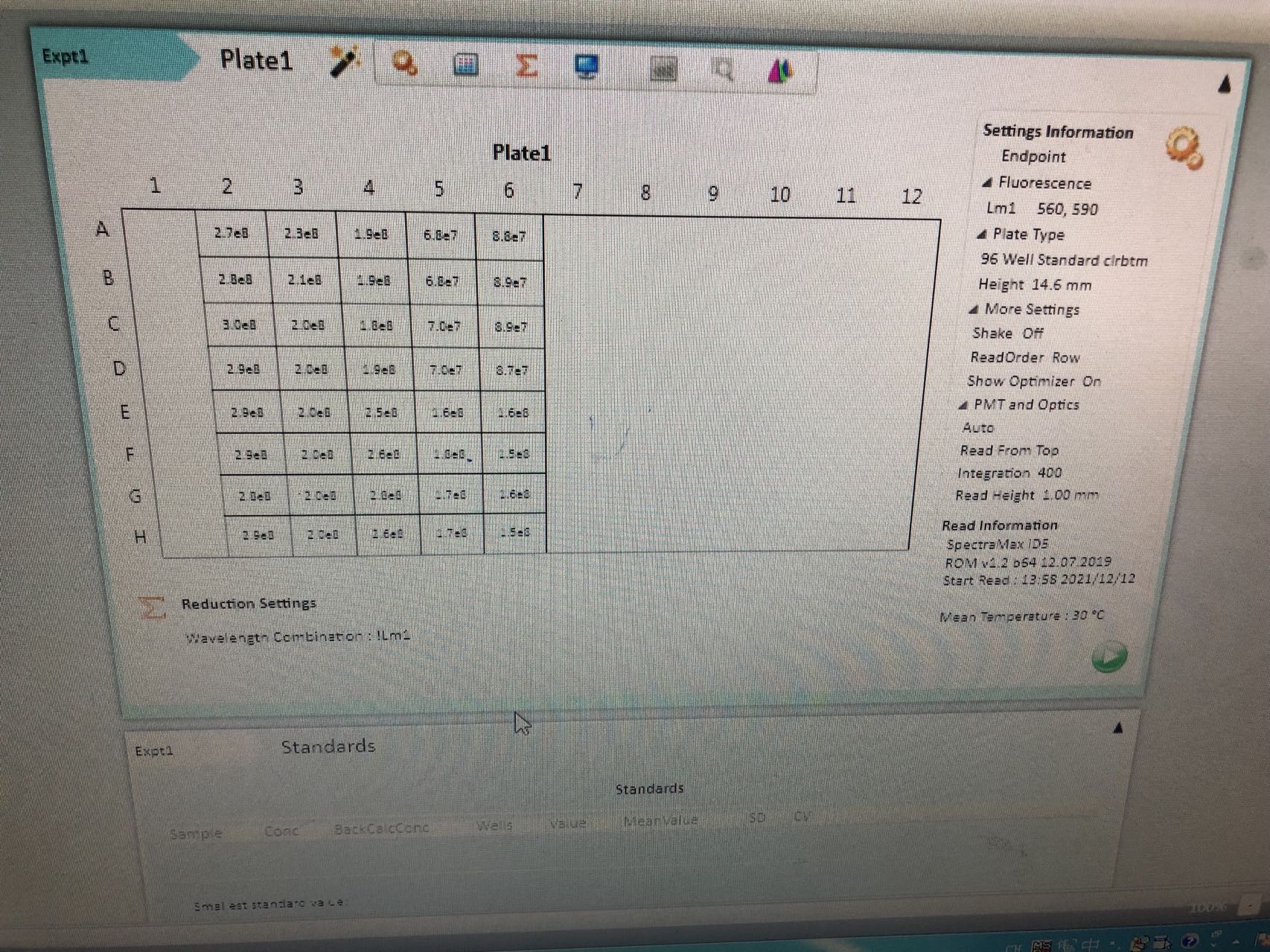

Supplement: Supplementary file 3 [file DataSheet1.ZIP › Antibacterial activity/Evaluation of bacterial viability/S. aureus/╧╕╛·╗ε┴a╩2╛▌ ╜≡╞╧.jpg]

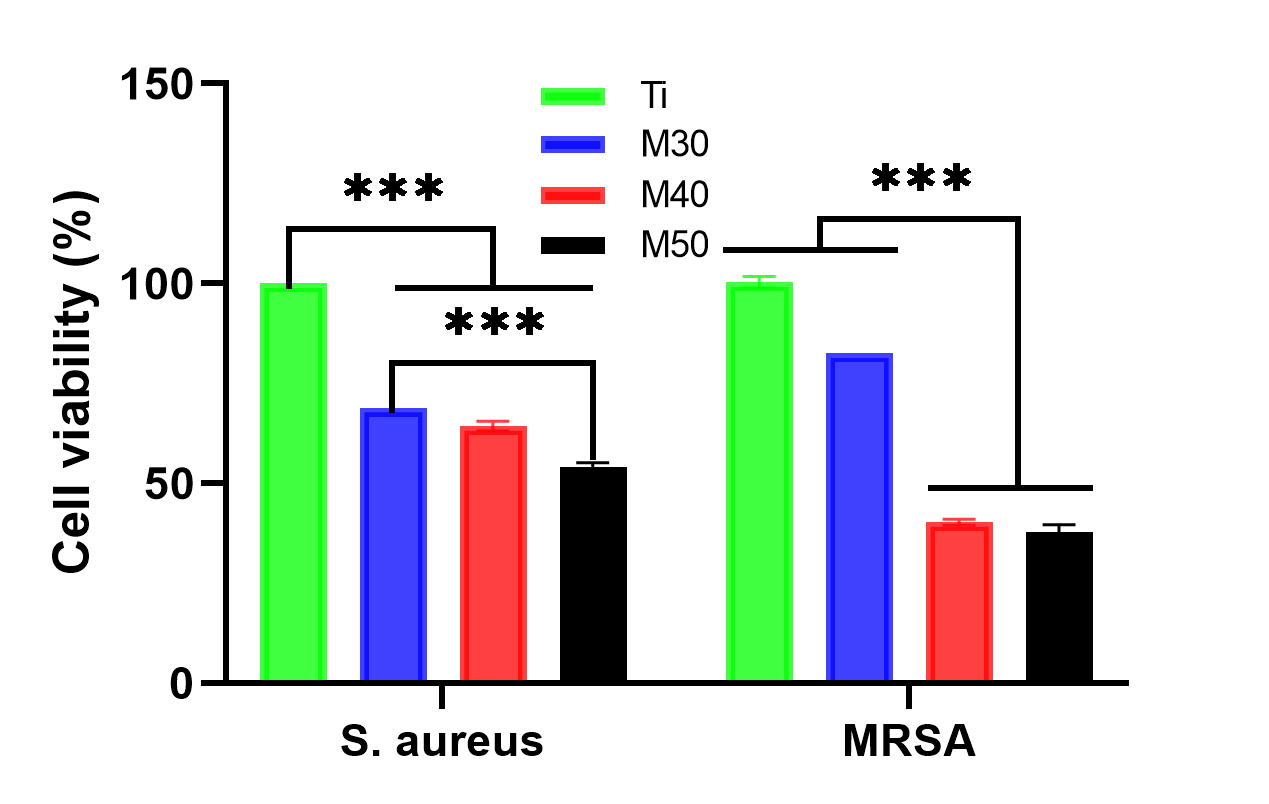

Supplement: Supplementary file 3 [file DataSheet1.ZIP › Antibacterial activity/Evaluation of bacterial viability/╫ε╓╒╩2╛▌ú1⁄2═╝/Data 1.tif]

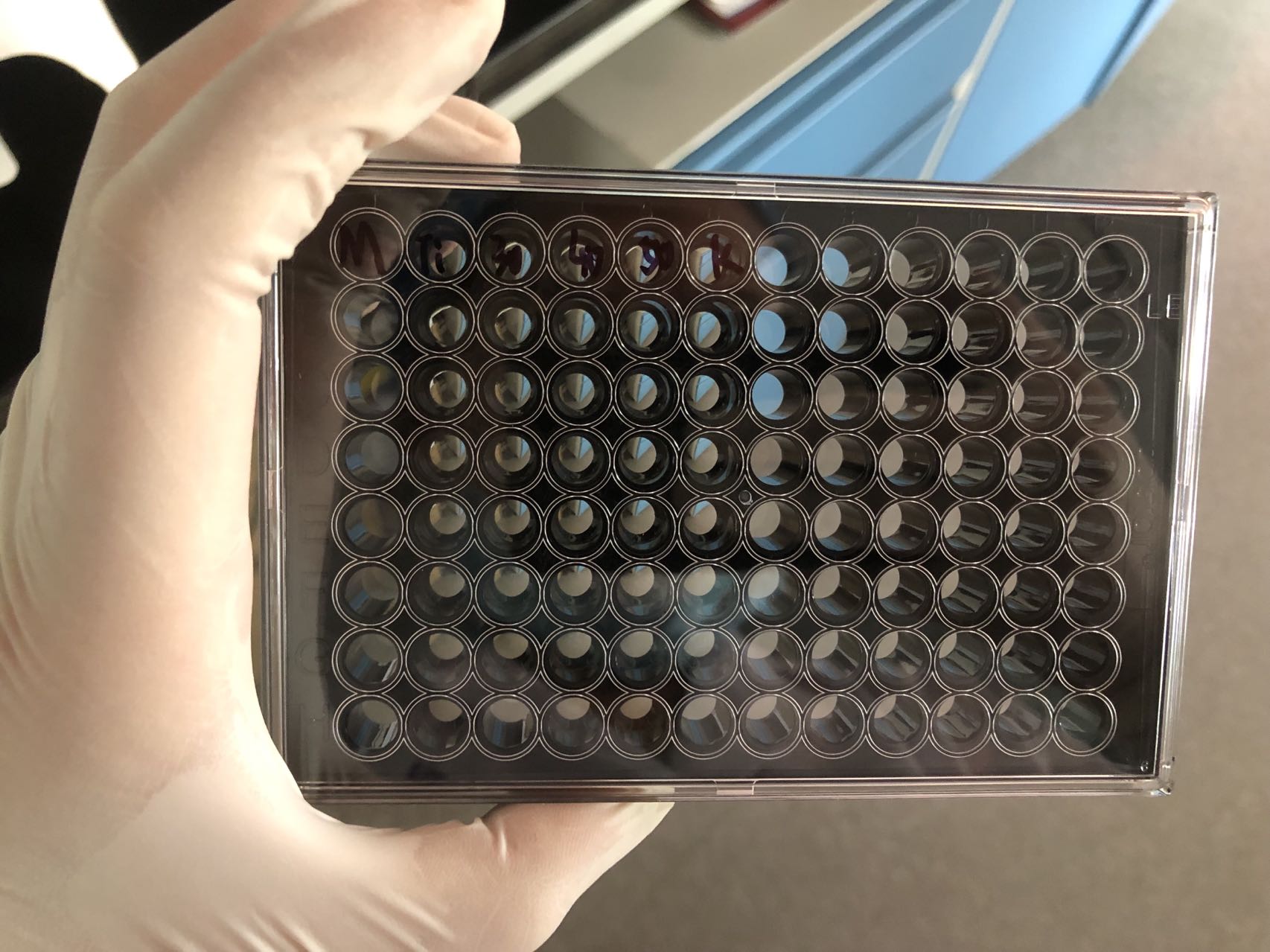

Supplement: Supplementary file 3 [file DataSheet1.ZIP › Antibacterial activity/Intracellular reactive oxide assay/Mrsa/96┐╫║┌░σ Mrsa.jpg]

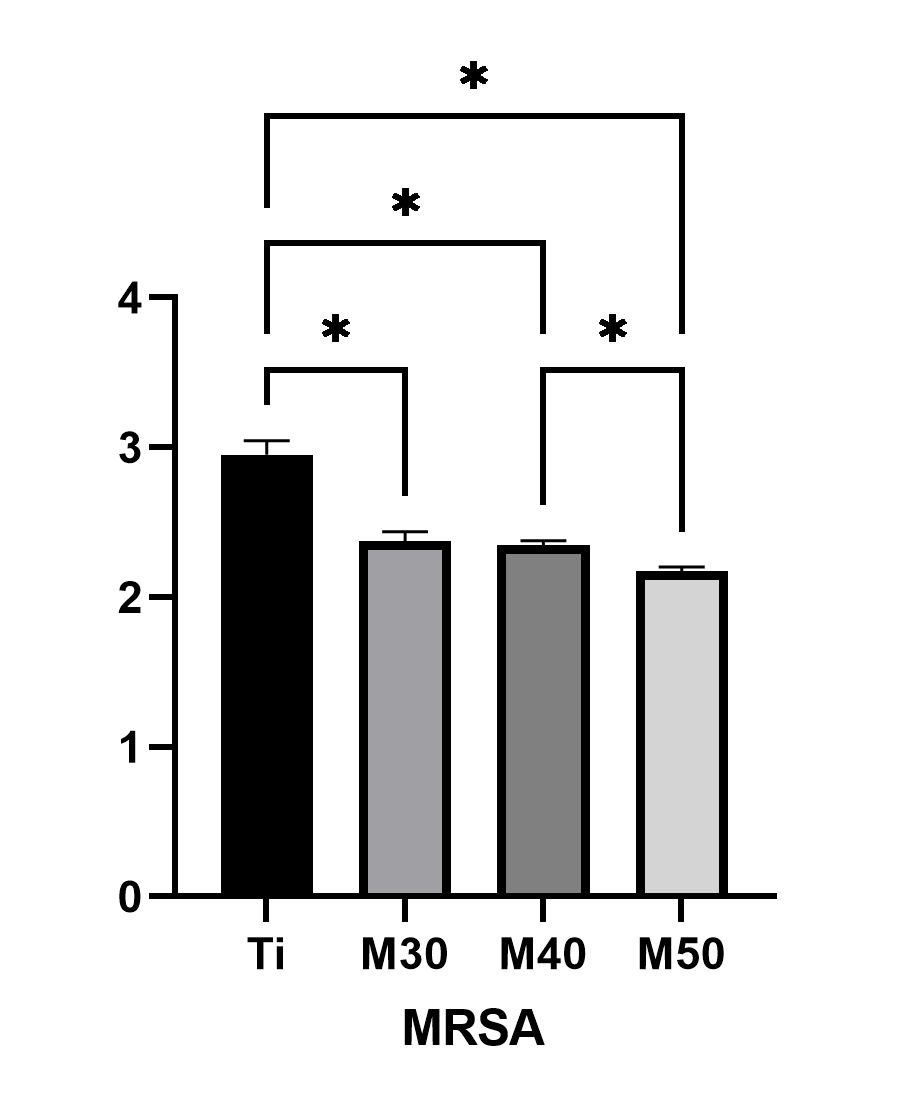

Supplement: Supplementary file 3 [file DataSheet1.ZIP › Antibacterial activity/Intracellular reactive oxide assay/Mrsa/Data 1.tif]

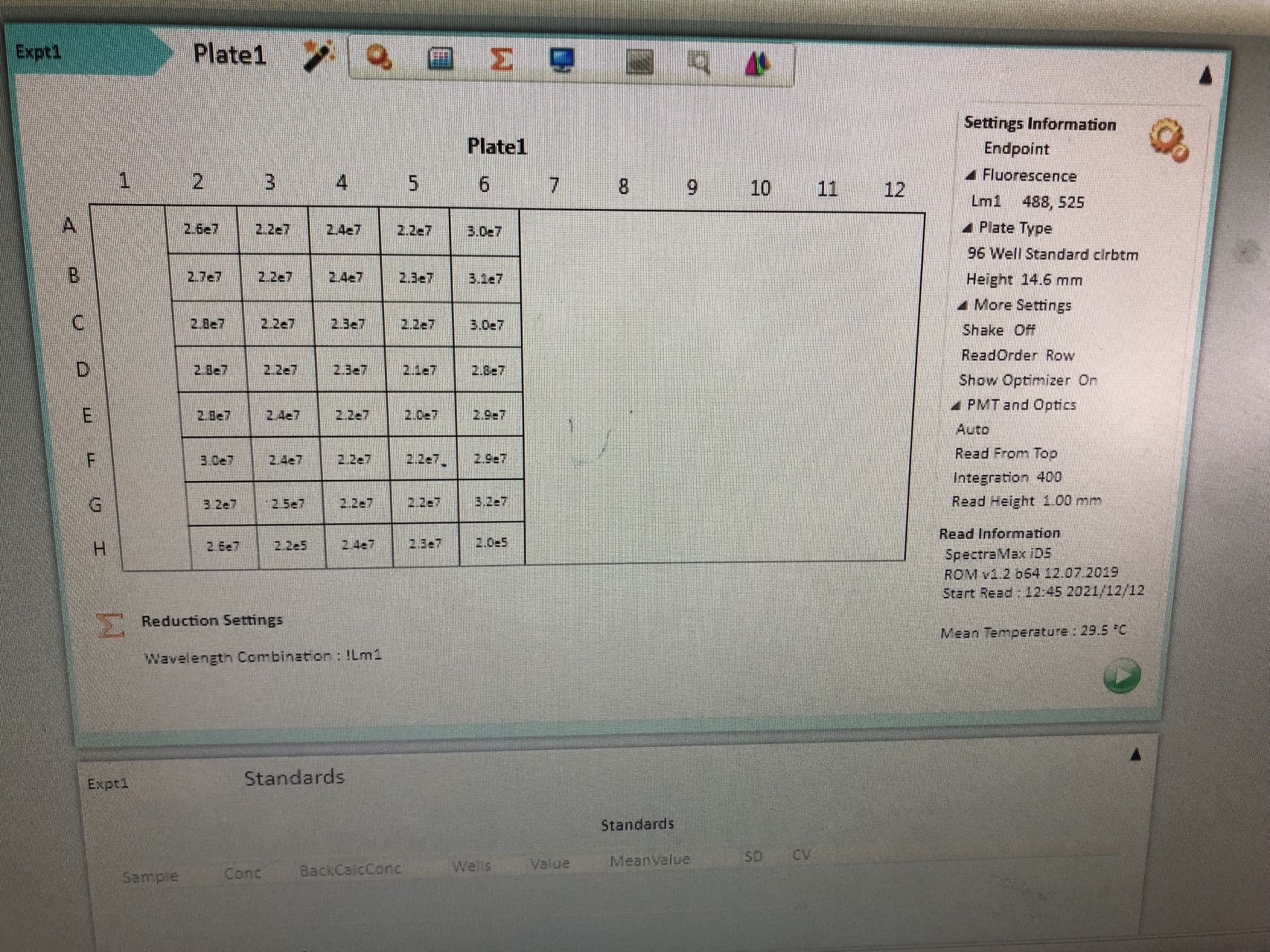

Supplement: Supplementary file 3 [file DataSheet1.ZIP › Antibacterial activity/Intracellular reactive oxide assay/Mrsa/╗ε╨╘╤⌡╩2╛▌ Mrsa.jpg]

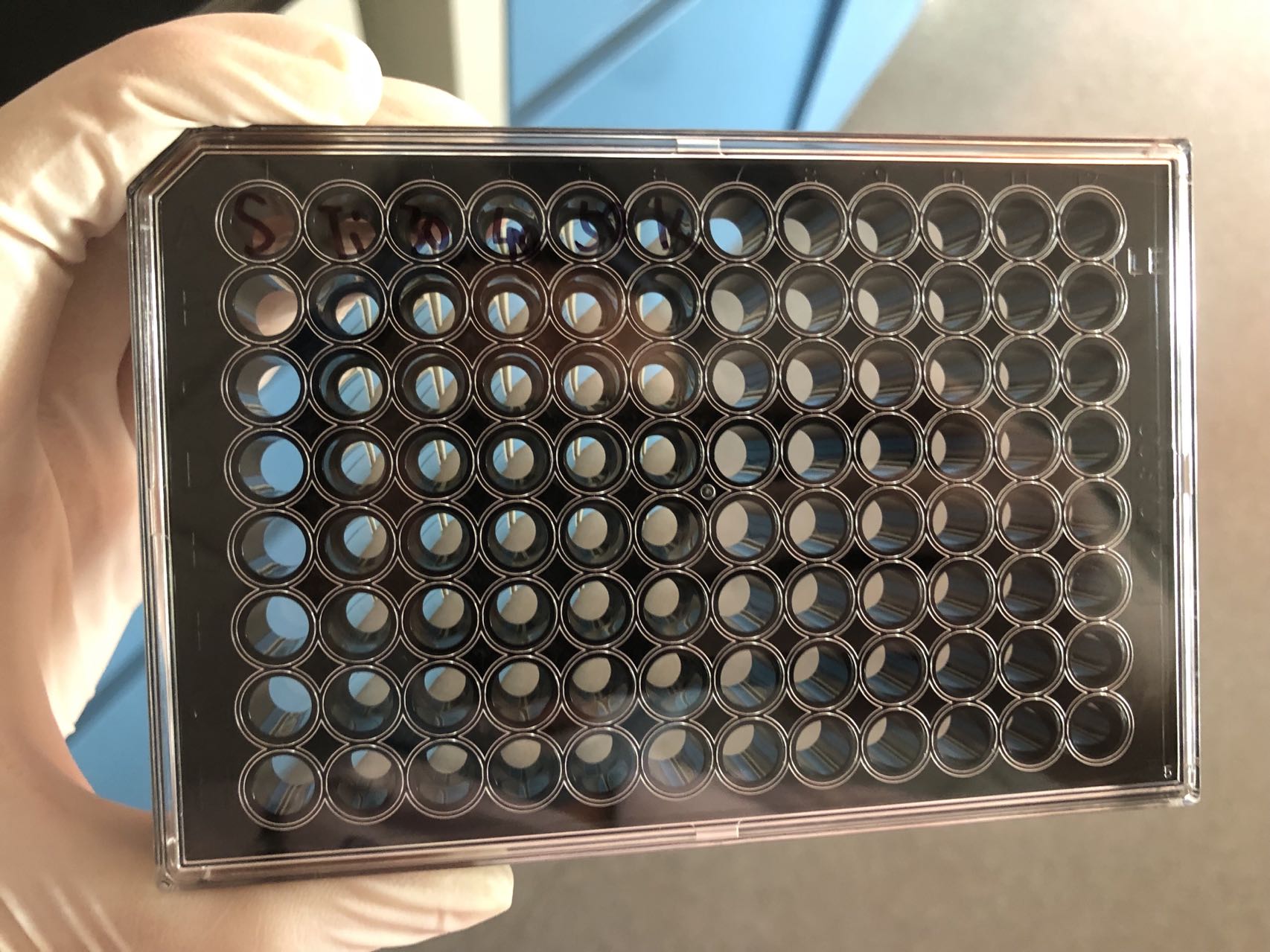

Supplement: Supplementary file 3 [file DataSheet1.ZIP › Antibacterial activity/Intracellular reactive oxide assay/S. aureus/96┐╫║┌░σ ╜≡╞╧.jpg]

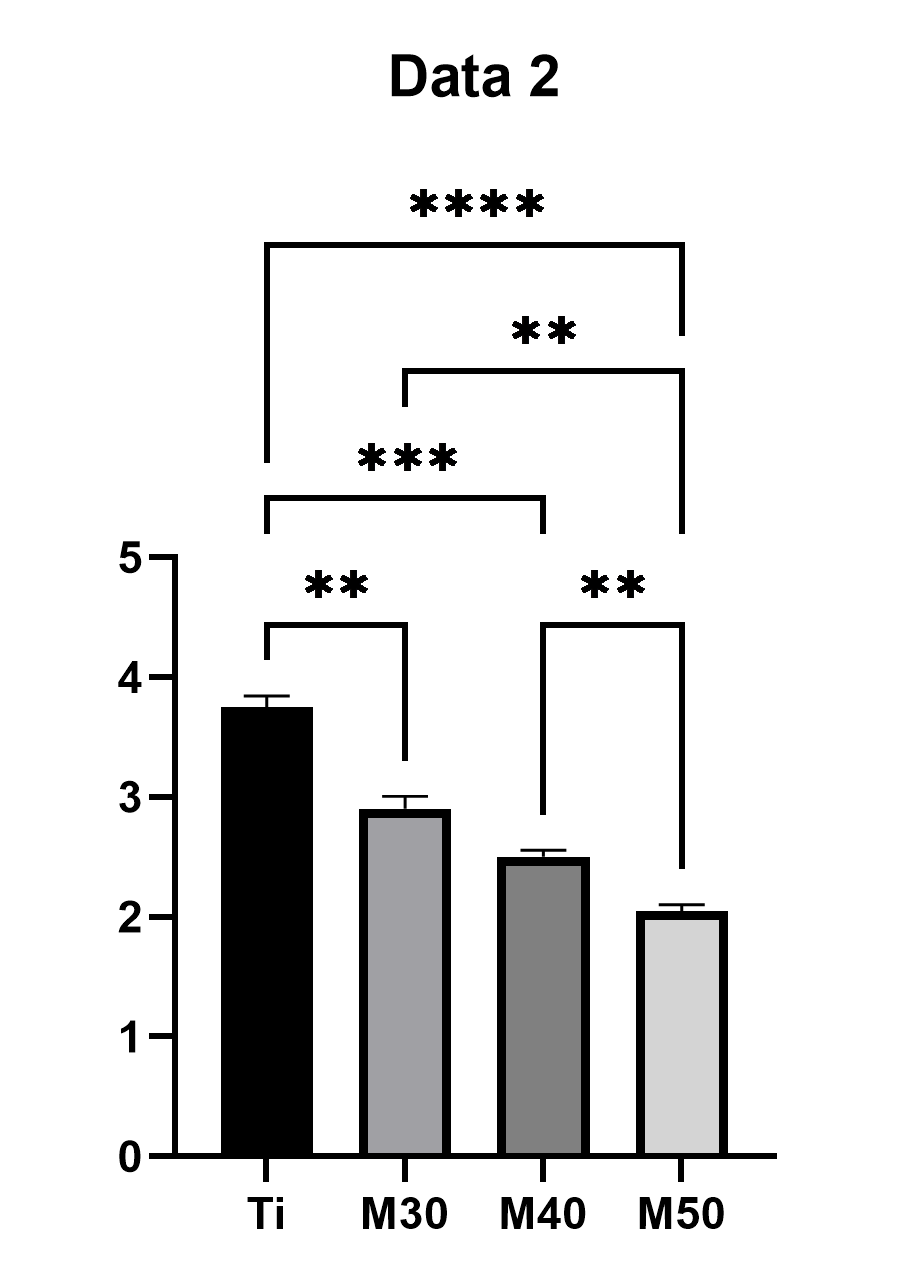

Supplement: Supplementary file 3 [file DataSheet1.ZIP › Antibacterial activity/Intracellular reactive oxide assay/S. aureus/Data 2.tif]

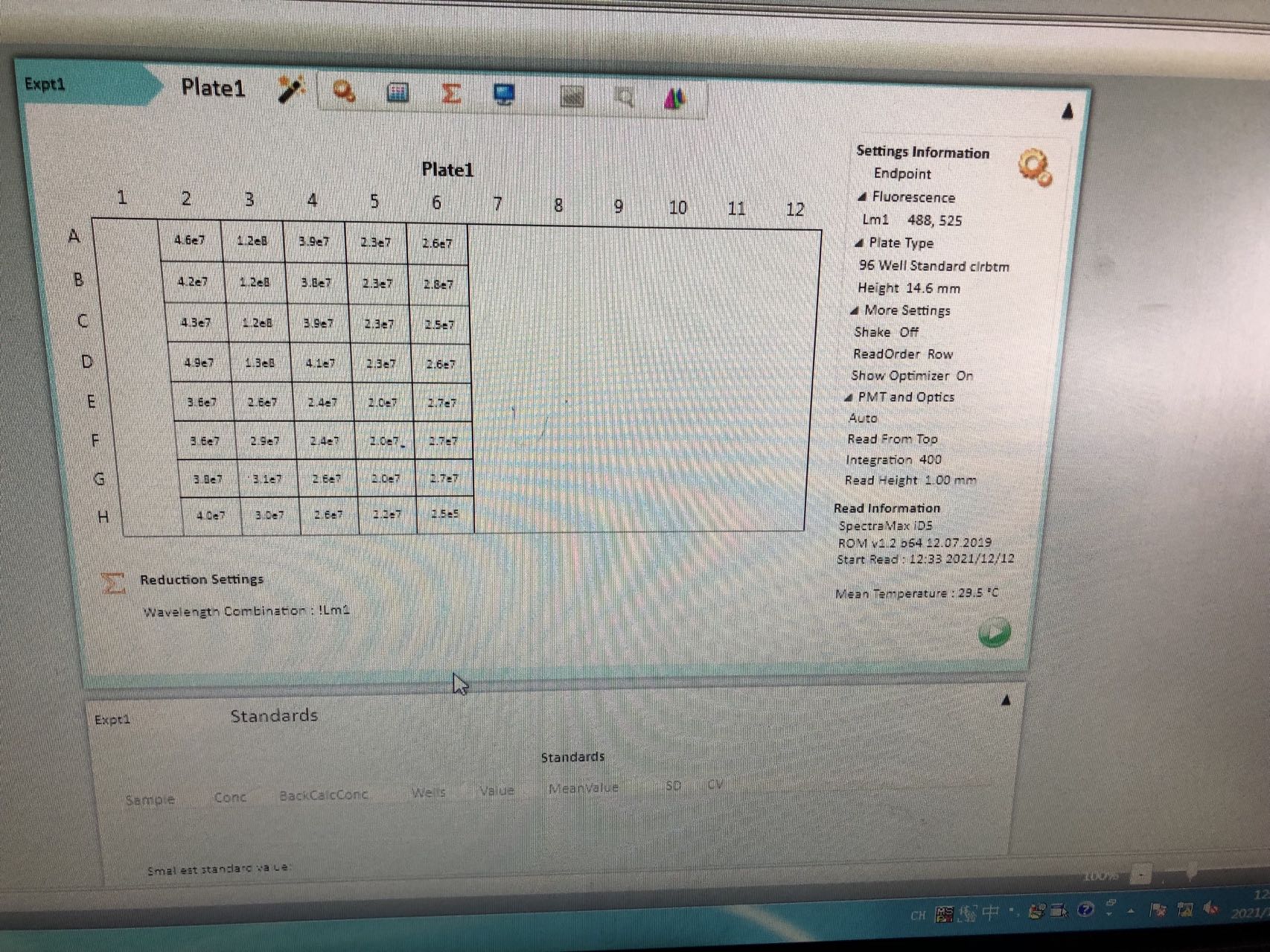

Supplement: Supplementary file 3 [file DataSheet1.ZIP › Antibacterial activity/Intracellular reactive oxide assay/S. aureus/╗ε╨╘╤⌡╩2╛▌ ╜≡╞╧.jpg]

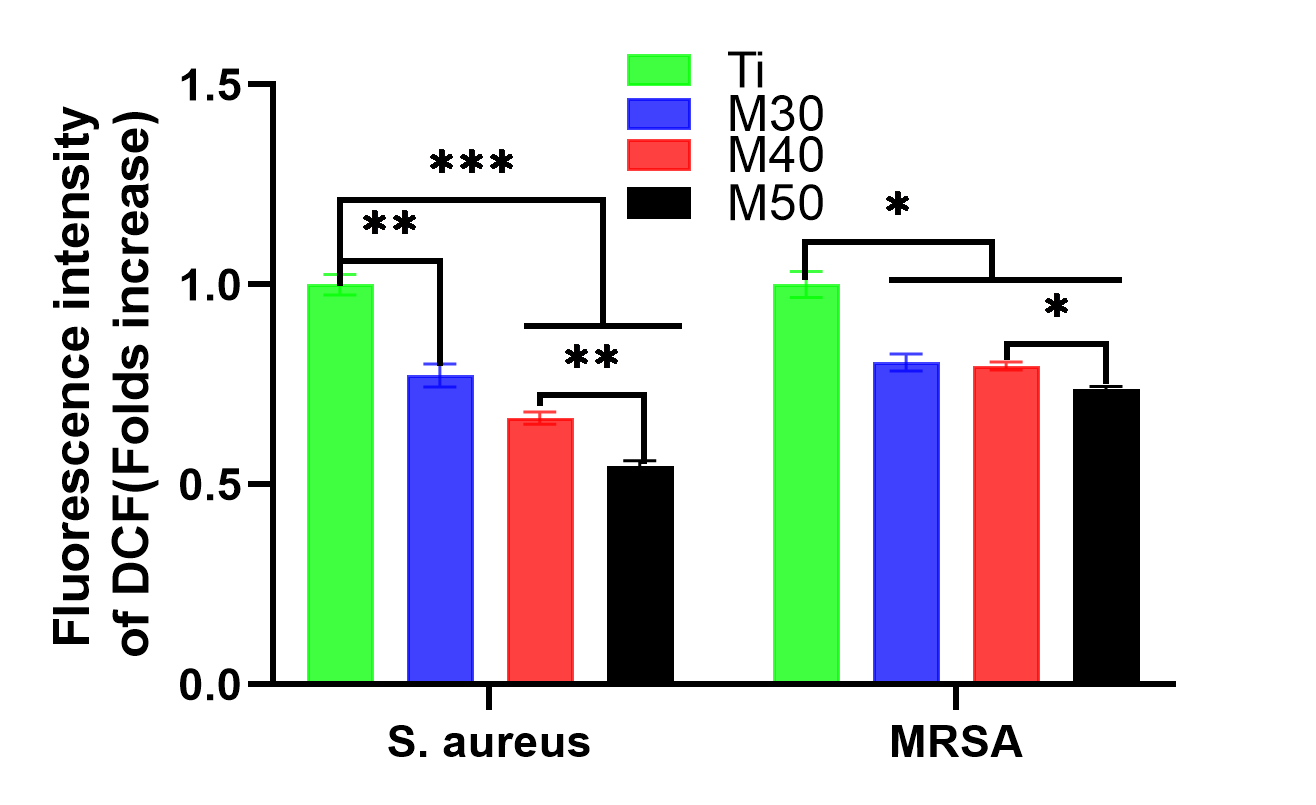

Supplement: Supplementary file 3 [file DataSheet1.ZIP › Antibacterial activity/Intracellular reactive oxide assay/╫ε╓╒╗¡═╝╩2╛▌ú1⁄2═╝/Data 3.tif]

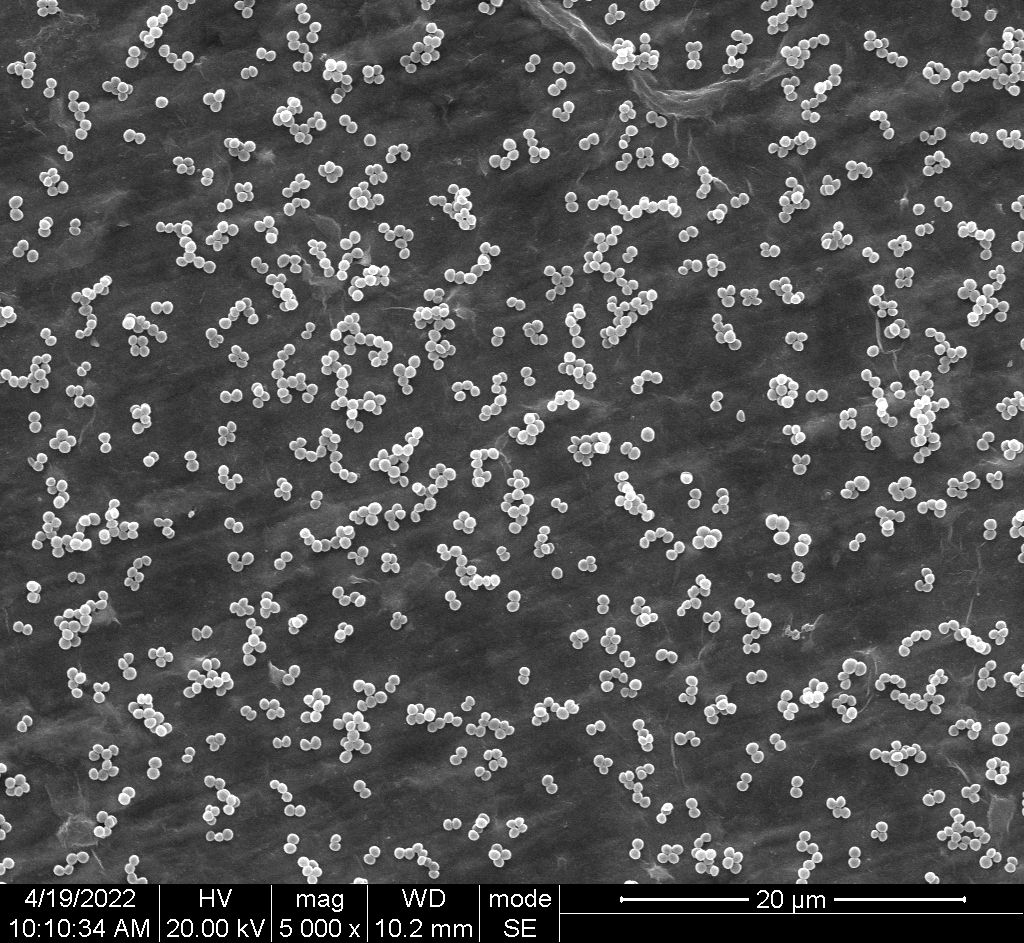

Supplement: Supplementary file 3 [file DataSheet1.ZIP › Antibacterial activity/SEM observation/MRSA/2 M30 5k.jpg]

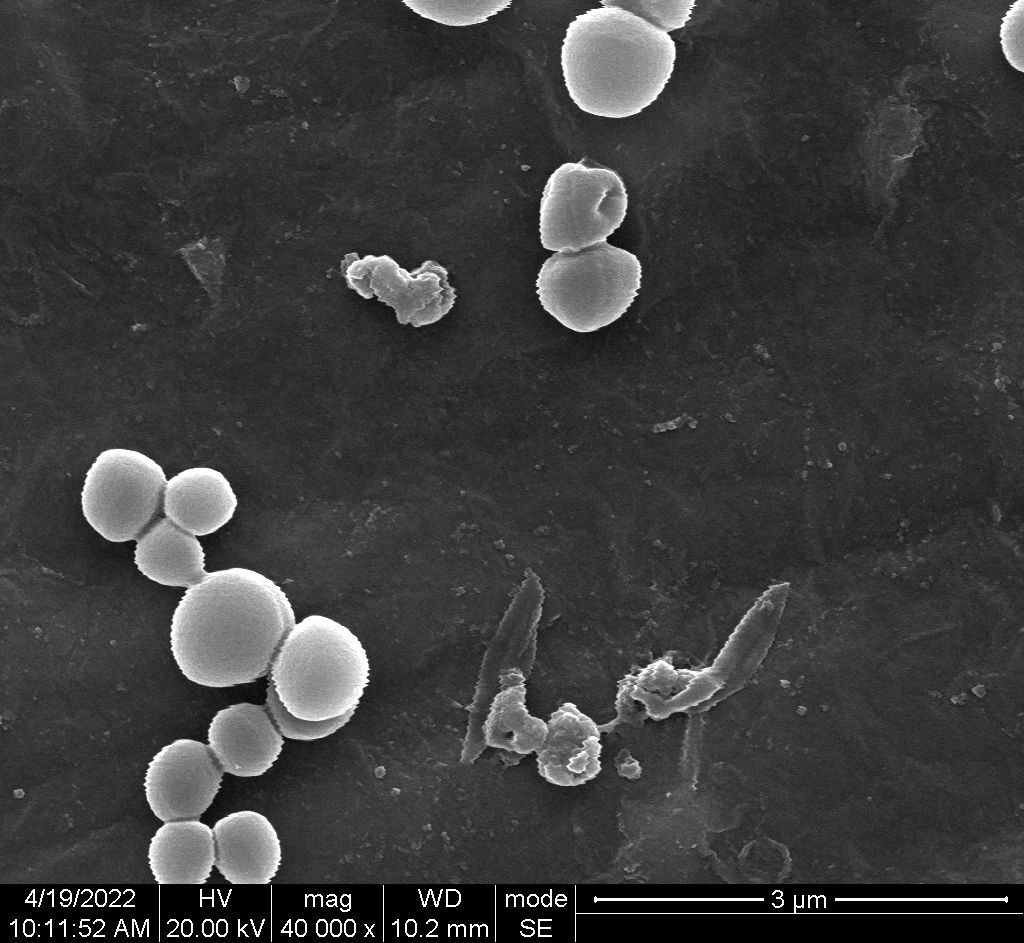

Supplement: Supplementary file 3 [file DataSheet1.ZIP › Antibacterial activity/SEM observation/MRSA/2_001 M30 4w.jpg]

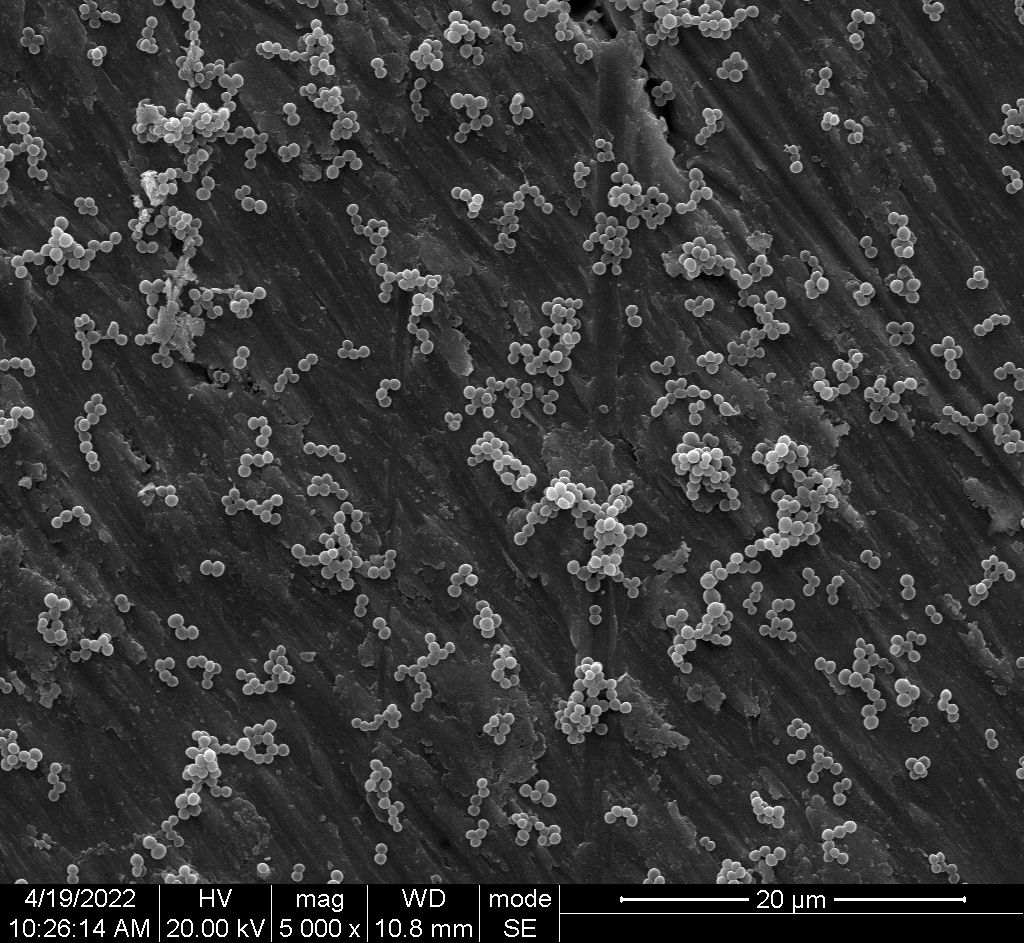

Supplement: Supplementary file 3 [file DataSheet1.ZIP › Antibacterial activity/SEM observation/MRSA/5_002 Ti 5k.jpg]

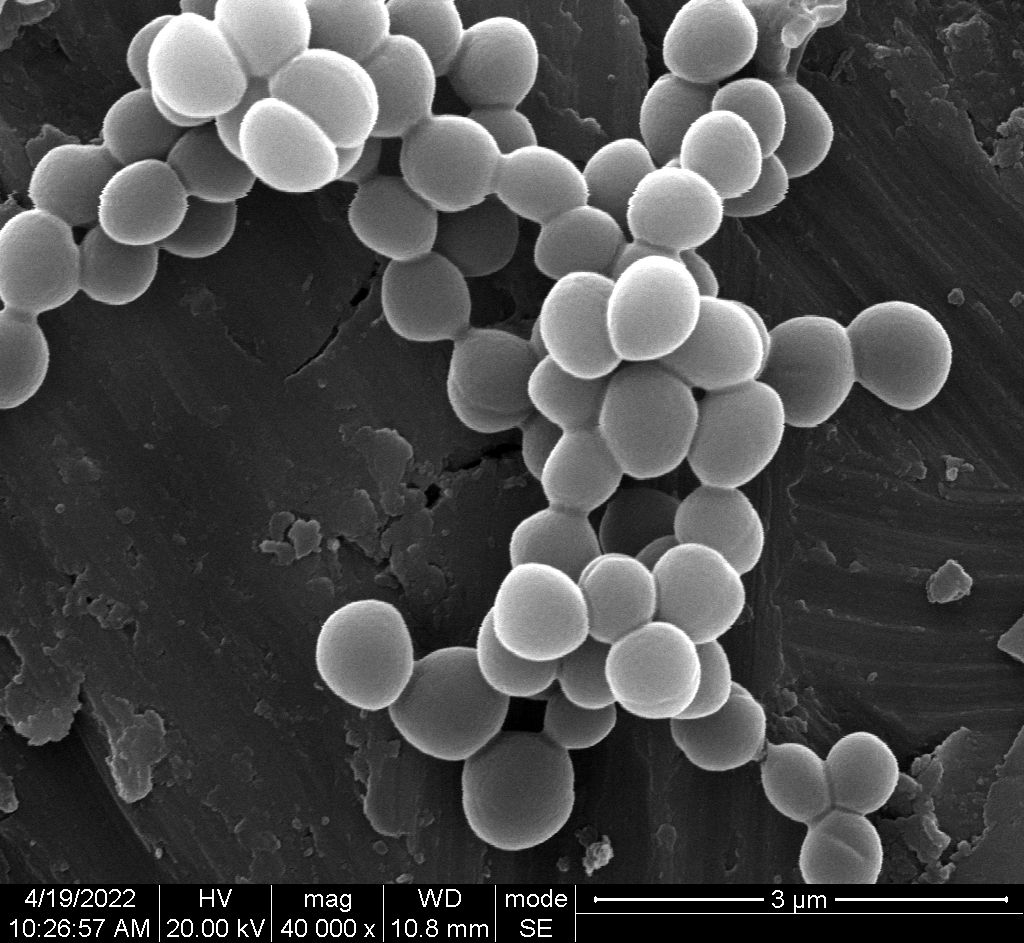

Supplement: Supplementary file 3 [file DataSheet1.ZIP › Antibacterial activity/SEM observation/MRSA/5_003 Ti 4w.jpg]

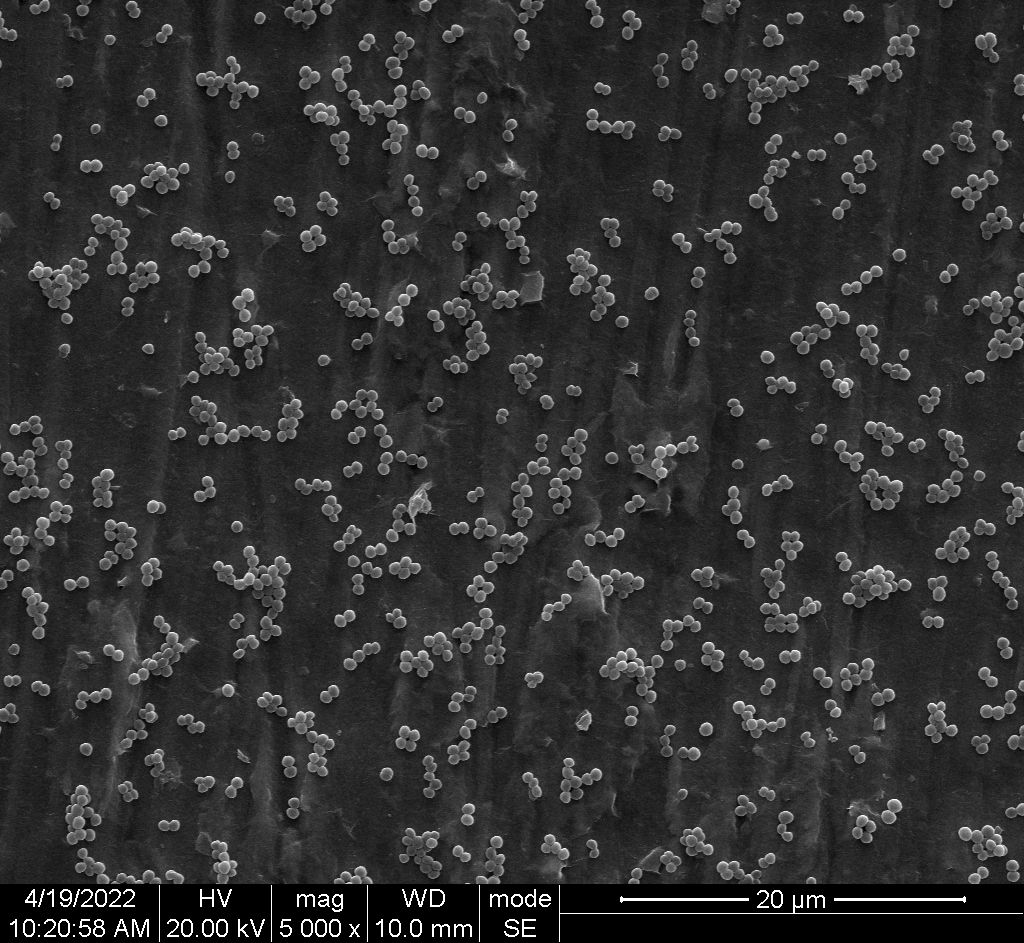

Supplement: Supplementary file 3 [file DataSheet1.ZIP › Antibacterial activity/SEM observation/MRSA/6 M40 5k.jpg]

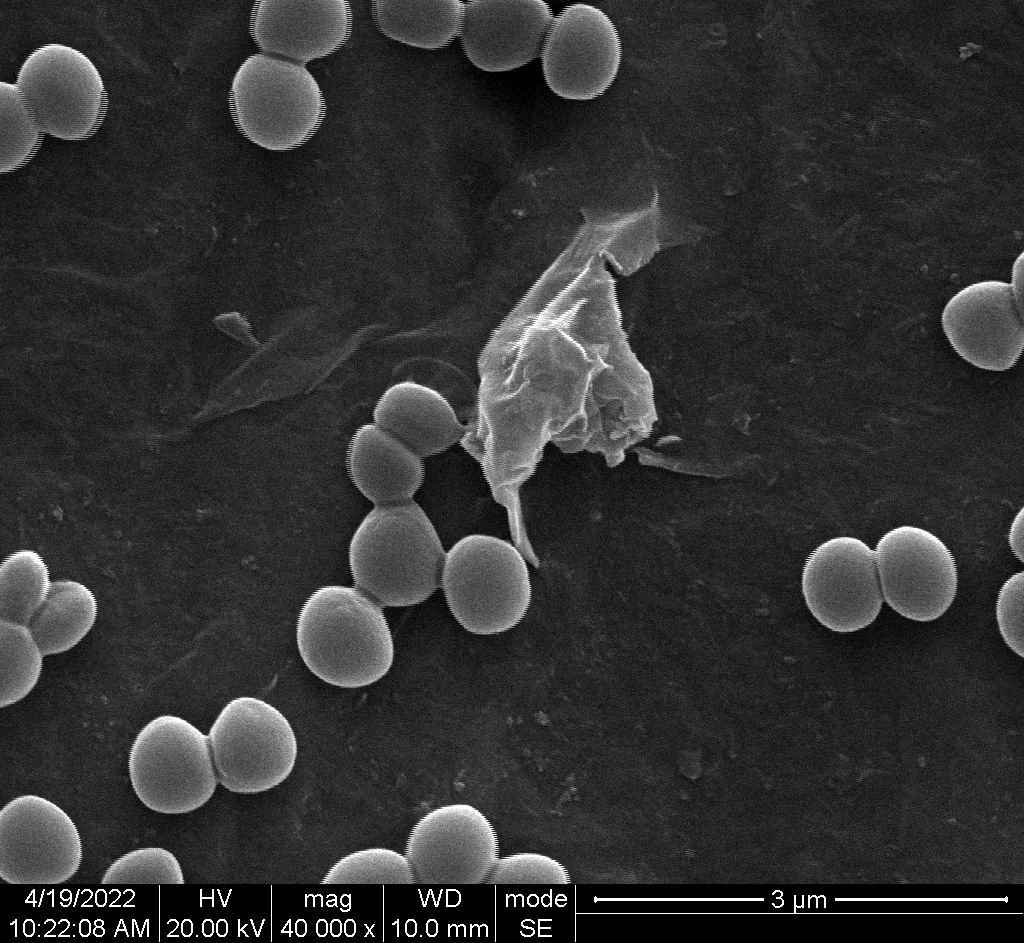

Supplement: Supplementary file 3 [file DataSheet1.ZIP › Antibacterial activity/SEM observation/MRSA/6_001 M40 4w.jpg]

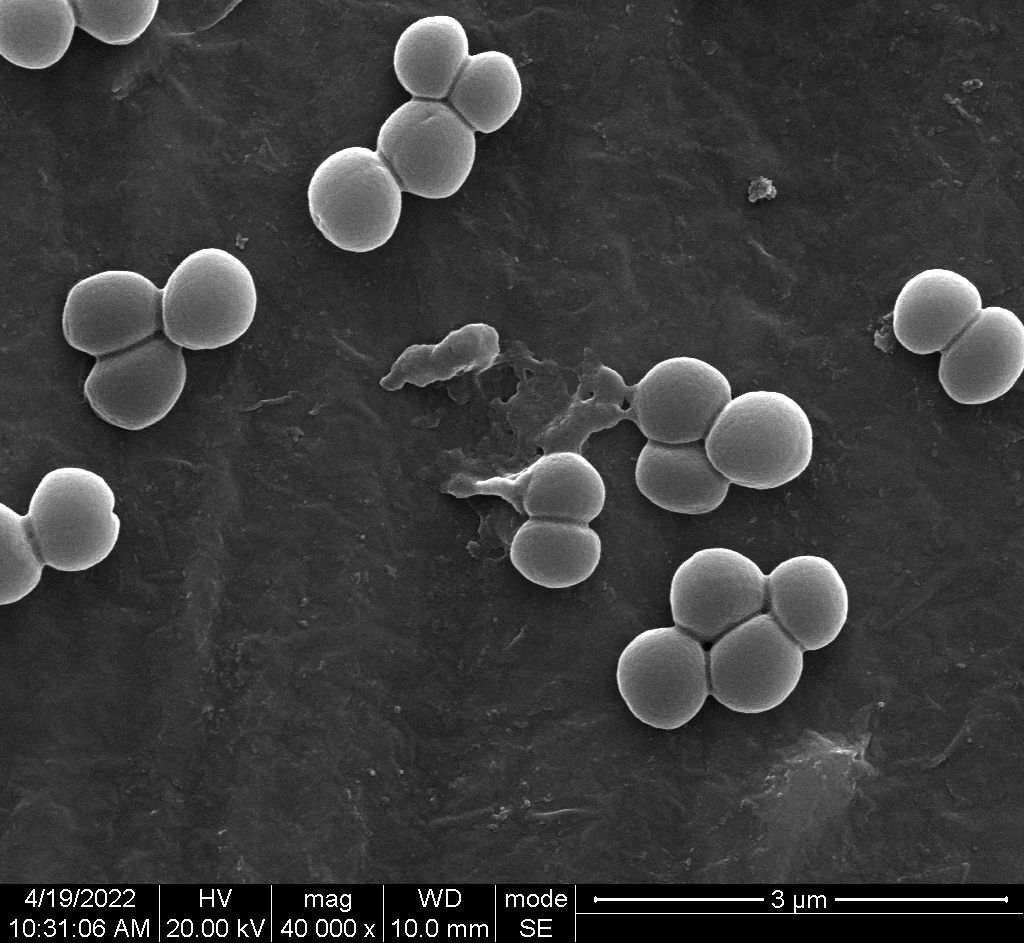

Supplement: Supplementary file 3 [file DataSheet1.ZIP › Antibacterial activity/SEM observation/MRSA/6_002 M50 4w.jpg]

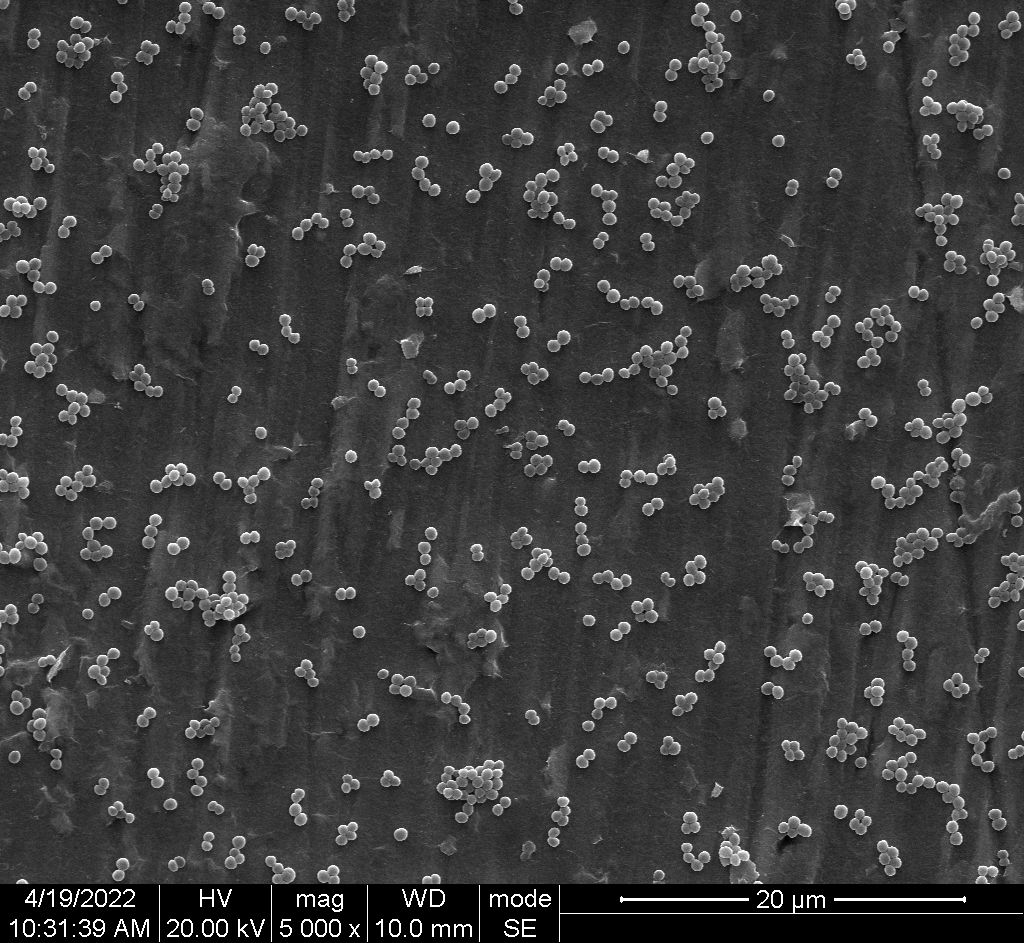

Supplement: Supplementary file 3 [file DataSheet1.ZIP › Antibacterial activity/SEM observation/MRSA/6-003 M50 5k.jpg]

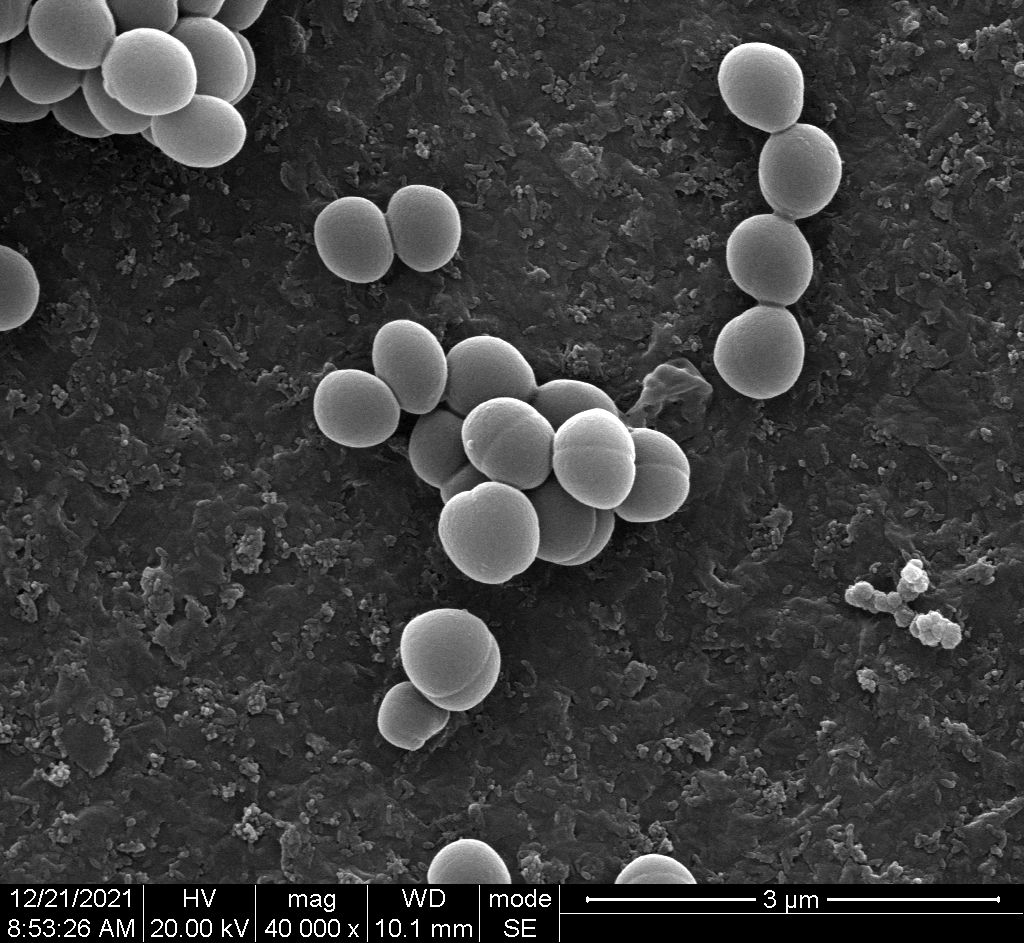

Supplement: Supplementary file 3 [file DataSheet1.ZIP › Antibacterial activity/SEM observation/S. aureus/M30 4w.jpg]

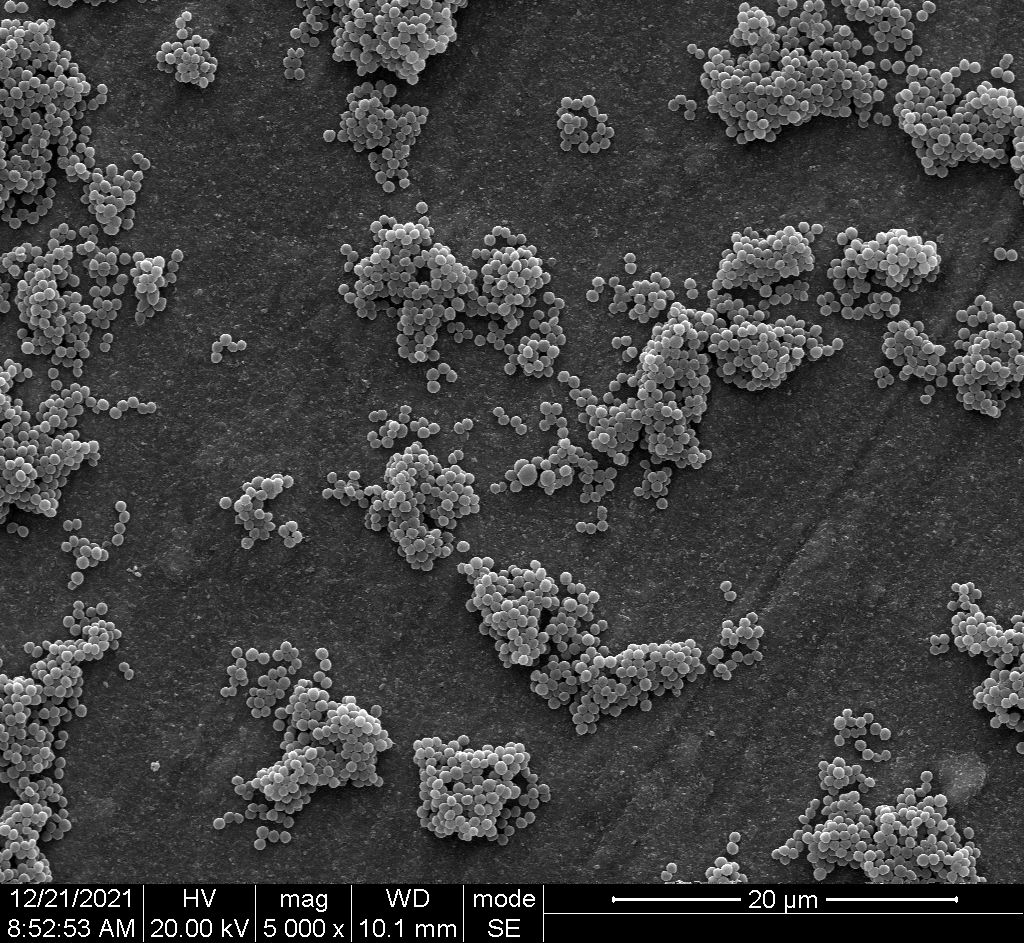

Supplement: Supplementary file 3 [file DataSheet1.ZIP › Antibacterial activity/SEM observation/S. aureus/M30 5k.jpg]

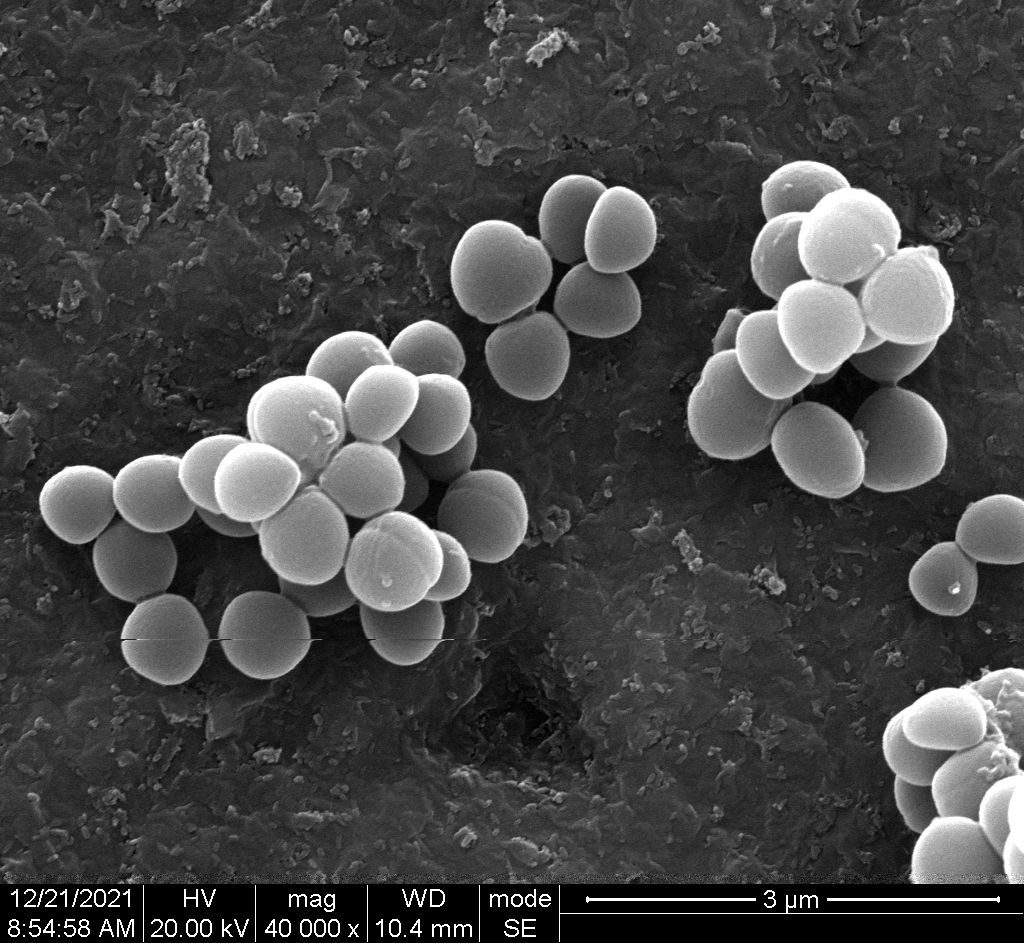

Supplement: Supplementary file 3 [file DataSheet1.ZIP › Antibacterial activity/SEM observation/S. aureus/M40 4w.jpg]

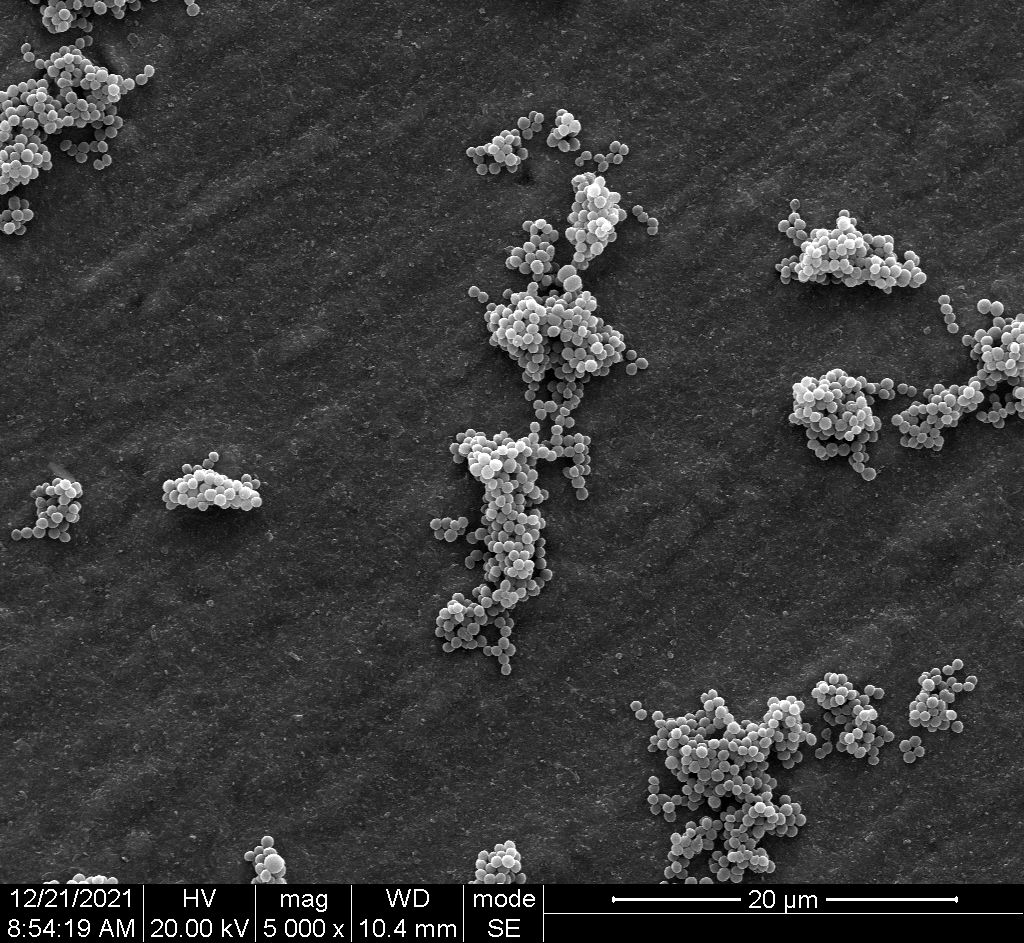

Supplement: Supplementary file 3 [file DataSheet1.ZIP › Antibacterial activity/SEM observation/S. aureus/M40 5k.jpg]

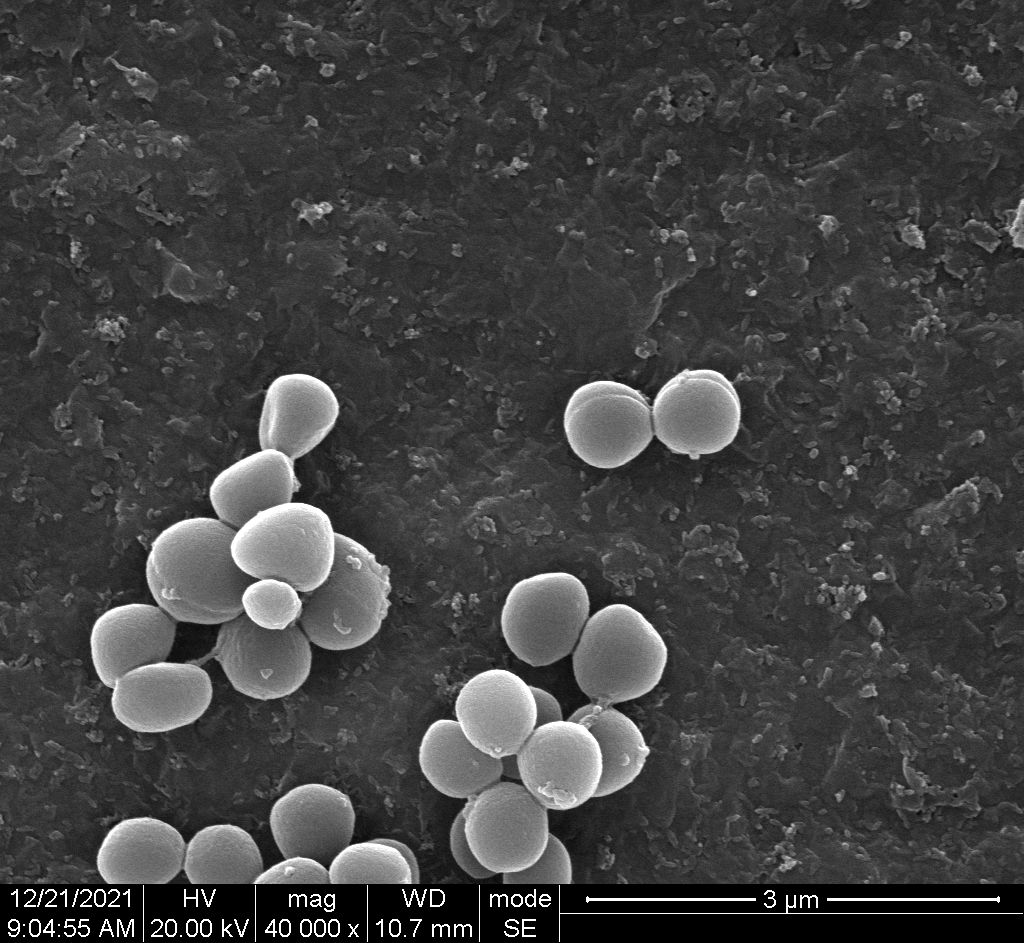

Supplement: Supplementary file 3 [file DataSheet1.ZIP › Antibacterial activity/SEM observation/S. aureus/M50 4w.jpg]

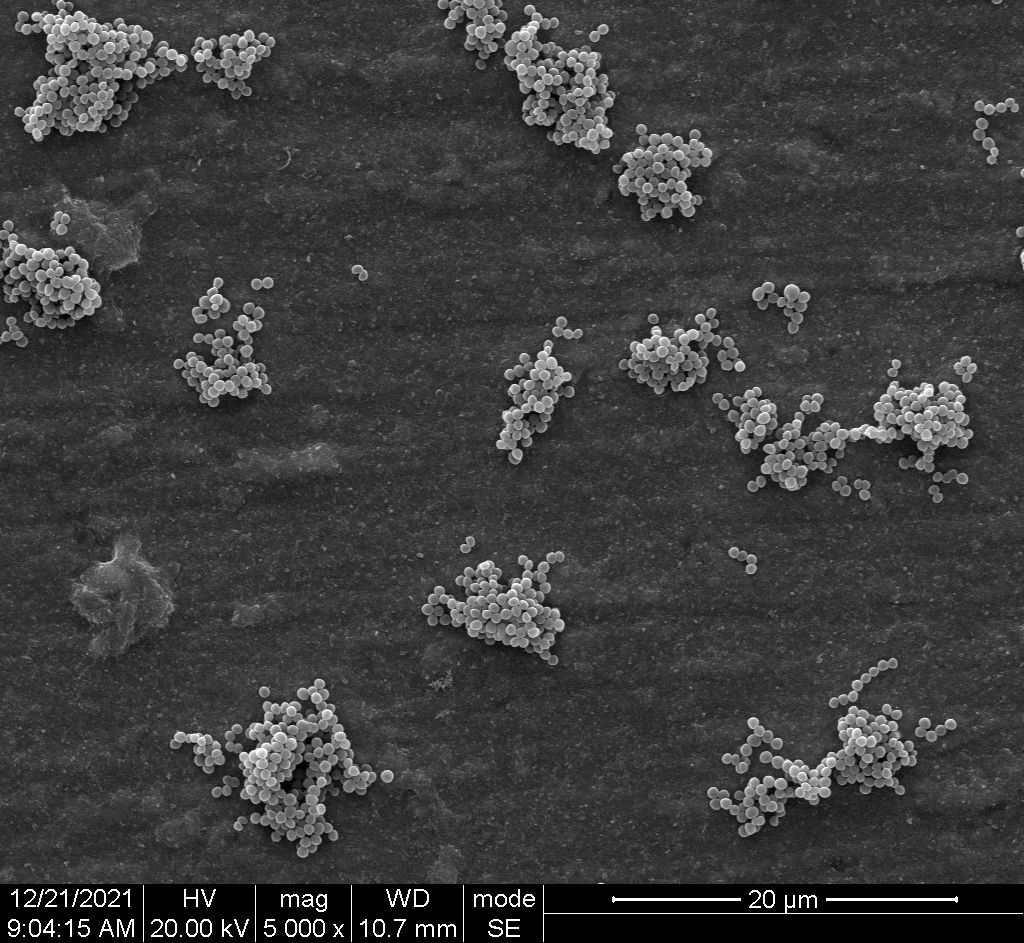

Supplement: Supplementary file 3 [file DataSheet1.ZIP › Antibacterial activity/SEM observation/S. aureus/M50 5k.jpg]

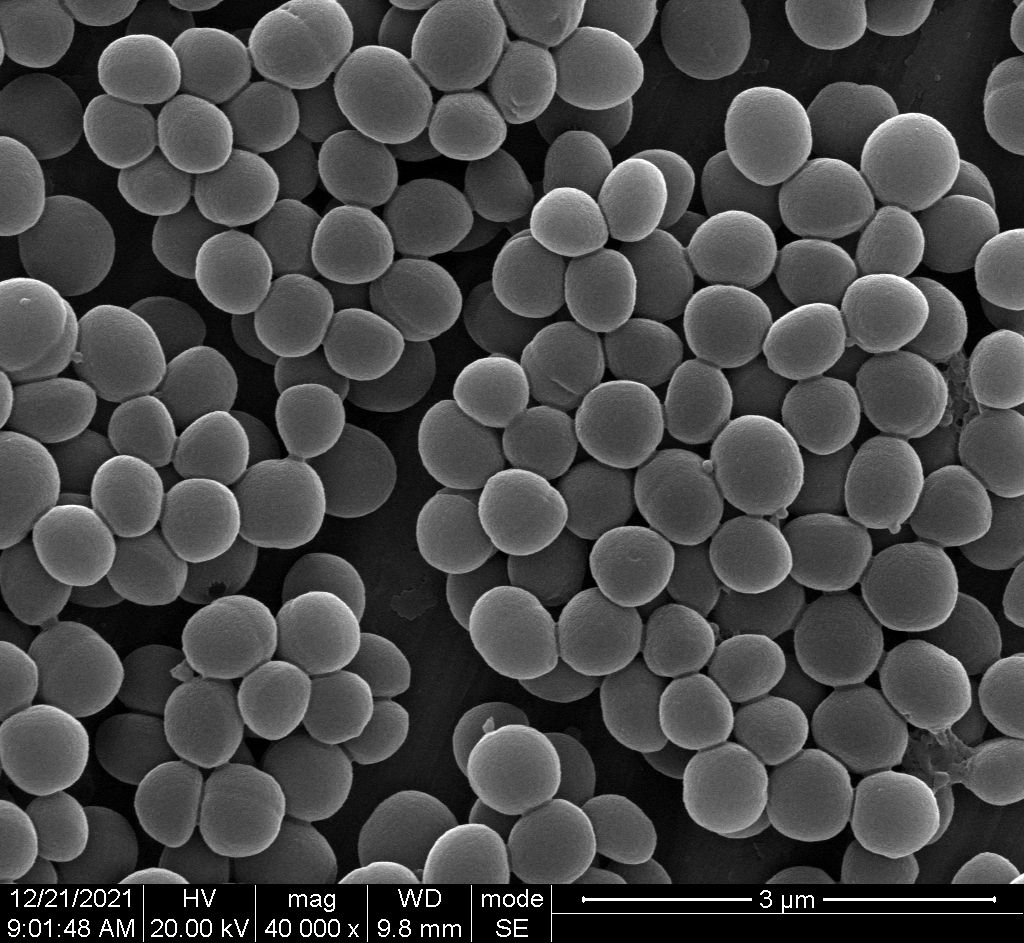

Supplement: Supplementary file 3 [file DataSheet1.ZIP › Antibacterial activity/SEM observation/S. aureus/Ti 4w.jpg]

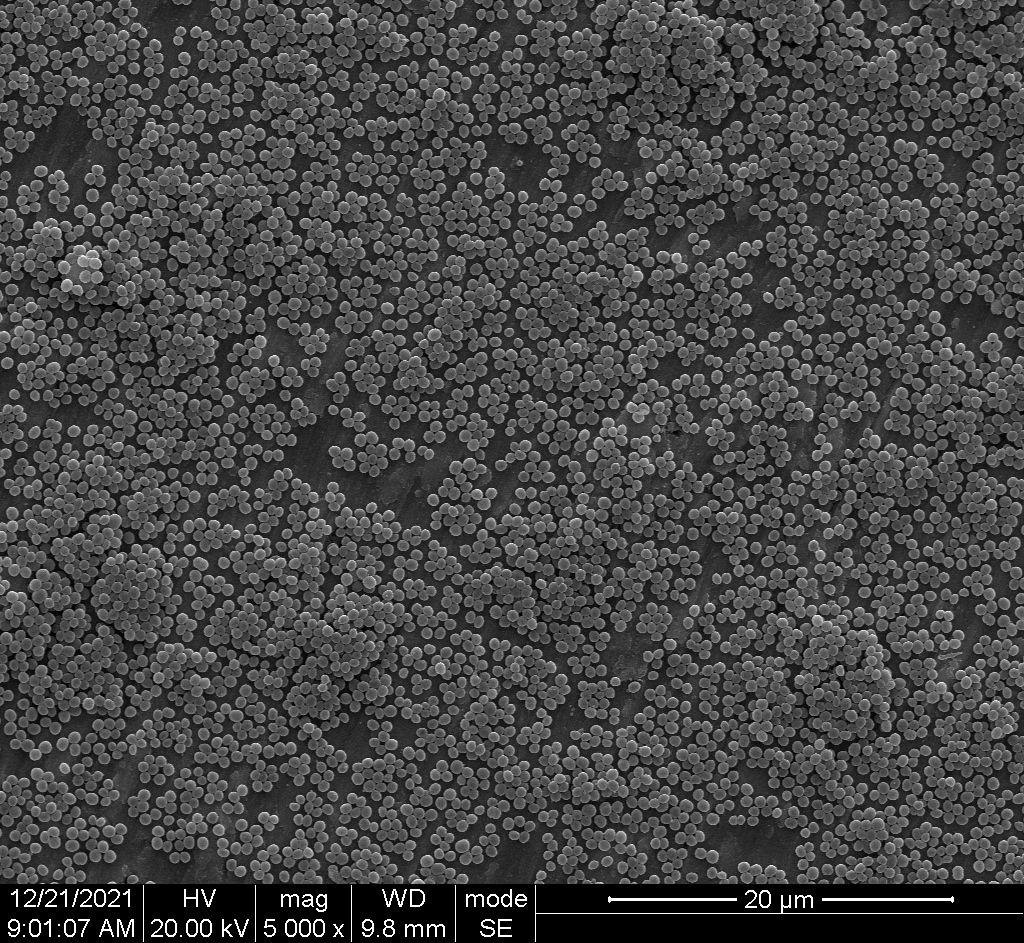

Supplement: Supplementary file 3 [file DataSheet1.ZIP › Antibacterial activity/SEM observation/S. aureus/Ti 5k.jpg]

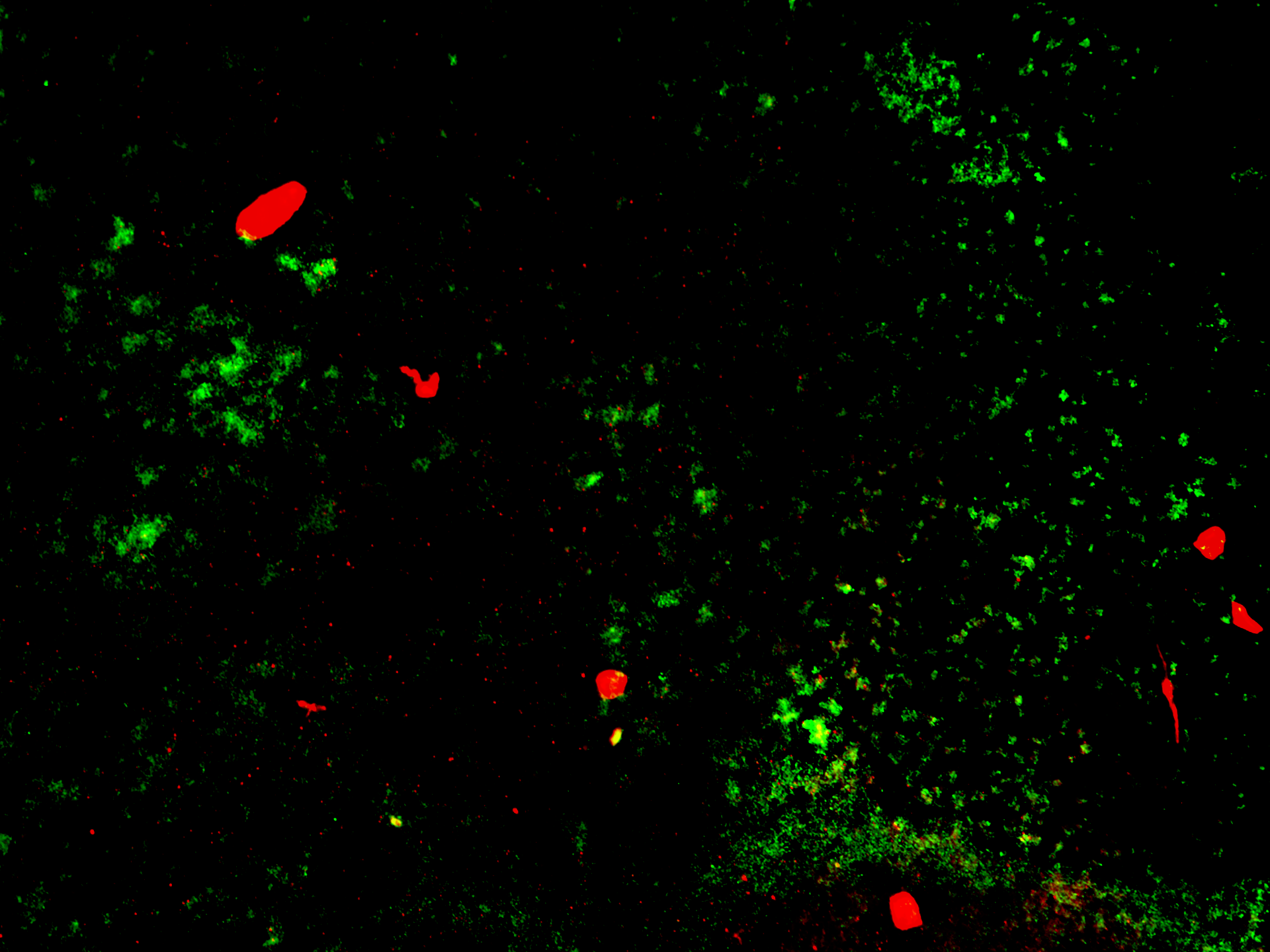

Supplement: Supplementary file 4 [file DataSheet6.ZIP › live dead(bacteria)/MRSA/M30.tif]

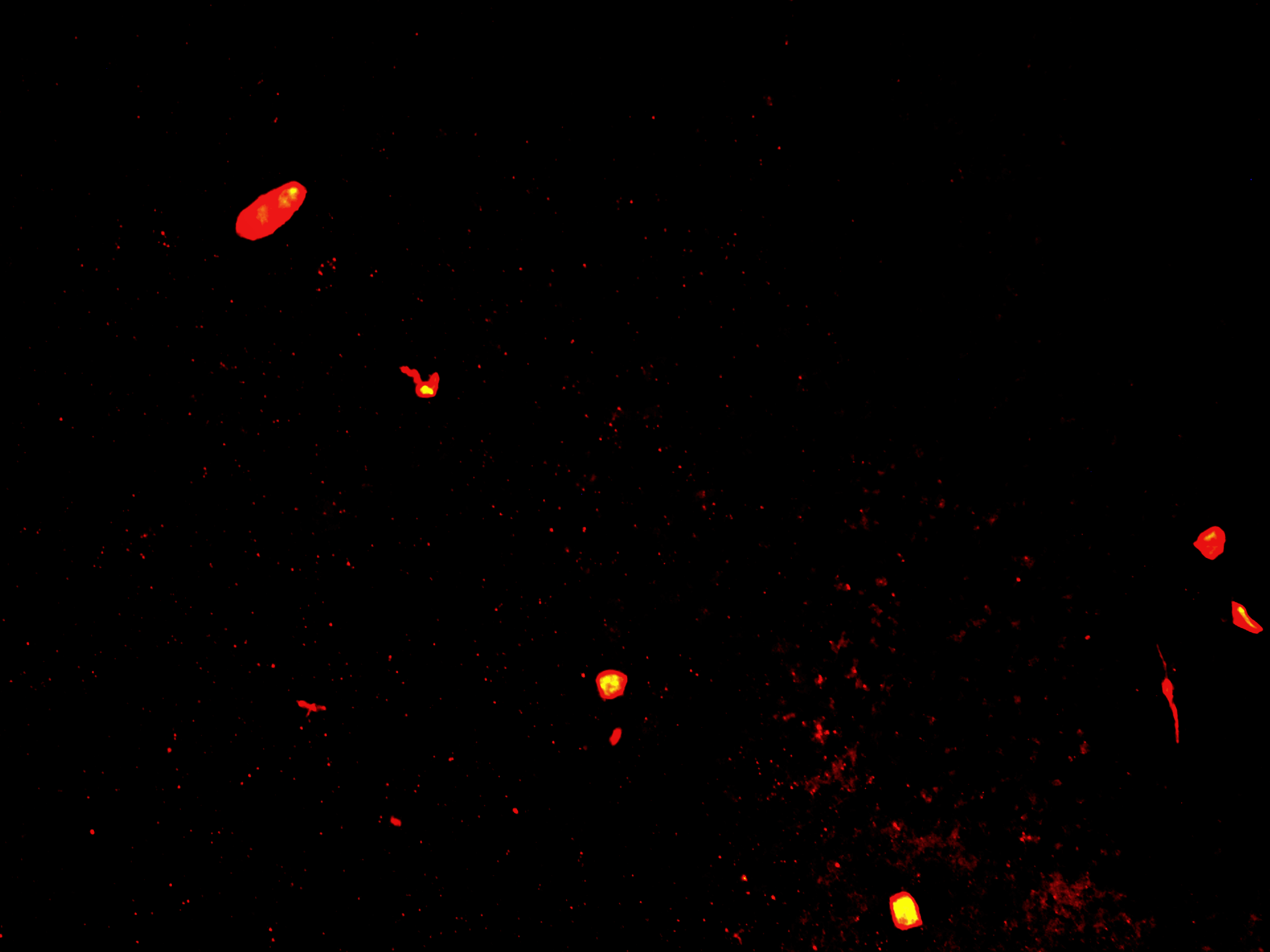

Supplement: Supplementary file 4 [file DataSheet6.ZIP › live dead(bacteria)/MRSA/M30D.tif]

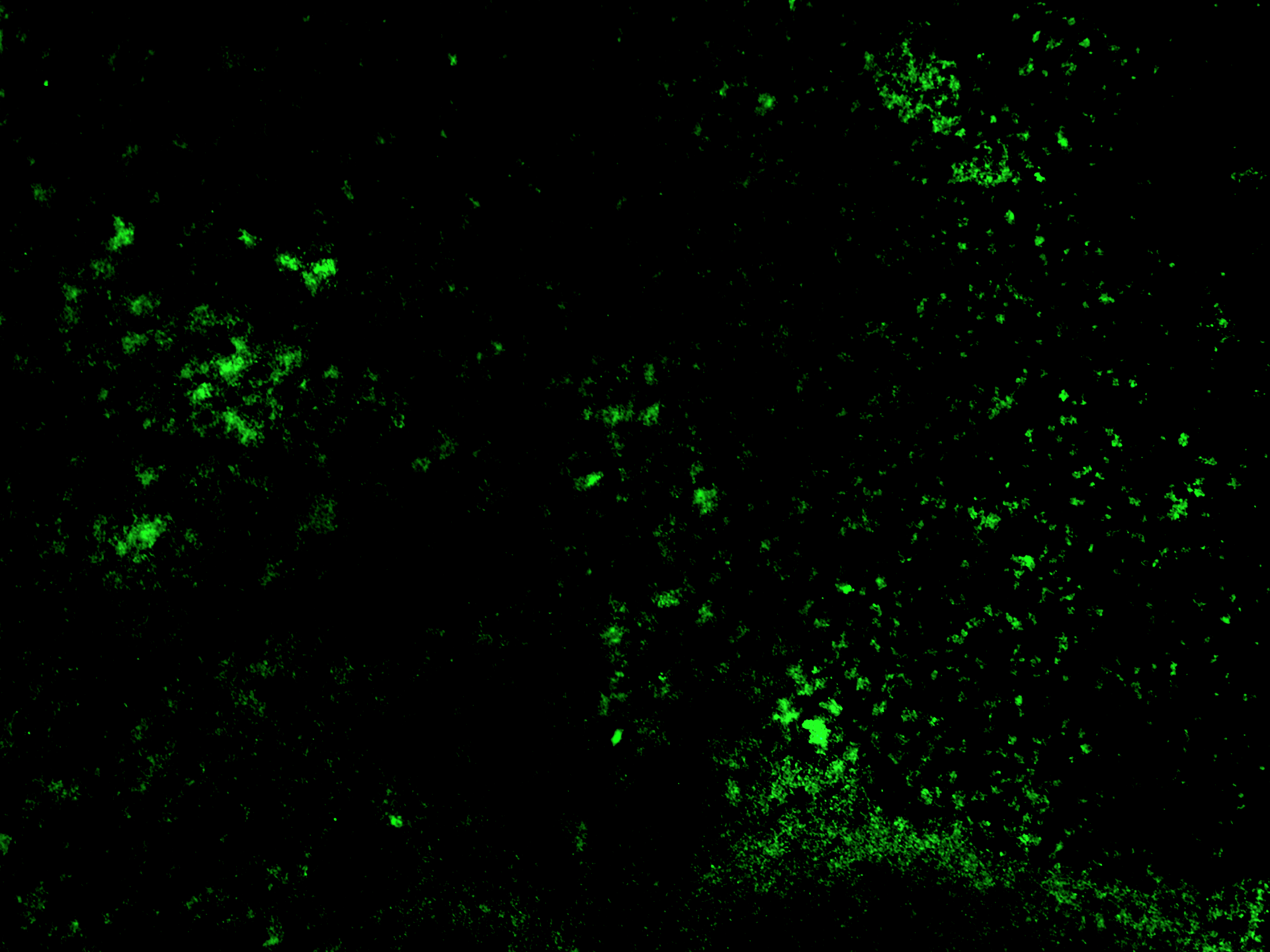

Supplement: Supplementary file 4 [file DataSheet6.ZIP › live dead(bacteria)/MRSA/M30L.tif]

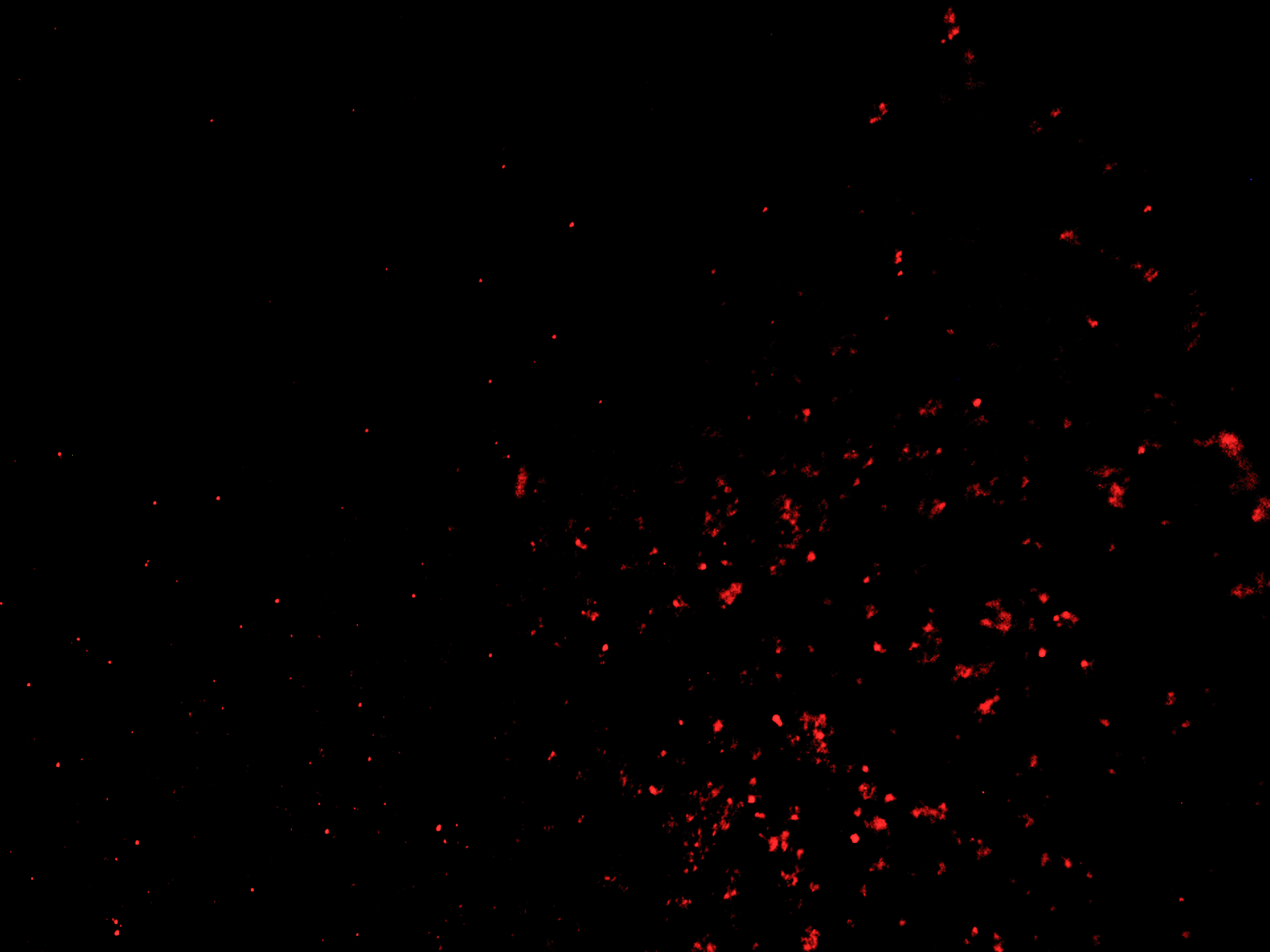

Supplement: Supplementary file 4 [file DataSheet6.ZIP › live dead(bacteria)/MRSA/M40 D.tif]

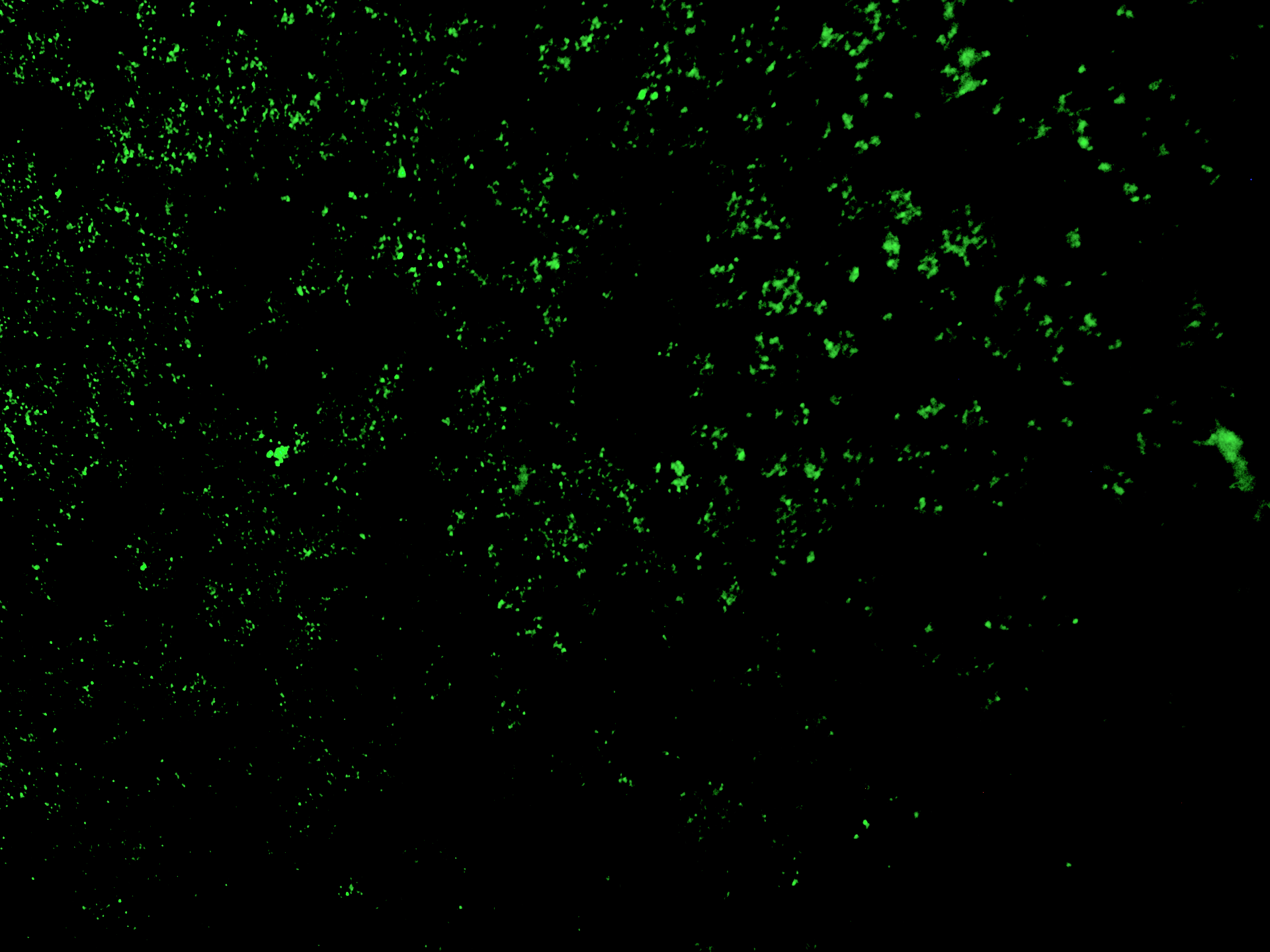

Supplement: Supplementary file 4 [file DataSheet6.ZIP › live dead(bacteria)/MRSA/M40 L.tif]

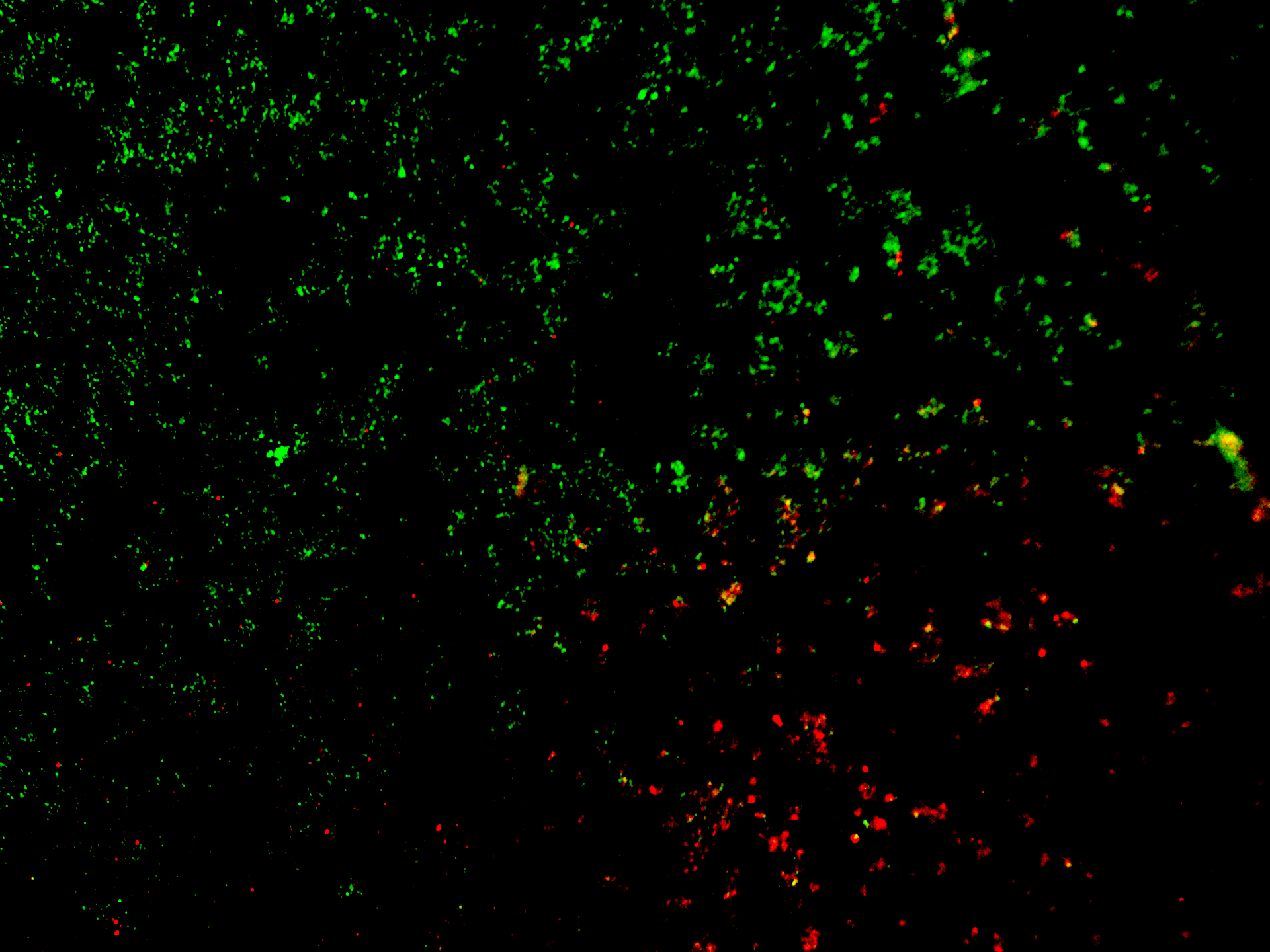

Supplement: Supplementary file 4 [file DataSheet6.ZIP › live dead(bacteria)/MRSA/M40.tif]

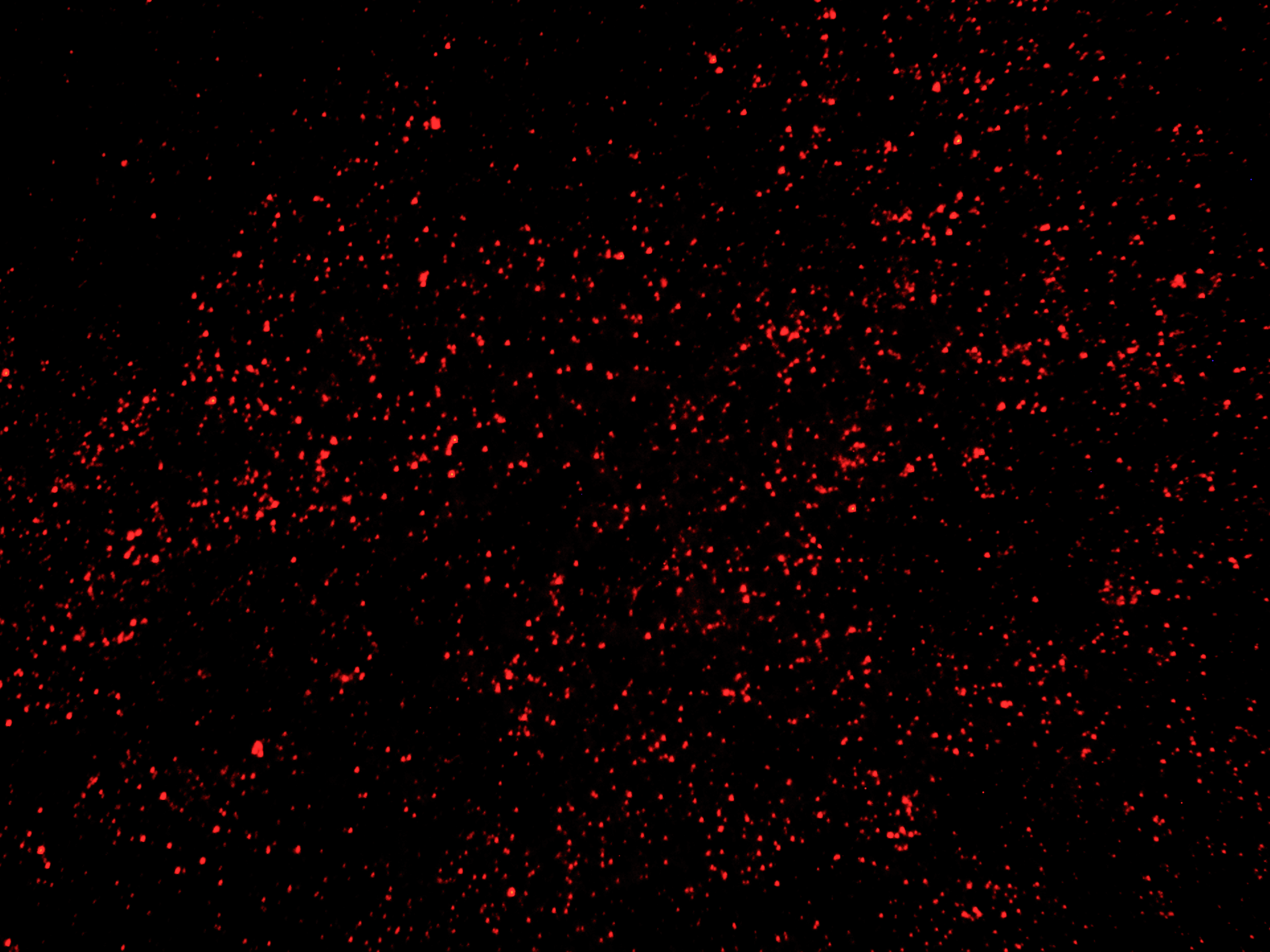

Supplement: Supplementary file 4 [file DataSheet6.ZIP › live dead(bacteria)/MRSA/M50 D.tif]

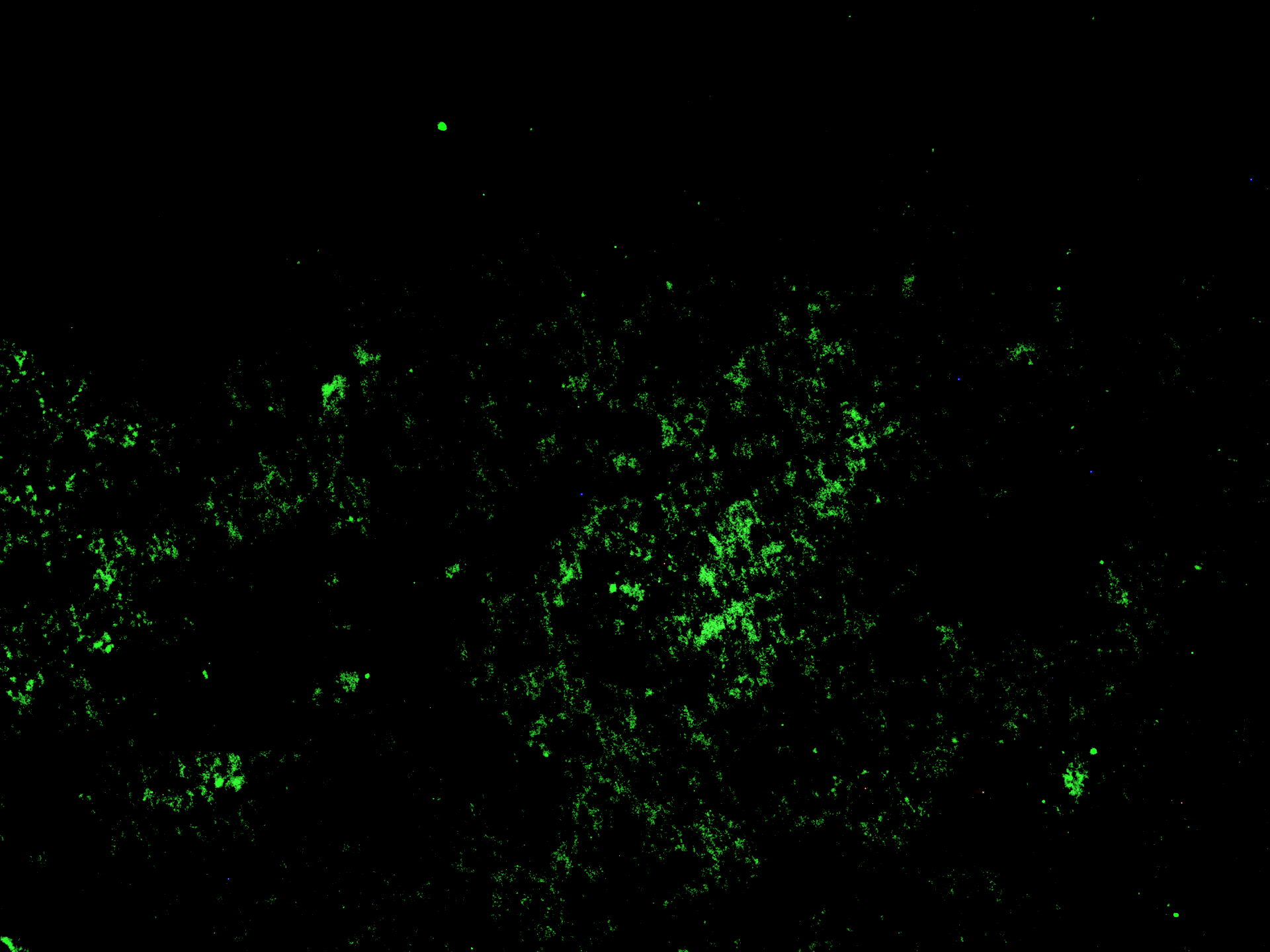

Supplement: Supplementary file 4 [file DataSheet6.ZIP › live dead(bacteria)/MRSA/M50 L.tif]
