# Supplementary figures and images for: Electrophoretic-deposited MXene titanium coatings in regulating bacteria and cell response for peri-implantitis (part 2 of 2)
Source: Front Chem. 2022 Sep 29;10:991481. doi: 10.3389/fchem.2022.991481 (PMC9558740; doi:10.3389/fchem.2022.991481)

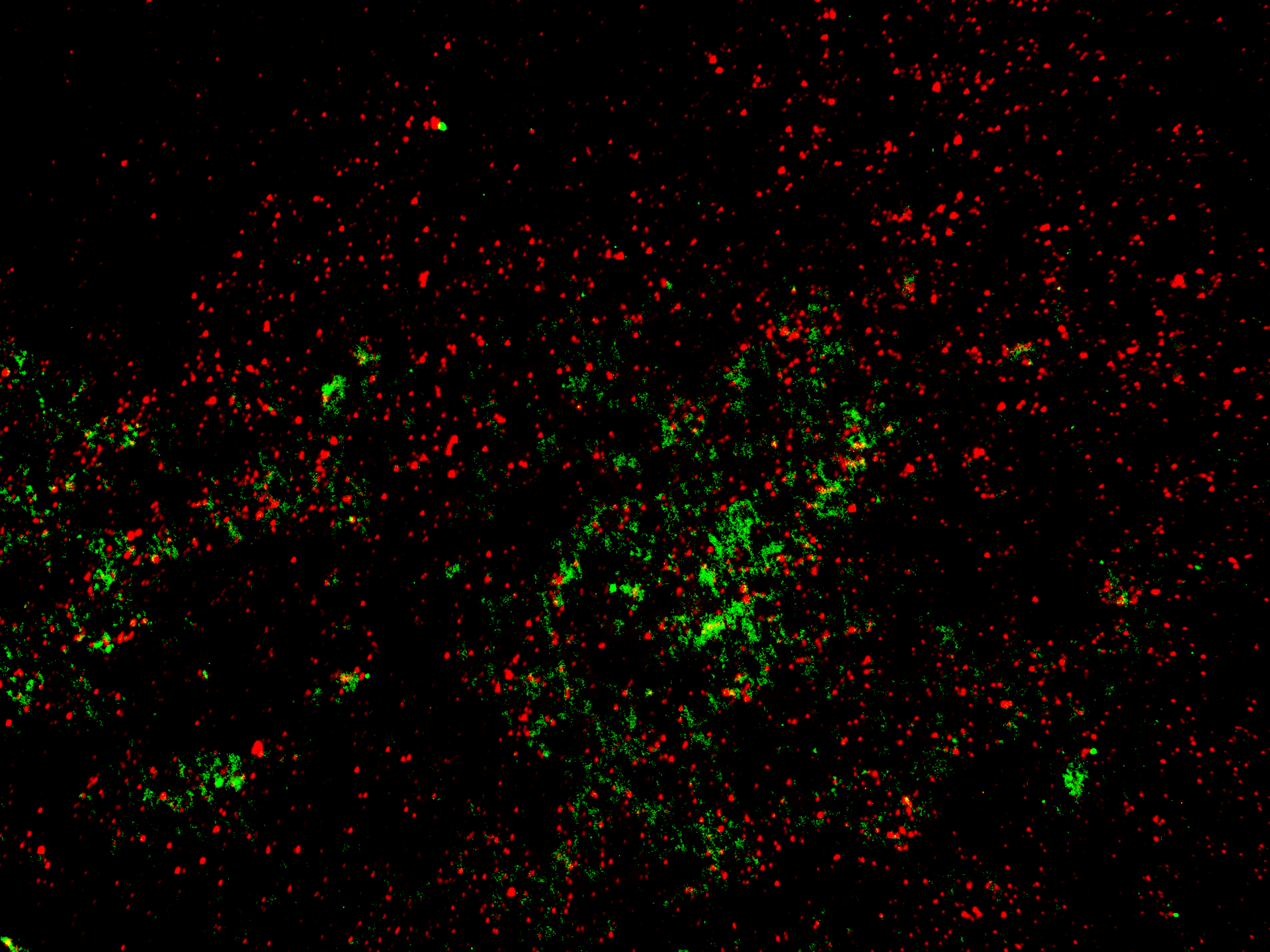

Supplement: Supplementary file 4 [file DataSheet6.ZIP › live dead(bacteria)/MRSA/M50.tif]

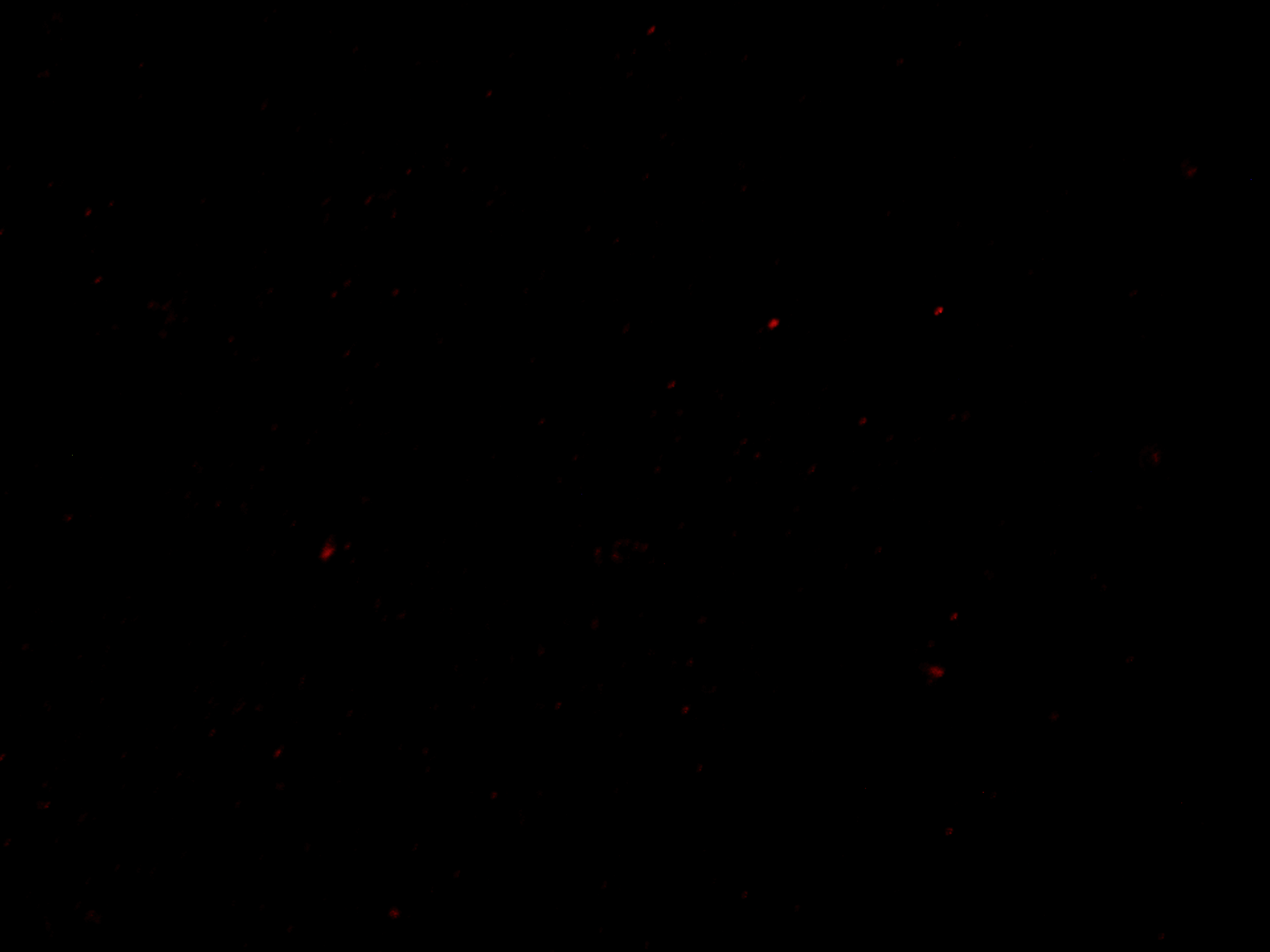

Supplement: Supplementary file 4 [file DataSheet6.ZIP › live dead(bacteria)/MRSA/Ti D.tif]

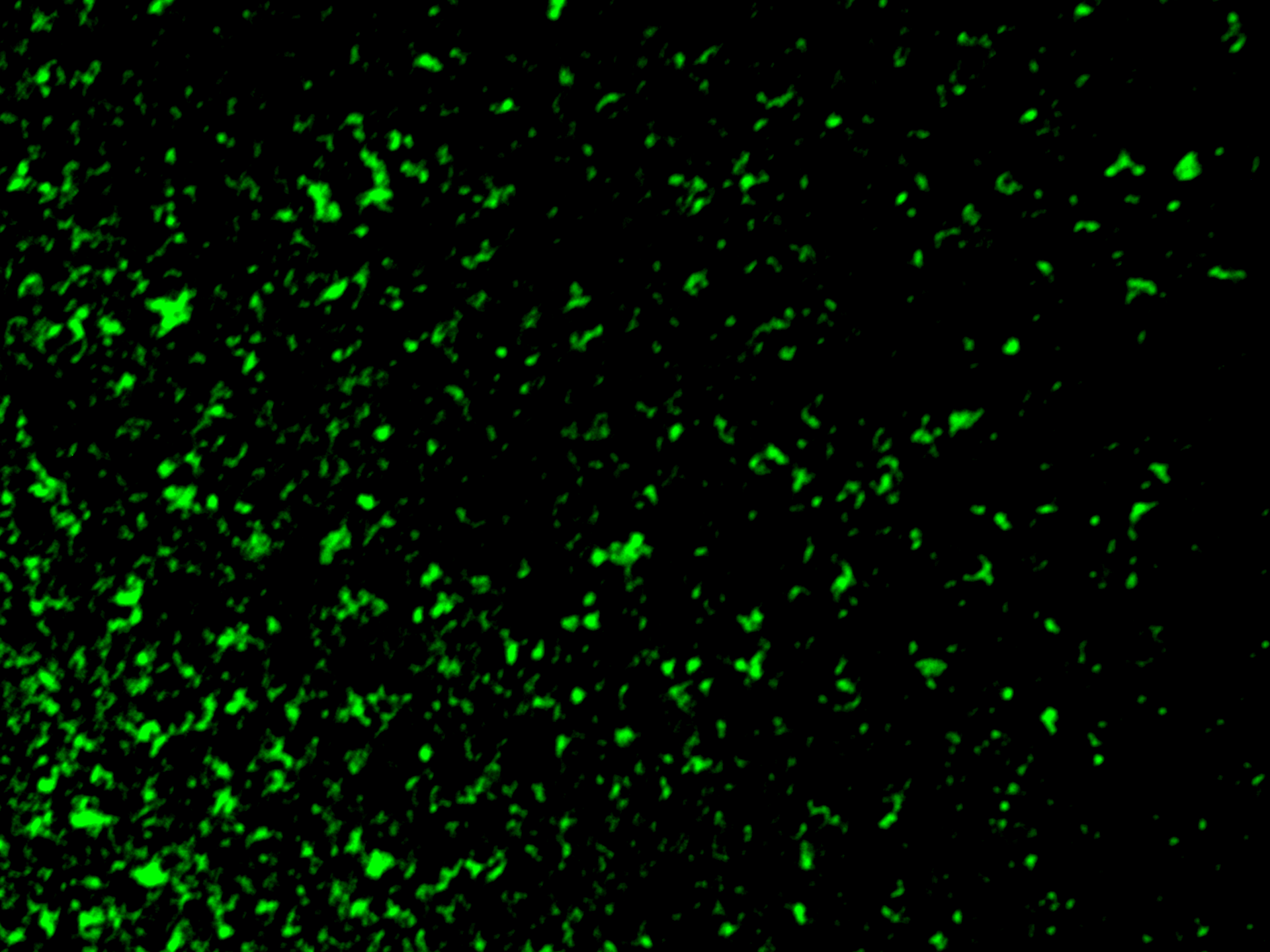

Supplement: Supplementary file 4 [file DataSheet6.ZIP › live dead(bacteria)/MRSA/Ti L.tif]

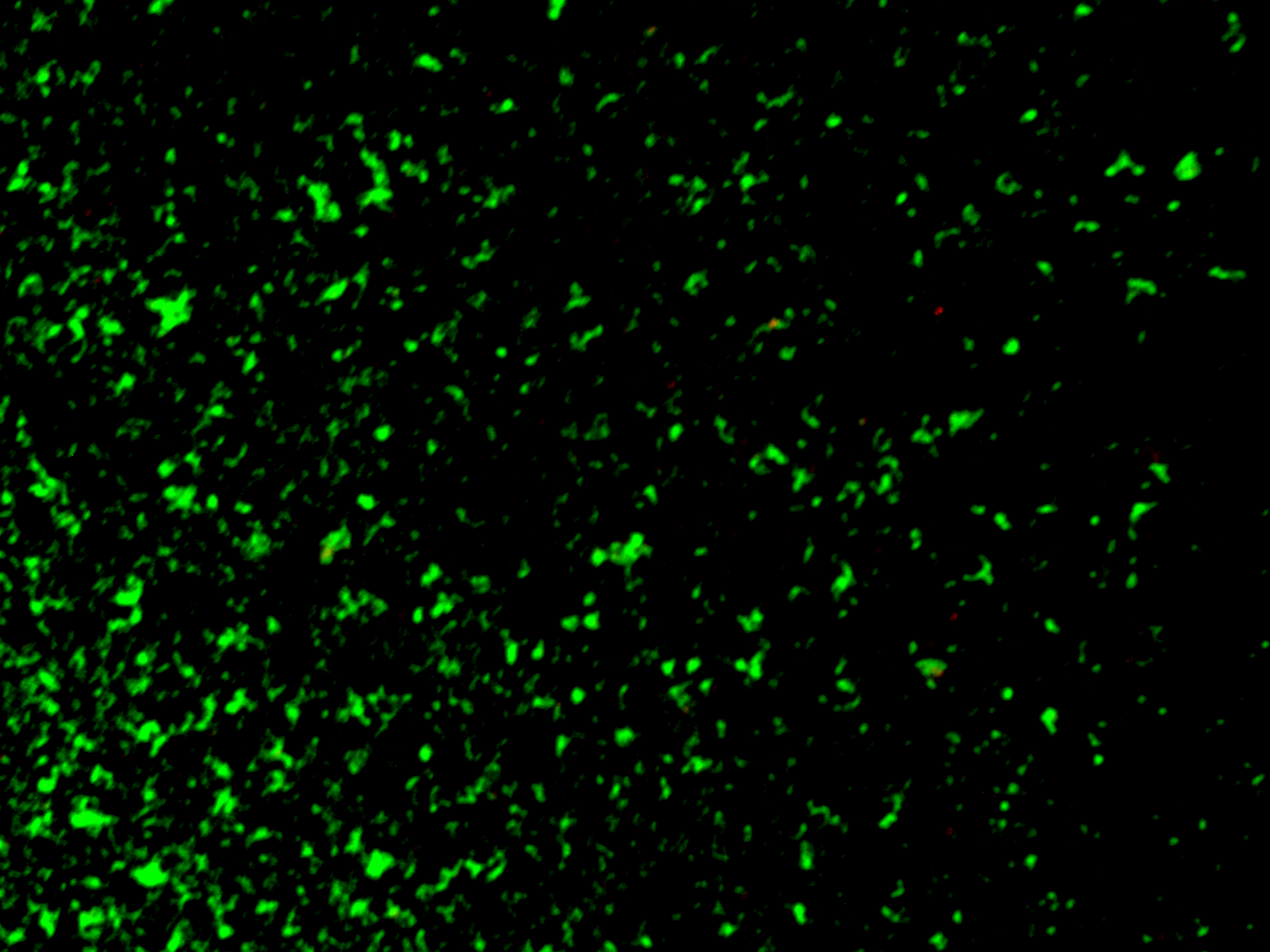

Supplement: Supplementary file 4 [file DataSheet6.ZIP › live dead(bacteria)/MRSA/Ti.tif]

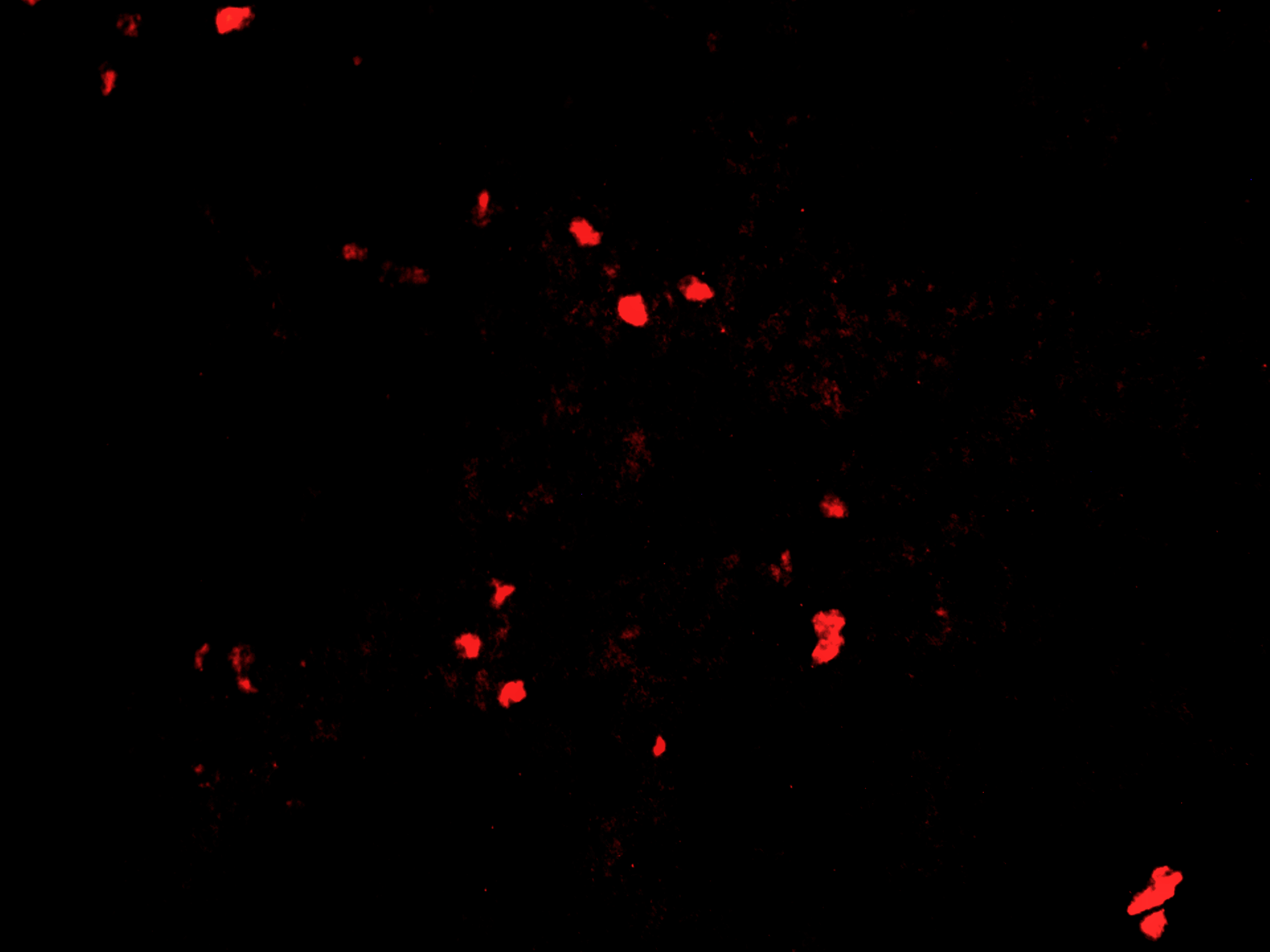

Supplement: Supplementary file 4 [file DataSheet6.ZIP › live dead(bacteria)/S. aureus/M30 D.tif]

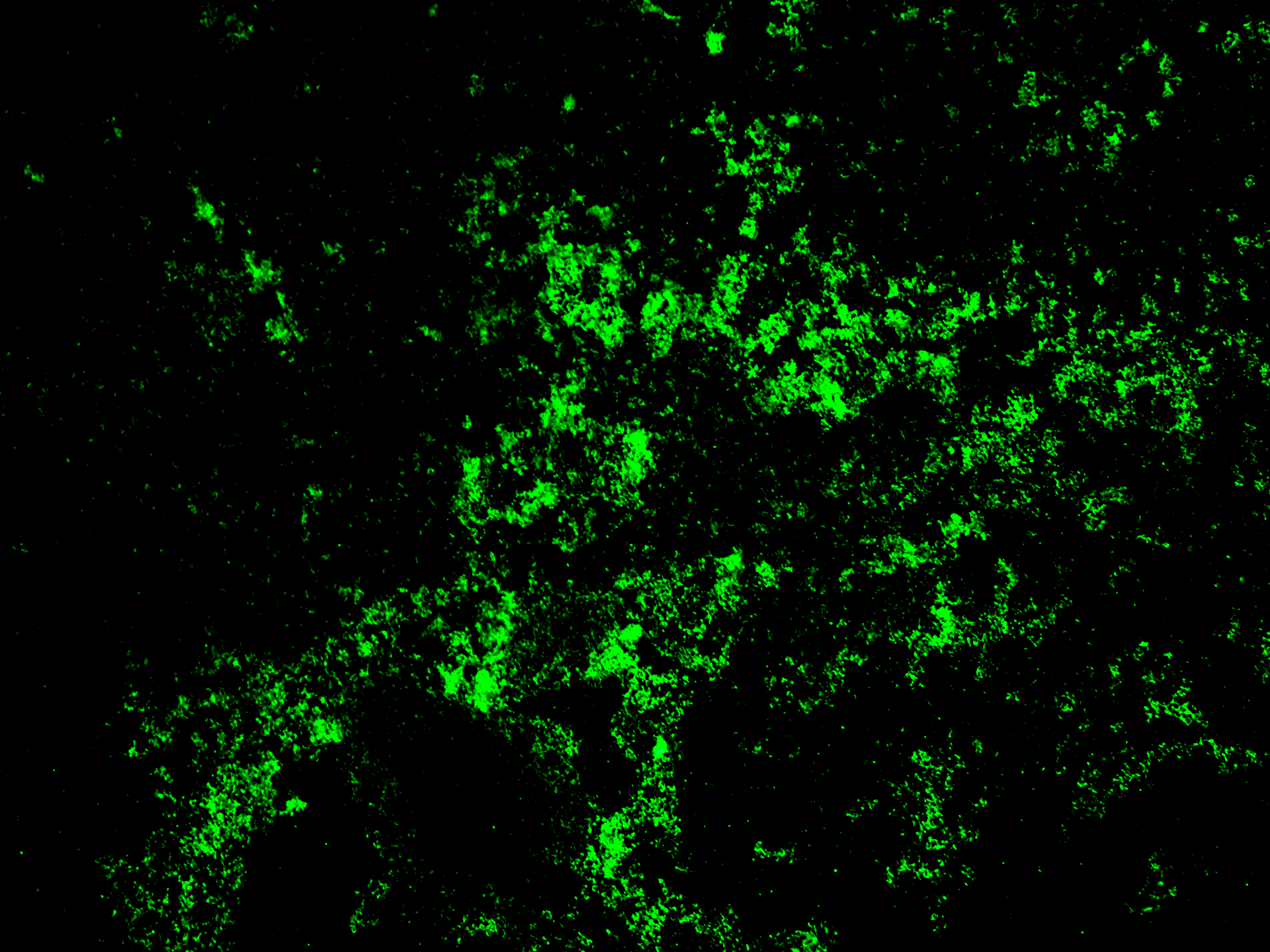

Supplement: Supplementary file 4 [file DataSheet6.ZIP › live dead(bacteria)/S. aureus/M30 L.tif]

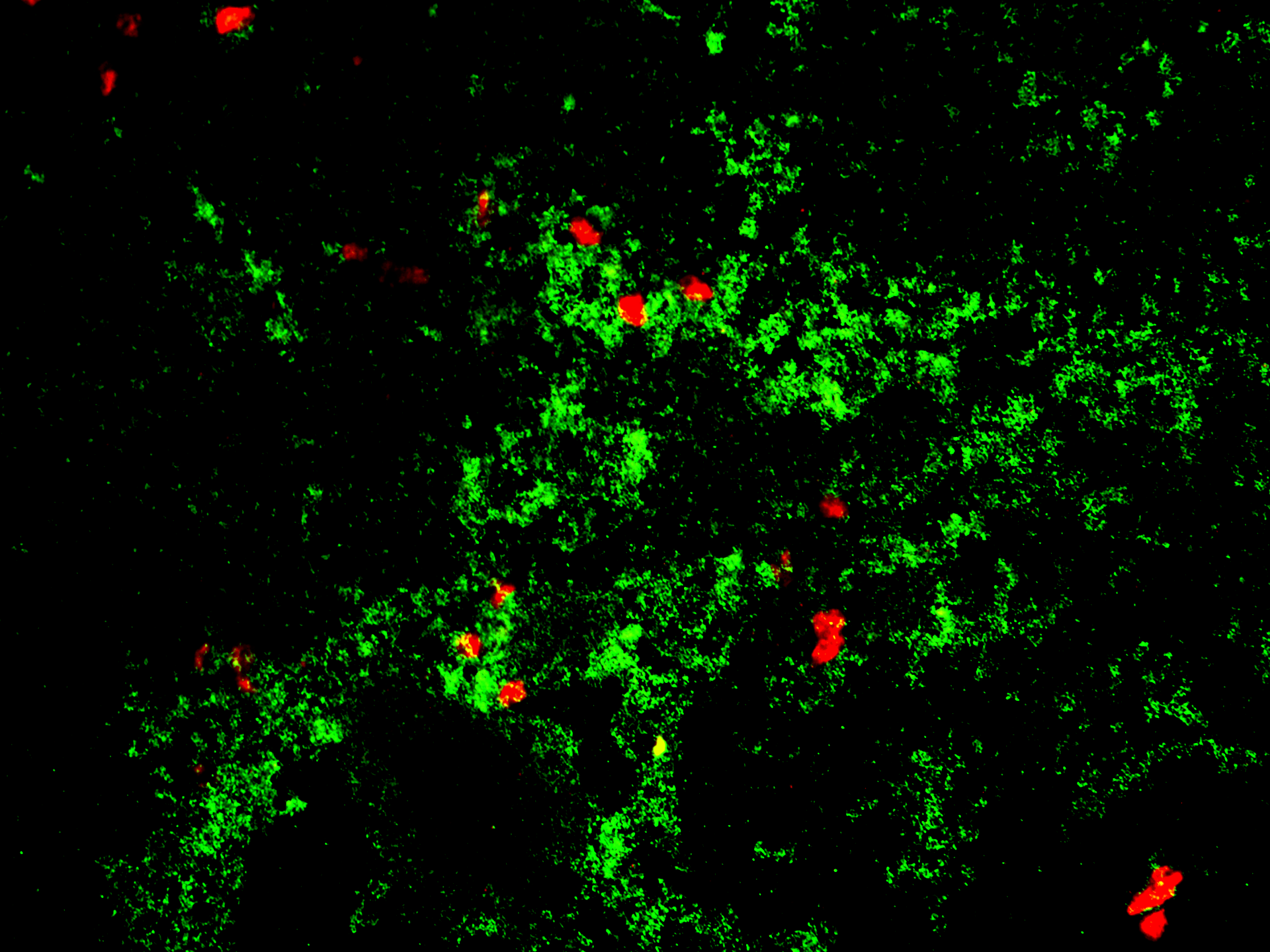

Supplement: Supplementary file 4 [file DataSheet6.ZIP › live dead(bacteria)/S. aureus/M30.tif]

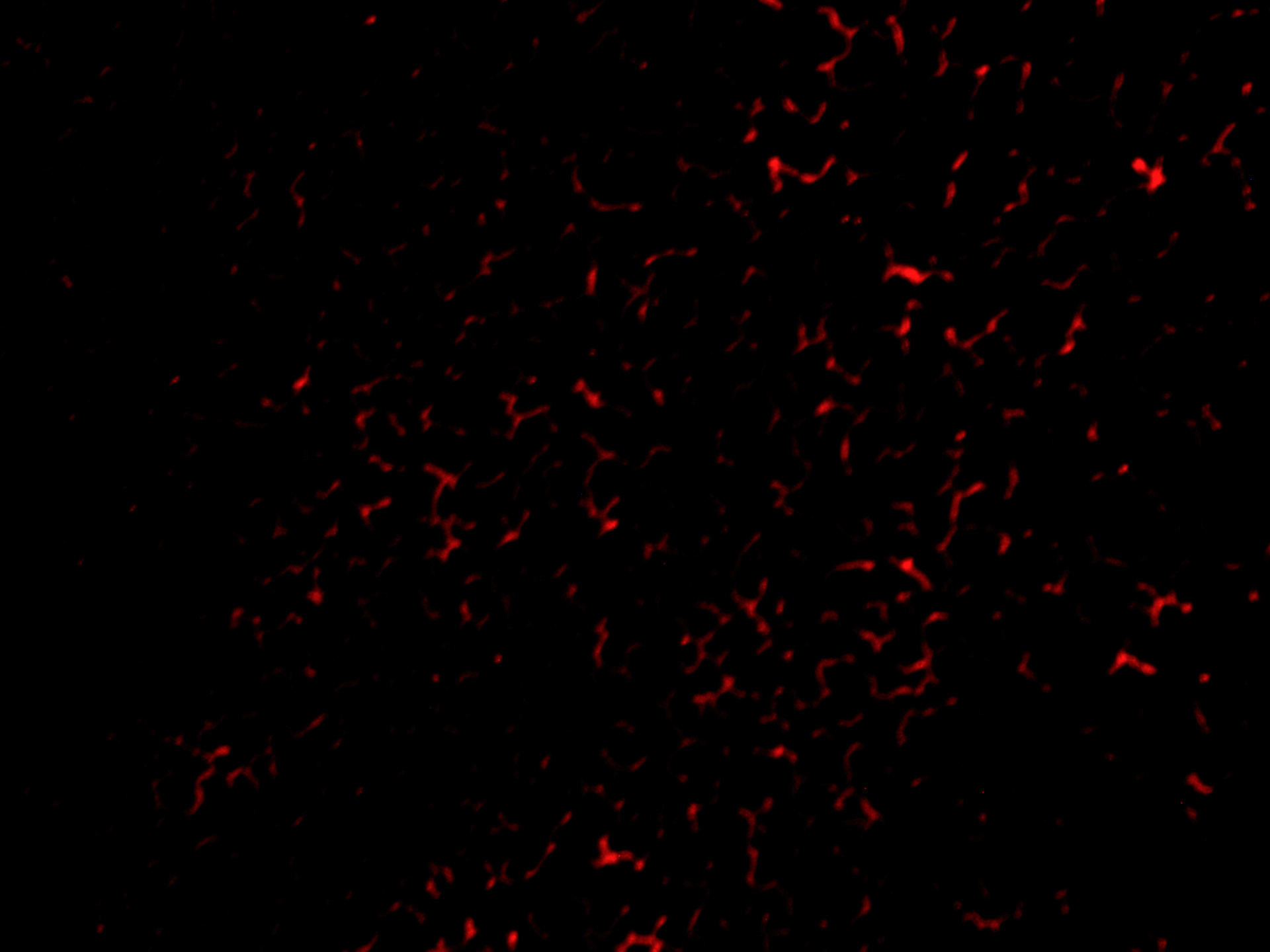

Supplement: Supplementary file 4 [file DataSheet6.ZIP › live dead(bacteria)/S. aureus/M40 D.tif]

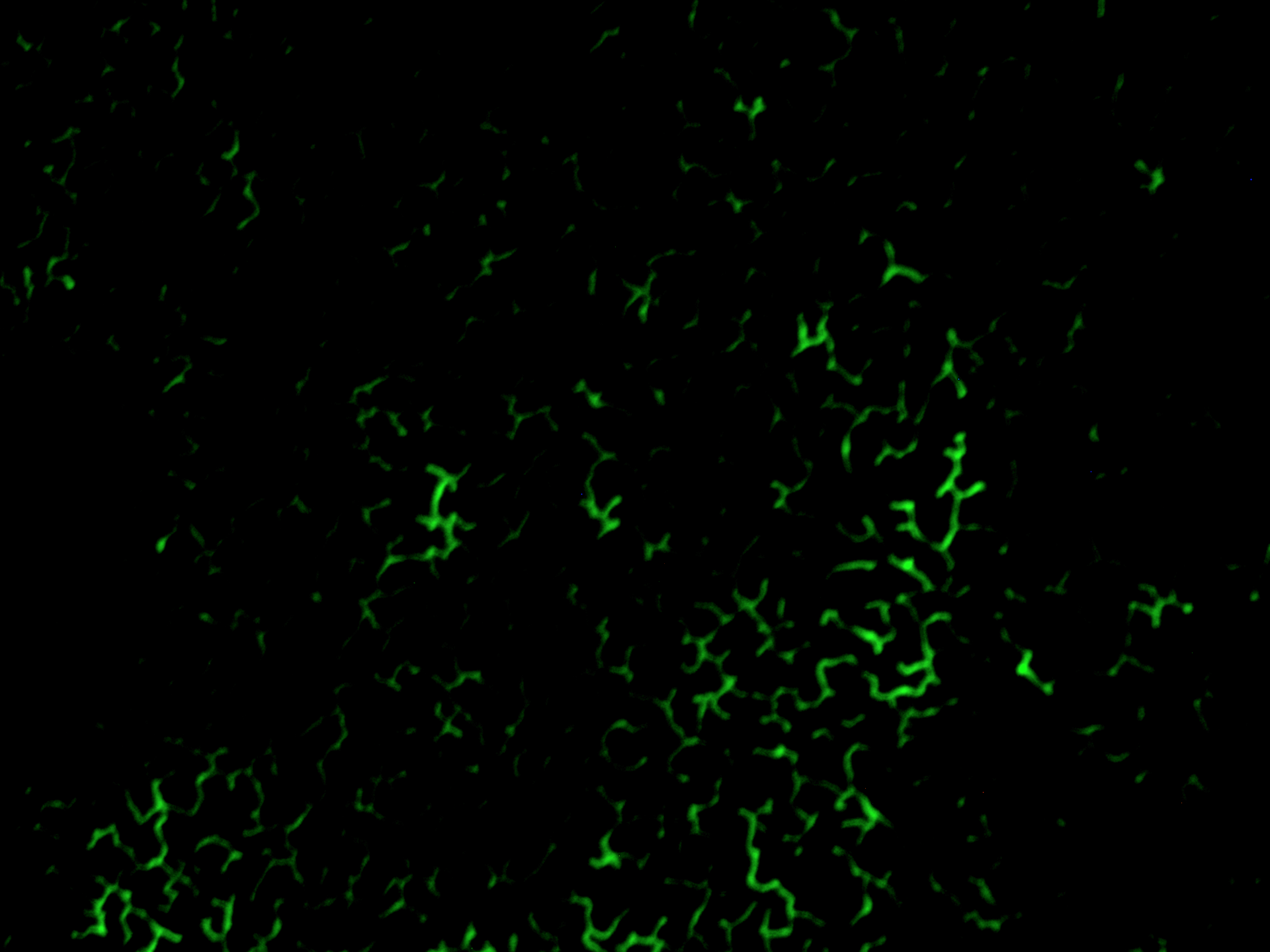

Supplement: Supplementary file 4 [file DataSheet6.ZIP › live dead(bacteria)/S. aureus/M40 L.tif]

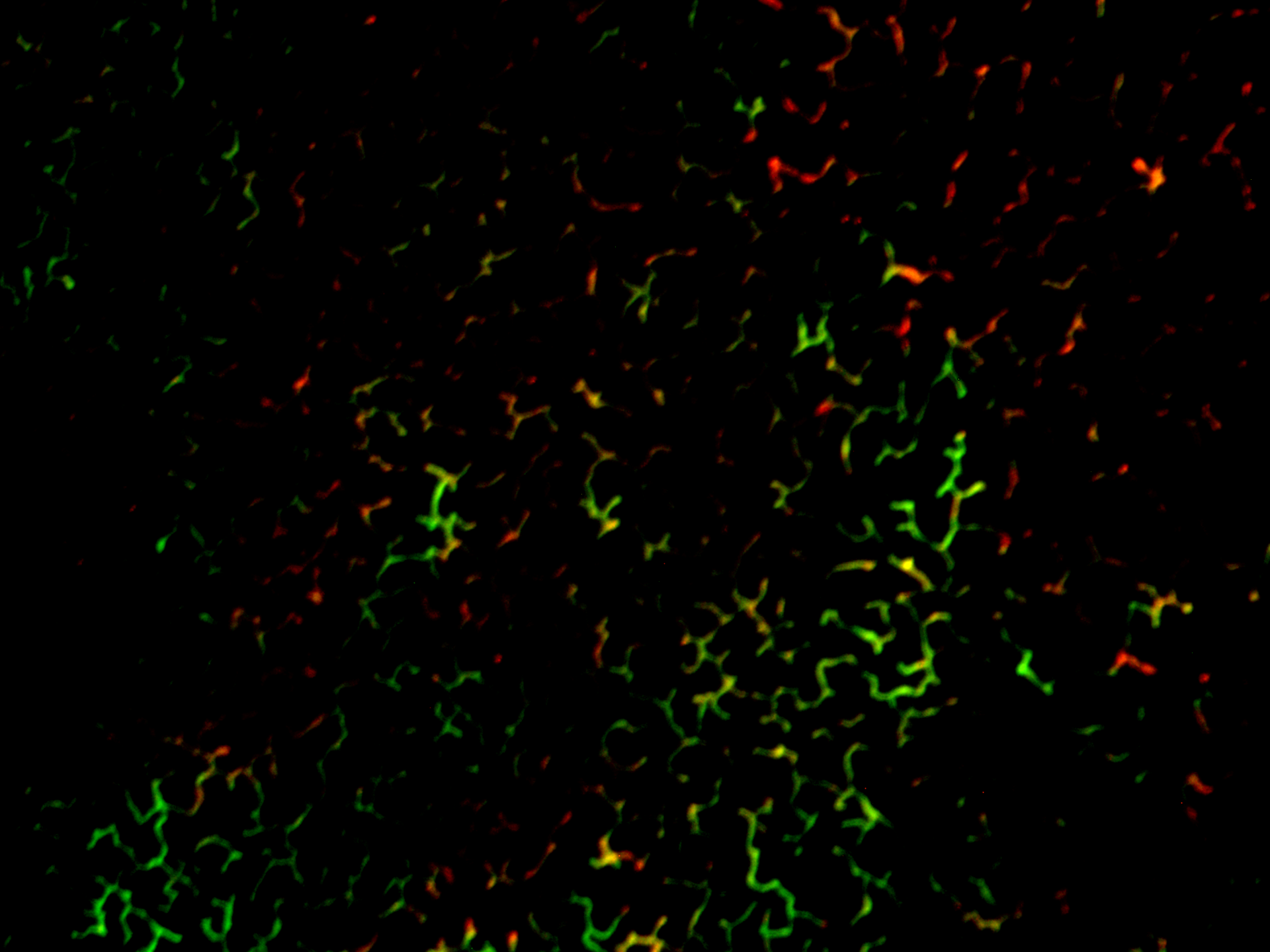

Supplement: Supplementary file 4 [file DataSheet6.ZIP › live dead(bacteria)/S. aureus/M40.tif]

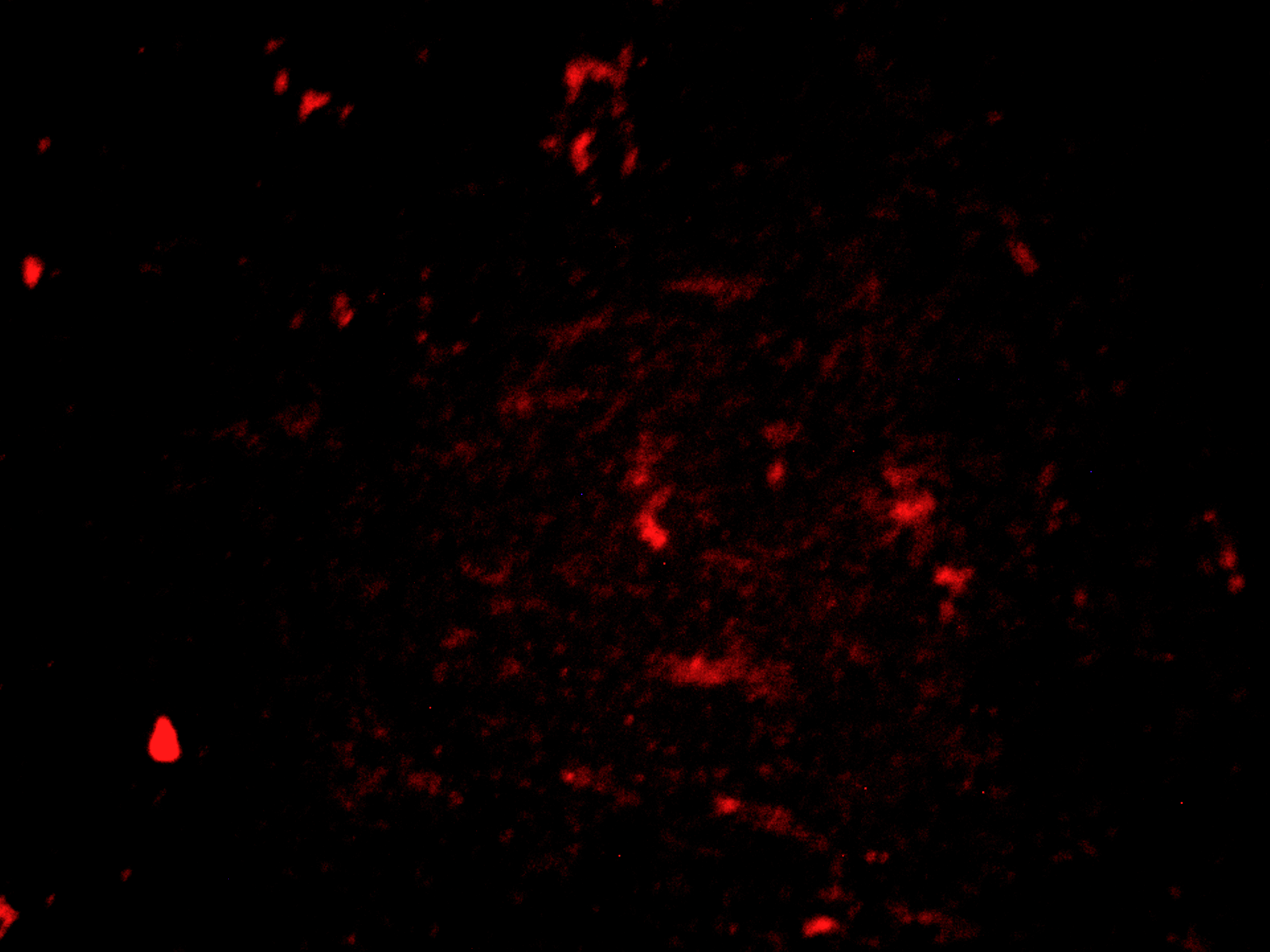

Supplement: Supplementary file 4 [file DataSheet6.ZIP › live dead(bacteria)/S. aureus/M50 D.tif]

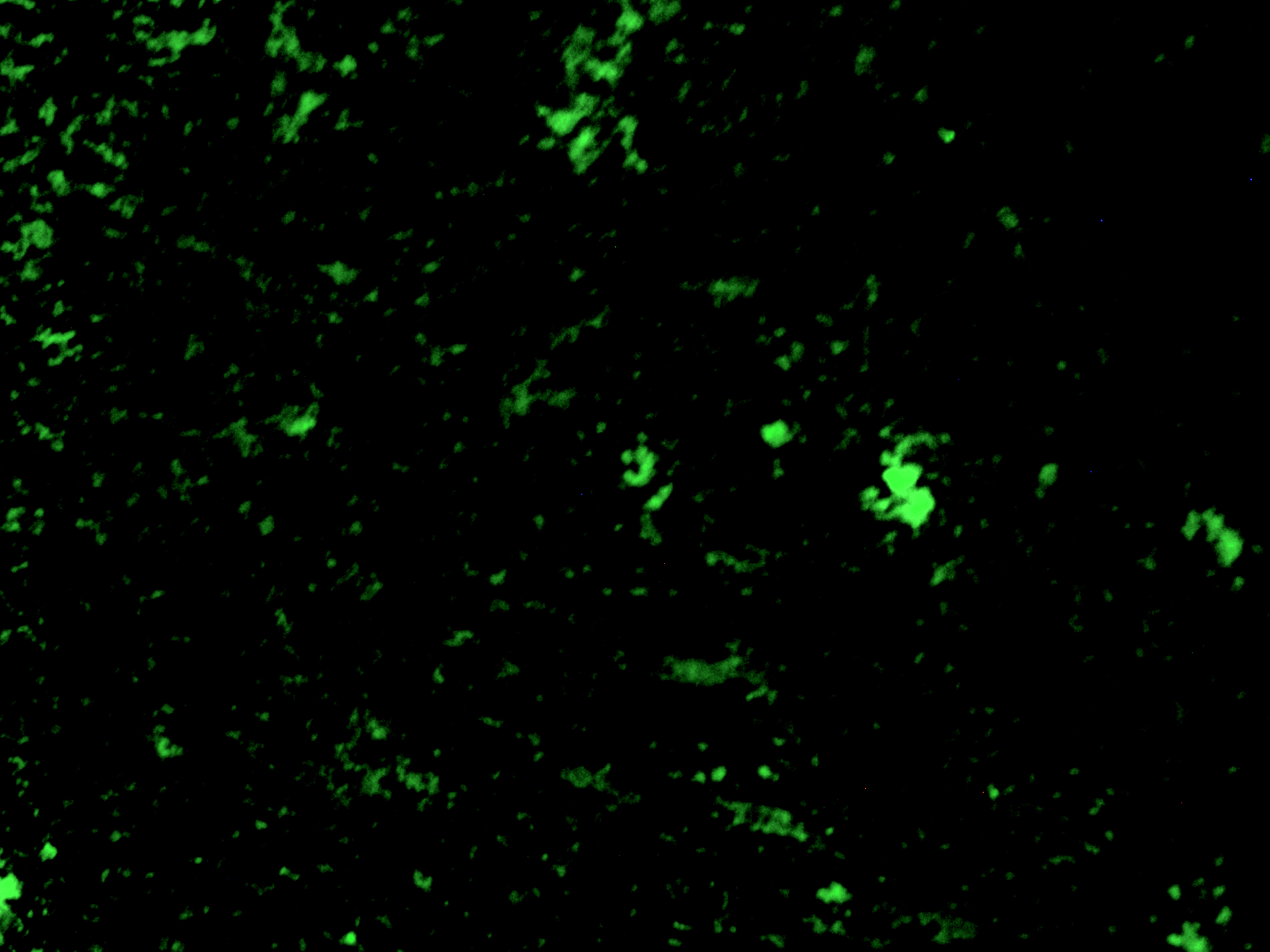

Supplement: Supplementary file 4 [file DataSheet6.ZIP › live dead(bacteria)/S. aureus/M50 L.tif]

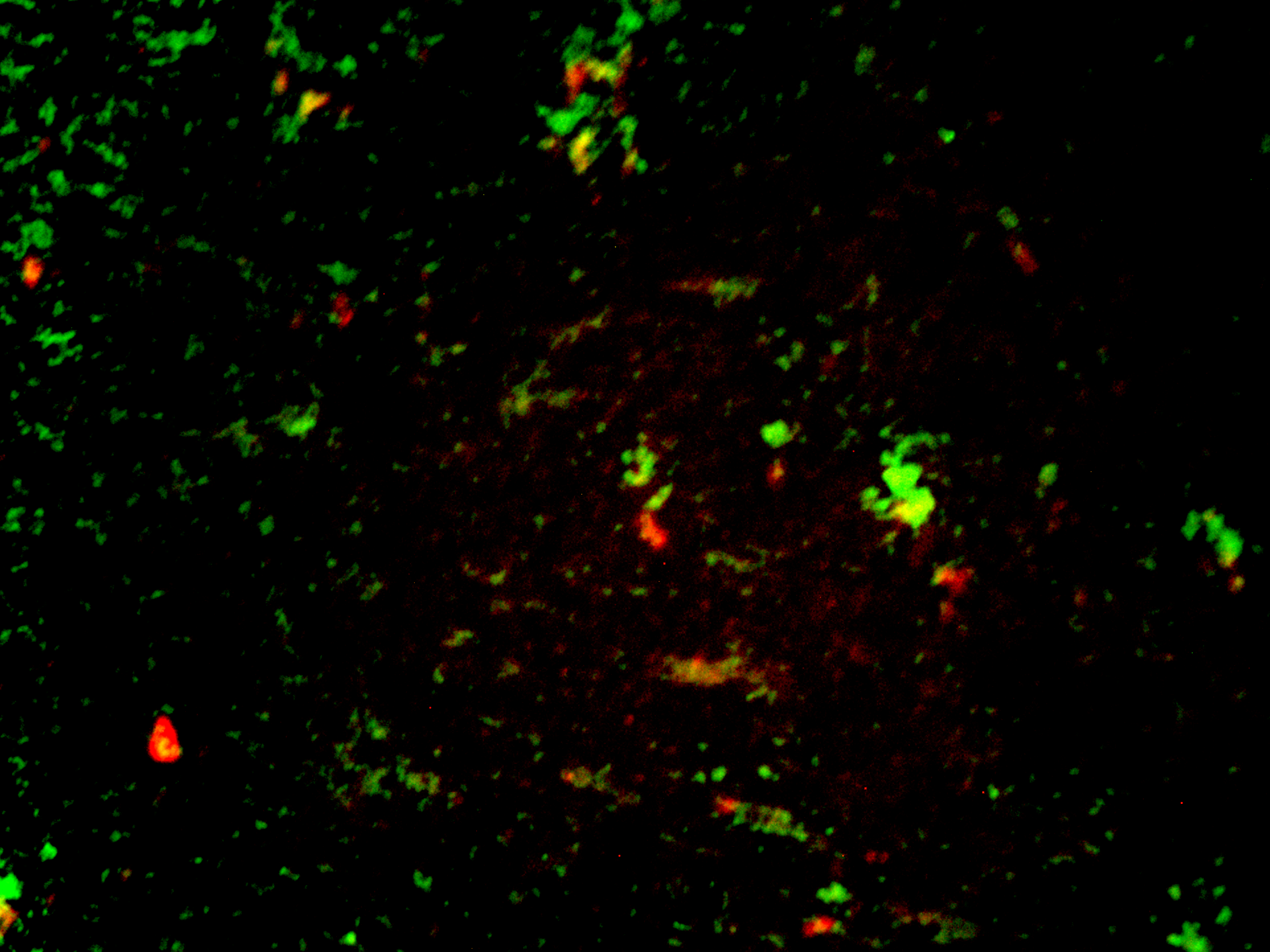

Supplement: Supplementary file 4 [file DataSheet6.ZIP › live dead(bacteria)/S. aureus/M50.tif]

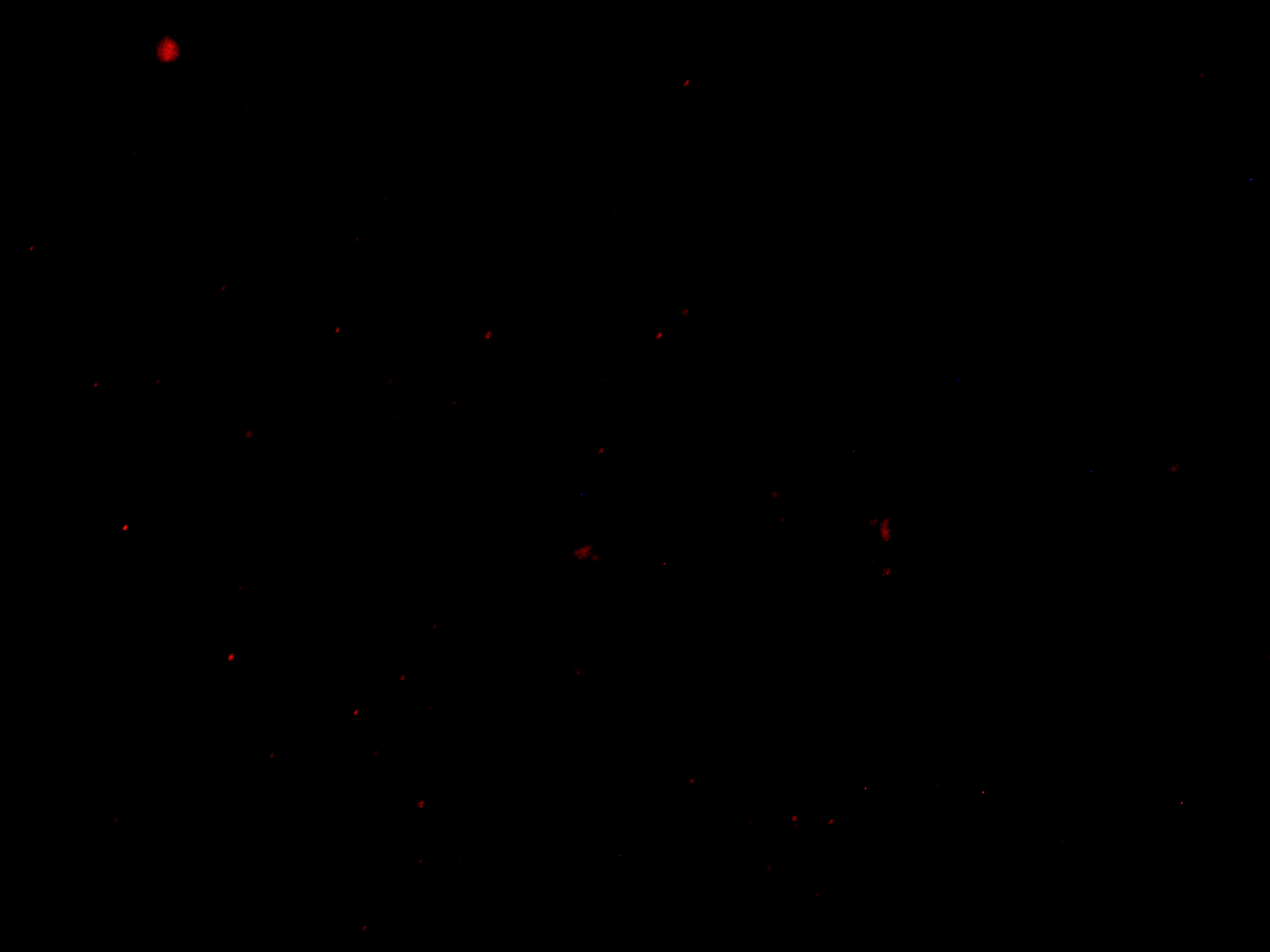

Supplement: Supplementary file 4 [file DataSheet6.ZIP › live dead(bacteria)/S. aureus/Ti D.tif]

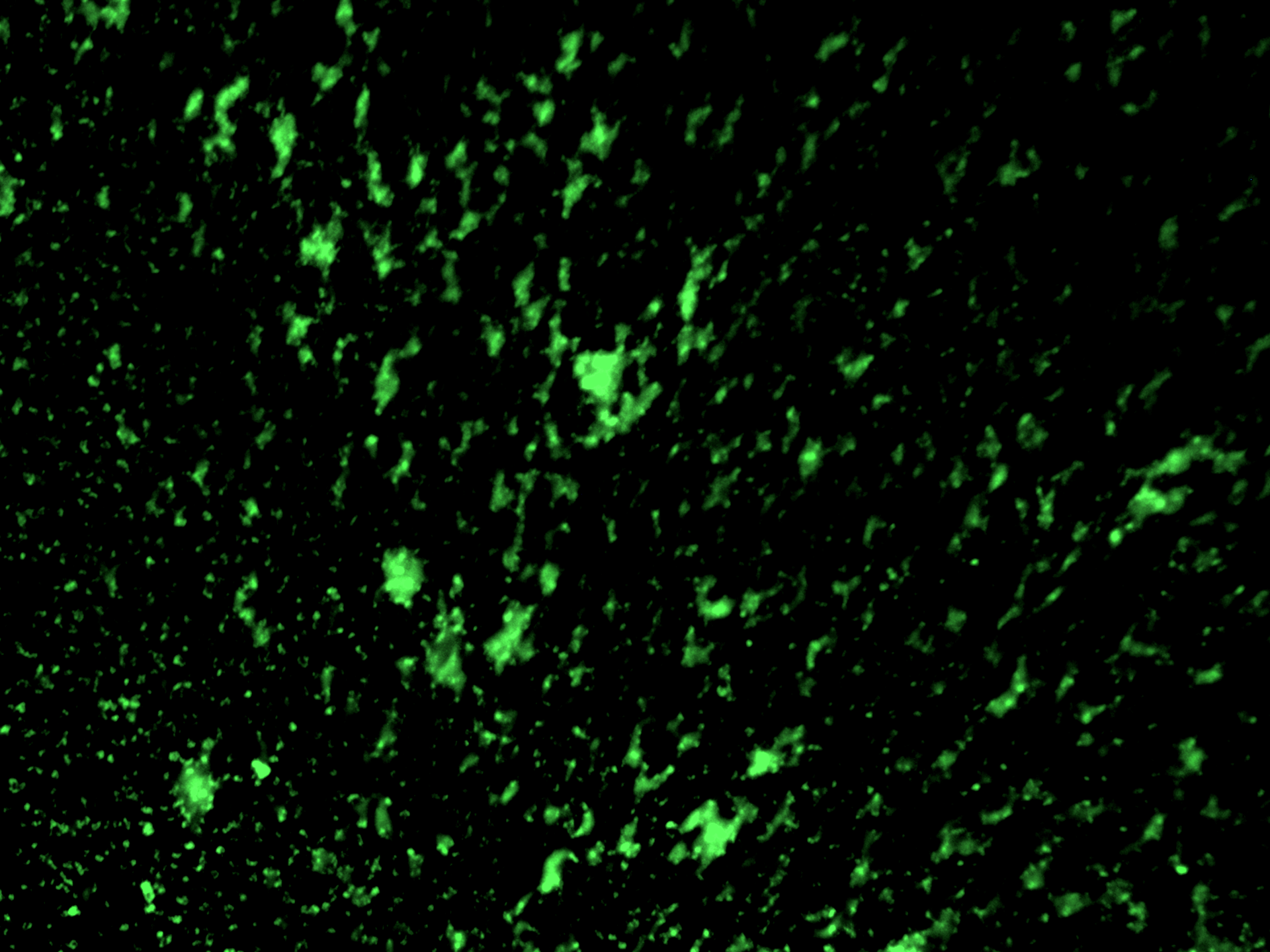

Supplement: Supplementary file 4 [file DataSheet6.ZIP › live dead(bacteria)/S. aureus/Ti L.tif]

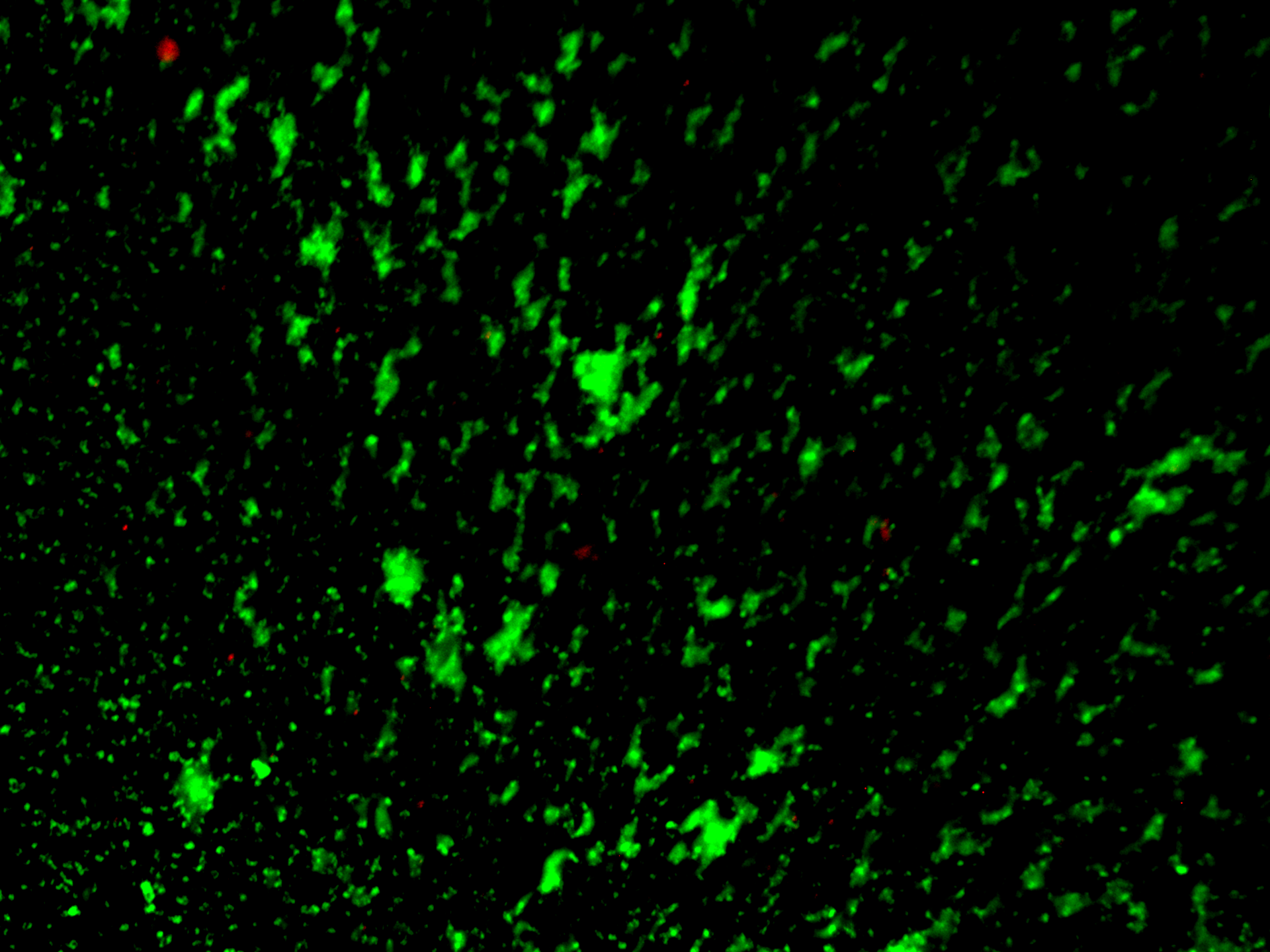

Supplement: Supplementary file 4 [file DataSheet6.ZIP › live dead(bacteria)/S. aureus/Ti.tif]

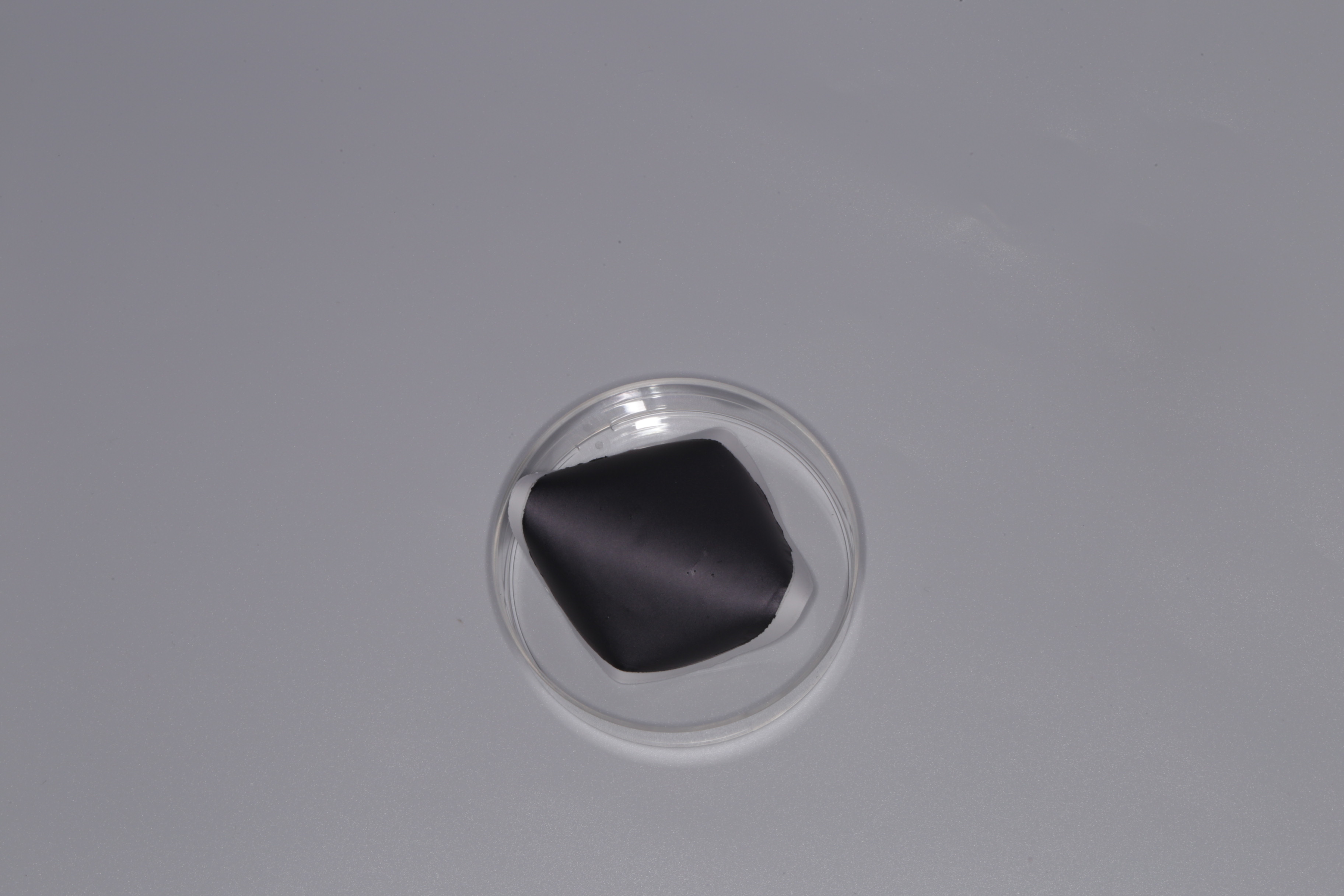

Supplement: Supplementary file 5 [file DataSheet2.ZIP › Physical map/IMG_9290.JPG]

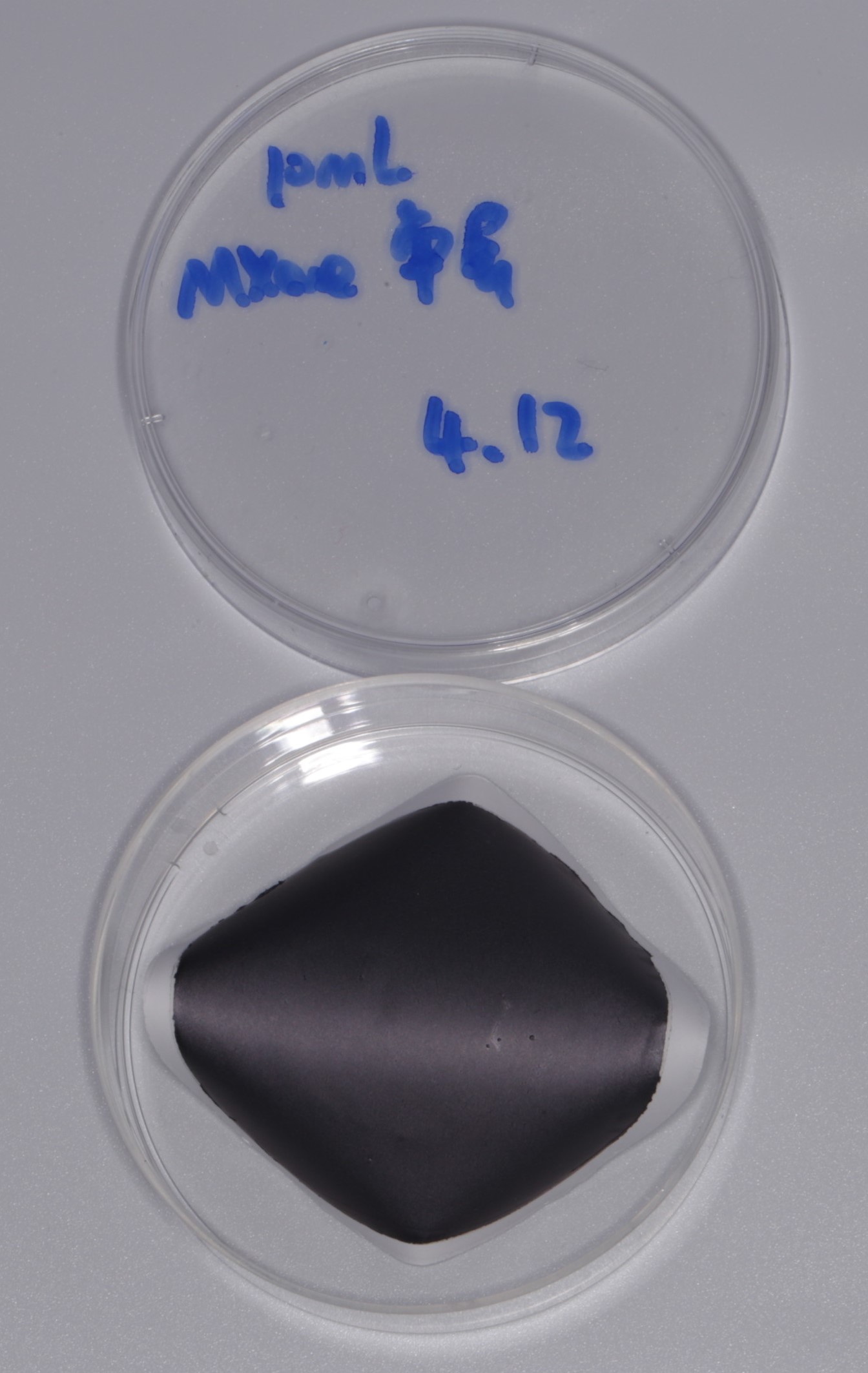

Supplement: Supplementary file 5 [file DataSheet2.ZIP › Physical map/IMG_9291.JPG]

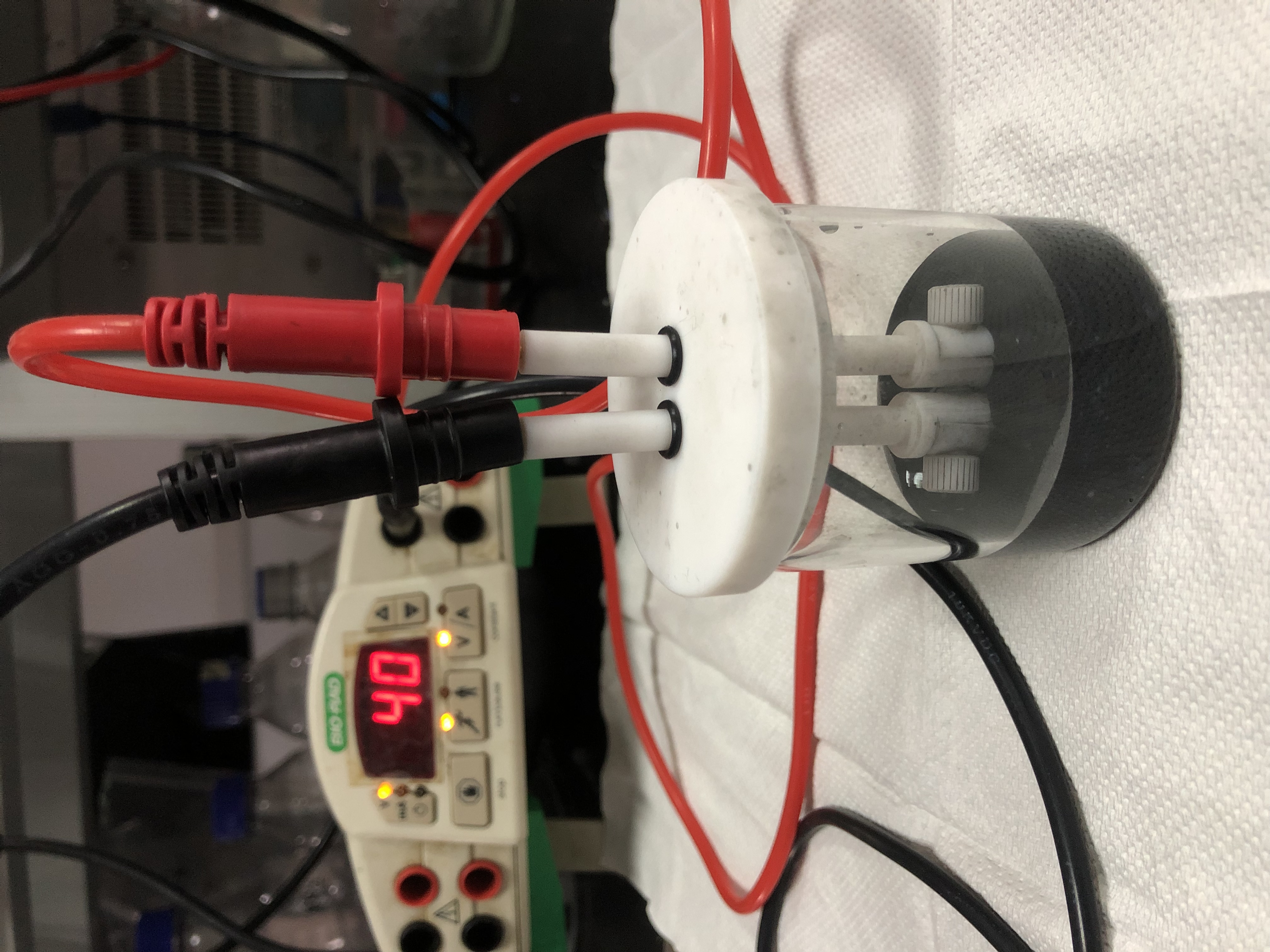

Supplement: Supplementary file 5 [file DataSheet2.ZIP › Physical map/process.jpg]

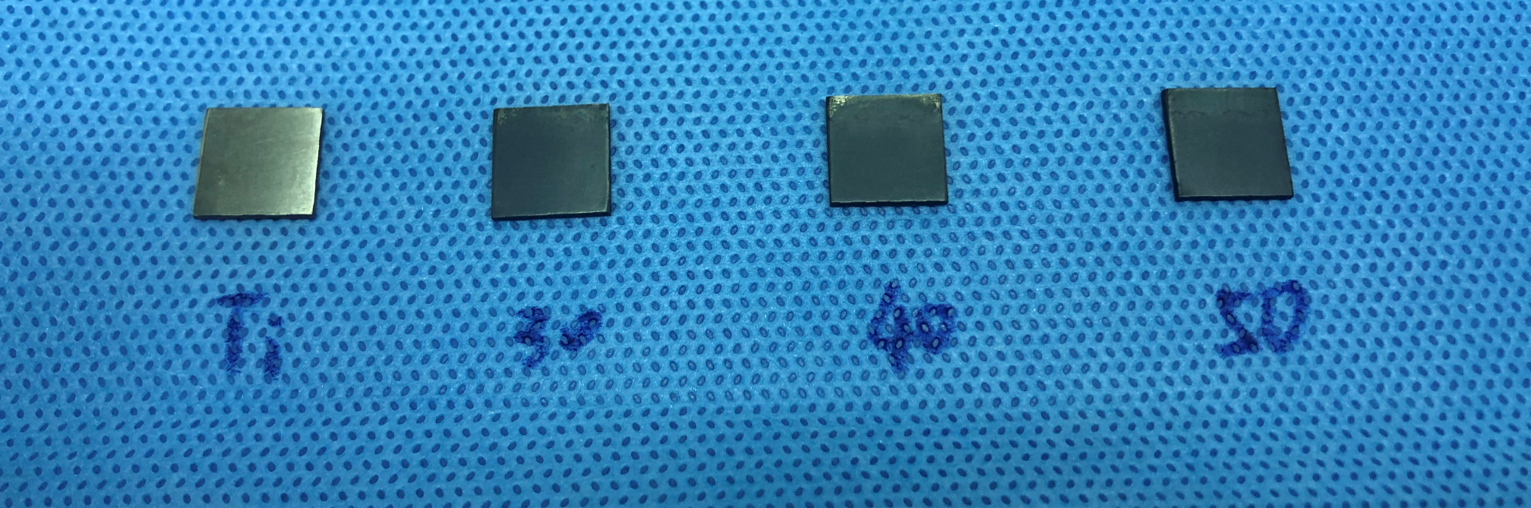

Supplement: Supplementary file 5 [file DataSheet2.ZIP › Physical map/samples.tif]

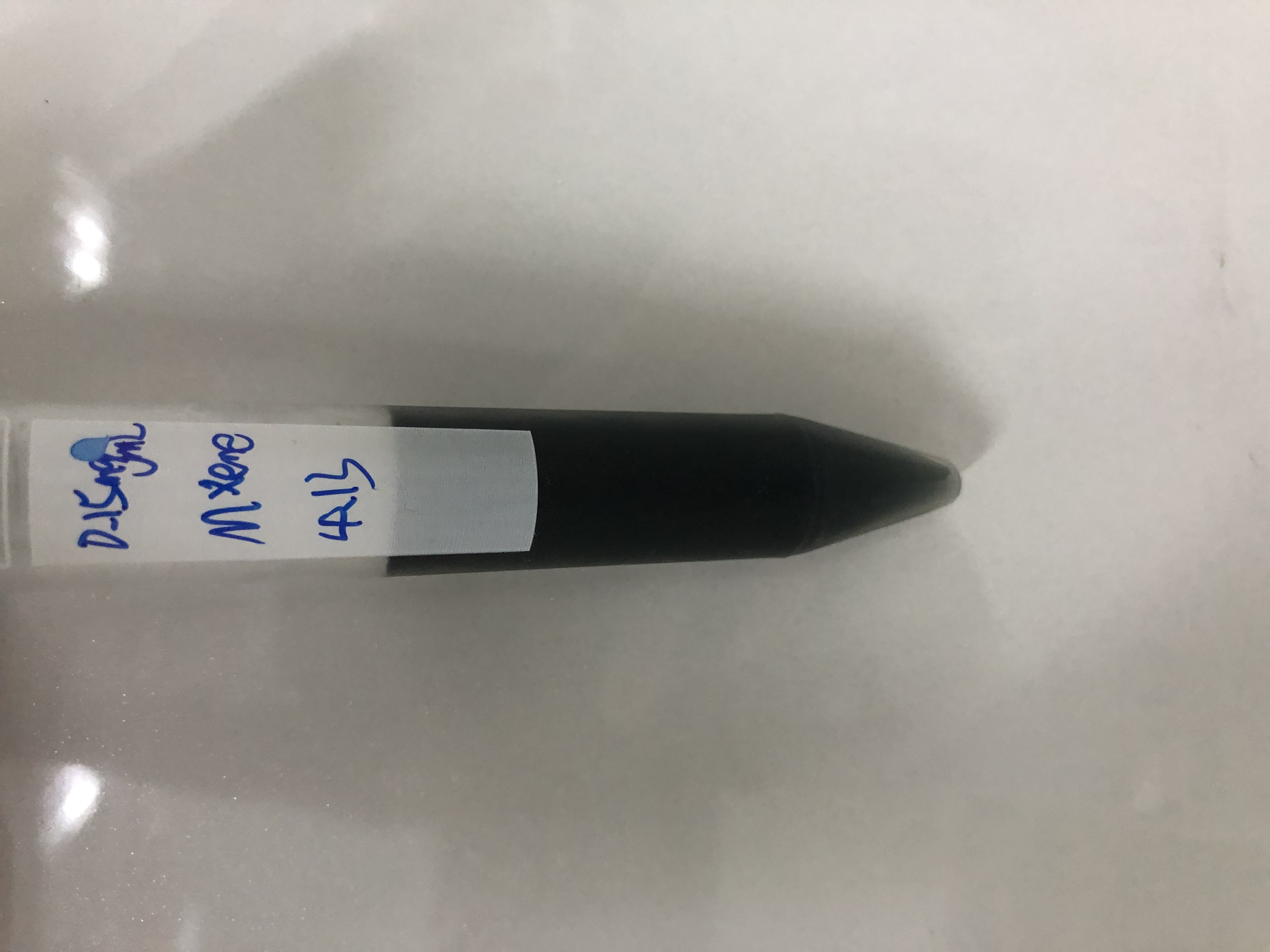

Supplement: Supplementary file 5 [file DataSheet2.ZIP › Physical map/Ti3C2Tx solution.jpg]

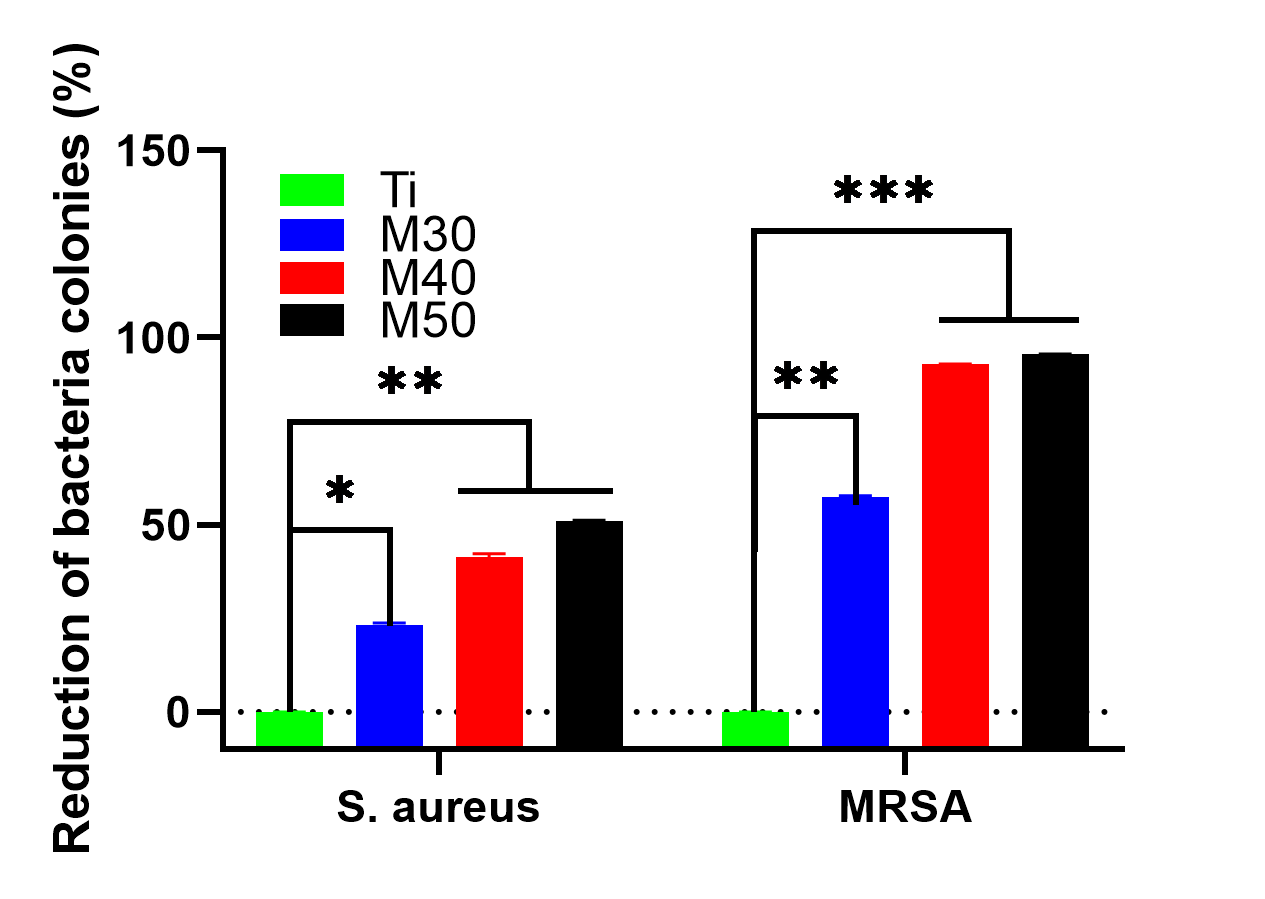

Supplement: Supplementary file 6 [file DataSheet5.ZIP › Reduction of bacterial colonies/Data 1.tif]

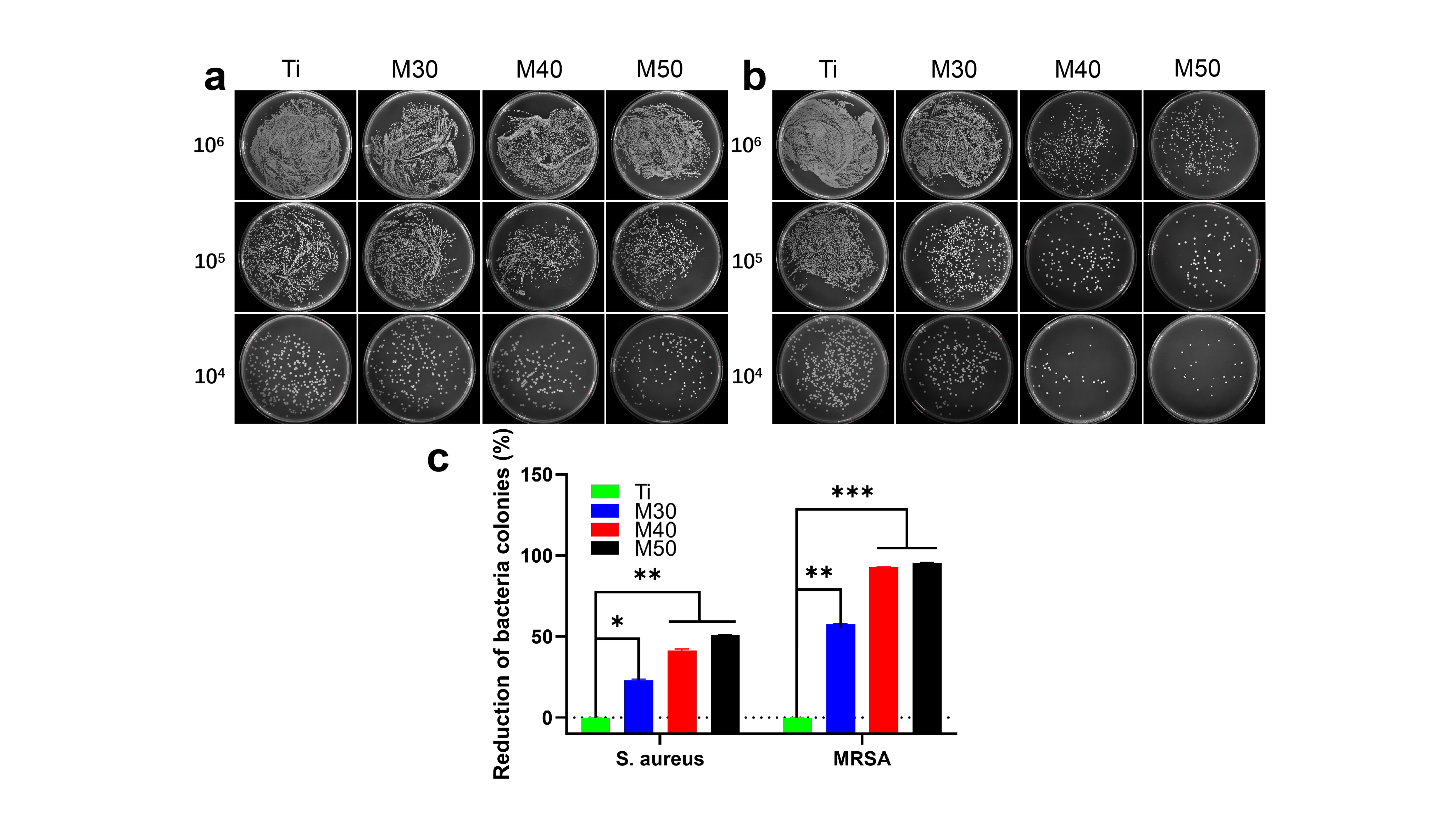

Supplement: Supplementary file 6 [file DataSheet5.ZIP › Reduction of bacterial colonies/Fig. 11..tif]
